# Pooled prevalence and subgroup variations of Tetralogy of Fallot among children and adolescents with congenital heart defect in Sub-Saharan Africa: A systematic review and meta-analysis

**DOI:** 10.1371/journal.pone.0311686

**Published:** 2025-01-17

**Authors:** Biniam Endale Geleta, Abay Mulu

**Affiliations:** Department of Anatomy, School of Medicine, Addis Ababa University, Addis Ababa, Ethiopia; All India Institute of Medical Sciences, INDIA

## Abstract

**Background:**

Tetralogy of Fallot is one of the critical congenital heart defects needing intervention within the first year of life.

**Objective:**

This review aims to systematically assess the prevalence of Tetralogy of Fallot among children and adolescents with congenital heart defects in Sub-Saharan Africa from January 2000 to January 2024.

**Methods:**

All original observational studies focused on children and adolescent population diagnosed with congenital heart defects within Sub-Saharan Africa; reported the primary outcome of interest were included. Prisma guidelines were utilized to perform this systematic review and meta-analysis. Electronic databases including Medline (PubMed), Scopus, Google Scholar, and African Index Medicus were searched. A weighted inverse variance random-effects model was employed to estimate the pooled prevalence of Tetralogy of Fallot.

**Results:**

Thirty-one studies included encompassing a total of 11,265 participants from 15 Sub-Saharan African countries with representation from Southern (4 studies, 619 participants), Central (5 studies, 2,220 participants), Eastern (11 studies, 3,384 participants), and Western (11 studies, 5,042 participants). Cross-sectional studies comprised (25 studies, 8,909 participants), and cohort design (6 studies, 2,356 participants). The analysis revealed a pooled prevalence of 10% (95% CI: 9%; 12%) with I^2^ (77%, p-value < 0.01). The subgroup analysis based on geographic regions revealed statistically significant difference.

**Conclusions and future implications:**

The prevalence of Tetralogy of Fallot observed was found considerably higher compared to global estimate and reports of developed countries. In a subgroup analysis based on the geographic region, a surprisingly high prevalence was reported across all regions of Sub-Saharan Africa. The substantial disparities and high prevalence observed underscores the complex interplay of factors influencing occurrence of Tetralogy of Fallot. Identifying the true scope of Tetralogy of Fallot burden may help policymakers and healthcare providers to prioritize interventions, optimize resource allocation, and potentially improve its outcomes in Sub-Saharan Africa.

## Introduction

Congenital heart disease (CHD) is one of the most common birth defects worldwide. Its global incidence is estimated at 8–12 per 1000 live births [1]. Tetralogy of Fallot (TOF) is one of critical CHDs needing intervention within the first year of life. It is the most common type of cyanotic CHD characterized by combination of multiple cardiac lesions including ventricular septal defect, pulmonary stenosis, overriding aorta, and right ventricular hypertrophy [2, 3].

While extensively studied in developed regions, studies focusing on TOF in developing regions, particularly the Sub-Sahara Africa is scarce [4]. There are multiple responsible factors for the development of TOF. These include chromosomal disorders, maternal uncontrolled diabetes, maternal rubella, and maternal intake of retinoic acid. There are also chances of recurrence in about 3% of cases [5]. About 25% of patients with TOF have one form of chromosomal disorder. The common chromosomal disorders associated with TOF are trisomy-13, trisomy-18, trisomy-21, micro-deletion at 22q11.2 and others including mutations in transcription factors like NKX2.5 [6].

Limited resources in low- and middle-income countries (LMICs) create a significant burden of CHD, including TOF. Early diagnosis and intervention are crucial for this critical condition, while these regions often face significant challenges due to a lack of both resources and expertise [7]. Healthcare access in Sub-Saharan Africa is very low, with an estimated pool of 42.6%. There is also a variation noted with the highest estimate of healthcare access for Southern region and lowest for Central region [8].

In developed countries, around 50% of TOF cases are diagnosed prenatally, often as early as 12 weeks of gestation [5, 6]. Early diagnosis and repair within the first year of life is recommended to preserve heart function and prevent life-threatening complications [9]. Unfortunately, such timely interventions are often unavailable and restricted in Sub-Saharan regions due to both resource limitations and a lack of expertise, which may lead to increased morbidity and mortality [7].

While TOF ranks among the leading cyanotic congenital heart defects, and top five CHDs worldwide, its prevalence varies significantly across different regions. Globally, it accounts for 3% to 5% of all CHD cases, with an estimated incidence of 2.8 to 3.4 per 10,000 live births [2, 10].

Developed countries like the United States (US) reported a birth prevalence of 3.9 per 10,000 live births, with an estimated 1,660 babies born with TOF each year [2, 11]. In the US, TOF also represents approximately 6.7% of all CHDs [2]. Highly populous Asian country, China presents a different magnitude. While the overall birth prevalence of CHD is reported as 9 per 1,000 live births, major eastern cities show a range of 3 to 8.03 per 1,000 live births. Notably, 30% of these cases are critical CHD like TOF requiring early intervention [12, 13]. Another two studies from China reported 5.25% and 5.3% prevalence of TOF among CHD cases [13, 14]. Data from the European population-based consortium registry reported a significant increase in TOF prevalence from 2004 to 2012 [15]. A Spanish study identified TOF as the most common cyanotic CHD, with a birth prevalence of 0.41% [16]. Meanwhile, a 15-year Norwegian study reported a TOF prevalence of 2.1% among CHD cases [17].

While the global picture of TOF is gradually becoming clearer, it remains unclear in Sub-Saharan Africa. Limitation of literature makes it difficult to establish a clear burden of TOF in the region [4]. World health organization estimates 1% of live births from LMIC, particularly Sub-Saharan Africa region are diagnosed by CHD. Unfortunately, only 10% of them get access to proper health care service [18].

The burden of TOF varies significantly across different African regions. Notably, many regions in Sub-Saharan Africa exhibit a higher prevalence of TOF compared to global estimates and reports from North America, Europe, and Asia [2, 10, 12, 13, 17]. Studies from West Africa (Nigeria, Niger, and Senegal) reported TOF prevalence ranging from 7% to 15% among all CHD cases [4, 19–28]. Similar trends are observed in Central Sub-Saharan Africa (Cameroon, Republic of the Congo) with a range of 6% to 13% [29–33]. Studies from the southern region, Malawi and Zimbabwe highlights a higher report with 18% and 19.6% proportion among children and adolescent populations with CHD, respectively [34–36].

Eastern region showed a TOF prevalence comparable to many other Sub-Saharan regions. Ethiopia, the most populous country, reported a range of 5.1% to 15.8%, and Tanzania (11.2%) [37–41]. Sudan exhibited a higher proportion at 13.3% to 14.1% [42, 43]. Other Eastern African countries like Somalia, Uganda, and Kenya reported proportions of 5%, 5%, and 8.4%, respectively [44–46]. Contrasting to this, studies from North Africa (Egypt, Libya) presented a distinguishing picture with a reported low prevalence of 0.5% to 3.5% [47–49].

Generally, previous studies show significant variations in TOF prevalence across different geographic regions. These geographic disparities might be linked to several factors, including underlying genetic predisposition, environmental exposures, and socioeconomic inequalities. Understanding these variations is crucial for informing the development of effective healthcare strategies tailored to each region’s unique burden.

While TOF is a recognized CHD globally, data on its prevalence in Sub-Saharan Africa remains scarce. Existing studies often report TOF cases as a proportion within the broader CHD population in the region. However, these studies are scattered and have not been systematically analyzed to provide a comprehensive picture of TOF prevalence across Sub-Saharan Africa. This lack of a pooled estimate hinders the ability to accurately assess the true burden of TOF in this region.

Given the limited healthcare resources available in Sub-Saharan Africa, particularly for complex surgeries like those often required for TOF, understanding the pooled prevalence of this condition is crucial. By identifying the true scope of the TOF burden, policymakers and healthcare providers can prioritize interventions, optimize resource allocation, and potentially improve outcomes of TOF in Sub-Saharan Africa.

This review aims to systematically assess the prevalence of TOF among children and adolescents with CHD in Sub-Saharan Africa from January 2000 to January 2024. It evaluates the available literature to estimate the overall prevalence, and sex distribution of TOF among CHD patients.

## Methods

### Study design and setting

This study employed a systematic review and meta-analysis method to estimate the pooled prevalence of TOF among children and adolescents with CHD in Sub-Saharan Africa. The review focused on the Sub-Saharan African region, encompassing countries located entirely or partially south of the Sahara Desert. To ensure comprehensive geographic coverage, we included Sudan and Somalia based on World Bank reason despite their geographic exclusion. We also included Djibouti, acknowledging its geographic location in Sub-Saharan Africa while recognizing its potential exclusion by organizations like World Bank [50].

### Systematic search strategy

A comprehensive search strategy was employed to identify relevant studies. Electronic databases including Medline (PubMed), Scopus, Google Scholar, and African Index Medicus were searched. To ensure comprehensiveness, the reference lists of included articles were also manually searched for additional relevant publications. The search was conducted on January 5, 2024.

This search strategy combined key terms and Medical subject headings (MeSH). The search strategy only utilized keywords across three databases: Google Scholar, Scopus, and African Index Medicus. These keywords addressed terms related to prevalence: “prevalence, proportion, pattern, epidemiology, occurrence, frequency, magnitude, and burden”. The congenital heart defect related keywords: “congenital anomaly*, birth defect*, birth anomaly*, congenital abnormality*, congenital heart disease, CHD, cardiac congenital anomaly, cyanotic heart disease”. Keywords specific to TOF were “Tetralogy of Fallot and TOF”. Finally, the geographic focus was addressed using the terms Sub-Saharan Africa and the names of each individual country within the region. Boolean operators ("AND" and "OR") were used to combine these keywords effectively.

For Medline/PubMed specifically, the search utilized combined relevant MeSH and keywords above. The MeSH utilized include: "Prevalence"[Mesh], AND "Congenital Abnormalities"[Mesh] OR "Heart Defects, Congenital"[Mesh], OR "Tetralogy of Fallot"[Mesh] AND "Africa South of the Sahara"[Mesh]. Boolean operators ("AND" and "OR") were used to combine these MeSH and keywords effectively (S1 File).

### Study selection process

#### Inclusion criteria

All original observational studies (case-control, cohort, or cross-sectional) focused on children and adolescent population (an age range of 0 to 18 years) diagnosed with CHD within Sub-Saharan Africa (considering both geographic definition and economic classifications); reported the primary outcome of interest; and published in English after 2000 were included in this systematic review and meta-analysis.

#### Exclusion criteria

Studies not focusing on children and adolescents with CHD, conducted outside Sub-Saharan Africa, published before 2000, not reporting TOF prevalence and total CHD cases, or employing non-original research designs (case reports, case series, review articles, and conference abstracts) were excluded. Additionally, studies in languages other than English, non-human studies, limited accessibility, those with significant methodological limitations including selection bias, inadequate data collection methods, or lacking echocardiographic confirmation of TOF were also excluded.

#### Study screening

Two reviewers (BG and AM) independently screened all retrieved references based on title and abstract. The same reviewers independently assessed the full texts of potentially eligible studies. Disagreements were resolved by discussion during the review.

### Data extraction

A pre-defined data extraction form was created in Microsoft Excel. This form included columns for author name, year of publication, country, region, study design, economic classification, reported cases of TOF, total cases of CHD, and sex distribution. Two independent reviewers (BG and AM) utilized the Excel spreadsheet to extract data from the included studies. Any discrepancies arising during data extraction were resolved through discussions. In cases where data were incomplete, we standardized the analysis by calculating the prevalence of TOF among all cases of CHD for which data were more consistently reported, even if some studies only provided the number of TOF cases.

### Quality assessment

To assess the methodological quality of the included studies, two independent reviewers (BG and AM) employed the Joanna Briggs Institute (JBI) quality appraisal checklist specifically designed for observational studies. Any discrepancies in quality scores between the reviewers were resolved through discussions. Studies scoring above half on all criteria within their respective designs were classified as low risk or good quality. Conversely, studies scoring below half were categorized as high risk or poor quality [51].

### Data synthesis

#### Quantitative synthesis (Meta-analysis)

A meta-analysis was conducted to estimate the overall pooled prevalence of TOF. This involved statistically pooling data from the included studies. The proportion of TOF in each study was calculated. These data were then combined across studies using meta-analysis techniques.

#### Heterogeneity assessment

We examined the heterogeneity of effect size using I^2^ statistics. The I^2^ statistic value of zero indicates true homogeneity, whereas the values 25 to 50%, 50 to 75%, and above 75% represent low, moderate, and high heterogeneity, respectively [52]. Appropriate statistical model was employed based on value of this heterogeneity assessment.

#### Subgroup analysis

To further investigate potential sources of variation in the reported prevalence of TOF, subgroup analyses were conducted. The included studies were systematically categorized based on key characteristics. This involved examining geographic location within Sub-Saharan Africa to identify potential variations attributable to regional factors. Additionally, study design was evaluated to assess whether research methodology introduced any biases in prevalence estimates. Furthermore, publication year comparisons allowed investigation of potential changes in TOF prevalence over time. Finally, World Bank income level categories were used to explore the potential association between a country’s economic status and TOF prevalence. This multifaceted approach to subgroup analyses allows for a more detailed understanding of the variations observed in reported TOF prevalence across Sub-Saharan Africa.

#### Publication bias assessment

The potential for publication bias was evaluated using two methods. First, a funnel plot was visually inspected for asymmetry. Second, Egger’s regression test was employed for a more objective assessment of publication bias.

#### Sensitivity analysis

Sensitivity analysis was conducted to explore the influence of single individual studies on the combined effect size. This was done by systematically removing each study one at a time and recalculating the pooled prevalence estimate to evaluate the influence of any single study on the overall results.

#### Presentation of the result

Results depicted visually through tables and forest plots. While the data in the forest plot is displayed as proportions for accuracy, we converted them to percentages for the presentation in the results and discussion sections. This conversion facilitates clearer comparison with findings reported as percentages in other relevant literatures.

### Statistical methods

Statistical analyses were conducted using R software, version 4.3.3. A meta-analysis was performed by utilizing "meta" package in R, employing a random-effects model to estimate the overall pooled prevalence of TOF. Heterogeneity test, publication bias assessment and sensitivity analysis were also performed using functions within R software. A meta-regression analysis was also conducted to explore if sample size influences the observed effect sizes.

## Results

### Search results

The literature search identified a total of 3,735 records from various electronic databases and cross references: PubMed (Medline) (3,575), Google Scholar (124), African Index Medicus (10), Scopus (2), and other sources (24). These records reported the prevalence of TOF among children and adolescents with CHD in Sub-Saharan Africa.

Duplicates (234) were removed using EndNote^™^ reference management software. After initial title and abstract screening, an additional 3,422 articles were excluded for not meeting the inclusion criteria for this review.

**Fig 1:**
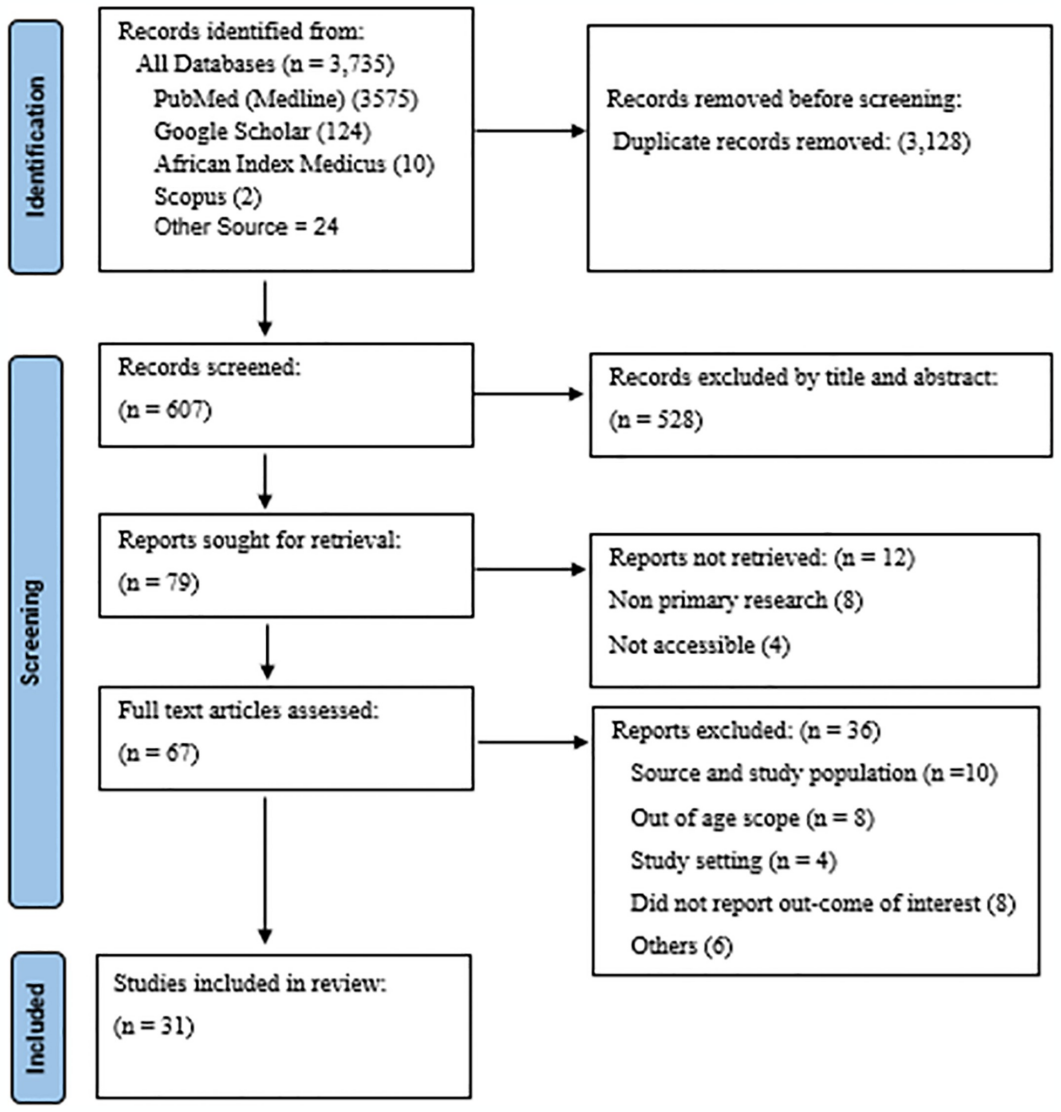
PRISMA (Preferred Reporting Items for Systematic Reviews and Meta-Analyses) flow diagram. Results of search and reasons for exclusion of studies for TOF prevalence review in Sub-Saharan Africa [53].

Following retrieval of 79 potentially relevant articles, a further 12 articles were excluded during full text review. Finally, after assessing 67 full text articles, 36 additional articles were excluded. A total of 31 studies ultimately met the eligibility criteria and were included in this systematic review and meta-analysis (Fig 1).

### Characteristics of included articles

All 31 studies included in this systematic review and meta-analysis utilized either a cross-sectional or cohort design. Cross-sectional studies comprised the majority of included studies (25 studies, 8,909 participants), with the remaining studies employing a cohort design (6 studies, 2,356 participants) (Table 1).

**Table 1:** Characteristics of included studies on TOF prevalence in Sub-Saharan Africa (2006–2023).

Author(s) Name	Year	Country	Region	Study Design	Total CHD	TOF Cases
Mazhani et al	2020	Botswana	South	Cross sectional	170	11
Puepi et al	2022	Cameroon	Central	Cross sectional	77	10
Nkoke et al	2017	Cameroon	Central	Cross sectional	227	14
Kamdem et al	2018	Cameroon	Central	Cross sectional	370	29
Chelo et al	2016	Cameroon	Central	Cross sectional	1230	102
Loufoua-Lemay et al	2016	Congo	Central	Cohort	316	32
Massoure et al	2013	Djibouti	East	Cohort	27	3
Mehadi et al	2006	Ethiopia	East	Cross sectional	177	9
Talarge et al	2018	Ethiopia	East	Cross sectional	97	11
Malede et al	2006	Ethiopia	East	Cross sectional	76	12
Tsega et al	2022	Ethiopia	East	Cross sectional	228	22
Awori	2013	Kenya	East	Cross sectional	214	18
Puri et al	2018	Malawi	South	Cohort	24	3
Kennedy et al	2013	Malawi	South	Cross sectional	139	25
Zakaria et al	2021	Niger	West	Cross sectional	167	16
Chinawa et al	2013	Nigeria	West	Cross sectional	71	9
Ekure et al	2009	Nigeria	West	Cross sectional	73	9
Adebayo et al	2016	Nigeria	West	Cross sectional	192	22
Otaigbe et al	2014	Nigeria	West	Cohort	332	28
Sadoh et al	2013	Nigeria	West	Cross sectional	605	48
Chinawa et al	2021	Nigeria	West	Cross sectional	367	51
Ekure et al	2018	Nigeria	West	Cross sectional	767	103
Ekure et al	2017	Nigeria	West	Cross sectional	1296	155
Animasahun et al	2016	Nigeria	West	Cross sectional	1123	165
Coundoul et al	2023	Senegal	West	Cross sectional	49	5
Yusuf et al	2021	Somalia	East	Cross sectional	160	8
Ibrahim et al	2012	Sudan	East	Cross sectional	143	19
Abdelrahman et al	2022	Sudan	East	Cross sectional	596	84
Zuechner et al	2019	Tanzania	East	Cohort	1371	154
Aliku et al	2021	Uganda	East	Cross sectional	295	15
Bannerman et al	2020	Zimbabwe	South	Cohort	286	56

This review encompassed 15 Sub-Saharan African countries, with representation from all 4 geographic regions: Southern (4 studies, 619 participants), Central (5 studies, 2,220 participants), Eastern (11 studies, 3,384 participants), and Western (11 studies, 5,042 participants) (Table 1). Publication dates ranged from January 2006 to September 2023. Notably, no study published between 2000 and 2005 was included. Three studies with a total sample size of 326 were published between 2006 and 2011. The publication years for the remaining studies were: 2012–2017 (13 studies, 5,915 participants) and 2018–2023 (15 studies, 5,024 participants) (Table 1).

In terms of income level, 1 study (170 participants) was from a country with upper-middle-income economy, 19 studies (8,993 participants) were from lower-middle-income, and 11 studies (2,102 participants) were from low-income countries (S1 Table).

While our systematic review included 31 studies, data on the sex distribution of TOF was only available in 10 studies, representing a total of 3,073 participants (Table 2).

**Table 2:** Sex distribution of TOF in Sub-Saharan African studies included in the review.

Author	Year	Country	Region	Design	CHD	TOF	Male	Female
Kamdem et al	2018	Cameroon	Central	Cross sectional	370	29	20	9
Loufoua-Lemay et al	2016	Congo	Central	Cohort	316	32	20	12
Massoure et al	2013	Djibouti	East	Cohort	27	3	2	1
Malede et al	2006	Ethiopia	East	Cross sectional	76	12	4	8
Sadoh et al	2013	Nigeria	West	Cross sectional	605	48	30	18
Animasahun et al	2016	Nigeria	West	Cross sectional	1123	165	99	65
Coundoul et al	2023	Senegal	West	Cross sectional	49	5	3	2
Mazhani et al	2020	Botswana	South	Cross sectional	170	11	6	5
Mehadi et al	2006	Ethiopia	East	Cross sectional	177	9	5	4
Yusuf et al	2021	Somalia	East	Cross sectional	160	8	6	2

### Quality assessment

The JBI critical appraisal checklists were employed to evaluate the methodological quality of studies. Cohort studies (n = 6) were assessed using the JBI checklist for cohort designs, achieving scores between 6 and 8 out of 11 points. Strengths commonly observed in cohort studies included clear inclusion/exclusion criteria and well-defined exposure measures. However, limitations identified across some cohorts included potential for selection bias during recruitment and incomplete follow-up data.

Cross-sectional studies (n = 25) underwent assessment with the JBI checklist for prevalence studies, scoring between 5 and 8 out of a possible 9 points. A common strength in cross-sectional studies was the use of standardized data collection instruments. However, limitations included the inability to establish causal relationships due to the study design and potential for measurement bias if instruments lacked reliability.

For instance, one high-scoring cohort study implemented strong blinding of participants and researchers to minimize bias, while a lower-scoring cohort had limitations in following participants over time. Conversely, a high-scoring cross-sectional study utilized a well-validated questionnaire, whereas a lower-scoring study relied on a less reliable self-reported measure. Due to the large number of studies (31), a detailed assessment of each study is not presented here, but the score range provides a general overview of methodological quality.

### Meta-analysis

**Fig 2:**
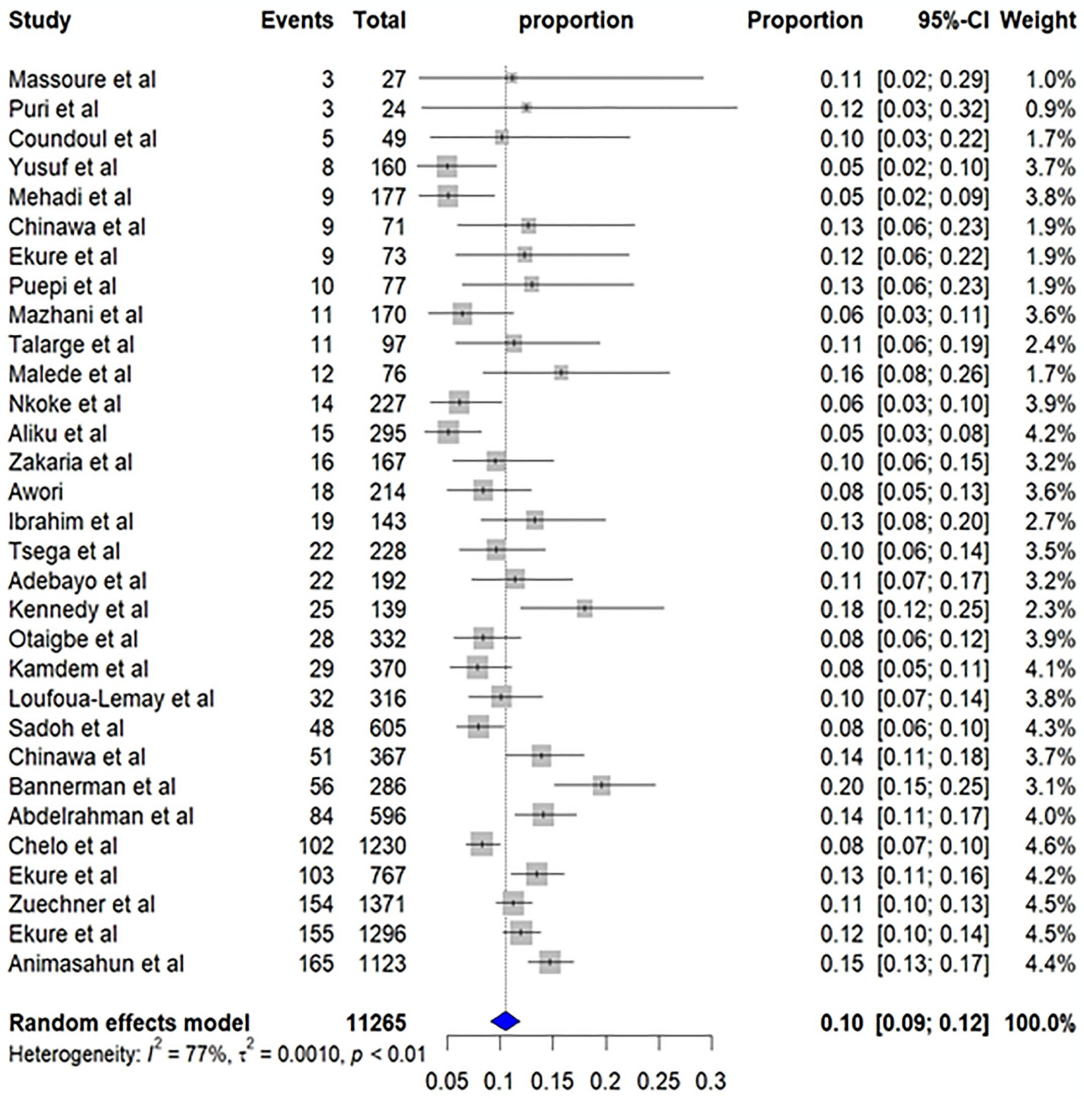
Forest plot of overall pooled prevalence of Tetralogy of Fallot in Sub-Saharan Africa.

Thirty-one studies encompassing a total of 11,265 CHD cases in children and adolescents from 15 Sub-Saharan African countries were included in the meta-analysis to estimate the pooled prevalence of TOF. The analysis revealed a pooled prevalence of 10% (95% CI: 9%; 12%). However, substantial heterogeneity was detected across the studies, indicated by a high I^2^ statistic value (I^2^ = 77%, p-value < 0.01) (Fig 2). Due to this heterogeneity, a random-effects model was employed to estimate the pooled prevalence of TOF.

We also investigated sex distribution of TOF. Pooled estimates revealed a higher proportion of males, 5% (95% CI: 4%; 7%) compared to females, 3% (95% CI: 2%; 4%) among CHD cases of 10 studies reported sex distribution.

A meta-regression analysis investigated whether sample size influenced observed effect sizes. The results showed a positive coefficient (0.115), showing a positive trend where larger studies have large effect sizes. However, this trend was not statistically significant (p-value = 0.96). Therefore, sample size does not appear to be a significant source of heterogeneity in the effect sizes across the studies of this review.

### Subgroup analysis

**Fig 3:**
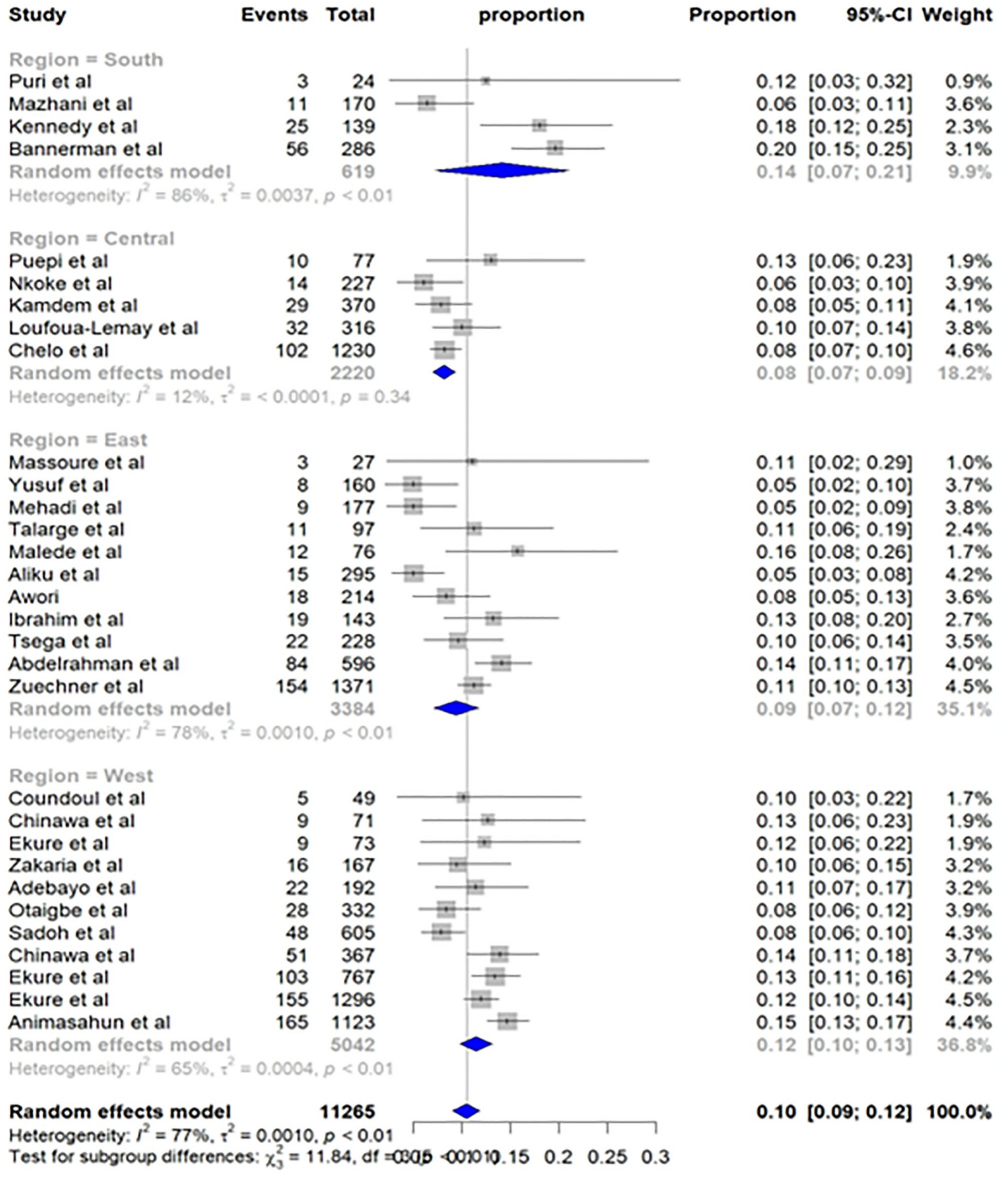
Subgroup analysis by region—Pooled prevalence of TOF in Sub-Saharan Africa.

The subgroup analysis revealed a significant variation (p-value = 0.01) in the prevalence of TOF across different geographic regions of Sub-Saharan Africa. The highest prevalence was observed in the Southern region, with an estimated 14% (95% CI: 7%; 21%). This was followed by the Western region, 12% (95% CI: 10%; 13%), Eastern Region, 9% (95% CI: 7%; 12%), and the Central region, 8% (95% CI: 7%; 9%) (Fig 3).

**Fig 4:**
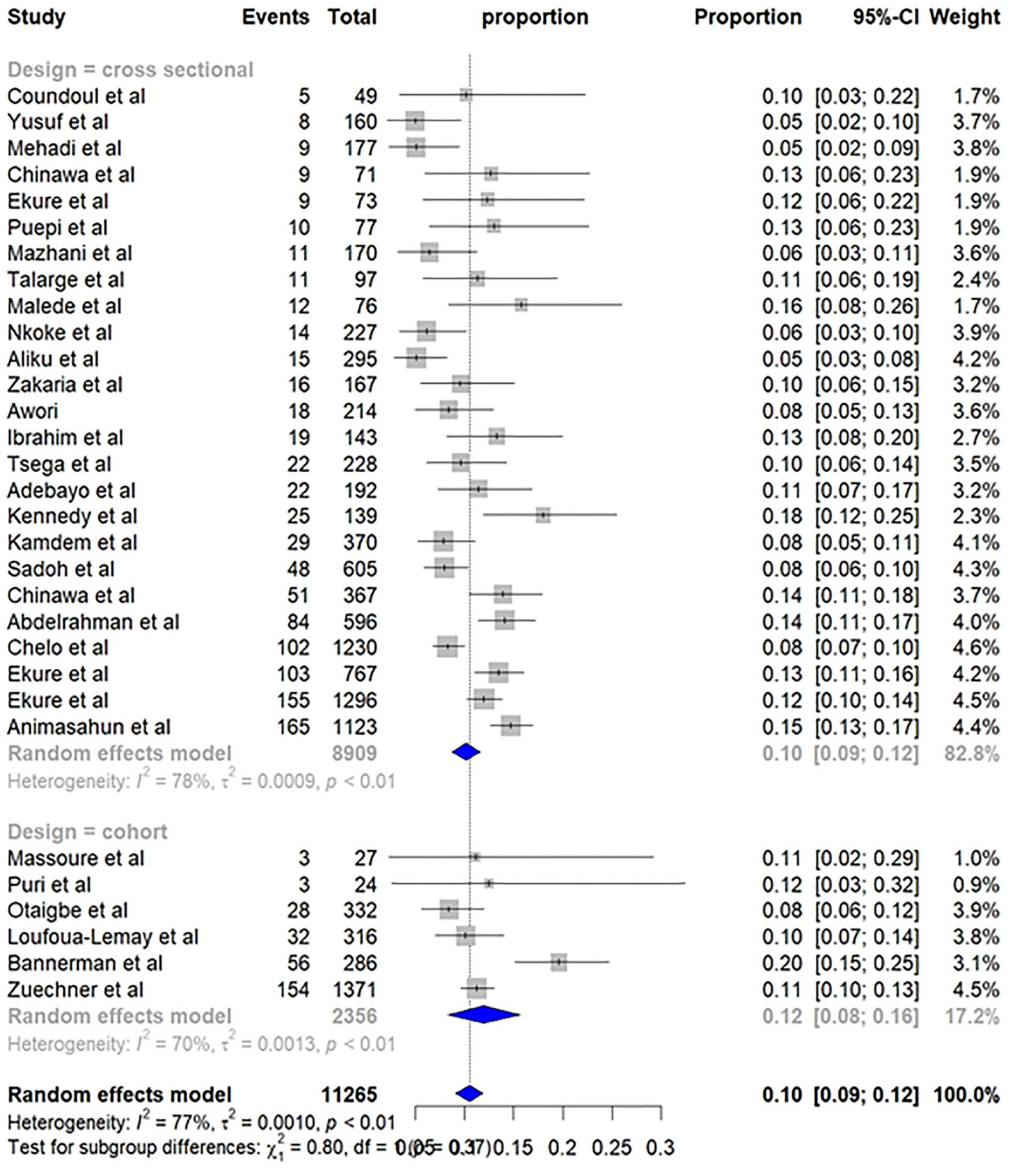
Subgroup analysis by study design—Pooled prevalence of TOF in Sub-Saharan Africa.

The prevalence of TOF was further examined by study design. Cross-sectional studies estimated a pooled prevalence of 10% (95% CI: 9%; 12%), while cohort studies yielded a higher estimate of 12% (95% CI: 8%; 16%). However, the statistical test did not detect a significant difference in prevalence between the two study designs (p-value = 0.37) (Fig 4).

**Fig 5:**
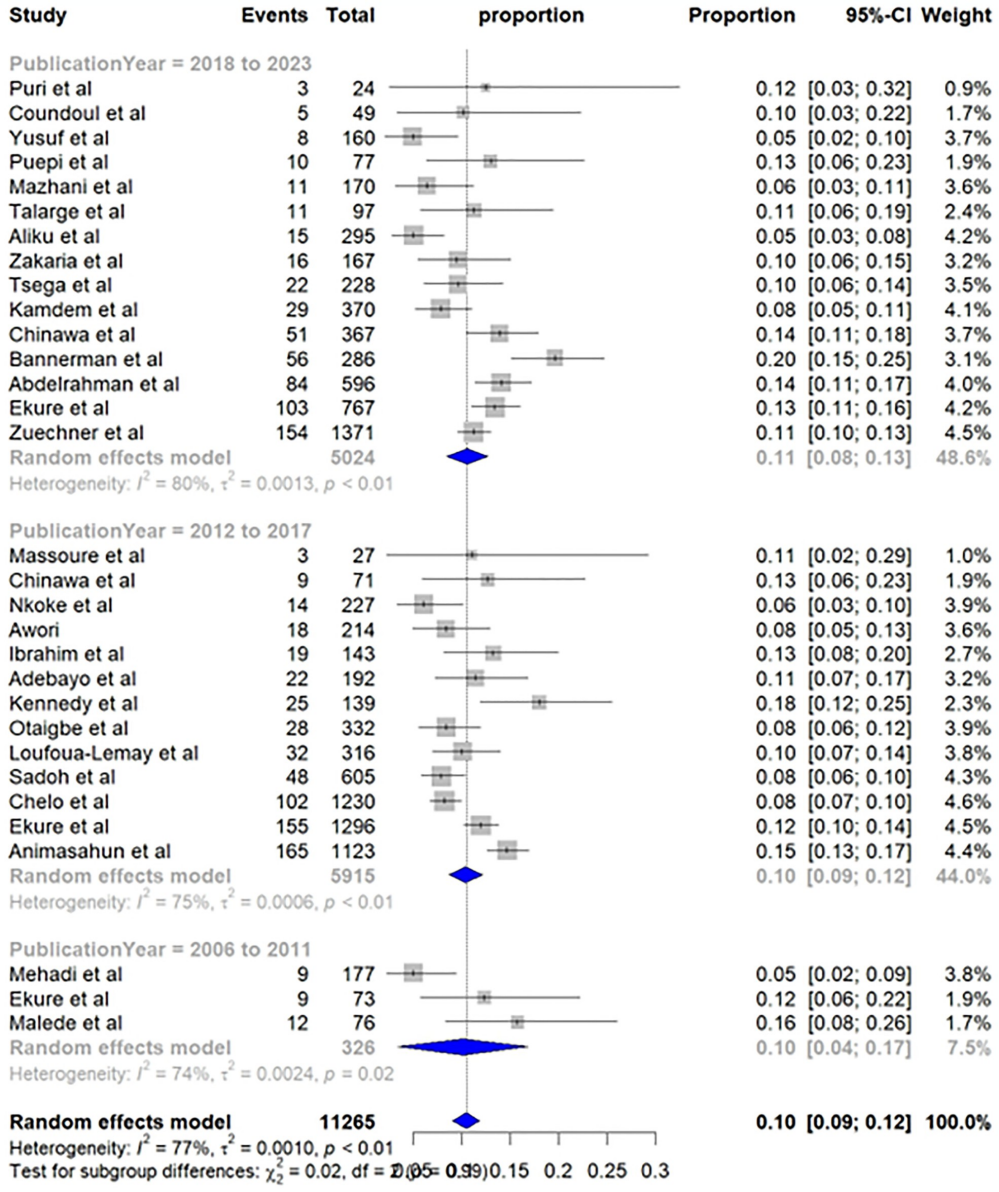
Subgroup analysis by publication year—Pooled prevalence of TOF in Sub-Saharan Africa.

Subgroup analysis based on publication year also revealed no statistically significant difference (p-value = 0.98). Studies published between 2018 and 2023 reported slightly higher prevalence, with an estimated 11% (95% CI: 8%; 13%). This was followed by studies published between 2012 and 2017, 10% (95% CI: 9%; 12%), and 2006 to 2011, 10% (95% CI: 4%; 17%) (Fig 5).

**Fig 6:**
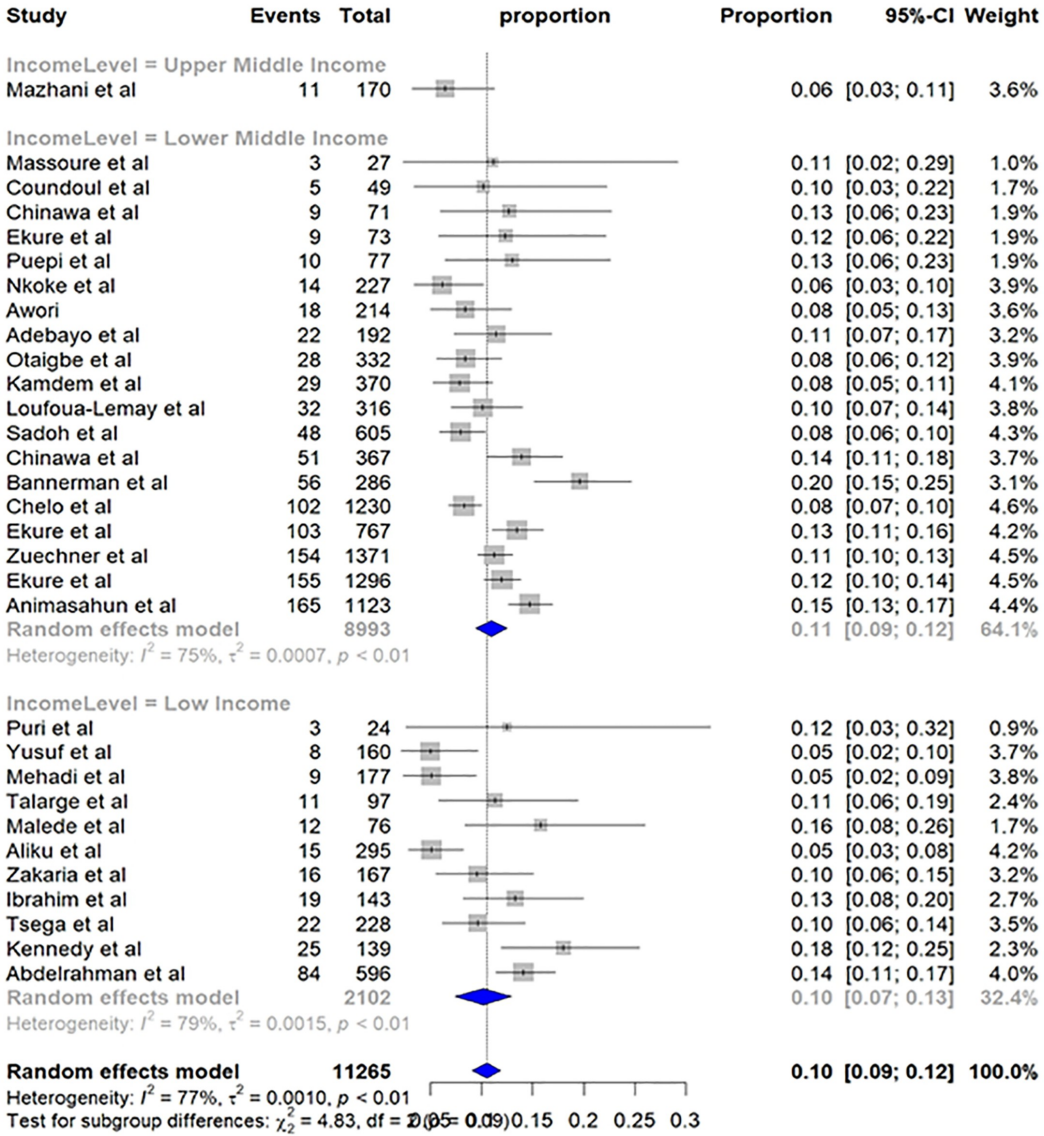
Subgroup analysis by income level—Pooled prevalence of TOF in Sub-Saharan Africa.

The subgroup analysis examining income level also did not identify a statistically significant difference in TOF prevalence between income categories (p-value = 0.08). Countries with lower-middle-income economies reported a slightly higher pooled prevalence of TOF (11%, 95% CI: 9%; 12%), compared to lower-income economies (10%, 95% CI: 7%; 13%) (Fig 6).

### Sensitivity analysis

To assess the robustness of our findings and ensure as they were duly influenced by any single study, we conducted a leave-one-out sensitivity analysis, by recalculating the pooled prevalence of TOF after sequentially omitting each study from the original analysis. The results demonstrated minimal variation in the pooled estimate, ranging from 10.16% (28) to 10.71% [35]. This suggests that our finding was not dependent on a single data source (Table 3).

**Table 3:** Results of leave-one-out sensitivity analysis for pooled prevalence of TOF in Sub-Saharan Africa.

Omitted Study	Estimate	95% Confidence Interval
1	0.1048	0.0912; 0.1183
2	0.1049	0.0913; 0.1185
3	0.1050	0.0913; 01187
4	0.1069	0.0935; 0.1203
5	0.1045	0.0909; 0.1182
6	0.1046	0.0909; 0.1183
7	0.1069	0.0935; 0.1203
8	0.1045	0.0908; 0.1181
9	0.1048	0.0910; 0.1186
10	0.1064	0.0927; 0.1201
11	0.1040	0.0904; 0.1175
12	0.1066	0.0930; 0.1202
13	0.1071	0.0938; 0.1204
14	0.1053	0.0914; 0.1192
15	0.1058	0.0919; 0.1197
16	0.1042	0.0905; 0.1179
17	0.1053	0.0914; 0.1193
18	0.1047	0.0908; 0.1186
19	0.1030	0.0897; 0.1163
20	0.1058	0.0919; 0.1198
21	0.1061	0.0922; 0.1200
22	0.1052	0.0912; 0.1192
23	0.1061	0.0922; 0.1200
24	0.1036	0.0899; 0.1173
25	0.1016	0.0890; 0.1141
26	0.1034	0.0897; 0.1170
27	0.1060	0.0921; 0.1200
28	0.1036	0.0899; 0.1174
29	0.1047	0.0906; 0.1188
30	0.1043	0.0903; 0.1183
31	0.1028	0.0894; 0.1163
**Combined**	**0.1049**	**0.0915; 0.1184**

### Publication bias

**Fig 7:**
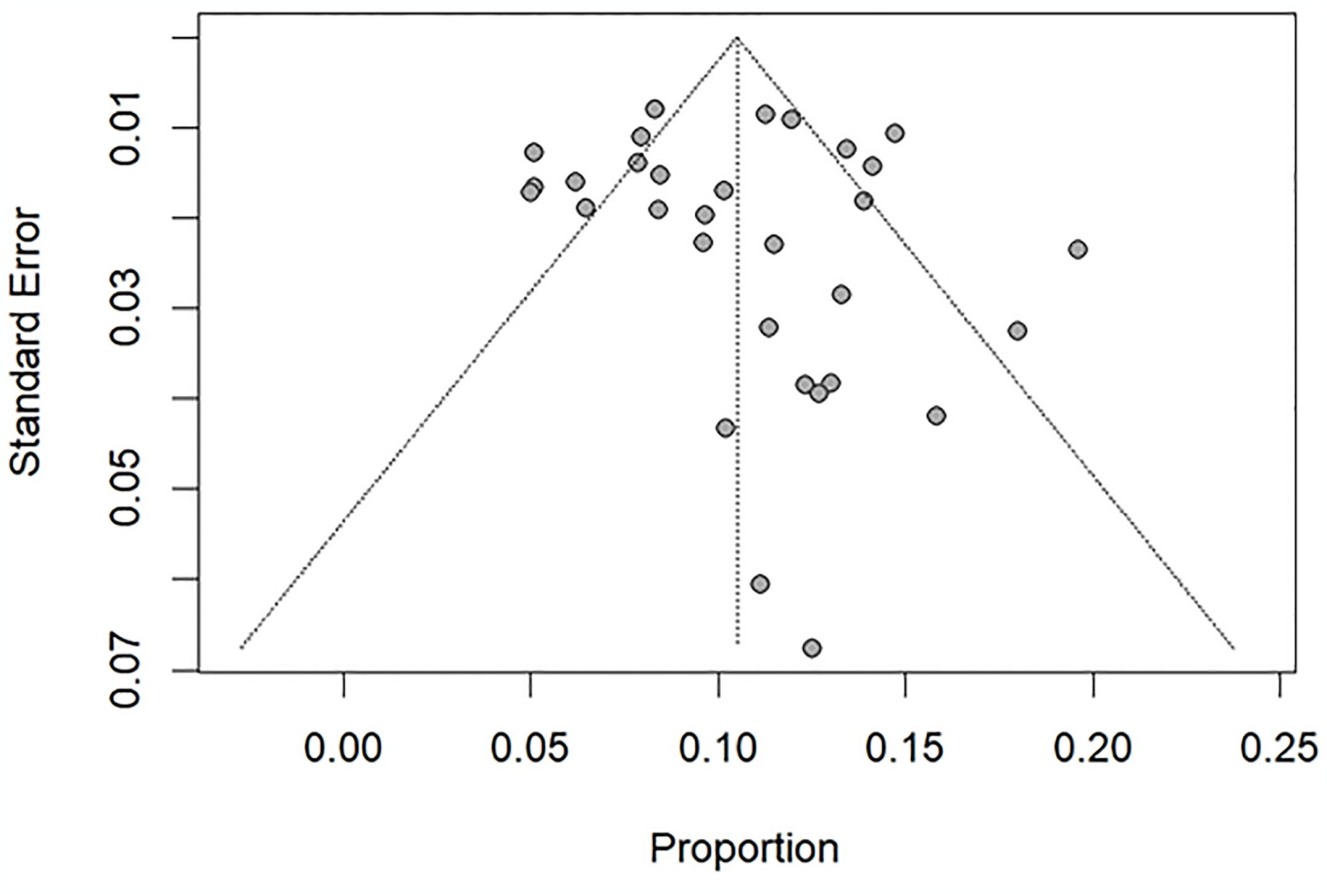
Funnel plot for assessment of publication bias of pooled TOF prevalence in Sub-Saharan Africa.

Visual inspection of the funnel plot revealed asymmetrical distribution (Fig 7). Which could potentially indicate publication bias. However, a statistical test for publication bias, Egger’s test yielded a non-significant p value (0.49). This may suggest that publication bias is unlikely to be a major concern in this analysis.

## Discussion

To our knowledge, this is the first meta-analysis conducted in sub-Saharan Africa that estimates the pooled prevalence of TOF among children and adolescents with CHD.

Our present study identified a 10% prevalence (95% CI: 9; 12%) of TOF among children and adolescents with CHD in Sub-Saharan Africa. This finding aligns with reports from West Africa, where studies in Senegal and Niger documented similar proportions of 10.2% and 9.6%, respectively [19, 28].

Despite limitations in healthcare access and diagnostic expertise in Sub-Saharan Africa, the prevalence observed in this region was found considerably higher compared to developed countries. Global estimates suggest a range of 3% to 5% [10]. Similarly, studies from China and the USA reported lower prevalence of 5.2% and 6.7%, respectively [2, 13, 14]. Furthermore, our findings are significantly higher than studies from Norway (2.1%), and North Africa (Egypt: 0.5%, and Libya: 2 to 3.5%) [17, 47–49]. The observed variations of our finding from developed regions highlights the potential influence of factors beyond healthcare access. Genetic variations, environmental factors, and socio-economic disparities might play a role for the higher TOF prevalence report of Sub-Saharan Africa.

While our findings align with some West African reports, a closer look reveals significant geographic variations within Sub-Saharan Africa. Studies conducted in Malawi and Zimbabwe, located in the southern region, reported considerably higher proportions of TOF, with approximately 18% and 20% of cases among children and adolescents with CHD, respectively [34–36]. In contrast, studies from East African countries like Somalia, and Uganda documented much lower proportions of 5% each [45, 46]. Despite this observed variations, a potentially higher burden of TOF is detected in the studied population.

In a subgroup analysis based on the geographic region, a surprisingly high prevalence was reported across all regions of Sub-Saharan Africa, exceeding global estimates and reports from developed countries [2, 10, 13, 14, 17]. Notably, the Southern region reported the highest prevalence, 14% (95% CI: 7%; 21%), followed by the Western region, 12% (95% CI: 10%; 13%). Significant variations also emerged within Sub-Saharan Africa. Regions with better healthcare infrastructure, like the Southern, generally had higher TOF prevalence compared to regions with poor healthcare systems, such as the Eastern (9%, 95% CI: 7%; 12%) and Central regions (8%, 95% CI: 7%; 9%). This observed pattern within Sub-Sahara may be due to a link between better healthcare service and increased chance of TOF diagnosis. Furthermore, other factors, potentially genetic or environmental, may also contribute to these variations.

The analysis also revealed variations based on the type of study design. While there was not a statistically significant difference overall, sub-groups of studies using a cohort design reported a higher pooled prevalence of TOF, 12% (95% CI: 8%; 16%) compared to studies with a cross-sectional design. Cross-sectional studies reported a prevalence of 10% (95% CI: 9%; 12%), which is in line with the overall pooled finding. This difference might be due to an imbalance in the number of studies included in each group, and inherited strict and prolonged observation window of patients in the cohort studies.

The analysis based on publication year also did not reveal a statistically significant difference. However, there is a worth considering trend in the pattern. Studies published between 2018 and 2023 reported a slightly higher proportion of TOF at 11% (95% CI: 8%; 13%). In contrast, studies published in earlier times, 2006–2011 and 2012–2017 reported a slightly lower prevalence at 10% (95% CI: 9%; 12%) and 10% (95% CI: 4%; 17%), respectively. This observed difference could be due to time-related improvement in health care infrastructures, and advancements in diagnosing abilities.

Subgroup analysis based on World Bank income level categories also did not reveal a statistically significant difference in TOF prevalence (p-value = 0.08). However, a trend emerged, with lower-middle-income economies exhibiting a slightly higher pooled prevalence of TOF, 11% (95% CI: 9%; 12%) compared to lower-income economies, 10% (95% CI: 7%; 13%). This difference may be attributed to variations in socioeconomic status, potentially influencing access to diagnostic tools or healthcare services that could impact TOF diagnosis rates.

Generally, this review and meta-analysis found a significantly high prevalence of TOF among children and adolescents with CHD in Sub-Saharan Africa. This high finding hold significant implications for policy makers and health system administrators, as strengthening healthcare infrastructure and expertise in diagnosing and managing this complex case across Sub-Saharan Africa is a pressing need.

Variations were also noted among the sub-populations of this region. The substantial disparities observed underscores the complex interplay of factors influencing its occurrence. While healthcare access differences might be linked to within region variations, other factors beyond healthcare access (genetic and environmental influences) likely play a role for a reported high prevalence.

The strength of this systematic review is its offering a valuable first attempt to synthesize existing data on TOF prevalence in the region. It provided a comprehensive initial picture by including 31 studies, encompassing all regions within the Sub-Sahara, and considering subgroup analysis. The review also employed a standardized quality assessment approach to strengthening its methodology.

However, there are some worth considering limitations in this review. Firstly, the exclusion of non-English articles might have limited the captured data since many articles in the region are by other languages like French. Secondly, the review faced a scarcity of studies directly reporting TOF prevalence, relying instead on studies investigating CHD prevalence where TOF cases were indirectly reported. Despite employing this approach being necessary due to limited data, it likely contributed to the observed heterogeneity. Third, the review has not considered grey literature sources. This may have further affected the comprehensiveness of our findings.

## Conclusion

This review identified a high prevalence (10%) of TOF among children and adolescents with CHD in Sub-Saharan Africa. This estimate significantly exceeds those reported in developed countries, highlighting the complex interplay of factors influencing TOF occurrence in the region.

Subgroup analysis also revealed a high prevalence across all Sub-Saharan African regions, even surpassing global estimates. Furthermore, regions with stronger economies and better healthcare infrastructure generally had higher reported TOF prevalence. While the healthcare infrastructure difference might contribute, this finding warrants further investigation into other potential reasons in the region.

These findings underscore the urgent need to explore the underlying causes of TOF in Sub-Saharan Africa. While economic and healthcare disparities may be relevant for variations noted within Sub-Sahara, factors beyond healthcare access, such as potential genetic or environmental influences, likely play a role for high prevalence reported. Understanding these complexities is crucial to improve strategies for prevention, diagnosis, and management of this complex defect in the region.

## Recommendations and future implications

In light of this study’s findings, future research may need to focus deeper into the potential causes behind the notably high burden of TOF reported in Sub-Saharan Africa. This may need to encompass the roles of geographic variations and associated factors, including genetic predisposition, environmental influences, and socioeconomic disparities. Exploring the observed disparities in TOF burden across different geographic regions within Sub-Saharan Africa is also crucial.

This high burden of TOF finding is especially important for policy makers and health system administrators in Sub-Saharan Africa. It highlights the urgent need to strengthen healthcare infrastructure and expertise in diagnosing and managing this complex case.

## Supporting information

S1 Table Detailed characteristics of included studies. (XLSX)

S2 Table All search results with reason of exclusion. (DOCX)

S3 Table Results of quality assessment. (DOCX)

S4 Table Extracted data. (XLSX)

S1 Checklist PRISMA 2020 checklist. (DOCX)

S1 File Search strategy. MeSH and keywords employed in different databases.
(DOCX)

S2 File Total references searched. (TXT)

S3 File Duplicate references removed. (TXT)

## Abbreviations

CDC: Center for Disease Control and Prevention
CHD: Congenital Heart Defect
JBI: Joanna and Briggs Institute
LMIC: Low- and Middle-Income Countries
TOF: Tetralogy of Fallot
US: United States
