# Supplementary material for: Pooled prevalence and subgroup variations of Tetralogy of Fallot among children and adolescents with congenital heart defect in Sub-Saharan Africa: A systematic review and meta-analysis
Source: PLoS One. 2025 Jan 17;20(1):e0311686. doi: 10.1371/journal.pone.0311686 (PMC11741593; doi:10.1371/journal.pone.0311686)
Supplement: S2 Table — (DOCX) [file pone.0311686.s002.docx]

| **Author** | **Publication Year** | | **Title** | | **Reason of exclusion** |
| --- | --- | --- | --- | --- | --- |
| 1. Kowero, E.; Burengelo, D.; Philbert, D.; Sirili, N. | 2023 | | "…with the third eye we can detect mistakes and correct them…” Supportive supervision and the management of Clubfoot by Ponseti method: Qualitative experiences from Mwanza, Tanzania | | unrelated topic |
| 1. Bialik, V.; Berant, M. | 1997 | | "Immunity" of Ethiopian Jews to developmental dysplasia of the hip: a preliminary sonographic study | | Year of publication |
| 1. Nabatanzi, M.; Seruwagi, G. K.; Tushemerirwe, F. B.; Atuyambe, L.; Lubogo, D. | 2021 | | "Mine did not breastfeed", mothers' experiences in breastfeeding children aged 0 to 24 months with oral clefts in Uganda | | unrelated topic |
| 1. Kierdorf, H.; Witzel, C.; Kierdorf, U.; Skinner, M. M.; Skinner, M. F. | 2015 | | "Missing perikymata"–fact or fiction? A study on chimpanzee (Pan troglodytes verus) canines | | unrelated topic |
| 1. Marbán-Castro, E.; Enguita-Fernàndez, C.; Romero-Acosta, K. C.; Arrieta, G. J.; Marín-Cos, A.; Mattar, S.; Menéndez, C.; Maixenchs, M.; Bardají, A. | 2022 | | "One feels anger to know there is no one to help us!". Perceptions of mothers of children with Zika virus-associated microcephaly in Caribbean Colombia: A qualitative study | | unrelated topic |
| 1. Hlongwa, P.; Rispel, L. C. | 2018 | | "People look and ask lots of questions": caregivers' perceptions of healthcare provision and support for children born with cleft lip and palate | | unrelated topic |
| 1. Hard, G. C.; Atkinson, F. F. | 1967 | | "Slobbers" in laboratory guinea-pigs as a form of chronic fluorosis | | Year of publication |
| 1. Zohra, R.; Song, M. S.; Iliham, N.; Dolikun, M. | 2016 | | [A comparative study on characterizations of genetic recombination hotspots in PPARG gene between Kirgiz and Uyghur ethnic groups in Xinjiang] | | unrelated topic |
| 1. Armstrong, O.; Karayuba, R. | 1993 | | [A rare cause of intestinal obstruction revealed during pregnancy (Kamenge University Hospital Center, Bujumbura, Burundi)] | | Year of publication |
| 1. Kimbally-Kaky, G.; Makoumbou, P.; Nzingoula, S. | 2002 | | [Acute rheumatic fever among children in the Republic of Congo: report of 56 cases] | | unrelated topic |
| 1. Eckes, L. | 1976 | | [Altitude adaptation. V. (conclusion). Morbidity and mortality. Literature] | | Year of publication |
| 1. Saka, B.; Kombaté, K.; Mouhari-Toure, A.; Akakpo, S.; Djeha, A.; Pitché, P.; Tchangaï-Walla, K. | 2010 | | [An acrodermatitis-enteropathica-like eruption in an exclusively breast-fed premature infant] | | unrelated topic |
| 1. Retief, A. E.; Kotzé, G. M.; Fox, M. F.; Venter, P. A.; Van Zyl, J. A.; Van Niekerk, W. A. | 1979 | | [An analysis of cytogenetic studies of 2334 patients] | | Year of publication |
| 1. Yue, Q.; Wang, H.; Zhang, B.; Zhao, K. P. | 2014 | | [Analysis of the HapMap data on SNPs in SUMO1 and association study of rs7599810 in trios with non-syndromic cleft lip with or without cleft palate] | | unrelated topic |
| 1. Kouam, L.; Kamdom-Moyo, J. | 1994 | | [Anencephaly associated with hydramnios. A case diagnosed late by ultrasonographic examination in the third trimester of pregnancy] | | Year of publication |
| 1. Amadou, A.; Sonhaye, L.; Douaguibe, B.; Tchaou, M.; Agoda-Koussema, L. K.; Etteh Adjenou, K.; N'Dakena, K. | 2013 | | [Anencephaly in twin pregnancy: a series of 6 cases] | | unrelated topic |
| 1. Tshibangu, K.; Sengeyi, M. A.; Tozin, R.; Nguma, M. | 1989 | | [Anencephaly in underdeveloped countries. Apropos of 22 cases in Kinshasa, Záĭre] | | Year of publication |
| 1. Iioki, L. H.; Azika, M. E.; Ikourou-Yoka, P.; Bouramoue, V. | 1993 | | [Anencephaly. 10 cases seen in 3 years in Brazzaville (Congo)] | | Year of publication |
| 1. Kasanga, T. K.; Mujinga, D. T.; Zeng, F. T.; Banza, M. I.; Mukakala, A. K.; Musapudi, E. M.; Mwamba, F. K.; Katambwa, P. M.; Nafatalewa, D. K.; Ngoie, C. N.; Cabala, V. P. K.; Kapessa, N. D.; Mbuyi-Musanzayi, S. | 2021 | | [Anorectal malformations: a 6-years review at the University Clinics of Lubumbashi] | | bookSection |
| 1. Kasanga, T. K.; Mujinga, D. T.; Zeng, F. T.; Banza, M. I.; Mukakala, A. K.; Musapudi, E. M.; Mwamba, F. K.; Katambwa, P. M.; Nafatalewa, D. K.; Ngoie, C. N.; Cabala, V. P. K.; Kapessa, N. D.; Mbuyi-Musanzayi, S. | 2021 | | [Anorectal malformations: a 6-years review at the University Clinics of Lubumbashi] | | Duplicate |
| 1. Doumbouya, N.; Brouh, Y.; Attié, R.; Keita, M.; Agbo-Panzo, D.; Diallo, A. F.; Barry, O. T.; Camara, A.; Baldé, I. | 2006 | | [Apparent malformations in an African area: factors influencing consultation delay in pediatric surgery] | | unrelated topic |
| 1. Koate, P.; N'Do, D.; Sankale, M. | 1974 | | [Arterial hypertension in Senegalese] | | Year of publication |
| 1. Goracci, G.; Marci, F.; Negri, P. L.; Treccani, A. | 1983 | | [Aspects of dental fluorosis in subjects from regions with water rich in fluorine and their classification] | | Year of publication |
| 1. Diatewa, B. M.; Maneh, N.; Domingo, A. S.; Mewamba Wamba, R.; Amouzou, D.; Didier Ayena, K.; Patrice Balo, K. | 2021 | | [Association between consanguinity with the ocular congenital abnormalities in Togo] | | unrelated topic |
| 1. Sengeyi, M. A.; Tandu-Umba, B. | 2006 | | [Association of neonatal polydactyly and maternal glucose tolerance impairment in the Congolese environment] | | unrelated topic |
| 1. Diouf, B.; Dia, D.; Ka, M. M.; Ka, E. F.; Diop, T. M. | 1998 | | [Autosomal dominant polycystosis in the hospital milieu in Dakar (Senegal)] | | Year of publication |
| 1. Barbotin, M.; Ducloux, M.; Perquis, P. | 1973 | | [Basedow's disease in Senegal (apropos of 25 clinical cases)] | | Year of publication |
| 1. Barbotin, M.; Ducloux, M.; Perquis, P. | 1975 | | [Basedow's disease in Senegal (apropos of 25 clinical cases)] | | Year of publication |
| 1. Pitché, P.; Agbèrè, A. D.; Gbadoé, A. J.; Tatagan, A.; Tchangaï-Walla, K. | 1998 | | [Bourneville's tuberous sclerosis and childhood epilepsy Apropos of 4 cases in Togo] | | Duplicate |
| 1. Pitché, P.; Agbèrè, A. D.; Gbadoé, A. J.; Tatagan, A.; Tchangaï-Walla, K. | 1998 | | [Bourneville's tuberous sclerosis and childhood epilepsy Apropos of 4 cases in Togo] | | Year of publication |
| 1. Ouedraogo, M.; Ouedraogo, S. M.; Zoubga, Z. A.; Badoum, G.; Ouedraogo, G.; Boncoungou, K.; Drabo, Y. J. | 2002 | | [Bronchopulmonary Kaposi's disease in 2 AIDS patients living in a zone of high tuberculosis/HIV prevalence] | | unrelated topic |
| 1. Mahé, A.; Flageul, B.; Bobin, P. | 1996 | | [Bullous IgA linear dermatosis of children in Mali] | | Year of publication |
| 1. Tougouma, S. J.; Kissou, S. A.; Yaméogo, A. A.; Yaméogo, N. V.; Bama, A.; Barro, M.; Héma, A.; Kaguembèga, L.; Nacro, B. | 2016 | | [Cardiopathies in children hospitalized at the University hospital Souro Sanou, Bobo-Dioulasso: echocardiographic and therapeutic aspects] | | unrelated topic |
| 1. Koaté, P.; Diouf, S.; Padonou, N.; Sylla, M. | 1977 | | [Cardiovascular abnormalities in black Africians (apropos of 326 cases in Senegalese)] | | Year of publication |
| 1. Diop, I. B.; Ba, S. A.; Sarr, M.; Kane, A.; Ly, M.; Diouf, S. M.; Ndiaye, M.; Fall, M.; Sow, D. | 1994 | | [Cardiovascular manifestations of Marfan's syndrome apropos of 6 cases] | | Year of publication |
| 1. Wandja, S. N.; Mussa, K.; Kamche, E. M.; Kalangu, K. | 2006 | | [Causes of neuropaediatric morbidity in Africa] | | unrelated topic |
| 1. Dumas, M.; Girard, P. L.; Borel, G. | 1976 | | [Cerebral vascular malformations in Senegal. II. Development, treatment and prognosis] | | Year of publication |
| 1. Borel, G.; Girard, P. L.; Dumas, M. | 1975 | | [Cerebrovascular malformations in Senegal (biographical, anatomical and clinical study)] | | Year of publication |
| 1. Morán, J. M.; Leal, A.; Espín, M. T.; Maciá Botejara, E.; Amaya, J. L.; Correa, M. I.; Saenz Santamaría, J. | 2011 | | [Changes in the fat composition and histomorphology of the liver in TPN, with and without ultra-short bowel] | | unrelated topic |
| 1. Ortega Valdes, G. | 1984 | | [Characteristics of occlusion of 241 Namibian students at the Isle of Youth, 1982] | | Year of publication |
| 1. Bénateau, H.; Traoré, H.; Chatellier, A.; Caillot, A.; Ambroise, B.; Veyssière, A. | 2015 | | [Child care management in maxillofacial humanitarian mission] | | unrelated topic |
| 1. Kpemissi, E.; Bakonde, B.; Agbere, A.; Boko, E.; Kessie, K.; Medji, L. A. | 1995 | | [Choanal atresia. An unrecognized malformation. Report of 3 cases at the Lomé University Hospital Center] | | Year of publication |
| 1. Sangwa, C. M.; Mukuku, O.; Tshisuz, C.; Panda, J. M.; Kakinga, M.; Kitembo, M. F.; Mutomb, J. F.; Odimba, B. F. | 2014 | | [Cleft lip palate in the Katanga Province of the Democratic Republic of Congo: epidemiological, clinicopathological and therapeutic aspects] | | unrelated topic |
| 1. Fatigba, O. H.; Mensah, E.; Salako, A. A.; Babio, R.; Mensa Savi De Tove, K.; Gandaho, P. | 2011 | | [Clinical and radio-anatomical aspects of traumatic brain injuries after road crash at one hospital, Benin] | | unrelated topic |
| 1. Cissé, A.; Cissé, A. F.; Touré, A.; Souaré, I. S.; Bah, H.; Kourouma, S.; Cissé, B.; Koulibaly, M.; Morel, Y.; Diaby, M. M.; Koné, S.; Ka, M. L.; Doukouré, M. | 2006 | | [Clinical and tomographic aspects of 29 cases of phakomatosis in Guinea] | | unrelated topic |
| 1. Norheim, A. J. | 2011 | | [Clubfoot in Uganda] | | unrelated topic |
| 1. Diop, A.; Thiam, O.; Guèye, M. L.; Seck, M.; Touré, A. O.; Cissé, M.; Dieng, M. | 2018 | | [Complicated Meckel diverticula: about 15 cases] | | Duplicate |
| 1. Diop, A.; Thiam, O.; Guèye, M. L.; Seck, M.; Touré, A. O.; Cissé, M.; Dieng, M. | 2018 | | [Complicated Meckel diverticula: about 15 cases] | | unrelated topic+B2572:B2583 |
| 1. Ouangré, E.; Zida, M.; Bazongo, M.; Sanou, A.; Bonkoungou, G. P.; Doamba, R. N.; Sawadogo, E. Y.; Ouédraogo, S.; Zongo, N.; Traore, S. S. | 2015 | | [Complications of Meckel diverticulum (MD) in adults: report of 11 cases CHU-Yalgado Ouedraogo in Burkina Faso] | | unrelated topic |
| 1. Thognon, P.; Fall, B.; Ouedraogo, T.; Toure, C. T.; Diop, A. | 1990 | | [Complications of Meckel's diverticulum in Dakar (apropos of 2 cases)] | | Year of publication |
| 1. Métras, D.; Turquin, H.; Coulibaly, A. O.; Ouattara, K. | 1979 | | [Congenital cardiopathies in a tropical environment. Study of 259 cases seen at Abidjan from 1969-1976] | | Year of publication |
| 1. Diop, I. B.; Ba, S. A.; Ba, K.; Sarr, M.; Kane, A.; Fall, M.; Guisse, A.; Sow, D.; Diouf, S. M. | 1995 | | [Congenital cardiopathies: anatomo-clinical, prognostic, and therapeutic features apropos of 103 cases seen at the Cardiology Clinic of the Dakar University Hospital Center] | | Year of publication |
| 1. Mbaye, A.; Bodian, M.; Ngaïdé, A. A.; Abdourafiq, H.; Leye, Mcbo; Savodogo, S.; Aw, F.; Ndiaye, M.; Kouamé, I.; Babaka, K.; Dioum, M.; Gaye, N. D.; Sarr, S. A.; Ndiaye, M. B.; Kane, A. D.; Kane, A. | 2017 | | [Congenital heart disease in adolescents and adults: Management in a general cardiology department in Senegal] | | unrelated topic |
| 1. Kinda, G.; Millogo, G. R.; Koueta, F.; Dao, L.; Talbousouma, S.; Cissé, H.; Djiguimdé, A.; Yé, D.; Sorgho, C. L. | 2015 | | [Congenital heart disease: epidemiological and echocardiography aspects about 109 cases in Pediatric Teaching Hospital Charles de Gaulle (CDG CHUP) in Ouagadougou, Burkina Faso] | | language |
| 1. Koate, P.; Padonou, N.; Sankale, M. | 1973 | | [Congenital heart diseases in the Senegalese (apropos of 151 patients in Dakar)] | | Year of publication |
| 1. Lubala, T. K.; Shongo, M. Y.; Munkana, A. N.; Mutombo, A. M.; Mbuyi, S. M.; wa Momat, F. K. | 2012 | | [Congenital malformations in Lubumbashi (Democratic Republic of Congo): about 72 cases observed and advocacy for the development of a National Registry of Congenital Malformations and a National Reference Center for Human Genetics] | | doesn’t include primary outcome |
| 1. Fall, M.; Diadhiou, F.; Kuakuvi, N.; Kessie, F.; Martin, S. L. | 1977 | | [Congenital malformations observed in the University Hospital Center of Dakar (neonatology unit)] | | Year of publication |
| 1. Kaimbo Wa Kaimbo, D.; Mwilambwe Wa Mwilambwe, A.; Kayembe, D. L.; Leys, A.; Missotten, L. | 1994 | | [Congenital malformations of the eyeball and its appendices in Zaire] | | Year of publication |
| 1. Diatewa, B. M.; Maneh, N.; Domingo, A. S.; Gnansa, K. E.; Ayikoue, Y. F. A.; Balo, K. P. | 2021 | | [Congenital ocular anomalies at the University Hospital Campus in Lomé, Togo] | | unrelated topic |
| 1. Atipo-Tsiba, P. W. | 2016 | | [Consanguineous marriage and morbi-mortality, short literature review based on an exceptional association: Usher syndrome and Von Recklinghausen neurofibromatosis] | | unrelated topic |
| 1. Ould Zein, H.; Ould Lebchir, D.; Ould Jiddou, M.; Ould Khalifa, I.; Bourlon, F.; Mechmeche, R. | 2006 | | [Consultation of congenital heart diseases in pediatric cardiology in Mauritania] | | unrelated topic |
| 1. Sy, H. S.; Diouf, S.; Diop, A.; Sarr, M. | 2004 | | [Contribution to the study of the congenital anomalies at the Albert-Royer paediatric hospital of Dakar] | | bookSection |
| 1. Dia, D.; Dieng, M. T.; Ndiaye, A. M.; Ndiaye, B.; Develoux, M. | 1999 | | [Crusted scabies in Dakar apropos of 11 cases seen in a year] | | Year of publication |
| 1. Laroche, R.; Barouti, H.; Sirol, J. | 1977 | | [Cutaneo-osseous Kaposi's disease] | | Year of publication |
| 1. Kaimbo, K.; Maertens, K. | 1986 | | [Dacryocystitis in children] | | Year of publication |
| 1. Dénakpo, J.; Lokossou, A.; Tonato-Bagnan, J. A.; Alao, J.; Hounkpatin, B.; Komongui, D. G.; Tamou-Tabé, D. A.; Perrin, R. X. | 2012 | | [Delivery in free position perhaps a solution to change delivery in traditional position in delivery rooms in Africa: results of a prospective study in Cotonou in Bénin] | | unrelated topic |
| 1. Faye, M.; Diawara, C. K.; Ndiaye, K. R.; Yam, A. A. | 2008 | | [Dental fluorosis and dental caries prevalence in Senegalese children living in a high-fluoride area and consuming a poor fluoridated drinking water] | | unrelated topic |
| 1. Tambo, F. F.; Nonga, B. N.; Andze, O. G.; Chiabi, A.; Minkande, J. Z.; Ngowe, M. N.; Gonsu, F. J.; Sosso, M. A. | 2010 | | [Difficulties in the management of esophageal atresia in developing countries] | | unrelated topic |
| 1. Perret, J. L.; Ngou-Milama, E.; Nguemby-Mbina, C. | 1995 | | [Distribution of glycosylated hemoglobin (HbA1) in an internal medicine service in Gabon] | | Year of publication |
| 1. Tandu-Umba, N. F.; Ntabona, B.; Mputu, L. | 1984 | | [Epidemiologic study of visible congenital malformations in Zaire] | | Year of publication |
| 1. Abellard, J.; Decroix, B.; Kerebel, L. M. | 1989 | | [Epidemiologic survey of orodental health in Fada N'Gourma (Burkina Faso)] | | Year of publication |
| 1. Fiogbé, M. A.; Gbénou, A. S.; Metchihoungbé, S.; Koura, A. | 2013 | | [Epidemiological and clinical aspects of visible urogenital malformations among adolescent's schoolboys at Cotonou] | | unrelated topic |
| 1. Rakotondramarina, D.; Razafimalala, F.; Andrianaivo, P.; Rabeson, D.; Andriatsiva, R.; Andrianavalomahefa, W. | 2000 | | [Epidemiological aspects of tuberculosis in middle west of Madagascar] | | unrelated topic |
| 1. Obame, M. N. A.; Mikolo, A. L.; Ndjota, B. N.; Comlan, E.; Abegue, M.; Ndong, F. O. | 2023 | | [Epidemiological, clinical and therapeutic profile of esophageal atresia at the Mother and Child University Hospital of the Jeanne Ebori Foundation from 2019 to 2020] | | bookSection |
| 1. Ndiaye, M. B.; Diao, M.; Pessinaba, S.; Bodian, M.; Kane, A. D.; Mbaye, A.; Dia, M. M.; Ciss, E. C.; Sarr, M.; Kane, A.; Ba, S. A. | 2011 | | [Epidemiological, clinical and ultrasonographic aspects of right-sided infective endocarditis in Senegal: 6 cases] | | unrelated topic |
| 1. Pio, M.; Afassinou, Y.; Pessinaba, S.; Baragou, S.; N'Djao, J.; Atta, B.; Ehlan, E.; Damorou, F.; Goeh-Akué, E. | 2014 | | [Epidemiology and etiology of heart failure in Lome] | | unrelated topic |
| 1. Nika, E. R.; Mabiala Babela, J. R.; Moyen, E.; Kambourou, J.; Oko, A. P.; Pemba Loufoua, A. B.; Mbika Cardorelle, A.; Moyen, G. | 2015 | | [Epidemiology and outcome of Congolese children who had surgery for heart defects] | | unrelated topic |
| 1. Tchen, J.; Ouledi, A.; Lepère, J. F.; Ferrandiz, D.; Yvin, J. L. | 2006 | | [Epidemiology and prevention of malaria in the southwestern islands of the Indian Ocean] | | unrelated topic |
| 1. Goracci, G.; Colangelo, G.; Luzi, V. | 1982 | | [Epidemiology of cleft lip and palate in Somalia] | | Year of publication |
| 1. Attamo, H.; Diawara, N. A.; Garba, A. | 2002 | | [Epidemiology of scorpion envenomations in the pediatric service of the Agadez hospital center (Niger) in 1999] | | unrelated topic |
| 1. Lengani, A.; Coulibaly, G.; Laville, M.; Zech, P. | 1997 | | [Epidemiology of severe chronic renal insufficiency in Burkina Faso] | | Year of publication |
| 1. Maiga, Y.; Napon, C.; Kuate Tegueu, C.; Traore, Y.; Tekete, I.; Mounkoro, N.; Dolo, A.; Maiga, M. Y.; Traore, H. A. | 2010 | | [Epilepsy and women's life: particularities of their management. Literature review] | | unrelated topic |
| 1. Renoirte, P.; Schoevaerdts, J. C.; Wibin, E. | 1971 | | [Esophageal atresia in Central Africa] | | Year of publication |
| 1. Ndiaye, I. P.; Ndiaye, M. M.; Mauferon, J. B.; Diagne, M.; Diop, A. G. | 1989 | | [Etiological aspects of polyneuritis in Senegal] | | Year of publication |
| 1. Sengeyi, M. A.; Tshibangu, K.; Tozin, R.; Nguma, M.; Tandu, U.; Sinamuli, K.; Mbanzulu, P. N.; Tshiani, K. | 1990 | | [Etiopathogenesis and type of congenital malformations observed in Kinshasa (Zaïre)] | | Year of publication |
| 1. Brousse, V.; Imbert, P.; Mbaye, P.; Kieffer, F.; Thiam, M.; Ka, A. S.; Gerardin, P.; Sidi, D. | 2003 | | [Evaluation of long-term outcome of Senegalese children sent abroad for cardiac surgery] | | unrelated topic |
| 1. Ngom, G.; Fall, I.; Sankale, A. A.; Konate, I.; Dieng, M.; Sanou, A.; Ndiaye, L.; Ndoye, M. | 2004 | | [Evaluation of the management of omphalocele at Dakar] | | unrelated topic |
| 1. Ban, T. | 1972 | | [Experimental autonomic disorders] | | Year of publication |
| 1. Salvi, P. F.; Balducci, G.; Dente, M.; Sarti, P.; Bocchetti, T.; Farah, P.; Ziparo, V. | 2012 | | [Experimental teaching program on cooperation between "Sapienza" University of Rome and the University Hospital "Le Bon Samaritain" in N'Djamena, Chad] | | unrelated topic |
| 1. Ouattara, K.; Daffe, S.; Timbely, A. | 1992 | | [Exstrophy of the bladder in adults] | | Year of publication |
| 1. Schoevaerdts, J. C.; Diomi, P. | 1974 | | [Fifty seven cases of aneurysms observed in the Democratic Republic of Zaïre (author's transl)] | | Year of publication |
| 1. Brouwer, I. D.; de Bruin, A.; Hautvast, J. G.; Backer Dirks, O. | 1989 | | [Fluorosis in Senegal] | | Year of publication |
| 1. Kabre, A.; Badiane, S. B.; Sakho, Y.; Ba, M. C.; Et Gueye, M. | 1994 | | [Genetic and etiologic aspects od spina bifida in Senegal. Apropos of 211 cases collected at the neurosurgical clinic UHC of Fann] | | Year of publication |
| 1. Traoré, B.; Cissé, L. | 2018 | | [Giant verrucous nevus in a 15-year old girl: about a case] | | unrelated topic |
| 1. Campbell, C.; Louw, B. | 1992 | | [Guidance to parent of black babies with a cleft lip and palate] | | Year of publication |
| 1. Sow, N. F.; Lèye, M.; Basse, I.; Dieng, Y. J.; Seck, M. A.; Cissé, D. F.; Sow, A.; Fattah, M.; Kane, A.; Houngbadji, M.; Faye, P. M.; Fall, A. L.; Guèye, N. R. D.; Ndiaye, O. | 2018 | | [Heart failure with normal heart revealing complex arteriovenous malformation of the lower limb in a child: case study and literature review] | | unrelated topic |
| 1. Naseman, T. | 1980 | | [Herxheimer's chronic atrophic acrodermatitis acquired in South Africa] | | Year of publication |
| 1. Nasemann, T. | 1980 | | [Herxheimer's chronic atrophic acrodermatitis acquired in South Africa] | | Year of publication |
| 1. Acea Nebril, B.; Bouso Montero, M.; Blanco Freire, N.; Taboada Filgueira, L.; Freire Rodríguez, D.; Arnal Monreal, F.; Gómez Freijoso, C. | 1996 | | [Heterotopic gastric mucosa in the ileum with perforated ulcer] | | Year of publication |
| 1. Vignikin-Yehouessi, B.; Gomina, M.; Adjibabi, W.; Biotchane, I.; Vodouhe, S. J.; Hounkpe, Y. Y.; Medji, A. L. | 2006 | | [HIV and ENT manifestations: epidemiologic and clinic aspects at CNHU of Cotonou and CHD Oueme-Plateau] | | unrelated topic |
| 1. Badiane, M.; Ba, M.; Camara, B.; Tortey, E.; Badiane, S. B.; Sall, M. G.; Lamouche, P. | 1989 | | [Hydranencephaly: report of a case] | | Year of publication |
| 1. Salem-Memou, S.; Chavey, S.; Elmoustapha, H.; Mamoune, A.; Moctar, A.; Salihy, S.; Boukhrissi, N. | 2020 | | [Hydrocephalus in newborns and infants at the Nouakchott National Hospital] | | bookSection |
| 1. Salem-Memou, S.; Chavey, S.; Elmoustapha, H.; Mamoune, A.; Moctar, A.; Salihy, S.; Boukhrissi, N. | 2020 | | [Hydrocephalus in newborns and infants at the Nouakchott National Hospital] | | Duplicate |
| 1. Korsaga/Somé, N.; Salissou, L.; Tapsoba, G. P.; Ouédraogo, M. S.; Traoré, F.; Doulla, M.; Barro/Traoré, F.; Niamba, P.; Traoré, A. | 2016 | | [Ichthyosis and social stigma in Burkina Faso] | | unrelated topic |
| 1. Kouadio-Yapo, C. G.; Dou, G. S. P.; Aka, N. A. D.; Zika, K. D.; Adoubryn, K. D.; Dosso, M. | 2018 | | [Identification of yeasts from commercial attiéké in Abidjan (Côte d'Ivoire): Preliminary study] | | Duplicate |
| 1. Kouadio-Yapo, C. G.; Dou, G. S. P.; Aka, N. A. D.; Zika, K. D.; Adoubryn, K. D.; Dosso, M. | 2018 | | [Identification of yeasts from commercial attiéké in Abidjan (Côte d'Ivoire): Preliminary study] | | unrelated topic |
| 1. van Wyk, P. J.; Bütow, K. W.; van der Merwe, C. A.; Kleynhans, E. E. | 1987 | | [Incidence and clinical appearance of cleft deformities in the Transvaal] | | Year of publication |
| 1. Yameogo, N. V.; Kologo, K. J.; Yameogo, A. A.; Yonaba, C.; Millogo, G. R.; Kissou, S. A.; Toguyeni, B. J.; Samadoulougou, A. K.; Pignatelli, S.; Simpore, J.; Zabsonre, P. | 2014 | | [Infective endocarditis in sub-Saharan african children, cross-sectional study about 19 cases in Ouagadougou at Burkina Faso] | | unrelated topic |
| 1. Basse, I.; Fall, A. L.; Seck, N.; Boiro, D.; Ba, A.; Diawara, N. N.; Niang, F.; Diop, D. C. O.; Ndongo, A. A.; Thiam, L.; Diagne/Guéye, N. R.; Ndiaye, M. | 2018 | | [Interauricular communications in children: diagnosis and treatment, about 49 cases at the Children?s University Hospital in Dakar] | | unrelated topic |
| 1. Randriamizao, H. M. R.; Rakotondrainibe, A.; Rahanitriniaina, N. M. P.; Rajaonera, A. T.; Andriamanarivo, M. L. | 2017 | | [Intraoperative management of esophageal atresia: small steps that cannot be ignored in Madagascar] | | unrelated topic |
| 1. Diakité, M. L.; Berthé, H. Jg; Timbely, A.; Diallo, M.; Maiga, M.; Diakité, A.; Ouattara, K.; Faure, A. | 2013 | | [Issues inherent to the management of disorders of sex development in Point G Hospital] | | unrelated topic |
| 1. Doumbo, O.; Soula, G.; Kodio, B.; Perrenoud, M. | 1992 | | [Ivermectin and pregnancy in mass treatment in Mali] | | Year of publication |
| 1. Wechsler, J.; Delcourt, A.; Raphael, M.; Frances-Michel, C.; Demay, G.; Pinaudeau, Y.; Chomette, G. | 1983 | | [Kaposi's disease. Form with lymph node manifestation in a young African. Difficulty in the initial diagnosis and relation to dysimmune lymphadenitis] | | Year of publication |
| 1. Noukpozounkou, S. B.; Lawani, I.; Elegbede, O. T. A.; Seto, D. M.; Assan, B. R.; Houegban, Ascr; Koco, H.; Fiogbe, M. A. | 2018 | | [Littré's strangled umbilical hernia in children: a rare complication due to a common malformation of the small bowel] | | unrelated topic |
| 1. Kayembe, B.; Ntumba, M. K. | 1987 | | [Local retention of various deciduous teeth in adolescent and adult subjects in Zaire] | | Duplicate |
| 1. Kayembe, B.; Ntumba, M. K. | 1987 | | [Local retention of various deciduous teeth in adolescent and adult subjects in Zaire] | | Year of publication |
| 1. Sakho, Y.; Badiane, S. B.; Kabre, A.; Ba, S.; Ba, M. C.; Gueye, E. M.; Diene, S.; Gueye, M. | 1998 | | [Lumbosacral intraspinal lipomas associated or not with a tethered cord syndrome (series of 8 cases)] | | Year of publication |
| 1. Bacon, W. | 1977 | | [Malocclusions in young Senegalese of school age] | | Year of publication |
| 1. Amadou, I.; Coulibaly, Y. M.; Coulibaly, O. M.; Konaté, D.; Coulibaly, Y.; Coulibaly, M. T.; Maiga, B.; Doumbia, A.; Traoré, F.; Karembé, B.; Djire, M. K.; Kamate, B.; Daou, M. B.; Barry, A.; Cissé, M. E.; Coulibaly, O.; Dembélé, A. | 2023 | | [Management Of Blader Exstrophy In Children At The CHU Gabriel Touré] | | unrelated topic |
| 1. Rasamoelisoa, J.; Raobijaona, H.; Tovone, X. G.; Rajaonarivelo, A.; Rakotoarimanana, D. R. | 2000 | | [Management of childhood heart disease in Madagascar. What perspective for tomorrow?] | | unrelated topic |
| 1. Sankale, A. A.; Ndiaye, A.; Baillet, A.; Ndiaye, L.; Ndoye, M. | 2012 | | [Management of nasolabial clefts: the issue in Dakar] | | unrelated topic |
| 1. Kouéta, F.; Ouédraogo Yugbaré, S. O.; Dao, L.; Dao, F.; Yé, D.; Kam, K. L. | 2011 | | [Medical audit of neonatal deaths with the "three delay" model in a pediatric hospital in Ouagadougou] | | unrelated topic |
| 1. Cardorelle, A. M.; Okoko, A. R.; Perez, A. C.; Moyen, G. | 2004 | | [Medical transportation of Congolese children by the Foundation "Terre des hommes" Netherlands (1989–1998)] | | unrelated topic |
| 1. Beogo, R.; Andonaba, J. B.; Bouletreau, P.; Traore Sawadogo, H.; Traore, A. | 2012 | | [Multiple facial squamous cell carcinomas in a child, revealing a xeroderma pigmentosum] | | unrelated topic |
| 1. Ouaïmon, D. S.; Ndoma Ngatchoukpo, V.; Sopio, J. I. J.; Borohoul, A. R. | 2023 | | [Neglected cleft palate treatment at the Pediatric university hospital of Bangui (Central African Republic)] | | unrelated topic |
| 1. Souna, B. S.; Ganda, S.; Aboubacar, C. L.; Assoumane, I. | 2009 | | [Neglected idiopathic congenital clubfoot : surgical treatment before 20 years old. Retrospective study of 40 feet] | | unrelated topic |
| 1. Fatigba, O. H.; Savi de Tove, K. M.; Allode, S. A.; Babio, R.; Padonou, J. | 2011 | | [Neurosurgical management of head injury in Parakou, Benin: use of radiography] | | unrelated topic |
| 1. Takou Tsapmene, V.; Bilong, Y.; Mah Mungyeh, E.; Assumpta Bella, L. | 2022 | | [Ocular abnormalities of children born prematurely at the Yaoundé Gynaeco-Obstetrics And Pediatric Hospital] | | unrelated topic |
| 1. Kerebel, B.; Gaillard, A.; Loubet, P. | 1974 | | [Odontologic study of a case of historic rachitis] | | Year of publication |
| 1. Kerebel, B.; Gaillard, A.; Loubet, P. | 1975 | | [Odontologic study of a case of historic rachitis] | | Year of publication |
| 1. Bandré, E.; Niandolo, K. A.; Wandaogo, A.; Bankolé, R.; Mobiot, M. L. | 2010 | | [Oesophageal atresia: management in Sub Saharian countries] | | bookSection |
| 1. Kanté, L.; Togo, A.; Diakité, I.; Maiga, A.; Traoré, A.; Samaké, A.; Samaké, H.; Dembélé, B. T.; Keita, M.; Coulibaly, Y.; Diallo, G. | 2010 | | [Omphalocele in general and pediatric surgery in Gabriel Touré] | | unrelated topic |
| 1. Seye, C.; Mbaye, P. A.; Ndoye, N. A.; Diouf, C.; Fall, M.; Sagna, A.; Ndour, O.; Ngom, G. | 2020 | | [Omphalomesenteric fistulas; epidemiological diagnostic and therapeutic features: study of four cases in the Department of Pediatric Surgery at the Aristide Le Dantec University Hospital in Dakar] | | bookSection |
| 1. Metras, D.; Ouezzin-Coulibaly, A.; Ouattara, K.; Chauvet, J.; Longechaud, A.; Millet, P. | 1983 | | [Open-heart surgery in tropical Africa. Results and peculiar problems of the 1st 300 cases of extracorporeal circulation performed in Abidjan] | | Year of publication |
| 1. Amadou, I.; Coulibaly, Y.; Coulibaly, M. T.; Coulibaly, M. O.; Traoré, B.; Keita, M.; Traoré, F.; Sogoba, Y.; Koné, A.; Djiré, M. K.; Kamaté, B.; Doumbia, A.; Diall, H.; Coulibaly, O.; Maiga, B.; Ali Ada, M. O.; Konaté, M.; Diakité, I.; Maiga, M.; Ouologem, H.; Diallo, G. | 2018 | | [Pathologies of peritoneo-vaginal canal in pediatric surgery at the teaching hospital Gabriel Touré] | | unrelated topic |
| 1. Metras, D.; Coulibaly, A. Q.; Ouattara, K. | 1979 | | [Personal experience of cardiac surgery at Abidjan. Initial results (author's transl)] | | Year of publication |
| 1. Marchetti, G.; Wagener, H. H. | 1974 | | [Pharmacological and toxicological characterization of a multicomponent preparation in the treatment of circulatory disorders] | | Year of publication |
| 1. Chamorro, G.; Salazar, M.; Salazar, S.; Mendoza, T. | 1993 | | [Pharmacology and toxicology of Guatteria gaumeri and alpha-asarone] | | Year of publication |
| 1. Bartsch, R.; Nowak, R.; Femmer, K.; Stade, K. | 1970 | | [Pharmacology of the new antihistaminic 9,9-dioxopromethazine (Prothanon)] | | Year of publication |
| 1. Saboye, J. | 1999 | | [Plastic surgery training missions in developing countries. A 10-year experience at missions in Mali] | | Year of publication |
| 1. Golubeva, M. I.; Shashkina, L. F.; Proĭnova, V. A.; Fedorova, E. A.; Nechushkina, L. V. | 1985 | | [Preclinical study of the safety of the antihistaminic preparation dimebon] | | Year of publication |
| 1. Diouf, M.; Cisse, D.; Lo, C. M.; Ly, M.; Faye, D.; Ndiaye, O. | 2012 | | [Pregnant women living in areas of endemic fluorosis in Senegal and low birthweight newborns: case-control study] | | unrelated topic |
| 1. Chabal, J.; Goudote, E. | 1967 | | [Present-day problems of pediatric surgery in Senegal (apropos of 1000 cases)] | | Year of publication |
| 1. Bodian, M.; Ngaïdé, A. A.; Mbaye, A.; Sarr, S. A.; Jobe, M.; Ndiaye, M. B.; Kane, A. D.; Aw, F.; Gaye, N. D.; Ba, F. G.; Bah, M. B.; Tabane, A.; Dioum, M.; Diagne, D.; Diao, M.; Diack, B.; Sarr, M.; Kane, A.; Bâ, S. A. | 2015 | | [Prevalence of congenital heart diseases in Koranic schools (daara) in Dakar: a cross-sectional study based on clinical and echocardiographic screening in 2019 school children] | | study population |
| 1. Diagne, F.; Diop-Ba, K.; Yam, A. A.; Diop, F. | 2001 | | [Prevalence of dental agenesis: a radiologic and clinical study in Dakar] | | unrelated topic |
| 1. Alquier, R. P.; Pochan, Y. | 1991 | | [Primary cheiloplasty: ambulatory surgical technique in African countries] | | Year of publication |
| 1. Huber, A.; Boldt, H. W. | 1968 | | [Problems of obstetrics and gynecology in an African developing country (Ethiopia)] | | Year of publication |
| 1. Ikama, M. S.; Nkalla-Lambi, M.; Kimbally-Kaky, G.; Loumouamou, M. L.; Nkoua, J. L. | 2013 | | [Profile of infective endocarditis at Brazzaville University Hospital] | | unrelated topic |
| 1. Diao, B.; Diallo, Y.; Fall, P. A.; Ngom, G.; Fall, B.; Ndoye, A. K.; Fall, I.; Ba, M.; Ndoye, M.; Diagne, B. A. | 2008 | | [Prune Belly syndrome: epidemiologic, clinic and therapeutic aspects] | | unrelated topic |
| 1. Diagne, J. P.; Sow, A. S.; Ka, A. M.; Wane, A. M.; Ndoye Roth, P. A.; Ba, E. A.; De Medeiros, M. E.; Ndiaye, J. M.; Diallo, H. M.; Kane, H.; Sow, S.; Nguer, M.; Sy, E. M.; Ndiaye, P. A. | 2017 | | [Rare causes of childhood leukocoria] | | unrelated topic |
| 1. Awa, H. D. M.; Mvondo, R. M. N.; Nguefack, S.; Messanga, C. B.; Ndombo, P. O. K. | 2019 | | [Rare diseases and their clinical oral manifestations in two Hospitals in Yaoundé] | | unrelated topic |
| 1. Ek, M.; Vladic-Stjernholm, Y.; Günther, A.; Hällsjö-Sander, C.; Jacobsen, P. H. | 2016 | | [Rheumatic heart disease behind life-threatening heart failure in pregnancy] | | unrelated topic |
| 1. Haugstvedt, S. | 1990 | | [Separation of Siamese twins. Neither child was given preference] | | Year of publication |
| 1. Vague, J.; Favier, G. | 1977 | | [Sex hormones and homosexuality] | | Year of publication |
| 1. Mahé, A.; Bobin, P.; Coulibaly, S.; Tounkara, A. | 1997 | | [Skin diseases disclosing human immunodeficiency virus infection in Mali] | | Duplicate |
| 1. Mahé, A.; Bobin, P.; Coulibaly, S.; Tounkara, A. | 1997 | | [Skin diseases disclosing human immunodeficiency virus infection in Mali] | | Year of publication |
| 1. Monsia, A.; Fiogbé, M. A.; Dieth, A. G.; Zokou, G. H.; Aguéhoundé, C. | 2008 | | [Surgery of congenital clubfoot in Don Orione, Health Center for physical handicaps of Ivory Coast (About 554 feet)] | | unrelated topic |
| 1. Moreau, J. L.; Schvartz, A.; Saffieddine, G.; Baume, L. J. | 1985 | | [Taurodontism studies in Senegal] | | Year of publication |
| 1. Niang, L.; Diao, B.; Gueye, S. M.; Fall, P. A.; Moby-Mpah, H.; Jalloh, M.; Ndoye, A. K.; Diagne, B. A. | 2007 | | [Testicular cancer is a rare condition in black people. We report 5 cases of testicular cancer in men with undescended testis] | | unrelated topic |
| 1. Diop, I. B.; Ba, S. A.; Sarr, M.; Kane, A.; Hane, L.; Dieye, O.; Ndiaye, M.; Ba, K.; Sow, D.; Fall, M.; Diouf, S. M. | 1997 | | [Tetralogy of Fallot. Anatomo-clinical, prognostic and therapeutic features] | | Year of publication |
| 1. Draehmpaehl, D. | 1994 | | [The behavior of sperm antibody titers after immunization of guinea pigs with their own sperm and after experimental unilateral cryptorchism] | | Year of publication |
| 1. Sviderskaia, G. E.; Dmitrieva, L. E. | 1991 | | [The effect of long-term hyperbarism on pregnant guinea pigs and their progeny] | | Duplicate |
| 1. Sviderskaia, G. E.; Dmitrieva, L. E. | 1991 | | [The effect of long-term hyperbarism on pregnant guinea pigs and their progeny] | | Year of publication |
| 1. Longombe, A. O.; Tshimbila Kabangu, J. M. | 2012 | | [The epidemiological approach of clefts lip and palate in the eastern of Democratic Republic of Congo] | | unrelated topic |
| 1. Maksimović, J.; Maksimović, M. | 2016 | | [The first record of the delivery of conjoined twins in the Srem county in the 19th century] | | unrelated topic |
| 1. Ba, M. C.; Ly Ba, A.; Hossini, A.; Diallo, O.; Thiam, A. B.; Ndoye, N.; Sakho, Y.; Badiane, S. B. | 2007 | | [The occipital encephaloceles: report of 16 cases] | | unrelated topic |
| 1. Fall, P. A.; Gueye, S. M.; Ndoye, A.; Sylla, C.; Abdallahi, M. O.; Diame, A. A.; Ba, M.; Diagne, B. A. | 2000 | | [The pathology of the peritoneo-vaginal process in the young males: clinical and therapeutical aspects in 160 cases] | | unrelated topic |
| 1. Ngouala, G. A.; Affangla, D. A.; Leye, M.; Kane, A. | 2015 | | [The prevalence of symptomatic infantile heart disease at Louga Regional Hospital, Senegal] | | unrelated topic |
| 1. Oulai, S.; Cissé, L.; Enoh, J.; Yao, A.; Maho, S.; Andoh, J. | 2008 | | [The psychological experience of the mothers whose babies were born deformed in the neonatological ward of the academic hospital center of Treichville (Côte-d'Ivoire)] | | unrelated topic |
| 1. Lim, C. K. | 2018 | | [The Recent Epidemic Spread of Zika Virus Disease] | | unrelated topic |
| 1. Aymé, S.; Julian, C.; Maurin, N.; Gambarelli, D.; Sudan, N.; Giraud, F. | 1985 | | [The register of stillbirths in Bouches-du-Rhône: evaluation after 3 years in operation] | | Year of publication |
| 1. Hohlweg, W. | 1971 | | [The significance of sex hormones secreted by fetal gonads for the development of the sex-specific genital apparatus and for the determination of heterosexual or homosexual behavior] | | Year of publication |
| 1. Konaté, I.; Diao, M. L.; Cissé, M.; Dieng, M.; Ka, O.; Touré, C. T. | 2010 | | [The surgical treatment results of ulcerous pyloro-duodenal stenosis: about 160 cases] | | unrelated topic |
| 1. Tanaka, K.; Utsunomiya, T. | 1982 | | [The toxicity of N,N-dimethylformamide (DMF)] | | Year of publication |
| 1. Mvogo, C. E.; Ellong, A.; Ndjock, R.; Bella, A. L.; Luma, H. | 2006 | | [Torticollis in a group of strabismic patients in Cameroon] | | unrelated topic |
| 1. Liubimov, B. I.; Mitrofanov, V. S.; Porfir'eva, R. P.; Smol'nikova, N. M.; Strekalov, S. N. | 1976 | | [Toxicity of etmozine, a new antiarrhythmic agent] | | Year of publication |
| 1. Kakou Guikahué, M.; Chauvet, J.; Seka, R.; Yapobi, Y.; Ake, E.; Kroo Aka, F.; Ekra, A.; Kachaner, J.; Bertrand, E. | 1990 | | [Transposition of the great vessels in western Africa. Apropos of 17 cases observed at the Institute of Cardiology in Abidjan] | | Year of publication |
| 1. Bourinet, V.; Thiam, K.; Guinde, J.; Laroumagne, S.; Dutau, H.; Astoul, P. | 2019 | | [Trans-vocal cord prostheses - preliminary experience treating benign laryngotracheal stenosis in adults] | | unrelated topic |
| 1. Diop Ba, K.; Diagne, F.; Ngom, P. I.; Badiane, A.; Diop, F.; Yam, A. A. | 2003 | | [Treating five cases of Class III skeletal brachygnatia maxilla with the Delaire mask] | | unrelated topic |
| 1. Théra, J. P.; Tiama, J. M.; Konipo, A.; Napo, A.; Bamani, S. | 2020 | | [Treatment of congenital ptosis in a low-income country: polypropylene frontalis sling at the African Institute of Tropical Ophthalmology] | | unrelated topic |
| 1. Clausing, P.; Buschmann, J. | 1984 | | [Trends in experimental teratology: behavioral teratology] | | Year of publication |
| 1. Sobrinho, L. G. | 1968 | | [True hermaphroditism in Africans: report of 2 cases] | | Year of publication |
| 1. Hadjadj, S.; Talarmin, F.; Andreu, J. P.; M'Baye, P. S. | 1996 | | [True hermaphroditism in Senegal: cultural and religious influences on sexual orientation] | | Year of publication |
| 1. Chalabi-Benabdallah, A.; Mohammed-Brahim, A.; Benlaldj, M. | 1989 | | [Tuberous sclerosis in children in western Algeria] | | Year of publication |
| 1. Sow, D. S.; Bah, M.; Traoré, D.; Dante, M. L.; Mariko, M.; Traoré, B.; N'Diaye, H. D.; Doumbia, N.; Sidibé, A. T. | 2018 | | [Turner syndrome in the hôpital du Mali, a case] | | unrelated topic |
| 1. Mbaye, M.; Sylla, N.; Thioub, M.; Sy, E. C. N.; Faye, M.; Thiam, A. B.; Ba, M. C.; Badiane, S. B. | 2019 | | [Update on intrasellar arachnoid cyst: a case study] | | bookSection |
| 1. Akpo, C.; Hodonou, R.; Njanteng Nounjio, R.; Hounnasso, P.; Goudote, E. | 1998 | | [Urethral diseases in children at surgical departments of the Cotonou Hospital. Apropos of 77 cases] | | Year of publication |
| 1. Coulibaly, B.; Dick, B.; Bankole, R.; Demoulet, C.; Gmagne, Y. M.; Moussa, B.; Vodi, L.; Mobiot, L. | 1994 | | [Valves of the posterior urethra in newborns, infants and children. Apropos of a series of 60 cases] | | Year of publication |
| 1. Ducka-Karska, K. | 1983 | | [Various dental anomalies in the black population of Lubumbashi (Zaire)] | | Year of publication |
| 1. Gasparová, J.; Jäger, V.; Andrik, P. | 1979 | | [Various stomatological findings in native Zambian children] | | Year of publication |
| 1. Akakpo, A. S.; Saka, B.; Téclessou, J. N.; Djalogue, L.; Mahamadou, G.; Mouhari-Touré, A.; Gottara, W. S.; Kombaté, K.; Tchangai-Walla, K.; Pitché, P. | 2018 | | [Vascular Cutaneous Abnormalities in Togo: a 120-Case Study] | | unrelated topic |
| 1. Diarra, O.; Ba, M.; Ndiaye, A.; Ciss, G.; Dia, A.; Ndiaye, M. | 2003 | | [Vascular dysplasia in vascular surgery in an African area: 28 cases at the Dakar teaching hospital] | | unrelated topic |
| 1. Mori, Y.; Otsuki, N.; Sakata, M.; Okamoto, K. | 2011 | | [Virology of the family Togaviridae] | | unrelated topic |
| 1. Micheau, P.; Lauwers, F. | 1999 | | [What are the objectives of a humanitarian reconstructive plastic surgery mission?] | | Year of publication |
| 1. Hébert, J. C.; Lefait, J. F.; Hébert, O. | 1994 | | [Xeroderma pigmentosum in negroid children. 5 cases in Mahori children] | | Year of publication |
| 1. Ndiaye, B.; Ball, M. D.; Strobel, M.; Niang, I. | 1983 | | [Xeroderma pigmentosum: 1st Senegalese case] | | Year of publication |
| 1. Dieng, M. T.; Niang, S. O.; Dangou, J. M.; Ndiaye, B. | 2001 | | [Xeroderma pigmentosum: report of 6 cases in Dakar] | | unrelated topic |
| 1. Gassama, M.; Tall, K.; Karabinta, Y.; Koné, M. B.; Traoré, B.; Keita, L.; Fofana, Y.; Sissoko, M.; Dicko, A.; Sylla, O.; Guindo, B.; Diakité, B.; Faye, O. | 2019 | | [Xerodermapigmentosum: Challenge of diagnosis in West Africa] | | unrelated topic |
| 1. A Makubi, C Hage, J Lwakatare, P Kisenge, J Makani… | 2014 | | … aetiology, clinical characteristics and prognosis of adults with heart failure observed in a tertiary hospital in Tanzania: the prospective Tanzania Heart Failure (TaHeF) … | | unrelated topic |
| 1. Shah, MJ; Silka, MJ; Silva, JNA; Balaji, S; ... | 2021 | | … Congenital Cardiology (AEPC). Endorsed by the Asia Pacific Heart Rhythm Society (APHRS), the Indian Heart Rhythm Society (IHRS), and the Latin American Heart … | | unrelated topic |
| 1. Mariki, HK | 2017 | | … profiles associated with congenital heart disease among children aged 0-59 months at Muhimbili national hospital and Jakaya Kikwete Cardiac Institute … | | book |
| 1. Oyania, F.; Kotagal, M.; Situma, M. | 2021 | | 15-Year-old with neglected recto-vestibular fistula in western Uganda: a case report | | bookSection |
| 1. Kruszka, P.; Addissie, Y. A.; McGinn, D. E.; Porras, A. R.; Biggs, E.; Share, M.; Crowley, T. B.; Chung, B. H.; Mok, G. T.; Mak, C. C.; Muthukumarasamy, P.; Thong, M. K.; Sirisena, N. D.; Dissanayake, V. H.; Paththinige, C. S.; Prabodha, L. B.; Mishra, R.; Shotelersuk, V.; Ekure, E. N.; Sokunbi, O. J.; Kalu, N.; Ferreira, C. R.; Duncan, J. M.; Patil, S. J.; Jones, K. L.; Kaplan, J. D.; Abdul-Rahman, O. A.; Uwineza, A.; Mutesa, L.; Moresco, A.; Obregon, M. G.; Richieri-Costa, A.; Gil-da-Silva-Lopes, V. L.; Adeyemo, A. A.; Summar, M.; Zackai, E. H.; McDonald-McGinn, D. M.; Linguraru, M. G.; Muenke, M. | 2017 | | 22q11.2 deletion syndrome in diverse populations | | unrelated topic |
| 1. Grand, C |  | | 23 to 26 February 2020 Cinnamon Grand Hotel, Colombo, Sri Lanka | | book |
| 1. Tour, PG | 2018 | | 27. European Stroke Conference, Athens, Greece 2018 Scientific Programme Overview-Friday 13 April 2018 | | unrelated topic |
| 1. Tsuzuki, T; Kato, M | 2019 | | 31 st European Congress of Pathology | | book |
| 1. Gripp, KW; Jones, KL; Wenger, TL; ... | 2021 | | 41st Annual David W. Smith workshop on malformations and morphogenesis: Abstracts of the 2020 annual meeting | | unrelated topic |
| 1. Ellaithi, M.; Werner, R.; Riepe, F. G.; Krone, N.; Kulle, A. E.; Diab, T.; Kamel, A. K.; Arlt, W.; Holterhus, P. M.; Sabir, O.; Hiort, O. | 2014 | | 46,XY disorder of sex development in a sudanese patient caused by a novel mutation in the HSD17B3 gene | | unrelated topic |
| 1. Beaton, A; Lacey, S; Mwambu, T; ... | 2012 | | 72 Pediatric Cardiology in the Tropics and Underdeveloped Countries | | unrelated topic |
| 1. Ellmann, A.; van Heerden, P. D.; van Heerden, B. B.; Klopper, J. F. | 2001 | | 99mTc-MIBI stress-rest myocardial perfusion scintigraphy in patients with complete left bundle branch block | | unrelated topic |
| 1. Sacarlal, J.; Nhacolo, A. Q.; Sigaúque, B.; Nhalungo, D. A.; Abacassamo, F.; Sacoor, C. N.; Aide, P.; Machevo, S.; Nhampossa, T.; Macete, E. V.; Bassat, Q.; David, C.; Bardají, A.; Letang, E.; Saúte, F.; Aponte, J. J.; Thompson, R.; Alonso, P. L. | 2009 | | A 10 year study of the cause of death in children under 15 years in Manhiça, Mozambique | | unrelated topic |
| 1. Ekpebegh, C. O.; Coetzee, E. J.; van der Merwe, L.; Levitt, N. S. | 2007 | | A 10-year retrospective analysis of pregnancy outcome in pregestational Type 2 diabetes: comparison of insulin and oral glucose-lowering agents | | unrelated topic |
| 1. Belay, K. E.; Ayalew, B. L.; Amogne, M. T.; Alemneh, T. A.; Geletew, T. K. | 2023 | | A 14-year-old male patient with diagnosis of Prader-Willi syndrome in Ethiopia: a case report | | unrelated topic |
| 1. Klopper, G. J.; Adeniyi, O. V. | 2023 | | A 20-year audit of the outcomes of stenting with the Montgomery Safe-T-Tube at a tertiary hospital in South Africa | | unrelated topic |
| 1. Kana, M. A.; Baduku, T. S.; Bello-Manga, H.; Baduku, A. S. | 2018 | | A 37-year-old Nigerian woman with Apert syndrome - medical and psychosocial perspectives: a case report | | Duplicate |
| 1. Kana, M. A.; Baduku, T. S.; Bello-Manga, H.; Baduku, A. S. | 2018 | | A 37-year-old Nigerian woman with Apert syndrome - medical and psychosocial perspectives: a case report | | unrelated topic |
| 1. Retief, A. E.; Bernstein, R.; Grace, H. J.; Nelson, M. M.; Jansen, S.; Benjamin, M.; Bester, R. | 1983 | | A 3-year cytogenetic survey of 9 661 patients in South Africa | | Year of publication |
| 1. Bodensteiner, J. B. | 2014 | | A 4-year-old Nigerian boy with cerebral palsy? | | unrelated topic |
| 1. Schoonraad, L.; Slogrove, A.; Engelbrecht, A.; Urban, M. F. | 2020 | | A 5-Year Retrospective Review of the Health Supervision Received by Children with Down Syndrome at a South African Regional Hospital | | unrelated topic |
| 1. Kinaston, R. L.; Roberts, G. L.; Buckley, H. R.; Oxenham, M. | 2016 | | A bioarchaeological analysis of oral and physiological health on the south coast of New Guinea | | unrelated topic |
| 1. Muturi, A.; Kotecha, V.; Ojee, C.; Mang'oka, D.; Muthuri, J. | 2016 | | A caecal pseoudotumour with an incidental adenomatoid testicular tumour in a man with right undescended testis: a case report | | unrelated topic |
| 1. Wonkam, A.; Hurst, S. | 2014 | | A call for policy action in sub-Saharan Africa to rethink diagnostics for pregnancy affected by sickle cell disease: differential views of medical doctors, parents and adult patients predict value conflicts in Cameroon | | unrelated topic |
| 1. Aliku, T. O.; Lubega, S.; Lwabi, P. | 2014 | | A case of anomalous origin of the left coronary artery presenting with acute myocardial infarction and cardiovascular collapse | | unrelated topic |
| 1. Samuel, M. O.; Adamu, S. S.; Ogiji, E. E.; Bello, D. F.; Allagh, J. S.; Ate, I. U.; Rabol, J. S. | 2015 | | A case of congenital left brachium agenesis (amelia, brachiomelia monobrachium) in Sahel goat ecotype | | unrelated topic |
| 1. Ellaithi, M.; Gisselsson, D.; Nilsson, T.; Elagib, A.; Fadl-Elmula, I.; Abdelgadir, M. | 2007 | | A case of Cornelia de Lange syndrome from Sudan | | unrelated topic |
| 1. Amalaseelan, J. V.; Pieris, D. C.; Munasinghe, M. | 2010 | | A case of intra cardiac yolk sac tumour | | unrelated topic |
| 1. Samson, G.; Viljoen, D. | 1995 | | A case of lateral facial cleft, cleft lip and palate, anophthalmia, microtia, clavicular agenesis and asternia | | Year of publication |
| 1. Samuel, M. O.; Wachida, N.; Abenga, J. H.; Kisani, A. I.; Adamu, S. S.; Hambesha, P.; Gyang, E.; Oyedipe, E. O. | 2014 | | A case of omphalo-ischiopagus (dicephalic dithoracic abdominopagus tetrascelus tetrabrachius) in lambs | | unrelated topic |
| 1. Omobowale, T. O.; Olopade, J. O.; Usende, I. L.; Azeez, I. A. | 2013 | | A case of polydactyly in the hind-limbs of a West African Dwarf goat in South-West Nigeria | | unrelated topic |
| 1. Arigbede, A. O.; Adesuwa, A. A. | 2012 | | A case of quackery and obsession for diastema resulting in avoidable endodontic therapy | | unrelated topic |
| 1. Mapelli, M.; Zagni, P.; Calbi, V.; Twalib, A.; Ferrara, R.; Agostoni, P. | 2021 | | A Case of William's Syndrome in a Ugandan Child: A Feasible Diagnosis Even in a Low-Resource Setting | | bookSection |
| 1. Odongo, C. N.; Godier-Furnemont, A.; Moro, J.; Oyania, F. | 2023 | | A case report of an asymptomatic necrotic Meckel's diverticulum in an inguinal hernia during elective surgery in a resource limited setting: Littre's hernia | | unrelated topic |
| 1. Dohbit, J. S.; Meka, E.; Tochie, J. N.; Kamla, I.; Mwadjie, D.; Foumane, P. | 2017 | | A case report of bicornis bicollis uterus with unilateral cervical atresia: an unusual aetiology of chronic debilitating pelvic pain in a Cameroonian teenager | | unrelated topic |
| 1. Koning, M.; Koning, J.; Kancherla, V.; O'Neill, P.; Dorsey, A.; Zewdie, K.; Yesehak, B.; Ashagre, Y.; Woldermarium, M.; Biluts, H. | 2023 | | A case study of ReachAnother Foundation as a change champion for developing spina bifida neurosurgical care and advocating for primary prevention in Ethiopia | | unrelated topic |
| 1. Sakuma, M. | 1987 | | A case study of the mesiolingual randtubercle on the second maxillary molar in the Chewa dentition | | Year of publication |
| 1. Amoa, A. B.; Klufio, C. A.; Amos, L. | 2002 | | A case-control study of early neonatal deaths at the Port Moresby General Hospital to determine associated risk factors | | unrelated topic |
| 1. Watts, T.; Harris, R. R. | 1982 | | A case-control study of stillbirths at a teaching hospital in Zambia, 1979-80: antenatal factors | | Year of publication |
| 1. Amoa, A. B.; Klufio, C. A.; Moro, M.; Kariwiga, G.; Mola, G. | 1998 | | A case-control study of stillbirths at the Port Moresby General Hospital | | Year of publication |
| 1. Alighieri, C.; Kissel, I.; D'Haeseleer, E.; Bruneel, L.; Bettens, K.; Sseremba, D.; Pype, P.; Van Lierde, K. | 2020 | | A cleft care workshop for speech and language pathologists in resource-limited countries: The participants' experiences about cleft care in Uganda and satisfaction with the training effect | | unrelated topic |
| 1. Woods, D. L.; Draper, R. R. | 1980 | | A clinical assessment of stillborn infants | | Year of publication |
| 1. de Vries, P. J.; Wilde, L.; de Vries, M. C.; Moavero, R.; Pearson, D. A.; Curatolo, P. | 2018 | | A clinical update on tuberous sclerosis complex-associated neuropsychiatric disorders (TAND) | | unrelated topic |
| 1. Topley, J. M.; Dawodu, A. H.; Ammar, M. M. | 1995 | | A cluster of choanal atresia | | Year of publication |
| 1. Morgan, N. V.; Essop, F.; Demuth, I.; de Ravel, T.; Jansen, S.; Tischkowitz, M.; Lewis, C. M.; Wainwright, L.; Poole, J.; Joenje, H.; Digweed, M.; Krause, A.; Mathew, C. G. | 2005 | | A common Fanconi anemia mutation in black populations of sub-Saharan Africa | | unrelated topic |
| 1. Kim, J. W.; Hong, K. W.; Go, M. J.; Kim, S. S.; Tabara, Y.; Kita, Y.; Tanigawa, T.; Cho, Y. S.; Han, B. G.; Oh, B. | 2012 | | A common variant in SLC8A1 is associated with the duration of the electrocardiographic QT interval | | unrelated topic |
| 1. Chinawa, J. M.; Duru, C. O.; Chinawa, A. T.; Chukwu, B. F. | 2022 | | A comparative analysis on risk of pulmonary hypertension in children with Atrio-ventricular (AV) canal defect: a multi-centre study | | unrelated topic |
| 1. Carmeli, E.; Kessel, S.; Bar-Chad, S.; Merrick, J. | 2004 | | A comparison between older persons with down syndrome and a control group: clinical characteristics, functional status and sensorimotor function | | unrelated topic |
| 1. Smythe, T.; Gova, M.; Muzarurwi, R.; Foster, A.; Lavy, C. | 2018 | | A comparison of outcome measures used to report clubfoot treatment with the Ponseti method: results from a cohort in Harare, Zimbabwe | | unrelated topic |
| 1. Bulut, G.; Bulut, H.; Ortac, R. | 2019 | | A comprehensive survey of natal and neonatal teeth in newborns | | unrelated topic |
| 1. Pharoah, P. O.; Connolly, K. J. | 1987 | | A controlled trial of iodinated oil for the prevention of endemic cretinism: a long-term follow-up | | Year of publication |
| 1. Baab, K. L.; Brown, P.; Falk, D.; Richtsmeier, J. T.; Hildebolt, C. F.; Smith, K.; Jungers, W. | 2016 | | A Critical Evaluation of the Down Syndrome Diagnosis for LB1, Type Specimen of Homo floresiensis | | unrelated topic |
| 1. Grace, H. J.; Ally, F. E.; Nelemans, A. P.; Kint, B. | 1979 | | A cytogenetic study of a mentally retarded population in South Africa | | Year of publication |
| 1. Chouinard, A. | 1987 | | A deadly shadow: AIDS in Africa | | Year of publication |
| 1. Ndibazza, J.; Lule, S.; Nampijja, M.; Mpairwe, H.; Oduru, G.; Kiggundu, M.; Akello, M.; Muhangi, L.; Elliott, A. M. | 2011 | | A description of congenital anomalies among infants in Entebbe, Uganda | | unrelated topic |
| 1. Hummel, D. B.; Dansky, R.; Leahy, B.; Levin, S. E. | 1988 | | A disproportionately high incidence of symptomatic coarctation of the aorta in white infants in the Transvaal | | Year of publication |
| 1. Ritzman, T. B.; Baker, B. J.; Schwartz, G. T. | 2008 | | A fine line: a comparison of methods for estimating ages of linear enamel hypoplasia formation | | Duplicate |
| 1. Ritzman, T. B.; Baker, B. J.; Schwartz, G. T. | 2008 | | A fine line: a comparison of methods for estimating ages of linear enamel hypoplasia formation | | unrelated topic |
| 1. Vivian, L.; Comitis, G.; Naidu, C.; Hunter, C.; Lawrenson, J. | 2018 | | A first qualitative snapshot: cardiac surgery and recovery in 10 children in the Red Cross War Memorial Children's Hospital, Cape Town, South Africa (2011-2016) | | unrelated topic |
| 1. Roberts, L.; George, S.; Greenberg, J.; Ramesar, R. S. | 2015 | | A Founder Mutation in MYO7A Underlies a Significant Proportion of Usher Syndrome in Indigenous South Africans: Implications for the African Diaspora | | Duplicate |
| 1. Roberts, L.; George, S.; Greenberg, J.; Ramesar, R. S. | 2015 | | A Founder Mutation in MYO7A Underlies a Significant Proportion of Usher Syndrome in Indigenous South Africans: Implications for the African Diaspora | | unrelated topic |
| 1. Litz Philipsborn, S.; Hartmajer, S.; Shtorch Asor, A.; Vinovezky, M.; Regev, M.; Singer, A.; Reinstein, E. | 2021 | | A founder mutation in TCTN2 causes Meckel-Gruber syndrome type 8 among Jews of Ethiopian and Yemenite origin | | Duplicate |
| 1. Litz Philipsborn, S.; Hartmajer, S.; Shtorch Asor, A.; Vinovezky, M.; Regev, M.; Singer, A.; Reinstein, E. | 2021 | | A founder mutation in TCTN2 causes Meckel-Gruber syndrome type 8 among Jews of Ethiopian and Yemenite origin | | unrelated topic |
| 1. van de Putte, R.; Dworschak, G. C.; Brosens, E.; Reutter, H. M.; Marcelis, C. L. M.; Acuna-Hidalgo, R.; Kurtas, N. E.; Steehouwer, M.; Dunwoodie, S. L.; Schmiedeke, E.; Märzheuser, S.; Schwarzer, N.; Brooks, A. S.; de Klein, A.; Sloots, C. E. J.; Tibboel, D.; Brisighelli, G.; Morandi, A.; Bedeschi, M. F.; Bates, M. D.; Levitt, M. A.; Peña, A.; de Blaauw, I.; Roeleveld, N.; Brunner, H. G.; van Rooij, Ialm; Hoischen, A. | 2020 | | A Genetics-First Approach Revealed Monogenic Disorders in Patients With ARM and VACTERL Anomalies | | unrelated topic |
| 1. Leslie, E. J.; Liu, H.; Carlson, J. C.; Shaffer, J. R.; Feingold, E.; Wehby, G.; Laurie, C. A.; Jain, D.; Laurie, C. C.; Doheny, K. F.; McHenry, T.; Resick, J.; Sanchez, C.; Jacobs, J.; Emanuele, B.; Vieira, A. R.; Neiswanger, K.; Standley, J.; Czeizel, A. E.; Deleyiannis, F.; Christensen, K.; Munger, R. G.; Lie, R. T.; Wilcox, A.; Romitti, P. A.; Field, L. L.; Padilla, C. D.; Cutiongco-de la Paz, E. M.; Lidral, A. C.; Valencia-Ramirez, L. C.; Lopez-Palacio, A. M.; Valencia, D. R.; Arcos-Burgos, M.; Castilla, E. E.; Mereb, J. C.; Poletta, F. A.; Orioli, I. M.; Carvalho, F. M.; Hecht, J. T.; Blanton, S. H.; Buxó, C. J.; Butali, A.; Mossey, P. A.; Adeyemo, W. L.; James, O.; Braimah, R. O.; Aregbesola, B. S.; Eshete, M. A.; Deribew, M.; Koruyucu, M.; Seymen, F.; Ma, L.; de Salamanca, J. E.; Weinberg, S. M.; Moreno, L.; Cornell, R. A.; Murray, J. C.; Marazita, M. L. | 2016 | | A Genome-wide Association Study of Nonsyndromic Cleft Palate Identifies an Etiologic Missense Variant in GRHL3 | | unrelated topic |
| 1. Francke, S.; Manraj, M.; Lacquemant, C.; Lecoeur, C.; Leprêtre, F.; Passa, P.; Hebe, A.; Corset, L.; Yan, S. L.; Lahmidi, S.; Jankee, S.; Gunness, T. K.; Ramjuttun, U. S.; Balgobin, V.; Dina, C.; Froguel, P. | 2001 | | A genome-wide scan for coronary heart disease suggests in Indo-Mauritians a susceptibility locus on chromosome 16p13 and replicates linkage with the metabolic syndrome on 3q27 | | unrelated topic |
| 1. Xu, J | 2018 | | A Genome-Wide Screen for Essential Genes that Controls the Formation of Human Heart Progenitors | | book |
| 1. Zilla, P.; Bolman, 3rd, R. M.; Boateng, P.; Sliwa, K. | 2020 | | A glimpse of hope: cardiac surgery in low- and middle-income countries (LMICs) | | unrelated topic |
| 1. Hoeper, M. M.; Humbert, M.; Souza, R.; Idrees, M.; Kawut, S. M.; Sliwa-Hahnle, K.; Jing, Z. C.; Gibbs, J. S. | 2016 | | A global view of pulmonary hypertension | | unrelated topic |
| 1. Kolvenbach, C. M.; Zheng, B.; Merz, L. M.; Mertens, N. D.; Mansour, B.; Wang, C.; Seltzsam, S.; Schneider, S.; Schierbaum, L.; Pantel, D.; Chen, J.; van der Ven, A. T.; Bello, J. O.; Shril, S.; Hildebrandt, F. | 2023 | | A homozygous truncating ETV4 variant in a Nigerian family with congenital anomalies of the kidney and urinary tract | | unrelated topic |
| 1. Mumpe-Mwanja, D.; Barlow-Mosha, L.; Williamson, D.; Valencia, D.; Serunjogi, R.; Kakande, A.; Namale-Matovu, J.; Nankunda, J.; Birabwa-Male, D.; Okwero, M. A.; Nsungwa-Sabiiti, J.; Musoke, P. | 2019 | | A hospital-based birth defects surveillance system in Kampala, Uganda | | Duplicate |
| 1. Mumpe-Mwanja, D.; Barlow-Mosha, L.; Williamson, D.; Valencia, D.; Serunjogi, R.; Kakande, A.; Namale-Matovu, J.; Nankunda, J.; Birabwa-Male, D.; Okwero, M. A.; Nsungwa-Sabiiti, J.; Musoke, P. | 2019 | | A hospital-based birth defects surveillance system in Kampala, Uganda | | unrelated topic |
| 1. Christofides, E.; Potgieter, A.; Chait, L. | 2006 | | A long term subjective and objective assessment of the scar in unilateral cleft lip repairs using the Millard technique without revisional surgery | | unrelated topic |
| 1. Ceyhan, D.; Kirzioglu, Z.; Emek, T. | 2019 | | A long-term clinical study on individuals with amelogenesis imperfecta | | unrelated topic |
| 1. Shehata, A. H.; Aziz Ghandour, I. A. | 1990 | | A map of natural fluoride in drinking water in Sudan | | Year of publication |
|  | 1970 | | A mixed bag of natural carcinogens | | Year of publication |
| 1. Carlson, L. C.; Stewart, B. T.; Hatcher, K. W.; Kabetu, C.; VanderBurg, R.; Magee, Jr., W. P. | 2016 | | A Model of the Unmet Need for Cleft Lip and Palate Surgery in Low- and Middle-Income Countries | | unrelated topic |
| 1. Wilson, W. D.; Johnson, P. T.; Sutherland, D. R.; Moné, H.; Loker, E. S. | 2005 | | A molecular phylogenetic study of the genus Ribeiroia (Digenea): trematodes known to cause limb malformations in amphibians | | unrelated topic |
| 1. Leslie, E. J.; Carlson, J. C.; Shaffer, J. R.; Feingold, E.; Wehby, G.; Laurie, C. A.; Jain, D.; Laurie, C. C.; Doheny, K. F.; McHenry, T.; Resick, J.; Sanchez, C.; Jacobs, J.; Emanuele, B.; Vieira, A. R.; Neiswanger, K.; Lidral, A. C.; Valencia-Ramirez, L. C.; Lopez-Palacio, A. M.; Valencia, D. R.; Arcos-Burgos, M.; Czeizel, A. E.; Field, L. L.; Padilla, C. D.; Cutiongco-de la Paz, E. M.; Deleyiannis, F.; Christensen, K.; Munger, R. G.; Lie, R. T.; Wilcox, A.; Romitti, P. A.; Castilla, E. E.; Mereb, J. C.; Poletta, F. A.; Orioli, I. M.; Carvalho, F. M.; Hecht, J. T.; Blanton, S. H.; Buxó, C. J.; Butali, A.; Mossey, P. A.; Adeyemo, W. L.; James, O.; Braimah, R. O.; Aregbesola, B. S.; Eshete, M. A.; Abate, F.; Koruyucu, M.; Seymen, F.; Ma, L.; de Salamanca, J. E.; Weinberg, S. M.; Moreno, L.; Murray, J. C.; Marazita, M. L. | 2016 | | A multi-ethnic genome-wide association study identifies novel loci for non-syndromic cleft lip with or without cleft palate on 2p24.2, 17q23 and 19q13 | | unrelated topic |
| 1. Nunes, M. A. S.; Magalhães, M. P.; Uva, M. S.; Heitor, P.; Henriques, A.; Manuel, V.; Miguel, G.; Júnior, A. F. | 2017 | | A multinational and multidisciplinary approach to treat CHD in paediatric age in Angola: initial experience of a medical-surgical centre for children with heart disease in Angola | | unrelated topic |
| 1. Kibr, G. | 2021 | | A Narrative Review of Nutritional Malpractices, Motivational Drivers, and Consequences in Pregnant Women: Evidence from Recent Literature and Program Implications in Ethiopia | | unrelated topic |
| 1. Venter, M.; Theron, E.; Williams, W.; Khan, W.; Stassen, W. | 2021 | | A national retrospective review of neonatal critical care transfers in dedicated critical care transport services in the private sector | | unrelated topic |
| 1. Ersek, R. A.; Kjellstrand, C.; Najarian, J. S.; Lillehei, R. C. | 1969 | | A new arterionous shunt design | | Year of publication |
| 1. Cartault, F.; Nava, C.; Malbrunot, A. C.; Munier, P.; Hebert, J. C.; N'Guyen, P.; Djeridi, N.; Pariaud, P.; Pariaud, J.; Dupuy, A.; Austerlitz, F.; Sarasin, A. | 2011 | | A new XPC gene splicing mutation has lead to the highest worldwide prevalence of xeroderma pigmentosum in black Mahori patients | | unrelated topic |
| 1. Musa, B. E. | 1984 | | A note on some abnormalities and anomalies in camels (Camelus dromedarius) | | Year of publication |
| 1. Yalcouyé, A.; Diallo, S. H.; Coulibaly, T.; Cissé, L.; Diallo, S.; Samassékou, O.; Diarra, S.; Coulibaly, D.; Keita, M.; Guinto, C. O.; Fischbeck, K.; Landouré, G. | 2019 | | A novel mutation in the GARS gene in a Malian family with Charcot-Marie-Tooth disease | | unrelated topic |
| 1. Pagnamenta, A. T.; Murray, J. E.; Yoon, G.; Sadighi Akha, E.; Harrison, V.; Bicknell, L. S.; Ajilogba, K.; Stewart, H.; Kini, U.; Taylor, J. C.; Keays, D. A.; Jackson, A. P.; Knight, S. J. | 2012 | | A novel nonsense CDK5RAP2 mutation in a Somali child with primary microcephaly and sensorineural hearing loss | | unrelated topic |
| 1. Durmus, B.; Durhan, A.; Gökkaya, B.; Kıtıki, B.; Yanıkoğlu, F.; Kargül, B. | 2017 | | A novel quantitative light-induced fluorescence device for monitoring molar-incisor hypomineralization | | unrelated topic |
| 1. du Toit, S. N. | 1973 | | A one-year survey of a rural orthopaedic clinic in Zululand | | Year of publication |
| 1. Sodipo, J. O. | 1975 | | A paediatric respiratory intensive care unit in Lagos | | Year of publication |
| 1. Kadambari, S. | 2007 | | A patient's journey | | unrelated topic |
| 1. Mnguni, M. N.; Enicker, B. C.; Madiba, T. E. | 2020 | | A perspective in the management of myelomeningocoele in the KwaZulu-Natal Province of South Africa | | unrelated topic |
| 1. Biselele, T.; Bambi, J.; Betukumesu, D. M.; Ndiyo, Y.; Tabu, G.; Kapinga, J.; Bola, V.; Makaya, P.; Tjabbes, H.; Vis, P.; Peeters-Scholte, C. | 2020 | | A Phase IIa Clinical Trial of 2-Iminobiotin for the Treatment of Birth Asphyxia in DR Congo, a Low-Income Country | | unrelated topic |
| 1. Swanepoel, M.; Haw, T. | 2018 | | A pilot study evaluating depression in mothers with children diagnosed with Down syndrome in state health care | | unrelated topic |
| 1. Msokera, C.; Xepoleas, M.; Collier, Z. J.; Naidu, P.; Magee, 3rd, W. | 2022 | | A plastic and reconstructive surgery landscape assessment of Malawi: a scoping review of Malawian literature | | unrelated topic |
| 1. Thylstrup, A.; Fejerskov, O.; Mosha, H. J. | 1978 | | A polarized light and microradiographic study of enamel in human primary teeth from a high fluoride area | | Year of publication |
| 1. Hoyme, H. E.; May, P. A.; Kalberg, W. O.; Kodituwakku, P.; Gossage, J. P.; Trujillo, P. M.; Buckley, D. G.; Miller, J. H.; Aragon, A. S.; Khaole, N.; Viljoen, D. L.; Jones, K. L.; Robinson, L. K. | 2005 | | A practical clinical approach to diagnosis of fetal alcohol spectrum disorders: clarification of the 1996 institute of medicine criteria | | unrelated topic |
| 1. Nkoke, C.; Makoge, C.; Dzudie, A.; Mfeukeu, L. K.; Luchuo, E. B.; Menanga, A.; Kingue, S. | 2017 | | A predominance of hypertensive heart disease among patients with cardiac disease in Buea, a semi-urban setting, South West Region of Cameroon | | unrelated topic |
| 1. Murphy, K. A. | 1999 | | A prehistoric example of polydactyly from the Iron Age site of Simbusenga, Zambia | | Year of publication |
| 1. El Wisy, A. B. | 1976 | | A preliminary study of the genital organs of indigenous cows in Uganda | | Year of publication |
| 1. Behrens, K. G. | 2020 | | A principled ethical approach to intersex paediatric surgeries | | unrelated topic |
| 1. Towle, I.; Irish, J. D. | 2019 | | A probable genetic origin for pitting enamel hypoplasia on the molars of Paranthropus robustus | | unrelated topic |
| 1. Shija, J. K.; Kingo, A. R. | 1985 | | A prospective clinical study of congenital anomalies seen at Harare Central Hospital, Zimbabwe | | Year of publication |
| 1. Adeboye, M.; Abdulkadir, M. B.; Adegboye, O. A.; Saka, A. O.; Oladele, P. D.; Oladele, D. M.; Eze, E. C.; Adeyemi, O. O.; Abubakar, U.; Grace, A.; Rotimi, B. F. | 2016 | | A Prospective Study of Spectrum, Risk Factors and Immediate Outcome of Congenital Anomalies in Bida, North Central Nigeria | | unrelated topic |
| 1. Donkor, P.; Plange-Rhule, G.; Amponsah, E. K. | 2007 | | A prospective survey of patients with cleft lip and palate in Kumasi | | unrelated topic |
| 1. Ambachew, R.; Gulilat, A.; Aberra, T.; Terefework, Z.; Bedilu, W.; Tarekegn, G.; Reja, A. | 2022 | | A rare case of 46,XX gonadal dysgenesis, Mayer-Rokitansky-Kuster-Hauser syndrome, pituitary and thyroid hypoplasia | | unrelated topic |
| 1. Chale-Matsau, B.; Kemp, T.; van Hougenhouck-Tulleken, W.; Karsas, M.; Pillay, T. S. | 2020 | | A Rare Cause of Virilization, Short Stature, and Hypertension | | unrelated topic |
| 1. Winterton, D. M.; Jooma, U.; Cox, S. G. | 2023 | | A rare occurrence of multiple intestinal atresias, with successful one-procedure resection and primary anastomosis | | unrelated topic |
| 1. Awuah, W. A.; Adebusoye, F. T.; Wellington, J.; Ghosh, S.; Tenkorang, P. O.; Machai, P. N. M.; Abdul-Rahman, T.; Mani, S.; Salam, A.; Papadakis, M. | 2023 | | A reflection of Africa's cardiac surgery capacity to manage congenital heart defects: a perspective | | unrelated topic |
| 1. Van der Merwe, A. E.; Steyn, M. | 2009 | | A report on the high incidence of supernumerary teeth in skeletal remains from a 19th century mining community from Kimberley, South Africa | | unrelated topic |
| 1. Hong, H.; Malfeld, S.; Smit, S.; Makhathini, L.; Fortuin, M.; Motsamai, T.; Tselana, D.; Manamela, M. J.; Motaze, N. V.; Ntshoe, G.; Kamupira, M.; Khosa-Lesola, E.; Mokoena, S.; Buthelezi, T.; Maseti, E.; Suchard, M. | 2022 | | A retrospective 5-year review of rubella in South Africa prior to the introduction of a rubella-containing vaccine | | unrelated topic |
| 1. Thanni, L. O.; Shonubi, A. M.; Akiode, O. | 2005 | | A retrospective audit of paediatric surgical admission in a sub-urban tertiary hospital | | unrelated topic |
| 1. Abdul-Mumin, A.; Cotache-Condor, C.; Owusu, S. A.; Grimm, A.; Mahama, H.; Wright, N.; Abantanga, F. A.; Smith, E. R.; Tabiri, S. | 2021 | | A retrospective review of gastroschisis epidemiology and referral patterns in northern Ghana | | unrelated topic |
| 1. Beringer, N.; Cilliers, A. | 2019 | | A retrospective review of right-sided hearts at a South African tertiary hospital | | unrelated topic |
| 1. Ogar, C. K.; Abiola, A.; Yuah, D.; Ibrahim, A.; Oreagba, I. A.; Amadi, E. C.; Adeyeye, M. C.; Oshikoya, K. A. | 2019 | | A Retrospective Review of Serious Adverse Drug Reaction Reports in the Nigerian VigiFlow Database from September 2004 to December 2016 | | unrelated topic |
| 1. Sarica, I.; Derindag, G.; Kurtuldu, E.; Naralan, M. E.; Caglayan, F. | 2019 | | A retrospective study: Do all impacted teeth cause pathology? | | unrelated topic |
| 1. Klufio, C. A.; Ardayfio, S. A.; Nartey, I. N.; Kissi, S. A. | 1973 | | A retrospective survey of caesarean sections at Korle Bu Teaching Hospital Accra: 1971–a review of 1077 cases | | Year of publication |
| 1. Anyanechi, C. E.; Saheeb, B. D. | 2014 | | A review of 156 odontogenic tumours in Calabar, Nigeria | | unrelated topic |
| 1. Moore, S. W.; Zaahl, M. G. | 2008 | | A review of genetic mutation in familial Hirschsprung's disease in South Africa: towards genetic counseling | | Duplicate |
| 1. Moore, S. W.; Zaahl, M. G. | 2008 | | A review of genetic mutation in familial Hirschsprung's disease in South Africa: towards genetic counseling | | unrelated topic |
| 1. Malherbe, H. L.; Modell, B.; Blencowe, H.; Strong, K. L.; Aldous, C. | 2023 | | A review of key terminology and definitions used for birth defects globally | | unrelated topic |
| 1. Hasford, F; Mumuni, AN; Trauernicht, C; Ige, TA; ... | 2022 | | A review of MRI studies in Africa with special focus on quantitative MRI: Historical development, current status and the role of medical physicists | | unrelated topic |
| 1. Mahungu, A. C.; Monnakgotla, N.; Nel, M.; Heckmann, J. M. | 2022 | | A review of the genetic spectrum of hereditary spastic paraplegias, inherited neuropathies and spinal muscular atrophies in Africans | | unrelated topic |
| 1. Onipe, T.; Edokpayi, J. N.; Odiyo, J. O. | 2020 | | A review on the potential sources and health implications of fluoride in groundwater of Sub-Saharan Africa | | unrelated topic |
| 1. Cohen, K.; Maartens, G. | 2019 | | A safety evaluation of bedaquiline for the treatment of multi-drug resistant tuberculosis | | unrelated topic |
| 1. Mekonen, H. K.; Berhe, Y.; Berihu, B. A.; Teka, H.; Hadgu, A.; Gebregziabher, L.; Berhe, E. H.; Magana, T.; Mulugeta, A. | 2021 | | A silent epidemic of major congenital malformations in Tigray, northern Ethiopia: hospital-based study | | unrelated topic |
| 1. Kwan, G. F.; Bukhman, A. K.; Miller, A. C.; Ngoga, G.; Mucumbitsi, J.; Bavuma, C.; Dusabeyezu, S.; Rich, M. L.; Mutabazi, F.; Mutumbira, C.; Ngiruwera, J. P.; Amoroso, C.; Ball, E.; Fraser, H. S.; Hirschhorn, L. R.; Farmer, P.; Rusingiza, E.; Bukhman, G. | 2013 | | A simplified echocardiographic strategy for heart failure diagnosis and management within an integrated noncommunicable disease clinic at district hospital level for sub-Saharan Africa | | unrelated topic |
| 1. Hertroijs, A. R. | 1974 | | A study of some factors affecting the attendance of patients in a leprosy control scheme | | Year of publication |
| 1. Nasir, A. A.; Ameh, E. A. | 2014 | | A survey of current practices in management of Hirschsprung's disease in Nigeria | | unrelated topic |
| 1. Wet, M De | 2013 | | A systematic health assessment of two dolphin species by-caught in shark nets off the KwaZulu-Natal coast, South Africa | | book |
| 1. Abdulkadir, M.; Abdulkadir, Z. | 2016 | | A systematic review of trends and patterns of congenital heart disease in children in Nigeria from 1964-2015 | | unrelated topic |
| 1. Malaza, N.; Masete, M.; Adam, S.; Dias, S.; Nyawo, T.; Pheiffer, C. | 2022 | | A Systematic Review to Compare Adverse Pregnancy Outcomes in Women with Pregestational Diabetes and Gestational Diabetes | | unrelated topic |
| 1. Adetiloye, V. A.; Dare, F. O.; Oyelami, O. A. | 1993 | | A ten-year review of encephalocele in a teaching hospital | | Year of publication |
| 1. Manyama, M.; Rambau, P.; Gilyoma, J.; Mahalu, W. | 2011 | | A variant branching pattern of the aortic arch: a case report | | unrelated topic |
| 1. Obwolo, M. J.; Lawson, G. | 1992 | | Abattoir survey of abnormal female porcine genital tracts in Zimbabwe | | Year of publication |
| 1. Chamberlain, G; Morgan, M | 2002 | | ABC of antenatal care | | book |
| 1. 2016 | 2016 | | Abdulkadir, M.; Abdulkadir, Z. | | non primary research |
| 1. Nwaneli, E. I.; Chukwuka, J. O.; Uju, C. M.; Epundu, C. O. | 2021 | | Ablepharon macrostomia syndrome: Absent prepuce in the first case report in West Africa | | bookSection |
| 1. Adekanye, A. O.; Adefemi, S. A.; Onawola, K. A.; James, J. A.; Adeleke, I. T.; Francis, M.; Sheshi, E. U.; Atakere, M. E.; Jibril, A. D. | 2017 | | Abnormalities of the external genitalia and groins among primary school boys in Bida, Nigeria | | unrelated topic |
| 1. Na, I.; Van Bulck, L.; Rassart, J.; Goossens, E.; Luyckx, K.; Van De Bruaene, A.; Moons, P. | 2022 | | Absence from work or school in young adults with congenital heart disease: is illness identity associated with absenteeism? | | unrelated topic |
| 1. Beresford, R. G.; Tatlidil, C.; Riddell, D. C.; Welch, J. P.; Ludman, M. D.; Neumann, P. E.; Greer, W. L. | 2000 | | Absence of fragile X syndrome in Nova Scotia | | unrelated topic |
| 1. Ajayi, N. O.; Lazarus, L.; Vanker, E. A.; Satyapal, K. S. | 2015 | | Absent left main coronary artery with variation in the origin of its branches in a South African population | | unrelated topic |
| 1. Congress, SAH | 2018 | | Abstracts: SA Heart Congress 2018 | | book |
| 1. Doubell, AF | 2009 | | Abstracts-SA Heart Congress 2009 | | book |
| 1. Congress, SAH | 2017 | | Abstracts-SA Heart Congress 2017 | | Duplicate |
| 1. Bode, C. O.; Odelola, M. A.; Odiachi, R. O. | 2001 | | Abuse and neglect in the surgically ill child | | unrelated topic |
| 1. Rowlands, A.; Deeb, A.; Ladjouze, A.; Hamza, R. T.; Musa, S. A.; Raza, J.; Jennane, F.; Abu-Libdeh, A.; Chanoine, J. P. | 2021 | | Access to fludrocortisone and to hydrocortisone in children with congenital adrenal hyperplasia in the WHO Eastern Mediterranean Region: it takes a village… | | unrelated topic |
| 1. Vlok, S. S. C.; Moore, S. W.; Schubert, P. T.; Pitcher, R. D. | 2020 | | Accuracy of colonic mucosal patterns at contrast enema for diagnosis of Hirschsprung disease | | unrelated topic |
| 1. Calbiani, F.; Careri, M.; Elviri, L.; Mangia, A.; Zagnoni, I. | 2004 | | Accurate mass measurements for the confirmation of Sudan azo-dyes in hot chilli products by capillary liquid chromatography-electrospray tandem quadrupole orthogonal-acceleration time of flight mass spectrometry | | unrelated topic |
| 1. Moore, S. W.; Johnson, G. | 2005 | | Acetylcholinesterase in Hirschsprung's disease | | unrelated topic |
| 1. Raza, J.; Mazen, I. | 2014 | | Achieving diagnostic certainty in resource-limited settings | | unrelated topic |
| 1. Perovic, O.; Duse, A.; Chibabhai, V.; Black, M.; Said, M.; Prentice, E.; Wadula, J.; Mahabeer, Y.; Han, K. S. S.; Mogokotleng, R.; Strasheim, W.; Lowe, M.; Jallow, S.; Ismail, H. | 2022 | | Acinetobacter baumannii complex, national laboratory-based surveillance in South Africa, 2017 to 2019 | | unrelated topic |
| 1. Curry, C.; Zuhlke, L.; Mocumbi, A.; Kennedy, N. | 2018 | | Acquired heart disease in low-income and middle-income countries | | unrelated topic |
| 1. Rossouw, J. E.; Steyn, K.; Berger, G. M.; Vermaak, W. J.; Kock, J.; Seftel, H. C.; Gevers, W. | 1988 | | Action limits for serum total cholesterol. A statement for the medical profession by an ad hoc committee of the Heart Foundation of Southern Africa | | Year of publication |
| 1. Okello, E.; Ndagire, E.; Atala, J.; Bowen, A. C.; DiFazio, M. P.; Harik, N. S.; Longenecker, C. T.; Lwabi, P.; Murali, M.; Norton, S. A.; Omara, I. O.; Oyella, L. M.; Parks, T.; Pulle, J.; Rwebembera, J.; Sarnacki, R. J.; Spurney, C. F.; Stein, E.; Tochen, L.; Watkins, D.; Zimmerman, M.; Carapetis, J. R.; Sable, C.; Beaton, A. | 2020 | | Active Case Finding for Rheumatic Fever in an Endemic Country | | unrelated topic |
| 1. Zhou, M.; Ma, X.; Sun, J.; Ding, G.; Cui, Q.; Miao, Y.; Hou, Y.; Jiang, M.; Bai, G. | 2017 | | Active fragments-guided drug discovery and design of selective tropane alkaloids using ultra-high performance liquid chromatography-quadrupole time-of-flight tandem mass spectrometry coupled with virtual calculation and biological evaluation | | unrelated topic |
| 1. Risteski, T; Andonovska, B; Ardjanova, M | 2022 | | Acute appendicitis during the COVID-19 pandemic versus before pandemic period in Republic of North Macedonia | | book |
| 1. Eyong, K.; Torty, C.; Asindi, A.; Ekanem, E. | 2020 | | Acute hemiplegia: aetiology and outcome in Nigerian children | | unrelated topic |
| 1. Alabi, B. S.; Ologe, F. E.; Dunmade, A. D.; Segun-Busari, S.; Olatoke, F. | 2006 | | Acute laryngeal obstruction in a Nigerian Hospital: Clinical presentation and management | | unrelated topic |
| 1. Ranjith, N.; Verho, N. K.; Verho, M.; Winkelmann, B. R. | 2002 | | Acute myocardial infarction in a young South African Indian-based population: patient characteristics on admission and gender-specific risk factor prevalence | | unrelated topic |
| 1. White, D. A.; Zar, H. J.; Madhi, S. A.; Jeena, P.; Morrow, B.; Masekela, R.; Risenga, S.; Green, R. | 2016 | | Acute viral bronchiolitis in South Africa: Diagnostic flow | | unrelated topic |
| 1. Padrini, R.; Speranza, G.; Nollo, G.; Bova, S.; Piovan, D.; Antolini, R.; Ferrari, M. | 1997 | | Adaptation of the QT interval to heart rate changes in isolated perfused guinea pig heart: influence of amiodarone and D-sotalol | | Year of publication |
| 1. Miller, L.; Schmidt, C. N.; Wanduru, P.; Wanyoro, A.; Santos, N.; Butrick, E.; Lester, F.; Otieno, P.; Walker, D. | 2023 | | Adapting the preterm birth phenotyping framework to a low-resource, rural setting and applying it to births from Migori County in western Kenya | | Duplicate |
| 1. Miller, L.; Schmidt, C. N.; Wanduru, P.; Wanyoro, A.; Santos, N.; Butrick, E.; Lester, F.; Otieno, P.; Walker, D. | 2023 | | Adapting the preterm birth phenotyping framework to a low-resource, rural setting and applying it to births from Migori County in western Kenya | | unrelated topic |
| 1. van der Knoop, B. J.; Zonnenberg, I. A.; Verbeke, Jiml; de Vries, L. S.; Pistorius, L. R.; van Weissenbruch, M. M.; Vermeulen, R. J.; de Vries, J. I. P. | 2020 | | Additional value of advanced neurosonography and magnetic resonance imaging in fetuses at risk for brain damage | | unrelated topic |
| 1. Keet, K. | 2018 | | Addressing publication bias in the anatomical literature by reporting zero prevalence of bicuspid aortic valve | | unrelated topic |
| 1. Sendeku, F. W.; Azeze, G. G.; Fenta, S. L. | 2020 | | Adherence to iron-folic acid supplementation among pregnant women in Ethiopia: a systematic review and meta-analysis | | unrelated topic |
| 1. Sendeku, F. W.; Azeze, G. G.; Fenta, S. L. | 2020 | | Adherence to iron-folic acid supplementation among pregnant women in Ethiopia: a systematic review and meta-analysis | | unrelated topic |
| 1. Curatolo, P.; Franz, D. N.; Lawson, J. A.; Yapici, Z.; Ikeda, H.; Polster, T.; Nabbout, R.; de Vries, P. J.; Dlugos, D. J.; Fan, J.; Ridolfi, A.; Pelov, D.; Voi, M.; French, J. A. | 2018 | | Adjunctive everolimus for children and adolescents with treatment-refractory seizures associated with tuberous sclerosis complex: post-hoc analysis of the phase 3 EXIST-3 trial | | unrelated topic+B2572:B2583 |
| 1. French, J. A.; Lawson, J. A.; Yapici, Z.; Ikeda, H.; Polster, T.; Nabbout, R.; Curatolo, P.; de Vries, P. J.; Dlugos, D. J.; Berkowitz, N.; Voi, M.; Peyrard, S.; Pelov, D.; Franz, D. N. | 2016 | | Adjunctive everolimus therapy for treatment-resistant focal-onset seizures associated with tuberous sclerosis (EXIST-3): a phase 3, randomised, double-blind, placebo-controlled study | | unrelated topic |
| 1. Franz, D. N.; Lawson, J. A.; Yapici, Z.; Ikeda, H.; Polster, T.; Nabbout, R.; Curatolo, P.; de Vries, P. J.; Dlugos, D. J.; Herbst, F.; Peyrard, S.; Pelov, D.; French, J. A. | 2021 | | Adjunctive everolimus therapy for tuberous sclerosis complex-associated refractory seizures: Results from the postextension phase of EXIST-3 | | unrelated topic |
| 1. Oladele, A. O.; Olabanji, J. K.; Awe, O. O. | 2012 | | Adolescent and adult cleft lip and palate, in Ile-Ife, Nigeria | | unrelated topic |
| 1. M'Bede, J. | 1985 | | Adolescent pregnancy in Africa | | Year of publication |
| 1. Fisher, B. W. | 1990 | | Adult heart disease in Mt Hagen: a study of 154 patients | | Year of publication |
| 1. Troost, E.; Roggen, L.; Goossens, E.; Moons, P.; De Meester, P.; Van De Bruaene, A.; Budts, W. | 2019 | | Advanced care planning in adult congenital heart disease: Transitioning from repair to palliation and end-of-life care | | unrelated topic |
| 1. Golan, A.; Sandbank, O.; Andronikou, A.; Rubin, A. | 1985 | | Advanced extra-uterine pregnancy | | Year of publication |
| 1. Degno, S.; Lencha, B.; Aman, R.; Atlaw, D.; Mekonnen, A.; Woldeyohannes, D.; Tekalegn, Y.; Hailu, S.; Woldemichael, B.; Nigussie, A. | 2021 | | Adverse birth outcomes and associated factors among mothers who delivered in Bale zone hospitals, Oromia Region, Southeast Ethiopia | | unrelated topic |
| 1. Gedefaw, G.; Alemnew, B.; Demis, A. | 2020 | | Adverse fetal outcomes and its associated factors in Ethiopia: a systematic review and meta-analysis | | Duplicate |
| 1. Gedefaw, G.; Alemnew, B.; Demis, A. | 2020 | | Adverse fetal outcomes and its associated factors in Ethiopia: a systematic review and meta-analysis | | unrelated topic |
| 1. Zheleva, B.; Verstappen, A.; Overman, D. M.; Ahmad, F.; Ali, S. K. M.; Al Halees, Z. Y.; Atallah, J. G.; Badhwar, I. E.; Baker-Smith, C.; Balestrini, M.; Basken, A.; Bassuk, J. S.; Benson, L.; Capelli, H.; Carollo, S.; Chowdhury, D.; Çiçek, M. S.; Cohen, M. I.; Cooper, D. S.; Deanfield, J. E.; Dearani, J.; Del Valle, B.; Dodds, K. M.; Du, J.; Edwin, F.; Ekure, E.; Fatema, N. N.; Gomanju, A.; Hasan, B.; Henry, L.; Hugo-Hamman, C.; Iyer, K. S.; Jatene, M. B.; Jenkins, K. J.; Karamlou, T.; Karl, T. R.; Kirklin, J. K.; Kreutzer, C.; Kumar, R. K.; Lopez, K. N.; Macedo, A. P.; Marino, B. S.; Marwali, E. M.; Meijboom, F. J.; Mattos, S. S.; Najm, H.; Newlin, D.; Novick, W. M.; Qureshi, S. S. A.; Rahmat, B.; Raylman, R.; Saltik, I. L.; Sable, C.; Sandoval, N.; Saxena, A.; Scanlan, E.; Sholler, G. F.; Smith, J.; St Louis, J. D.; Tchervenkov, C. I.; Tiong, K. G.; Vida, V.; Vosloo, S.; Weinstein, D. J. D.; Wilkinson, J. L.; Zuhlke, L.; Jacobs, J. P. | 2023 | | Advocacy at the Eighth World Congress of Pediatric Cardiology and Cardiac Surgery | | bookSection |
| 1. Oti, A. A.; Obiri-Yeboah, S.; Donkor, P. | 2014 | | Aesthetic outcome and the need for revision of unilateral cleft lip repair at Komfo Anokye Teaching Hospital | | unrelated topic |
| 1. Aukrust, C. G.; Paulsen, A. H.; Uche, E. O.; Kamalo, P. D.; Sandven, I.; Fjeld, H. E.; Strømme, H.; Eide, P. K. | 2022 | | Aetiology and diagnostics of paediatric hydrocephalus across Africa: a systematic review and meta-analysis | | unrelated topic |
| 1. Ndahetuye, J. B.; Persson, Y.; Nyman, A. K.; Tukei, M.; Ongol, M. P.; Båge, R. | 2019 | | Aetiology and prevalence of subclinical mastitis in dairy herds in peri-urban areas of Kigali in Rwanda | | unrelated topic |
| 1. Animashaun, A. | 1971 | | Aetiology of cerebral palsy in African children | | Year of publication |
| 1. Amoah, A. G.; Kallen, C. | 2000 | | Aetiology of heart failure as seen from a National Cardiac Referral Centre in Africa | | unrelated topic |
| 1. Izuora, G. I. | 1985 | | Aetiology of mental retardation in Nigerian children around Enugu | | Year of publication |
| 1. Adetayo, O.; Ford, R.; Martin, M. | 2012 | | Africa has unique and urgent barriers to cleft care: lessons from practitioners at the Pan-African Congress on Cleft Lip and Palate | | unrelated topic |
| 1. Yuko-Jowi, C. A. | 2012 | | African experiences of humanitarian cardiovascular medicine: a Kenyan perspective | | unrelated topic |
| 1. Rwenyonyi, C. M.; Birkeland, J. M.; Haugejorden, O.; Bjorvatn, K. | 2000 | | Age as a determinant of severity of dental fluorosis in children residing in areas with 0.5 and 2.5 mg fluoride per liter in drinking water | | unrelated topic |
| 1. Cleaton-Jones, P. | 1970 | | Agenesis and peg-shaped permanent maxillary lateral incisors in Kalahari Bushmen | | Year of publication |
| 1. Heeren, G. A.; Tyler, J.; Mandeya, A. | 2003 | | Agricultural chemical exposures and birth defects in the Eastern Cape Province, South Africa: a case-control study | | Duplicate |
| 1. Heeren, G. A.; Tyler, J.; Mandeya, A. | 2003 | | Agricultural chemical exposures and birth defects in the Eastern Cape Province, South Africa: a case-control study | | unrelated topic |
| 1. Na'uzo, A. M.; Tahir, A.; Lawal, T. O.; Sanni, U. A.; Ahmad, M. M.; Muhammad, N.; Sule, M. B.; Ahmed, H. | 2023 | | Aicardi syndrome in a Nigerian female child: A case report and literature review of a rare neuro-developmental disorder from North-Western Nigeria | | unrelated topic |
| 1. Sissolak, G.; Mayaud, P. | 2005 | | AIDS-related Kaposi's sarcoma: epidemiological, diagnostic, treatment and control aspects in sub-Saharan Africa | | unrelated topic |
| 1. Daynes, W. G. | 1973 | | Ainhum: its possible causation by ingestion of plants | | Year of publication |
| 1. Lou, B.; Barbieri, D. M.; Passavanti, M.; Hui, C.; Gupta, A.; Hoff, I.; Lessa, D. A.; Sikka, G.; Chang, K.; Fang, K.; Lam, L.; Maharaj, B.; Ghasemi, N.; Qiao, Y.; Adomako, S.; Foroutan Mirhosseini, A.; Naik, B.; Banerjee, A.; Wang, F.; Tucker, A.; Liu, Z.; Wijayaratna, K.; Naseri, S.; Yu, L.; Chen, H.; Shu, B.; Goswami, S.; Peprah, P.; Hessami, A.; Abbas, M.; Agarwal, N. | 2022 | | Air pollution perception in ten countries during the COVID-19 pandemic | | unrelated topic |
| 1. King, R. A.; Creel, D.; Cervenka, J.; Okoro, A. N.; Witkop, C. J. | 1980 | | Albinism in Nigeria with delineation of new recessive oculocutaneous type | | Year of publication |
| 1. Beulens, J. W.; Algra, A.; Soedamah-Muthu, S. S.; Visseren, F. L.; Grobbee, D. E.; van der Graaf, Y. | 2010 | | Alcohol consumption and risk of recurrent cardiovascular events and mortality in patients with clinically manifest vascular disease and diabetes mellitus: the Second Manifestations of ARTerial (SMART) disease study | | unrelated topic |
| 1. Ricci, C.; Wood, A.; Muller, D.; Gunter, M. J.; Agudo, A.; Boeing, H.; van der Schouw, Y. T.; Warnakula, S.; Saieva, C.; Spijkerman, A.; Sluijs, I.; Tjønneland, A.; Kyrø, C.; Weiderpass, E.; Kühn, T.; Kaaks, R.; Sánchez, M. J.; Panico, S.; Agnoli, C.; Palli, D.; Tumino, R.; Engström, G.; Melander, O.; Bonnet, F.; Boer, J. M. A.; Key, T. J.; Travis, R. C.; Overvad, K.; Verschuren, W. M. M.; Quirós, J. R.; Trichopoulou, A.; Papatesta, E. M.; Peppa, E.; Iribas, C. M.; Gavrila, D.; Forslund, A. S.; Jansson, J. H.; Matullo, G.; Arriola, L.; Freisling, H.; Lassale, C.; Tzoulaki, I.; Sharp, S. J.; Forouhi, N. G.; Langenberg, C.; Saracci, R.; Sweeting, M.; Brennan, P.; Butterworth, A. S.; Riboli, E.; Wareham, N. J.; Danesh, J.; Ferrari, P. | 2018 | | Alcohol intake in relation to non-fatal and fatal coronary heart disease and stroke: EPIC-CVD case-cohort study | | unrelated topic |
| 1. Morava, E.; Tiemes, V.; Thiel, C.; Seta, N.; de Lonlay, P.; de Klerk, H.; Mulder, M.; Rubio-Gozalbo, E.; Visser, G.; van Hasselt, P.; Horovitz, D. D. G.; de Souza, C. F. M.; Schwartz, I. V. D.; Green, A.; Al-Owain, M.; Uziel, G.; Sigaudy, S.; Chabrol, B.; van Spronsen, F. J.; Steinert, M.; Komini, E.; Wurm, D.; Bevot, A.; Ayadi, A.; Huijben, K.; Dercksen, M.; Witters, P.; Jaeken, J.; Matthijs, G.; Lefeber, D. J.; Wevers, R. A. | 2016 | | ALG6-CDG: a recognizable phenotype with epilepsy, proximal muscle weakness, ataxia and behavioral and limb anomalies | | unrelated topic |
| 1. Hegele, R. A.; Ban, M. R.; Carrington, C. V.; Ramdath, D. D. | 2001 | | Allele frequencies for candidate genes in atherosclerosis and diabetes among Trinidadian neonates | | unrelated topic |
| 1. Mudekereza, P. S.; Mudekereza, J. B.; Bahizire, G. M.; Lekuya, H. M.; Mudekereza, E. A.; Zabadayi, G. M.; Budema, P. M.; Balungwe, P. B.; Chimanuka, D.; Mubenga, L. M. | 2021 | | Alobar holoprosencephaly in mining-related areas of the Eastern region of the Democratic Republic of the Congo: A case series | | unrelated topic |
| 1. Rwenyonyi, C.; Bjorvatn, K.; Birkeland, J.; Haugejorden, O. | 1999 | | Altitude as a risk indicator of dental fluorosis in children residing in areas with 0.5 and 2.5 mg fluoride per litre in drinking water | | Year of publication |
| 1. Money, J. | 2002 | | Amative orientation: the hormonal hypothesis examined | | unrelated topic |
| 1. Morar, N.; Seedat, Y. K.; Naidoo, D. P.; Desai, D. K. | 1998 | | Ambulatory blood pressure and risk factors for coronary heart disease in black and Indian medical students | | Duplicate |
| 1. Morar, N.; Seedat, Y. K.; Naidoo, D. P.; Desai, D. K. | 1998 | | Ambulatory blood pressure and risk factors for coronary heart disease in black and Indian medical students | | Year of publication |
| 1. Ghayal, N; Koga, S; Josephs, K; Ahlskog, J; ... | 2019 | | American Association of Neuropathologists, Inc | | unrelated topic |
| 1. Sable, C.; Li, J. S.; Tristani-Firouzi, M.; Fagerlin, A.; Silver, R. M.; Yandel, M.; Yost, H. J.; Beaton, A.; Dale, J.; Engel, M.; Watkins, D.; Spurney, C.; Skinner, A. C.; Armstrong, S. C.; Shah, S. H.; Allen, N.; Davis, M.; Hou, L.; Van Horn, L.; Labarthe, D.; Lloyd-Jones, D.; Marino, B. | 2023 | | American Heart Association's Children's Strategically Focused Research Network Experience | | bookSection |
|  | 1999 | | Amniocentesis–too dangerous and too late? | | Year of publication |
| 1. Ihse, E.; Ybo, A.; Suhr, O.; Lindqvist, P.; Backman, C.; Westermark, P. | 2008 | | Amyloid fibril composition is related to the phenotype of hereditary transthyretin V30M amyloidosis | | Duplicate |
| 1. Ihse, E.; Ybo, A.; Suhr, O.; Lindqvist, P.; Backman, C.; Westermark, P. | 2008 | | Amyloid fibril composition is related to the phenotype of hereditary transthyretin V30M amyloidosis | | unrelated topic |
| 1. Sykes, L. M.; Essop, A. R.; Sukha, A. K. | 2001 | | An 8-year assessment of maxillofacial prosthetic patients treated in a Department of Prosthetic Dentistry | | unrelated topic |
| 1. Srinivasan, S.; Gunasekaran, S.; Mathivanan, S. K.; M, B. Bam; Jayagopal, P.; Dalu, G. T. | 2023 | | An active learning machine technique based prediction of cardiovascular heart disease from UCI-repository database | | unrelated topic |
| 1. Siyotula, T.; Arnold, M. | 2022 | | An analysis of neonatal mortality following gastro-intestinal and/or abdominal surgery in a tertiary hospital in South Africa | | Duplicate |
| 1. Siyotula, T.; Arnold, M. | 2022 | | An analysis of neonatal mortality following gastro-intestinal and/or abdominal surgery in a tertiary hospital in South Africa | | unrelated topic |
| 1. Manyama, M.; Rolian, C.; Gilyoma, J.; Magori, C. C.; Mjema, K.; Mazyala, E.; Kimwaga, E.; Hallgrimsson, B. | 2011 | | An assessment of orofacial clefts in Tanzania | | unrelated topic |
| 1. Steyn, M.; Nienaber, W. C.; Meiring, J. H. | 2002 | | An assessment of the health status and physical characteristics of an early 20th century community a + Maroelabult in the north west province, South Africa | | unrelated topic |
| 1. Lipschitz, R.; Beck, J. M.; Froman, C. | 1969 | | An assessment of the treatment of encephalomeningoceles | | Year of publication |
| 1. Awadia, A. K.; Birkeland, J. M.; Haugejorden, O.; Bjorvatn, K. | 2000 | | An attempt to explain why Tanzanian children drinking water containing 0.2 or 3.6 mg fluoride per liter exhibit a similar level of dental fluorosis | | unrelated topic |
| 1. Ezeh, G. O.; Oniyangi, O.; Nwatah, V. E.; Oyinwola, O. I.; Ekaidem, I. B.; Okonkwo, F. O.; Aikhionbare, H. A. | 2022 | | An audit of a decade of acute peritoneal dialysis in children with acute kidney injury: A single-center experience | | unrelated topic |
| 1. Ugochukwu, O.; Jerome, A. | 2010 | | An audit of intensive care unit admission in a pediatric cardio-thoracic population in Enugu, Nigeria | | unrelated topic |
| 1. Mazibuko, B.; Ramnarain, H.; Moodley, J. | 2012 | | An audit of pregnant women with prosthetic heart valves at a tertiary hospital in South Africa: a five-year experience | | unrelated topic |
| 1. Onyeaso, C. O. | 2003 | | An epidemiological survey of occlusal anomalies among secondary school children in Ibadan, Nigeria | | unrelated topic |
| 1. Fieger, A.; Marck, K. W.; Busch, R.; Schmidt, A. | 2003 | | An estimation of the incidence of noma in north-west Nigeria | | unrelated topic |
| 1. Adeyemo, W. L.; James, O.; Adeyemi, M. O.; Ogunlewe, M. O.; Ladeinde, A. L.; Butali, A.; Taiwo, O. A.; Emeka, C. I.; Ayodele, A. O.; Ugwumba, C. U. | 2013 | | An evaluation of surgical outcome of bilateral cleft lip surgery using a modified Millard's (Fork Flap) technique | | unrelated topic |
| 1. Mytton, J.; Harrison, V.; McLoughlin, A.; Thompson, R.; Overton, T. | 2008 | | An evaluation of the recording of folic acid use in the South West Congenital Anomaly Register | | unrelated topic |
| 1. Ghiorghis, B. | 1991 | | An infant with three legs–a case report and review of the literature | | Year of publication |
| 1. Pareyn, M.; Dvorak, V.; Halada, P.; Van Houtte, N.; Girma, N.; de Kesel, W.; Merdekios, B.; Massebo, F.; Leirs, H.; Volf, P. | 2020 | | An integrative approach to identify sand fly vectors of leishmaniasis in Ethiopia by morphological and molecular techniques | | unrelated topic |
| 1. Pentsuk, N.; van der Laan, J. W. | 2009 | | An interspecies comparison of placental antibody transfer: new insights into developmental toxicity testing of monoclonal antibodies | | unrelated topic |
| 1. Matsumoto, T.; Itoh, N.; Inoue, S.; Nakamura, M. | 2016 | | An observation of a severely disabled infant chimpanzee in the wild and her interactions with her mother | | unrelated topic |
| 1. Adesina, K. T.; Ogunlaja, O. A.; Olarinoye, A. O.; Aboyeji, A. P.; Akande, H. J.; Fawole, A. A.; Adeniran, A. S. | 2018 | | An observation of umbilical coiling index in a low risk population in Nigeria | | unrelated topic |
| 1. Swarts, L.; Leisegang, F.; Owen, E. P.; Henderson, H. E. | 2007 | | An OTC deficiency 'phenocopy' in association with Klinefelter syndrome | | unrelated topic |
| 1. Watkins, J. J. | 1975 | | An unusual eruption sequestrum. A case report | | Year of publication |
| 1. Mba, S. E.; Musara, A.; Kalangu, K.; Nyamapfene, B. | 2019 | | An unusual presentation of bobble-head doll syndrome in a patient with hydranencephaly and Chiari 3 malformation | | unrelated topic |
| 1. Abushouk, A. I.; Negida, A.; Ahmed, H. | 2016 | | An updated review of Zika virus | | unrelated topic |
| 1. Bereket, C.; Çakır-Özkan, N.; Şener, İ; Bulut, E.; Baştan, Aİ | 2015 | | Analyses of 1100 supernumerary teeth in a nonsyndromic Turkish population: A retrospective multicenter study | | unrelated topic |
| 1. Weatherley-White, R. C.; Ben, S.; Jin, Y.; Riccardi, S.; Arnold, T. D.; Spritz, R. A. | 2011 | | Analysis of genomewide association signals for nonsyndromic cleft lip/palate in a Kenya African Cohort | | unrelated topic |
| 1. Tchatchouang, S.; Nzouankeu, A.; Hong, E.; Terrade, A.; Denizon, M.; Deghmane, A. E.; Ndiang, S. M. T.; Pefura-Yone, E. W.; Penlap Beng, V.; Njouom, R.; Fonkoua, M. C.; Taha, M. K. | 2020 | | Analysis of Haemophilus species in patients with respiratory tract infections in Yaoundé, Cameroon | | unrelated topic |
| 1. Nasir, A. A.; Abdur-Rahman, L. O.; Adesiyun, O. O.; Bamigbola, K. T.; Adegboye, M. B.; Raji, H. O.; Adesiyun, O. A. M.; Adeniran, J. O. | 2019 | | Analysis of Presentations and Outcomes of Care of Children with Disorders of Sexual Development in a Nigerian Hospital | | unrelated topic |
| 1. Anttila, V.; Bulik-Sullivan, B.; Finucane, H. K.; Walters, R. K.; Bras, J.; Duncan, L.; Escott-Price, V.; Falcone, G. J.; Gormley, P.; Malik, R.; Patsopoulos, N. A.; Ripke, S.; Wei, Z.; Yu, D.; Lee, P. H.; Turley, P.; Grenier-Boley, B.; Chouraki, V.; Kamatani, Y.; Berr, C.; Letenneur, L.; Hannequin, D.; Amouyel, P.; Boland, A.; Deleuze, J. F.; Duron, E.; Vardarajan, B. N.; Reitz, C.; Goate, A. M.; Huentelman, M. J.; Kamboh, M. I.; Larson, E. B.; Rogaeva, E.; St George-Hyslop, P.; Hakonarson, H.; Kukull, W. A.; Farrer, L. A.; Barnes, L. L.; Beach, T. G.; Demirci, F. Y.; Head, E.; Hulette, C. M.; Jicha, G. A.; Kauwe, J. S. K.; Kaye, J. A.; Leverenz, J. B.; Levey, A. I.; Lieberman, A. P.; Pankratz, V. S.; Poon, W. W.; Quinn, J. F.; Saykin, A. J.; Schneider, L. S.; Smith, A. G.; Sonnen, J. A.; Stern, R. A.; Van Deerlin, V. M.; Van Eldik, L. J.; Harold, D.; Russo, G.; Rubinsztein, D. C.; Bayer, A.; Tsolaki, M.; Proitsi, P.; Fox, N. C.; Hampel, H.; Owen, M. J.; Mead, S.; Passmore, P.; Morgan, K.; Nöthen, M. M.; Rossor, M.; Lupton, M. K.; Hoffmann, P.; Kornhuber, J.; Lawlor, B.; McQuillin, A.; Al-Chalabi, A.; Bis, J. C.; Ruiz, A.; Boada, M.; Seshadri, S.; Beiser, A.; Rice, K.; van der Lee, S. J.; De Jager, P. L.; Geschwind, D. H.; Riemenschneider, M.; Riedel-Heller, S.; Rotter, J. I.; Ransmayr, G.; Hyman, B. T.; Cruchaga, C.; Alegret, M.; Winsvold, B.; Palta, P.; Farh, K. H.; Cuenca-Leon, E.; Furlotte, N.; Kurth, T.; others | 2018 | | Analysis of shared heritability in common disorders of the brain | | unrelated topic |
| 1. Grant, I. R.; Freercks, R. J.; Honiball, E. J.; Dube, B. | 2021 | | Analysis of the vascular access service for patients on haemodialysis in Livingstone Hospital | | unrelated topic |
| 1. Omodan, A.; Pillay, P.; Lazarus, L.; Gounden, K.; Madaree, A.; Satyapal, K. | 2020 | | Anatomical Classification of Tessier Craniofacial Clefts Numbers 3 and 4 | | unrelated topic |
| 1. Bekele, K. K.; Ekanem, P. E.; Meberate, B. | 2019 | | Anatomical patterns of cleft lip and palate deformities among neonates in Mekelle, Tigray, Ethiopia; implication of environmental impact | | unrelated topic |
| 1. Silva, R Da | 2013 | | Anatomical study of the variation in the branching patterns and histology of the aorta in a South African population | | book |
| 1. Saidi, H. S.; Olumbe, A. O.; Kalebi, A. | 2002 | | Anatomy and pathology of coronary artery in adult black Kenyans | | unrelated topic |
| 1. Cáceres, A.; Esko, T.; Pappa, I.; Gutiérrez, A.; Lopez-Espinosa, M. J.; Llop, S.; Bustamante, M.; Tiemeier, H.; Metspalu, A.; Joshi, P. K.; Wilsonx, J. F.; Reina-Castillón, J.; Shin, J.; Pausova, Z.; Paus, T.; Sunyer, J.; Pérez-Jurado, L. A.; González, J. R. | 2016 | | Ancient Haplotypes at the 15q24.2 Microdeletion Region Are Linked to Brain Expression of MAN2C1 and Children's Intelligence | | unrelated topic |
| 1. Harrison, JL; Prendergast, BD | 2016 | | and William A. Littler | | book |
| 1. Stell, S. K.; Moller, P. | 2017 | | Androgen-induced pseudo-hermaphroditic phenotypes in female Brevimyrus niger Günther 1866 (Teleostei, Mormyridae) | | unrelated topic |
| 1. Odhiambo, C.; Zeh, C.; Ondoa, P.; Omolo, P.; Akoth, B.; Lwamba, H.; Lando, R.; Williamson, J.; Otieno, J.; Masaba, R.; Weidle, P.; Thomas, T. | 2015 | | Anemia and Red Blood Cell Abnormalities in HIV-Infected and HIV-Exposed Breastfed Infants: A Secondary Analysis of the Kisumu Breastfeeding Study | | unrelated topic |
| 1. Adenekan, A. T.; Faponle, A. F.; Oginni, F. O. | 2011 | | Anesthetic challenges in oro-facial cleft repair in Ile-Ife, Nigeria | | unrelated topic |
| 1. Moscarella, E.; Mangieri, A.; Giannini, F.; Tchetchè, D.; Kim, W. K.; Sinning, J. M.; Landes, U.; Kornowski, R.; De Backer, O.; Nickenig, G.; De Biase, C.; Søndergaard, L.; De Marco, F.; Bedogni, F.; Ancona, M.; Montorfano, M.; Regazzoli, D.; Stefanini, G.; Toggweiler, S.; Tamburino, C.; Immè, S.; Tarantini, G.; Sievert, H.; Schäfer, U.; Kempfert, J.; Wöehrle, J.; Latib, A.; Calabrò, P.; Medda, M.; Tespili, M.; Colombo, A.; Ielasi, A. | 2022 | | Annular size and interaction with trans-catheter aortic valves for treatment of severe bicuspid aortic valve stenosis: Insights from the BEAT registry | | unrelated topic |
| 1. Kalangu, K. K.; Wolf, B. | 1994 | | Ano-cutaneous fistula associated with Bardet-Biedl syndrome in an African child | | Year of publication |
| 1. Manuel, V.; Sousa-Uva, M.; Morais, H.; Magalhães, M. P.; Pedro, A.; Miguel, G.; Nunes, M. A.; Gamboa, S.; Júnior, A. P. | 2015 | | Anomalous Origin of One Pulmonary Artery From the Ascending Aorta: From Diagnosis to Treatment in Angola | | unrelated topic |
| 1. Pepeta, L.; Takawira, F. F.; Cilliers, A. M.; Adams, P. E.; Ntsinjana, N. H.; Mitchell, B. J. | 2011 | | Anomalous origin of the left pulmonary artery from the ascending aorta in two children with pulmonary atresia, sub-aortic ventricular septal defect and right-sided major aorto-pulmonary collateral arteries | | unrelated topic |
| 1. Dormitzer, P. R.; Ellison, P. T.; Bode, H. H. | 1989 | | Anomalously low endemic goiter prevalence among Efe pygmies | | Year of publication |
| 1. Uba, A. F.; Chirdan, L. B.; Ardill, W.; Edino, S. T. | 2006 | | Anorectal anomaly: a review of 82 cases seen at JUTH, Nigeria | | unrelated topic |
| 1. Moore, S. W.; Tshifularo, N.; Banieghbal, B.; Le Grange, E.; Millar, A.; Lakhoo, K. | 2013 | | Anorectal atresia with gross terminal colonic distension in Africa | | unrelated topic |
| 1. Lukong, C. S.; Mshelbwala, P. M.; Anumah, M. A.; Ameh, E. A.; Nmadu, P. T. | 2012 | | Anorectal malformation coexisting with Hirschsprung's disease: a report of two patients | | unrelated topic |
| 1. Patankar, J. Z.; Mali, V. P.; Yashpal, R.; Neo, G. T.; Prabhakaran, K. | 2004 | | Anorectal malformation with congenital absence of vagina: a case report and review of the literature | | unrelated topic |
| 1. Sharma, S.; Mazingi, D.; Imam, S.; Chowdhury, T. K.; Saldaña, L. J.; Mashavave, N. Z.; Olivos, M.; Chowdhury, T. S.; Hoque, M.; Correa, C.; Banu, T. | 2023 | | Anorectal malformations in low and middle-income countries- spectrum, burden and management | | unrelated topic |
| 1. Vd Merwe, E.; Cox, S.; Numanoglu, A. | 2017 | | Anorectal malformations, associated congenital anomalies and their investigation in a South African setting | | unrelated topic |
| 1. Kamla, I.; Kamgaing, N.; Billong, S.; Tochie, J. N.; Tolefac, P.; de Paul Djientcheu, V. | 2019 | | Antenatal and postnatal diagnoses of visible congenital malformations in a sub-Saharan African setting: a prospective multicenter cohort study | | unrelated topic |
| 1. Omo-Aghoja, V. W.; Omo-Aghoja, L. O.; Ugboko, V. I.; Obuekwe, O. N.; Saheeb, B. D.; Feyi-Waboso, P.; Onowhakpor, A. | 2010 | | Antenatal determinants of oro-facial clefts in Southern Nigeria | | unrelated topic |
| 1. Kalangu, K.; Levy, L. F.; Makarawo, S.; Nkrumah, F. K. | 1990 | | Anterior encephalocoele–our experience in Harare, Zimbabwe, after the introduction of cat scanning | | Year of publication |
| 1. Hassanali, J.; Pokhariyal, G. P. | 1993 | | Anterior tooth relations in Kenyan Africans | | Year of publication |
| 1. Oredugba, F. A.; Savage, K. O. | 2002 | | Anthropometric finding in Nigerian children with sickle cell disease | | unrelated topic |
| 1. Wu, L.; Shryock, J. C.; Song, Y.; Li, Y.; Antzelevitch, C.; Belardinelli, L. | 2004 | | Antiarrhythmic effects of ranolazine in a guinea pig in vitro model of long-QT syndrome | | unrelated topic |
| 1. Lerbech, A. M.; Opintan, J. A.; Bekoe, S. O.; Ahiabu, M. A.; Tersbøl, B. P.; Hansen, M.; Brightson, K. T.; Ametepeh, S.; Frimodt-Møller, N.; Styrishave, B. | 2014 | | Antibiotic exposure in a low-income country: screening urine samples for presence of antibiotics and antibiotic resistance in coagulase negative staphylococcal contaminants | | unrelated topic |
| 1. Momoh, A. H.; Kwaga, J. K. P.; Bello, M.; Sackey, A. K. B.; Larsen, A. R. | 2018 | | Antibiotic resistance and molecular characteristics of Staphylococcus aureus isolated from backyard-raised pigs and pig workers | | unrelated topic |
| 1. Carvalho, M. J.; Sands, K.; Thomson, K.; Portal, E.; Mathias, J.; Milton, R.; Gillespie, D.; Dyer, C.; Akpulu, C.; Boostrom, I.; Hogan, P.; Saif, H.; Ferreira, A.; Nieto, M.; Hender, T.; Hood, K.; Andrews, R.; Watkins, W. J.; Hassan, B.; Chan, G.; Bekele, D.; Solomon, S.; Metaferia, G.; Basu, S.; Naha, S.; Sinha, A.; Chakravorty, P.; Mukherjee, S.; Iregbu, K.; Modibbo, F.; Uwaezuoke, S.; Audu, L.; Edwin, C. P.; Yusuf, A. H.; Adeleye, A.; Mukkadas, A. S.; Zahra, R.; Shirazi, H.; Muhammad, A.; Ullah, S. N.; Jan, M. H.; Akif, S.; Mazarati, J. B.; Rucogoza, A.; Gaju, L.; Mehtar, S.; Bulabula, A. N. H.; Whitelaw, A.; Roberts, L.; Walsh, T. R. | 2022 | | Antibiotic resistance genes in the gut microbiota of mothers and linked neonates with or without sepsis from low- and middle-income countries | | unrelated topic |
| 1. Mensah, K. B.; Opoku-Agyeman, K.; Ansah, C. | 2017 | | Antibiotic use during pregnancy: a retrospective study of prescription patterns and birth outcomes at an antenatal clinic in rural Ghana | | unrelated topic |
| 1. Rahman, A. E.; Hossain, A. T.; Zaman, S. B.; Salim, N.; K, C. A.; Day, L. T.; Ameen, S.; Ruysen, H.; Kija, E.; Peven, K.; Tahsina, T.; Ahmed, A.; Rahman, Q. S.; Khan, J.; Kong, S.; Campbell, H.; Hailegebriel, T. D.; Ram, P. K.; Qazi, S. A.; El Arifeen, S.; Lawn, J. E. | 2021 | | Antibiotic use for inpatient newborn care with suspected infection: EN-BIRTH multi-country validation study | | unrelated topic |
| 1. Molini, U.; Capobianco Dondona, A.; Hilbert, R.; Monaco, F. | 2018 | | Antibodies against Schmallenberg virus detected in cattle in the Otjozondjupa region, Namibia | | unrelated topic |
| 1. Caton, AR; Bell, EM; Druschel, CM; Werler, MM; ... | 2009 | | Antihypertensive medication use during pregnancy and the risk of cardiovascular malformations | | unrelated topic |
| 1. Afeke, I.; Amegan-Aho, K. H.; Adu-Amankwaah, J.; Orish, V. N.; Mensah, G. L.; Mbroh, H. K.; Jamfaru, I.; Hamid, A. M.; Ankrah, L. M.; Korbuvi, J.; Ablordey, A. | 2023 | | Antimicrobial profile of coagulase-negative staphylococcus isolates from categories of individuals at a neonatal intensive care unit of a tertiary hospital, Ghana | | unrelated topic |
| 1. Ibrahim, R. A.; Berhe, N.; Mekuria, Z.; Seyoum, E. T.; Balada-Llasat, J. M.; Abebe, T.; Mariam, S. H.; Tsige, E.; Fentaw Dinku, S.; Wang, S. H. | 2023 | | Antimicrobial Resistance and Virulence Gene Profile of Clinical Staphylococcus aureus: A Multi-Center Study from Ethiopia | | unrelated topic |
| 1. El Zowalaty, M. E.; Lamichhane, B.; Falgenhauer, L.; Mowlaboccus, S.; Zishiri, O. T.; Forsythe, S.; Helmy, Y. A. | 2023 | | Antimicrobial resistance and whole genome sequencing of novel sequence types of Enterococcus faecalis, Enterococcus faecium, and Enterococcus durans isolated from livestock | | unrelated topic |
| 1. Yehouenou, C. L.; Kpangon, A. A.; Affolabi, D.; Rodriguez-Villalobos, H.; Van Bambeke, F.; Dalleur, O.; Simon, A. | 2020 | | Antimicrobial resistance in hospitalized surgical patients: a silently emerging public health concern in Benin | | unrelated topic |
| 1. Gomba, A.; Chidamba, L.; Korsten, L. | 2016 | | Antimicrobial Resistance Profiles of Salmonella spp. from Agricultural Environments in Fruit Production Systems | | unrelated topic |
| 1. Laumen, J. G. E.; Van Dijck, C.; Abdellati, S.; De Baetselier, I.; Serrano, G.; Manoharan-Basil, S. S.; Bottieau, E.; Martiny, D.; Kenyon, C. | 2022 | | Antimicrobial susceptibility of commensal Neisseria in a general population and men who have sex with men in Belgium | | unrelated topic |
| 1. Elufisan, T. O.; Luna, I. C. R.; Oyedara, O. O.; Varela, A. S.; García, V. B.; Oluyide, B. O.; Treviño, S. F.; López, M. A. V.; Guo, X. | 2020 | | Antimicrobial susceptibility pattern of Stenotrophomonas species isolated from Mexico | | unrelated topic |
| 1. Masukume, G. | 2014 | | Antimongoloid as a pejorative term | | bookSection |
| 1. Alvarez, M.; Malécot, C. O.; Gannier, F.; Lignon, J. M. | 2005 | | Antimony-induced cardiomyopathy in guinea-pig and protection by L-carnitine | | unrelated topic |
| 1. Ramiharimanana, F. D.; Haddad, J. G.; Andrianavalonirina, M. A.; Apel, C.; Olivon, F.; Diotel, N.; Desprès, P.; Ramanandraibe, V. V.; El Kalamouni, C. | 2022 | | Antiviral Effect of Stenocline ericoides DC. and Stenocline inuloides DC., Two Flavonoid-Rich Endemic Plants from Madagascar, against Dengue and Zika Viruses | | unrelated topic |
| 1. Bamigboye-Taiwo, O. T.; Okeniyi, J. A.; Onakpoya, U. U.; Ojo, O. O.; Eyekpegha, J. O.; Oguns, A.; Adeyefa, B. | 2020 | | Aortic Valve Regurgitation following Transthoracic Open Surgical Ligation of Patent Ductus Arteriosus | | unrelated topic |
| 1. Loktionov, A.; Vorster, H.; O'Neill, I. K.; Nell, T.; Bingham, S. A.; Runswick, S. A.; Cummings, J. H. | 1999 | | Apolipoprotein E and methylenetetrahydrofolate reductase genetic polymorphisms in relation to other risk factors for cardiovascular disease in UK Caucasians and Black South Africans | | Year of publication |
| 1. Adeniyi, A. E.; Akisanya, C. O.; Ogah, O. S.; Akinremi, T. O.; Erinle, C. A. | 2008 | | Appendicitis and situs inversus viscerum in a 32-year-old female nigerian: a case report | | bookSection |
| 1. Aluko, O. O.; Sridhar, M. K. | 2005 | | Application of constructed wetlands to the treatment of leachates from a municipal solid waste landfill in Ibadan, Nigeria | | unrelated topic |
| 1. Cappuccio, F. P.; Oakeshott, P.; Strazzullo, P.; Kerry, S. M. | 2002 | | Application of Framingham risk estimates to ethnic minorities in United Kingdom and implications for primary prevention of heart disease in general practice: cross sectional population based study | | outside geographic scope |
| 1. Jin, L.; Thomas, B. | 2007 | | Application of molecular and serological assays to case based investigations of rubella and congenital rubella syndrome | | unrelated topic |
| 1. Idini, A.; Frau, F.; Gutierrez, L.; Dore, E.; Nocella, G.; Ghiglieri, G. | 2020 | | Application of Octacalcium Phosphate with an Innovative Household-scale Defluoridator Prototype and Behavioral Determinants of its Adoption in Rural Communities of the East African Rift Valley | | unrelated topic |
| 1. Ugboko, V. I.; Amole, A. O.; Ibitoye, B. | 2003 | | Applications of ultrasonography in oral and maxillofacial surgery: a review of the literature | | unrelated topic |
| 1. Claude, K. M.; Juvenal, K. L.; Hawkes, M. | 2012 | | Applying a knowledge-to-action framework for primary prevention of spina bifida in tropical Africa | | unrelated topic |
| 1. Pugh, R. N.; Hossain, M. M.; Malik, M.; El Mugamer, I. T.; White, M. A. | 1998 | | Arabian Peninsula men tend to insulin resistance and cardiovascular risk seen in South Asians | | Year of publication |
| 1. Igbigbi, P. S.; Msamati, B. C.; Shariff, M. B. | 2005 | | Arch index as a predictor of pes planus: a comparative study of indigenous Kenyans and Tanzanians | | unrelated topic |
| 1. Goossens, E.; van Deyk, K.; Budts, W.; Moons, P. | 2022 | | Are missed appointments in an outpatient clinic for adults with congenital heart disease the harbinger for care gaps? | | unrelated topic |
| 1. Okoye, J. O.; Ngokere, A. A. | 2020 | | Are the prevalence of Trisomy 13 and the incidence of severe holoprosencephaly increasing in Africa? | | unrelated topic |
| 1. Idon, P. I.; Ikusika, O. F.; Sotunde, O. A.; Ogundare, T. O. | 2022 | | Are there associations between the occurrence of dental fluorosis and the experience of dentine hypersensitivity? A cross-sectional study | | unrelated topic |
| 1. Sinzobahamvya, N. | 2014 | | Aristotle Comprehensive Complexity score for closure of patent ductus arteriosus | | unrelated topic |
| 1. Dick, I. E.; Joshi-Mukherjee, R.; Yang, W.; Yue, D. T. | 2016 | | Arrhythmogenesis in Timothy Syndrome is associated with defects in Ca(2+)-dependent inactivation | | unrelated topic |
| 1. Adam, I.; Elwasila, E.; Mohammed Ali, D. A.; Elansari, E.; Elbashir, M. I. | 2004 | | Artemether in the treatment of falciparum malaria during pregnancy in eastern Sudan | | unrelated topic |
| 1. Shaheen, S.; Bahar, M. E. H.; Mohammed, A. H. A.; Elbadri, S. F. A.; Johari, A. | 2018 | | Arterial tree anomalies in patients with clubfoot: an investigation carried out at Soba University Hospital | | unrelated topic |
| 1. Adam, I.; Ali, D. M.; Abdalla, M. A. | 2006 | | Artesunate plus sulfadoxine-pyrimethamine in the treatment of uncomplicated Plasmodium falciparum malaria during pregnancy in eastern Sudan | | unrelated topic |
| 1. Steyn, D. G. | 1969 | | Artificial fluoridation of public water supplies | | Year of publication |
| 1. Uner, D. D.; Izol, B. S. | 2019 | | Ascher syndrome: A case report | | bookSection |
| 1. Mushi, M. F.; Buname, G.; Bader, O.; Groß, U.; Mshana, S. E. | 2016 | | Aspergillus fumigatus carrying TR34/L98H resistance allele causing complicated suppurative otitis media in Tanzania: Call for improved diagnosis of fungi in sub-Saharan Africa | | unrelated topic |
| 1. Kinoti, S. N. | 1993 | | Asphyxia of the newborn in east, central and southern Africa | | Year of publication |
| 1. Bruneel, L.; Alighieri, C.; Bettens, K.; Musasizi, D.; Ojok, I.; D'Haeseleer, E.; Van Lierde, K. | 2019 | | Assessing health-related quality of life in patients with cleft palate in resource-limited countries: A preliminary evaluation of the VELO questionnaire in Uganda | | unrelated topic |
| 1. Balkus, J. E.; Neradilek, M.; Fairlie, L.; Makanani, B.; Mgodi, N.; Mhlanga, F.; Nakabiito, C.; Mayo, A.; Harrell, T.; Piper, J.; Bunge, K. E. | 2021 | | Assessing pregnancy and neonatal outcomes in Malawi, South Africa, Uganda, and Zimbabwe: Results from a systematic chart review | | unrelated topic |
| 1. Freene, N.; Del Pozo Cruz, B.; Davey, R. | 2016 | | Assessing the 'active couch potato' phenomenon in cardiac rehabilitation: rationale and study protocol | | unrelated topic |
| 1. Eshete, M.; Abate, F.; Abera, B.; Hailu, A.; Demissie, Y.; Mossey, P.; Butali, A. | 2021 | | Assessing the Practice of Birth Defect Registration at Addis Ababa Health Facilities | | unrelated topic |
| 1. Githuku, J. N.; Azofeifa, A.; Valencia, D.; Ao, T.; Hamner, H.; Amwayi, S.; Gura, Z.; Omolo, J.; Albright, L.; Guo, J.; Arvelo, W. | 2014 | | Assessing the prevalence of spina bifida and encephalocele in a Kenyan hospital from 2005-2010: implications for a neural tube defects surveillance system | | unrelated topic |
| 1. Awodele, O.; Popoola, D.; Odunsi, P.; Akinde, R.; Akintonwa, A. | 2013 | | Assessing the risk of birth defects associated with exposure to highly active anti-retroviral therapy during organogenesis in rats | | unrelated topic |
| 1. Schomaker, M.; Davies, M. A.; Cornell, M.; Ford, N. | 2018 | | Assessing the risk of dolutegravir for women of childbearing potential | | unrelated topic |
| 1. Mehta, U.; Heekes, A.; Kalk, E.; Boulle, A. | 2018 | | Assessing the value of Western Cape Provincial Government health administrative data and electronic pharmacy records in ascertaining medicine use during pregnancy | | unrelated topic |
| 1. Boye, A.; Boampong, V. A.; Takyi, N.; Martey, O. | 2016 | | Assessment of an aqueous seed extract of Parkia clappertoniana on reproductive performance and toxicity in rodents | | unrelated topic |
|  | 1993 | | Assessment of mortality levels, trends and differentials in relation to the goal of "Health for All" by year 2000 in some ECA member states | | Year of publication |
| 1. Chinawa, A. T.; Chinawa, J. M.; Duru, C. O.; Chukwu, B. F.; Obumneme-Anyim, I. | 2021 | | Assessment of Nutritional Status of Children With Congenital Heart Disease: A Comparative Study | | unrelated topic |
| 1. Wiehahn, G. J.; Bosch, G. P.; du Preez, R. R.; Pretorius, H. W.; Karayiorgou, M.; Roos, J. L. | 2004 | | Assessment of the frequency of the 22q11 deletion in Afrikaner schizophrenic patients | | unrelated topic |
| 1. Adesina, B. A.; Otuyemi, O. D.; Kolawole, K. A.; Adeyemi, A. T. | 2013 | | Assessment of the impact of tongue size in patients with bimaxillary protrusion | | unrelated topic |
| 1. Gohil, H. R.; Jumbi, T. M.; Kuria, D. K.; Osawa, F. | 2023 | | Assessment of the neonatal referral and transport system for patients with gastroschisis in Kenya | | unrelated topic |
| 1. Mulugeta, B.; Seyoum, G.; Mekonnen, A.; Ketema, E. | 2022 | | Assessment of the prevalence and associated risk factors of pediatric hydrocephalus in diagnostic centers in Addis Ababa, Ethiopia | | unrelated topic |
| 1. Rwenyonyi, C. M.; Birkeland, J. M.; Haugejorden, O. | 2000 | | Assessment of the validity and consequences of different methods of expressing the severity of dental fluorosis in a subject | | unrelated topic |
| 1. Ademiluyi, S. A.; Oyeneyin, J. O.; Sowemimo, G. O. | 1989 | | Associated congenital abnormalities in Nigeria children with cleft lip and palate | | Year of publication |
| 1. Folayan, M. O.; Alade, M.; Adeniyi, A.; El Tantawi, M.; Finlayson, T. L. | 2019 | | Association between developmental dental anomalies, early childhood caries and oral hygiene status of 3-5-year-old children in Ile-Ife, Nigeria | | unrelated topic |
| 1. Tingleff, T.; Räisänen, S.; Vikanes, Å; Sandvik, L.; Laine, K. | 2021 | | Association between maternal country of birth and preterm birth: A population-based register study of 910,752 deliveries | | unrelated topic |
| 1. Okonkwo, R. I.; Onyeabochukwu, A. D.; Izuka, E. O.; Duke-Onyeabo, C. P.; Obiora-Izuka, C. E.; Ejelonu, U. T.; Nwagha, U. I. | 2023 | | Association between maternal selenium levels and pregnancy outcome among human immunodeficiency virus-positive and human immunodeficiency virus-negative pregnant women in a tertiary health-care center in Owerri, Nigeria: A comparative cross-sectional study | | unrelated topic |
| 1. Slone, M.; Durrheim, K.; Lachman, P.; Kaminer, D. | 1998 | | Association between the diagnosis of mental retardation and socioeconomic factors | | Duplicate |
| 1. Slone, M.; Durrheim, K.; Lachman, P.; Kaminer, D. | 1998 | | Association between the diagnosis of mental retardation and socioeconomic factors | | Year of publication |
| 1. Fang, J.; Yuan, K.; Gindi, R. M.; Ward, B. W.; Ayala, C.; Loustalot, F. | 2018 | | Association of Birthplace and Coronary Heart Disease and Stroke Among US Adults: National Health Interview Survey, 2006 to 2014 | | unrelated topic |
| 1. Mulualem, D.; Hailu, D.; Tessema, M.; Whiting, S. J. | 2022 | | Association of Dietary Calcium Intake with Dental, Skeletal and Non-Skeletal Fluorosis among Women in the Ethiopian Rift Valley | | unrelated topic |
| 1. Abdel Hamid, M. M.; Ahmed, S.; Salah, A.; Tyrab, E. M.; Yahia, L. M.; Elbashir, E. A.; Musa, H. H. | 2015 | | Association of lipoprotein lipase gene with coronary heart disease in Sudanese population | | Duplicate |
| 1. Abdel Hamid, M. M.; Ahmed, S.; Salah, A.; Tyrab, E. M.; Yahia, L. M.; Elbashir, E. A.; Musa, H. H. | 2015 | | Association of lipoprotein lipase gene with coronary heart disease in Sudanese population | | unrelated topic |
| 1. Gowans, L. J.; Adeyemo, W. L.; Eshete, M.; Mossey, P. A.; Busch, T.; Aregbesola, B.; Donkor, P.; Arthur, F. K.; Bello, S. A.; Martinez, A.; Li, M.; Augustine-Akpan, E. A.; Deressa, W.; Twumasi, P.; Olutayo, J.; Deribew, M.; Agbenorku, P.; Oti, A. A.; Braimah, R.; Plange-Rhule, G.; Gesses, M.; Obiri-Yeboah, S.; Oseni, G. O.; Olaitan, P. B.; Abdur-Rahman, L.; Abate, F.; Hailu, T.; Gravem, P.; Ogunlewe, M. O.; Buxó, C. J.; Marazita, M. L.; Adeyemo, A. A.; Murray, J. C.; Butali, A. | 2016 | | Association Studies and Direct DNA Sequencing Implicate Genetic Susceptibility Loci in the Etiology of Nonsyndromic Orofacial Clefts in Sub-Saharan African Populations | | unrelated topic |
| 1. Perumal, N.; Wang, D.; Darling, A. M.; Wang, M.; Liu, E.; Urassa, W.; Pembe, A. B.; Fawzi, W. W. | 2022 | | Associations between Gestational Weight Gain Adequacy and Neonatal Outcomes in Tanzania | | unrelated topic |
| 1. Shonubi, A. M.; Akiode, O.; Salami, B. A.; Musa, A. A.; Sotimehin, S. A.; Sule, G. A. | 2006 | | Asymmetrical tetraphocomelia with radiohumeral synostosis | | unrelated topic |
| 1. Oguntola, S. O.; Hassan, M. O.; Duarte, R.; Vachiat, A.; Manga, P.; Naicker, S. | 2019 | | Atherosclerotic vascular disease is more prevalent among black ESKD patients on long-term CAPD in South Africa | | Duplicate |
| 1. Oguntola, S. O.; Hassan, M. O.; Duarte, R.; Vachiat, A.; Manga, P.; Naicker, S. | 2019 | | Atherosclerotic vascular disease is more prevalent among black ESKD patients on long-term CAPD in South Africa | | unrelated topic |
| 1. Casteigt, B.; Samuel, M.; Laplante, L.; Shohoudi, A.; Apers, S.; Kovacs, A. H.; Luyckx, K.; Thomet, C.; Budts, W.; Enomoto, J.; Sluman, M. A.; Lu, C. W.; Jackson, J. L.; Cook, S. C.; Chidambarathanu, S.; Alday, L.; Eriksen, K.; Dellborg, M.; Berghammer, M.; Johansson, B.; Mackie, A. S.; Menahem, S.; Caruana, M.; Veldtman, G.; Soufi, A.; Fernandes, S. M.; White, K.; Callus, E.; Kutty, S.; Brouillette, J.; Moons, P.; Khairy, P. | 2021 | | Atrial arrhythmias and patient-reported outcomes in adults with congenital heart disease: An international study | | unrelated topic |
| 1. Kirberger, R. M.; Berry, W. L. | 1992 | | Atrial septal defect in a dog: the value of Doppler echocardiography | | Year of publication |
| 1. Adiele, D. K.; Chinawa, J. M.; Arodiwe, I. O.; Gouthami, V.; Murthy, K. S.; Eze, J. C.; Obidike, E. O.; Ujunwa, F. A. | 2014 | | Atrial septal defects: Pattern, clinical profile, surgical techniques and outcome at Innova heart hospital: A 4-year review | | unrelated topic |
| 1. Miana, L. A.; Manuel, V.; Turquetto, A. L.; Issa, H. N.; Guerreiro, G. P.; Caneo, L. F.; Jatene, F. B.; Jatene, M. B. | 2020 | | Atrioventricular Valve Repair in Single Ventricle Physiology: Timing Matters | | unrelated topic |
| 1. Bankole, O.; Taiwo, J.; Nasiru, O. | 2011 | | Attitude and beliefs of traditional birth attendants to prematurely erupted teeth of infants in urban local government areas in Ibadan, Nigeria | | unrelated topic |
| 1. Adeyemi, A. T.; Bankole, O. O. | 2012 | | ATTITUDE OF CLEFT CARE SPECIALISTS IN AFRICA TOWARDS PRESURGICAL ORTHOPAEDICS | | unrelated topic |
| 1. Christianson, A. L.; Kruger, H.; Dini, L. | 1994 | | Atypical acrofacial dysostosis syndrome | | Year of publication |
| 1. Bello, S. A.; Ibikari, A. B.; Oketade, I.; Balogun, S. A. | 2019 | | Atypical Facial Clefts From Northcentral Nigeria, Review of 36 Cases | | Duplicate |
| 1. Bello, S. A.; Ibikari, A. B.; Oketade, I.; Balogun, S. A. | 2019 | | Atypical Facial Clefts From Northcentral Nigeria, Review of 36 Cases | | unrelated topic |
| 1. Ahmed, A. O.; Samaila, E.; Abah, E. R.; Oladigbolu, K. K.; Merali, H.; Abubakar, A. | 2010 | | Audiometric findings in Waardenburg's syndrome amongst the institutionalised deaf/ blind in Kaduna-Nigeria | | Duplicate |
| 1. Ahmed, A. O.; Samaila, E.; Abah, E. R.; Oladigbolu, K. K.; Merali, H.; Abubakar, A. | 2011 | | Audiometric findings in Waardenburg's syndrome amongst the institutionalised deaf/blind in Kaduna-Nigeria | | unrelated topic |
| 1. Bayebaye, CT |  | | Audit of blood product transfusion in paediatric congenital heart surgery on cardiopulmonary bypass | | book |
| 1. Obiora, U. J.; Ekpebe, P. A.; Okoye, C.; David-Idiapho, C. G. | 2020 | | Audit of Childhood Death in a Tertiary Care Center in Niger Delta Region of Nigeria | | Duplicate |
| 1. Obiora, U. J.; Ekpebe, P. A.; Okoye, C.; David-Idiapho, C. G. | 2020 | | Audit of Childhood Death in a Tertiary Care Center in Niger Delta Region of Nigeria | | unrelated topic |
| 1. Bayebaye, C. T.; Muteba, M. K.; Chakane, P. M. | 2018 | | Audit of transfusion of blood products in paediatric congenital heart surgery on cardiopulmonary bypass | | unrelated topic |
| 1. Munjanja, S. P.; Masona, D.; Gwaze, I.; Chipato, T. | 1987 | | Audit of ultrasound scanning: antenatal diagnosis of congenital abnormalities in Harare, Zimbabwe | | Year of publication |
| 1. Swanepoel, D. | 2007 | | Auditory pathology in cri-du-chat (5p-) syndrome: phenotypic evidence for auditory neuropathy | | unrelated topic |
| 1. Blumberg, B. S.; Wills, W.; Millman, I.; London, W. T. | 1973 | | Australia antigen in mosquitoes. Feeding experiments and field studies | | Year of publication |
|  | 1979 | | Australian study links excess heat with birth defects | | Year of publication |
| 1. Adams, S.; Xoagus, E. A.; Lazarus, D.; Lentin, R.; Hudson, D. A. | 2019 | | Autologous Fat Grafting for the Treatment of Mild to Moderate Velopharyngeal Insufficiency | | unrelated topic |
| 1. Porta, A.; Girardengo, G.; Bari, V.; George, Jr., A. L.; Brink, P. A.; Goosen, A.; Crotti, L.; Schwartz, P. J. | 2015 | | Autonomic control of heart rate and QT interval variability influences arrhythmic risk in long QT syndrome type 1 | | unrelated topic |
| 1. Okiro, P.; Wainwright, H.; Spranger, J.; Beighton, P. | 2015 | | Autopsy observations in lethal short-rib polydactyly syndromes | | unrelated topic |
| 1. Wiredu, E. K.; Tettey, Y. | 1998 | | Autopsy studies on still births in Korle Bu Teaching Hospital. II: Causes of death in 93 still births | | Year of publication |
| 1. Dlova, N. C.; Jordaan, F. H.; Sarig, O.; Sprecher, E. | 2014 | | Autosomal dominant inheritance of central centrifugal cicatricial alopecia in black South Africans | | unrelated topic |
| 1. Laleye, A.; Awede, B.; Agboton, B.; Azonbakin, S.; Biaou, O.; Sagbo, G.; Adjagba, M.; Audrezet, M. P.; Ferec, C.; Darboux, R. | 2012 | | Autosomal dominant polycystic kidney disease in University Clinic of Nephrology and Haemodialysis of Cotonou: clinical and genetical findings | | unrelated topic |
| 1. Lombard, E. H.; Kromberg, J. G.; Thomson, P. D.; Milner, L. S.; van Biljon, I.; Jenkins, T. | 1989 | | Autosomal recessive polycystic kidney disease. Evidence for high frequency of the gene in the Afrikaans-speaking population | | Year of publication |
| 1. ombard, E. H.; Kromberg, J. G.; Thomson, P. D.; Milner, L. S.; van Biljon, I.; Jenkins, T. | 1989 | | Autosomal recessive polycystic kidney disease. Evidence for high frequency of the gene in the Afrikaans-speaking population | | Year of publication |
| 1. Létard, P.; Drunat, S.; Vial, Y.; Duerinckx, S.; Ernault, A.; Amram, D.; Arpin, S.; Bertoli, M.; Busa, T.; Ceulemans, B.; Desir, J.; Doco-Fenzy, M.; Elalaoui, S. C.; Devriendt, K.; Faivre, L.; Francannet, C.; Geneviève, D.; Gérard, M.; Gitiaux, C.; Julia, S.; Lebon, S.; Lubala, T.; Mathieu-Dramard, M.; Maurey, H.; Metreau, J.; Nasserereddine, S.; Nizon, M.; Pierquin, G.; Pouvreau, N.; Rivier-Ringenbach, C.; Rossi, M.; Schaefer, E.; Sefiani, A.; Sigaudy, S.; Sznajer, Y.; Tunca, Y.; Guilmin Crepon, S.; Alberti, C.; Elmaleh-Bergès, M.; Benzacken, B.; Wollnick, B.; Woods, C. G.; Rauch, A.; Abramowicz, M.; El Ghouzzi, V.; Gressens, P.; Verloes, A.; Passemard, S. | 2018 | | Autosomal recessive primary microcephaly due to ASPM mutations: An update | | unrelated topic |
| 1. Adeyokunnu, A. A. | 1983 | | Autosomal trisomy 18 and 13 syndromes in Ibadan, Nigeria | | Year of publication |
| 1. Lusambili, A.; Nakstad, B. | 2023 | | Awareness and interventions to reduce dehydration in pregnant, postpartum women, and newborns in rural Kenya | | unrelated topic |
| 1. Adeleye, A. O.; Joel-Medewase, V. I. | 2015 | | Awareness and uptake of measures for preventing CNS birth defects among mothers of affected children in a sub-Saharan African neurosurgeon's practice | | unrelated topic |
| 1. Okon, U. A.; Ibrahim, B. S.; Usman, R.; Adedire, E.; Balogun, M. S.; Olayinka, A. | 2020 | | Awareness and use of folic acid among women of childbearing age in Benue State, Nigeria | | unrelated topic |
| 1. Agbenorku, P.; Agbenorku, M.; Iddi, A.; Amevor, E.; Kofitse, M.; Klutsey, E. | 2011 | | Awareness of breast developmental anomalies: a study in Jamasi, Ghana | | unrelated topic |
| 1. Rabiu, T. B.; Tiamiyu, L. O.; Awoyinka, B. S. | 2012 | | Awareness of spina bifida and periconceptional use of folic acid among pregnant women in a developing economy | | unrelated topic |
| 1. Owotade, F. J.; Ogundipe, O. K.; Ugboko, V. I.; Okoje, V. N.; Olasoji, H. O.; Makinde, O. N.; Orji, E. O. | 2014 | | Awareness, knowledge and attitude on cleft lip and palate among antenatal clinic attendees of tertiary hospitals in Nigeria | | unrelated topic |
| 1. Pavlinac, P. B.; Singa, B. O.; Tickell, K. D.; Brander, R. L.; McGrath, C. J.; Amondi, M.; Otieno, J.; Akinyi, E.; Rwigi, D.; Carreon, J. D.; Tornberg-Belanger, S. N.; Nduati, R.; Babigumira, J. B.; Meshak, L.; Bogonko, G.; Kariuki, S.; Richardson, B. A.; John-Stewart, G. C.; Walson, J. L. | 2021 | | Azithromycin for the prevention of rehospitalisation and death among Kenyan children being discharged from hospital: a double-blind, placebo-controlled, randomised controlled trial | | unrelated topic |
| 1. Rauf, A.; Rauf, W. U.; Navsa, N.; Ashraf, K. T. | 2012 | | Azygos lobe in a South African cadaveric population | | unrelated topic |
| 1. Orenstein, L. A.; Orenstein, E. W.; Teguete, I.; Kodio, M.; Tapia, M.; Sow, S. O.; Levine, M. M. | 2012 | | Background rates of adverse pregnancy outcomes for assessing the safety of maternal vaccine trials in sub-Saharan Africa | | unrelated topic |
| 1. Worku, S.; Abebe, T.; Alemu, A.; Seyoum, B.; Swedberg, G.; Abdissa, A.; Mihret, A.; Beyene, G. T. | 2023 | | Bacterial profile of surgical site infection and antimicrobial resistance patterns in Ethiopia: a multicentre prospective cross-sectional study | | unrelated topic |
| 1. Tesfaye, E.; Tadele, H. | 2022 | | Bacterial Sepsis among Children with Congenital Heart Disease in Tikur Anbessa Specialized Hospital, Addis Ababa, Ethiopia | | unrelated topic |
| 1. Awoleke, J. O.; Adanikin, A. I. | 2016 | | Baird-Pattinson Aetiological Classification and Phases of Delay Contributing to Stillbirths in a Nigerian Tertiary Hospital | | unrelated topic |
| 1. Fieggen, K.; Milligan, C.; Henderson, B.; Esterhuizen, A. I. | 2016 | | Bardet Biedl syndrome in South Africa: A single founder mutation | | unrelated topic |
| 1. Kazibwe, H.; Struthers, P. | 2009 | | Barriers experienced by parents of children with clubfoot deformity attending specialised clinics in Uganda | | unrelated topic |
| 1. Kingau, N. W.; Rhoda, A.; Mlenzana, N. | 2015 | | Barriers experienced by service providers and caregivers in clubfoot management in Kenya | | unrelated topic |
| 1. Massenburg, B. B.; Jenny, H. E.; Saluja, S.; Meara, J. G.; Shrime, M. G.; Alonso, N. | 2016 | | Barriers to Cleft Lip and Palate Repair Around the World | | unrelated topic |
| 1. Oli, J. M.; Adeyemo, A. A.; Okafor, G. O.; Ofoegbu, E. N.; Onyenekwe, B.; Chukwuka, C. J.; Onwasigwe, C. N.; Ufelle, S.; Chen, G.; Rotimi, C. N. | 2009 | | Basal insulin resistance and secretion in Nigerians with type 2 diabetes mellitus | | unrelated topic |
| 1. Hermans, M. P.; Bouenizabila, E.; Amoussou-Guenou, D. K.; Ahn, S. A.; Rousseau, M. F. | 2015 | | Baseline diabetes as a way to predict CV outcomes in a lipid-modifying trial: a meta-analysis of 330,376 patients from 47 landmark studies | | unrelated topic |
| 1. Wandera, M.; Twa-Twa, J. | 2003 | | Baseline survey of oral health of primary and secondary school pupils in Uganda | | unrelated topic |
| 1. Viljoen, D.; Ramesar, R.; Behari, D. | 1991 | | Beals syndrome: clinical and molecular investigations in a kindred of Indian descent | | Year of publication |
| 1. Wolf, B. H.; Ikeogu, M. O. | 1993 | | Beckwith-Wiedemann syndrome in Bulawayo, Zimbabwe | | Year of publication |
| 1. Mouafo Tambo, F. F.; Badjang, G. T.; Kamga, G. F.; Sadjo, S. A.; Kouna Tsala, I. N.; Ondobo, G. A.; Sosso, M. A. | 2023 | | Bedside reduction of gastroschisis: A preliminary experience in yaounde-cameroon | | unrelated topic |
| 1. Imperato, G. H.; Imperato, P. J. | 2006 | | Beliefs and practices concerning twins, hermaphrodites, and albinos among the Bamana and Maninka of Mali | | Duplicate |
| 1. Imperato, G. H.; Imperato, P. J. | 2006 | | Beliefs and practices concerning twins, hermaphrodites, and albinos among the Bamana and Maninka of Mali | | unrelated topic |
| 1. Gillam, B. | 1973 | | Beliefs of the Wapei people about conception, childbirth, and early child care | | Year of publication |
| 1. Wadman, M. | 2019 | | Beset by neural tube defects, Ethiopia may fortify salt | | bookSection |
| 1. Wiysonge, C. S.; Bradley, H. A.; Volmink, J.; Mayosi, B. M.; Mbewu, A.; Opie, L. H. | 2012 | | Beta-blockers for hypertension | | Duplicate |
| 1. Wiysonge, C. S.; Bradley, H.; Mayosi, B. M.; Maroney, R.; Mbewu, A.; Opie, L. H.; Volmink, J. | 2007 | | Beta-blockers for hypertension | | unrelated topic |
| 1. Wiysonge, C. S.; Bradley, H. A.; Volmink, J.; Mayosi, B. M.; Mbewu, A.; Opie, L. H. | 2012 | | Beta-blockers for hypertension | | unrelated topic |
|  | 1985 | | Betel-quid and areca-nut chewing | | Year of publication |
| 1. Bettens, K.; Alighieri, C.; Bruneel, L.; D'Haeseleer, E.; Luyten, A.; Sseremba, D.; Musasizib, D.; Ojok, I.; Hodges, A.; Galiwango, G.; Vermeersch, H.; Van Lierde, K. | 2022 | | Better speech outcomes after very early palatal repair?-A longitudinal case-control study in Ugandan children with cleft palate | | unrelated topic |
| 1. Gilboa, S. M.; Gregory, C. J.; Honein, M. A. | 2019 | | Better surveillance to protect mothers and infants from Zika | | unrelated topic |
| 1. Diaouga, H. S.; Laurent, H. L.; Yacouba, M. C.; Mamane, F. L. A.; Rahamatou, M. G.; Idi, N.; Nayama, M. | 2022 | | Bicornuate uterus and pregnancy: ambiguity diagnosis (a case report) | | bookSection |
| 1. Singh, B. | 1990 | | Bilateral choanal atresia: key to success with the transnasal approach | | Year of publication |
| 1. Mwipopo, E.; Massomo, M. M.; Moshiro, R.; Manji, K. P. | 2023 | | Bilateral cryptophthalmos with overlapping features of Manitoba oculo-tricho-anal (MOTA) syndrome and Fraser syndrome 2 | | unrelated topic |
| 1. Gesase, A. P. | 2006 | | Bilateral hands and feet postaxial polydactyly presenting with renal vascular anomalies | | unrelated topic |
| 1. Pallangyo, P.; Lyimo, F.; Bhalia, S.; Makungu, H.; Nyangasa, B.; Lwakatare, F.; Suranyi, P.; Janabi, M. | 2017 | | Bilateral multiple pulmonary artery aneurysms associated with cavitary pulmonary tuberculosis: a case report | | unrelated topic |
| 1. Li, J.; Wang, T.; Richard, S. A.; Zhang, C.; Xie, X.; Wang, C. | 2020 | | Bilateral pediatric pial arteriovenous fistulas accompanying a giant arachnoid cyst with torticollis: A case report | | unrelated topic |
| 1. Ibrahim, A. G.; Aliyu, S.; Ali, N. | 2014 | | Bilateral pelvi-ureteric junction obstruction: our experience in a developing country | | Duplicate |
| 1. Ibrahim, A. G.; Aliyu, S.; Ali, N. | 2014 | | Bilateral pelvi-ureteric junction obstruction: our experience in a developing country | | unrelated topic |
| 1. Mabogunje, O. A. | 1987 | | Biliary atresia in Zaria, Nigeria: a review | | Year of publication |
| 1. Brits, E.; Le Grange, S. M. | 2023 | | Biliary atresia: The profile, management and outcome of patients treated at a tertiary hospital in central South Africa | | unrelated topic |
| 1. Hou, Y.; Cao, X.; Dong, L.; Wang, L.; Cheng, B.; Shi, Q.; Luo, X.; Bai, G. | 2012 | | Bioactivity-based liquid chromatography-coupled electrospray ionization tandem ion trap/time of flight mass spectrometry for β₂AR agonist identification in alkaloidal extract of Alstonia scholaris | | unrelated topic |
| 1. Akande, A. A.; Adelekan, A.; Abdulazeez, I. M. | 2021 | | Biochemical Assessment of Cardiovascular Risk Associated with the Duration of Antipsychotic Therapy in Patients with Schizophrenia | | unrelated topic |
| 1. Koskeroglu, K.; Barel, M.; Hizlisoy, H.; Yildirim, Y. | 2023 | | Biofilm formation and antibiotic resistance profiles of water-borne pathogens | | unrelated topic |
| 1. Ozdemir, S.; Aydogan, O.; Koksal Cakirlar, F. | 2021 | | Biofilm Formation and Antimicrobial Susceptibility of Non-Diphtheria Corynebacterium Strains Isolated from Blood Cultures: First Report from Turkey | | unrelated topic |
| 1. Kitti, T.; Seng, R.; Thummeepak, R.; Boonlao, C.; Jindayok, T.; Sitthisak, S. | 2019 | | Biofilm Formation of Methicillin-resistant Coagulase-Negative Staphylococci Isolated from Clinical Samples in Northern Thailand | | unrelated topic |
| 1. Tournoy, T. K.; Moons, P.; Daelman, B.; De Backer, J. | 2023 | | Biological Age in Congenital Heart Disease-Exploring the Ticking Clock | | unrelated topic |
| 1. Rango, T.; Vengosh, A.; Jeuland, M.; Whitford, G. M.; Tekle-Haimanot, R. | 2017 | | Biomarkers of chronic fluoride exposure in groundwater in a highly exposed population | | unrelated topic |
| 1. Hassanali, J.; Amwayi, P. | 1993 | | Biometric analysis of the dental casts of Maasai following traditional extraction of mandibular permanent central incisors and of Kikuyu children | | Year of publication |
| 1. Burnett, S. E.; Case, D. T. | 2011 | | Bipartite medial cuneiform: new frequencies from skeletal collections and a meta-analysis of previous cases | | unrelated topic |
| 1. Ersdal, H. L.; Mduma, E.; Svensen, E.; Perlman, J. | 2012 | | Birth asphyxia: a major cause of early neonatal mortality in a Tanzanian rural hospital | | unrelated topic |
| 1. Bera, E.; McCausland, K.; Nonkwelo, R.; Mgudlwa, B.; Chacko, S.; Majeke, B. | 2010 | | Birth defects following exposure to efavirenz-based antiretroviral therapy during pregnancy: a study at a regional South African hospital | | unrelated topic |
| 1. Dryden, R. | 1997 | | Birth defects recognized in 10,000 babies born consecutively in Port Moresby General Hospital, Papua New Guinea | | Year of publication |
| 1. Sayed, A. R.; Bourne, D. E.; Nixon, J. M.; Klopper, J. M.; Op't Hof, J. | 1989 | | Birth defects surveillance. A pilot system in the Cape Peninsula | | Year of publication |
| 1. Bateman, C. | 2003 | | Birth deformities–a heavy burden | | unrelated topic |
| 1. Mehta, U. C.; van Schalkwyk, C.; Naidoo, P.; Ramkissoon, A.; Mhlongo, O.; Maharaj, N. R.; Naidoo, N.; Fieggen, K.; Urban, M. F.; Krog, S.; Welte, A.; Dheda, M.; Pillay, Y.; Moran, N. F. | 2019 | | Birth outcomes following antiretroviral exposure during pregnancy: Initial results from a pregnancy exposure registry in South Africa | | unrelated topic |
| 1. Tesfay, N.; Hailu, G.; Habtetsion, M.; Woldeyohannes, F. | 2023 | | Birth prevalence and risk factors of neural tube defects in Ethiopia: a systematic review and meta-analysis | | unrelated topic |
| 1. Theron, A.; Loveland, J. | 2015 | | Birth prevalence of anorectal malformation in the referral area for the University of the Witwatersrand tertiary hospitals, South Africa | | unrelated topic |
| 1. Theron, A.; Numanoglu, A. | 2017 | | Birth Prevalence of Anorectal Malformations for the Western Cape Province, South Africa, 2005 to 2012 | | unrelated topic |
| 1. Sadoh, W. E.; Okonkwo, I.; Okonkwo, C. A.; Eki-Udoko, F. E.; Emeruwa, E.; Monday, P.; Osueni, G. I.; Amake, J.; Eyo-Ita, E.; Otaigbe, B. E.; Oko-Oboh, G. A. | 2021 | | Birth prevalence of congenital heart disease among newborns in a tertiary hospital in Benin City, Nigeria | | Duplicate |
| 1. Sadoh, W. E.; Okonkwo, I.; Okonkwo, C. A.; Eki-Udoko, F. E.; Emeruwa, E.; Monday, P.; Osueni, G. I.; Amake, J.; Eyo-Ita, E.; Otaigbe, B. E.; Oko-Oboh, G. A. | 2021 | | Birth prevalence of congenital heart disease among newborns in a tertiary hospital in Benin City, Nigeria | | unrelated topic |
| 1. Linde, D Van Der; Konings, EEM; Slager, MA; ... | 2011 | | Birth prevalence of congenital heart disease worldwide: a systematic review and meta-analysis | | Duplicate |
| 1. Linde, D Van Der; Konings, EEM; Slager, MA; ... | 2011 | | Birth prevalence of congenital heart disease worldwide: a systematic review and meta-analysis | | Non primary research |
| 1. D Van Der Linde, EEM Konings, MA Slager… | 2011 | | Birth prevalence of congenital heart disease worldwide: a systematic review and meta-analysis | | unrelated topic |
| 1. Smythe, T.; Kuper, H.; Macleod, D.; Foster, A.; Lavy, C. | 2017 | | Birth prevalence of congenital talipes equinovarus in low- and middle-income countries: a systematic review and meta-analysis | | unrelated topic |
| 1. Oumer, M.; Tazebew, A.; Silamsaw, M. | 2021 | | Birth prevalence of neural tube defects and associated risk factors in Africa: a systematic review and meta-analysis | | unrelated topic |
| 1. Ssentongo, P.; Heilbrunn, E. S.; Ssentongo, A. E.; Ssenyonga, L. V. N.; Lekoubou, A. | 2022 | | Birth prevalence of neural tube defects in eastern Africa: a systematic review and meta-analysis | | unrelated topic |
| 1. Tiruneh, C.; Gebremeskel, T.; Necho, M.; Teshome, Y.; Teshome, D.; Belete, A. | 2022 | | Birth prevalence of omphalocele and gastroschisis in Sub-Saharan Africa: A systematic review and meta-analysis | | unrelated topic |
| 1. Kishimba, R. S.; Mpembeni, R.; Mghamba, J. M.; Goodman, D.; Valencia, D. | 2015 | | Birth prevalence of selected external structural birth defects at four hospitals in Dar es Salaam, Tanzania, 2011-2012 | | unrelated topic |
| 1. Mekonen, H. K.; Nigatu, B.; Lamers, W. H. | 2015 | | Birth weight by gestational age and congenital malformations in Northern Ethiopia | | unrelated topic |
| 1. Engs, RC | 2022 | | Bizarre Medicine | | book |
| 1. Zumelzu, C.; Le Roux-Villet, C.; Loiseau, P.; Busson, M.; Heller, M.; Aucouturier, F.; Pendaries, V.; Lièvre, N.; Pascal, F.; Brette, M. D.; Doan, S.; Charron, D.; Caux, F.; Laroche, L.; Petit, A.; Prost-Squarcioni, C. | 2011 | | Black patients of African descent and HLA-DRB1*15:03 frequency overrepresented in epidermolysis bullosa acquisita | | unrelated topic |
| 1. Alao, M. J.; Lalèyè, A.; Lalya, F.; Hans, Ch; Abramovicz, M.; Morice-Picard, F.; Arveiler, B.; Lacombe, D.; Rooryck, C. | 2012 | | Blepharophimosis, ptosis, epicanthus inversus syndrome with translocation and deletion at chromosome 3q23 in a black African female | | unrelated topic |
| 1. Beiram, M. M. | 1971 | | Blindness in the Sudan: prevalence and causes in Blue Nile Province | | Year of publication |
| 1. Kim, M. D.; Eun, S. Y.; Jo, S. H. | 2006 | | Blockade of HERG human K+ channel and IKr of guinea pig cardiomyocytes by prochlorperazine | | unrelated topic |
| 1. Ombelet, S.; Kpossou, G.; Kotchare, C.; Agbobli, E.; Sogbo, F.; Massou, F.; Lagrou, K.; Barbé, B.; Affolabi, D.; Jacobs, J. | 2022 | | Blood culture surveillance in a secondary care hospital in Benin: epidemiology of bloodstream infection pathogens and antimicrobial resistance | | unrelated topic |
| 1. Chen, Y.; Jia, T.; Yan, X.; Dai, L. | 2020 | | Blood glucose fluctuations in patients with coronary heart disease and diabetes mellitus correlates with heart rate variability: A retrospective analysis of 210 cases | | unrelated topic |
| 1. Gavrilovska-Brzanov, A | 2023 | | BLOOD LOSS AND FLUID REPLACEMENT IN PEDIATRIC PATIENTS | | unrelated topic |
| 1. Neilson, G.; Williams, G. | 1978 | | Blood pressure and valvular and congenital heart disease in Torres Strait Islanders | | Year of publication |
| 1. Erick, P. | 2020 | | Botswana: country report on children's environmental health | | unrelated topic |
| 1. Ameh, E. A. | 2001 | | Bowel resection in children | | unrelated topic |
| 1. Abdur-Rahman, L. O.; Adeniran, J. O.; Taiwo, J. O.; Nasir, A. A.; Odi, T. | 2009 | | Bowel resection in Nigerian children | | unrelated topic |
| 1. Apolot, D.; Erem, G.; Nassanga, R.; Kiggundu, D.; Tumusiime, C. M.; Teu, A.; Mugisha, A. M.; Sebunya, R. | 2022 | | Brain magnetic resonance imaging findings among children with epilepsy in two urban hospital settings, Kampala-Uganda: a descriptive study | | Duplicate |
| 1. Apolot, D.; Erem, G.; Nassanga, R.; Kiggundu, D.; Tumusiime, C. M.; Teu, A.; Mugisha, A. M.; Sebunya, R. | 2022 | | Brain magnetic resonance imaging findings among children with epilepsy in two urban hospital settings, Kampala-Uganda: a descriptive study | | unrelated topic |
| 1. Balcha, T. E.; Woldeyohannes, A. M.; Neknek, G. A. | 2022 | | Brain Magnetic Resonance Imaging Findings in Patients with Developmental Delay in Addis Ababa, Ethiopia | | unrelated topic |
| 1. Adekunle, A. A.; Adamson, O.; James, O.; Ogunlewe, O. M.; Butali, A.; Adeyemo, W. L. | 2020 | | Breastfeeding Practices Among Mothers of Children With Orofacial Clefts in an African Cohort | | unrelated topic |
| 1. Rossi, D; Granström, AL; Teunissen, NM; ... | 2023 | | Bridging the Gap: Reporting Baseline Characteristics, Process and Outcome Parameters in Hirschsprung's Disease. A Systematic Review | | book |
| 1. Kiweewa, F. M.; Tierney, C.; Butler, K.; Peters, M. G.; Vhembo, T.; Moodley, D.; Govender, V.; Mohtashemi, N.; Ship, H.; Musoke, P.; Dula, D.; George, K.; Chakhtoura, N.; Fowler, M. G.; Currier, J. S.; Bhattacharya, D. | 2022 | | Brief Report: Impact of Antiretroviral Regimen on Pregnancy and Infant Outcomes in Women With HIV/ HBV Coinfection | | unrelated topic |
|  | 2005 | | Brief report: Imported case of congenital rubella syndrome–New Hampshire, 2005 | | unrelated topic |
| 1. Ullrich, S. J.; Kakembo, N.; Grabski, D. F.; Cheung, M.; Kisa, P.; Nabukenya, M.; Tumukunde, J.; Fitzgerald, T. N.; Langer, M.; Situma, M.; Sekabira, J.; Ozgediz, D. | 2020 | | Burden and Outcomes of Neonatal Surgery in Uganda: Results of a Five-Year Prospective Study | | unrelated topic |
| 1. Nabwera, H. M.; Wang, D.; Tongo, O. O.; Andang'o, P. E. A.; Abdulkadir, I.; Ezeaka, C. V.; Ezenwa, B. N.; Fajolu, I. B.; Imam, Z. O.; Mwangome, M. K.; Umoru, D. D.; Akindolire, A. E.; Otieno, W.; Nalwa, G. M.; Talbert, A. W.; Abubakar, I.; Embleton, N. D.; Allen, S. J. | 2021 | | Burden of disease and risk factors for mortality amongst hospitalized newborns in Nigeria and Kenya | | unrelated topic |
| 1. Grabski, D. F.; Kakembo, N.; Situma, M.; Cheung, M.; Shikanda, A.; Okello, I.; Kisa, P.; Muzira, A.; Sekabira, J.; Ozgediz, D. | 2020 | | Burden of emergency pediatric surgical procedures on surgical capacity in Uganda: a new metric for health system performance | | unrelated topic |
| 1. Abdul-Mumin, A.; Anyomih, T. T. K.; Owusu, S. A.; Wright, N.; Decker, J.; Niemeier, K.; Benavidez, G.; Abantanga, F. A.; Smith, E. R.; Tabiri, S. | 2020 | | Burden of Neonatal Surgical Conditions in Northern Ghana | | unrelated topic |
| 1. Wakoya, R.; Afework, M. | 2023 | | Burden of Neural Tube Defects and Their Associated Factors in Africa: A Systematic Review and Meta-Analysis | | unrelated topic |
| 1. MD Connor, R Walker, G Modi, CP Warlow | 2007 | | Burden of stroke in black populations in sub-Saharan Africa | | unrelated topic |
| 1. Wu, V. K.; Poenaru, D.; Poley, M. J. | 2013 | | Burden of surgical congenital anomalies in Kenya: a population-based study | | Duplicate |
| 1. Wu, V. K.; Poenaru, D.; Poley, M. J. | 2013 | | Burden of surgical congenital anomalies in Kenya: a population-based study | | unrelated topic |
| 1. Wu, V. K.; Poenaru, D. | 2013 | | Burden of surgically correctable disabilities among children in the Dadaab Refugee Camp | | unrelated topic |
| 1. F Ataklte, S Erqou, S Kaptoge, B Taye… | 2015 | | Burden of undiagnosed hypertension in sub-saharan Africa: a systematic review and meta-analysis | | unrelated topic |
| 1. Arts, H. H.; Bongers, E. M.; Mans, D. A.; van Beersum, S. E.; Oud, M. M.; Bolat, E.; Spruijt, L.; Cornelissen, E. A.; Schuurs-Hoeijmakers, J. H.; de Leeuw, N.; Cormier-Daire, V.; Brunner, H. G.; Knoers, N. V.; Roepman, R. | 2011 | | C14ORF179 encoding IFT43 is mutated in Sensenbrenner syndrome | | unrelated topic |
| 1. Delany-Moretlwe, S.; Hughes, J. P.; Bock, P.; Ouma, S. G.; Hunidzarira, P.; Kalonji, D.; Kayange, N.; Makhema, J.; Mandima, P.; Mathew, C.; Spooner, E.; Mpendo, J.; Mukwekwerere, P.; Mgodi, N.; Ntege, P. N.; Nair, G.; Nakabiito, C.; Nuwagaba-Biribonwoha, H.; Panchia, R.; Singh, N.; Siziba, B.; Farrior, J.; Rose, S.; Anderson, P. L.; Eshleman, S. H.; Marzinke, M. A.; Hendrix, C. W.; Beigel-Orme, S.; Hosek, S.; Tolley, E.; Sista, N.; Adeyeye, A.; Rooney, J. F.; Rinehart, A.; Spreen, W. R.; Smith, K.; Hanscom, B.; Cohen, M. S.; Hosseinipour, M. C. | 2022 | | Cabotegravir for the prevention of HIV-1 in women: results from HPTN 084, a phase 3, randomised clinical trial | | unrelated topic |
| 1. Ghidoni, A.; Elliott, P. M.; Syrris, P.; Calkins, H.; James, C. A.; Judge, D. P.; Murray, B.; Barc, J.; Probst, V.; Schott, J. J.; Song, J. P.; Hauer, R. N. W.; Hoorntje, E. T.; van Tintelen, J. P.; Schulze-Bahr, E.; Hamilton, R. M.; Mittal, K.; Semsarian, C.; Behr, E. R.; Ackerman, M. J.; Basso, C.; Parati, G.; Gentilini, D.; Kotta, M. C.; Mayosi, B. M.; Schwartz, P. J.; Crotti, L. | 2021 | | Cadherin 2-Related Arrhythmogenic Cardiomyopathy: Prevalence and Clinical Features | | unrelated topic |
| 1. Huang, Z. J.; Dai, D. Z.; Li, N.; Na, T.; Ji, M.; Dai, Y. | 2007 | | Calcium antagonist property of CPU228, a dofetilide derivative, contributes to its low incidence of torsades de pointes in rabbits | | unrelated topic |
| 1. Hofmeyr, G. J.; Lawrie, T. A.; Atallah, A. N.; Duley, L.; Torloni, M. R. | 2014 | | Calcium supplementation during pregnancy for preventing hypertensive disorders and related problems | | unrelated topic |
| 1. Hofmeyr, G. J.; Lawrie, T. A.; Atallah Á, N.; Torloni, M. R. | 2018 | | Calcium supplementation during pregnancy for preventing hypertensive disorders and related problems | | unrelated topic |
| 1. Limpitikul, W. B.; Dick, I. E.; Joshi-Mukherjee, R.; Overgaard, M. T.; George, Jr., A. L.; Yue, D. T. | 2014 | | Calmodulin mutations associated with long QT syndrome prevent inactivation of cardiac L-type Ca(2+) currents and promote proarrhythmic behavior in ventricular myocytes | | unrelated topic |
| 1. Dryden, R. | 1999 | | Can birth defects be prevented? | | Year of publication |
| 1. Mwanda, O. W. | 1999 | | Cancers in children younger than age 16 years in Kenya | | Year of publication |
| 1. Yehouenou, C. L.; Soleimani, R.; Kpangon, A. A.; Simon, A.; Dossou, F. M.; Dalleur, O. | 2022 | | Carbapenem-Resistant Organisms Isolated in Surgical Site Infections in Benin: A Public Health Problem | | unrelated topic |
| 1. Brown, S. C.; Henderson, B. D.; Buys, D. A.; Theron, M.; Long, M. A.; Smit, F. | 2010 | | Cardiac abnormalities and facial anthropometric measurements in children from the Free State and Northern Cape provinces of South Africa with chromosome 22q11.2 microdeletion | | unrelated topic |
| 1. Ali, S. K. | 2009 | | Cardiac abnormalities of Sudanese patients with Down's syndrome and their short-term outcome | | unrelated topic |
| 1. Ramphul, K.; Ramphul, Y.; Verma, R.; Kumar, N.; Joynauth, J. | 2021 | | Cardiac Arrhythmias During Sickle Cell Disease Crisis in the United States | | unrelated topic |
| 1. Brown, K. G.; Willis, W. H. | 1975 | | Cardiac disease in Malawi | | Year of publication |
| 1. Nqayana, T.; Moodley, J.; Naidoo, D. P. | 2008 | | Cardiac disease in pregnancy | | unrelated topic |
| 1. Soliman, E. Z.; Juma, H. | 2008 | | Cardiac disease patterns in northern Malawi: epidemiologic transition perspective | | age scope |
| 1. Parkar, A. H. | 1973 | | Cardiac disorders in Nairobi | | Year of publication |
| 1. Coundoul, Aliou Mar and Faye, Abdou Aziz and Bop, Khadim and Fall, Amadou Lamine and Faye, Papa Moctar and Boye, Ndeye Fatou and Faye, Ndeye Tenning and Ndiaye, Ousmane | 2023 | | Cardiac Echography in Pediatrics at the Regional Hospital of Diourbel: Indication and Result | | Included |
| 1. Vervoort, D.; Vinck, E. E.; Tiwari, K. K.; Tapaua, N. | 2021 | | Cardiac Surgery and Small Island States: A Bridge Too Far? | | unrelated topic |
| 1. Mirabel, M.; Lachaud, M.; Offredo, L.; Lachaud, C.; Zuschmidt, B.; Ferreira, B.; Sidi, D.; Chauvaud, S.; Sok, P.; Deloche, A.; Marijon, E.; Jouven, X. | 2017 | | Cardiac surgery in low-income settings: 10 years of experience from two countries | | unrelated topic |
| 1. Byiringiro, S.; Nyirimanzi, N.; Mucumbitsi, J.; Kamanzi, E. R.; Swain, J. | 2020 | | Cardiac Surgery: Increasing Access in Low- and Middle-Income Countries | | unrelated topic |
| 1. Somers, K. | 1976 | | Cardiology: Africa versus Australia | | Year of publication |
| 1. Towbin, JA; Jefferies, JL | 2017 | | Cardiomyopathies due to left ventricular noncompaction, mitochondrial and storage diseases, and inborn errors of metabolism | | unrelated topic |
| 1. Towbin, JA; Jefferies, JL | 2017 | | Cardiomyopathy Compendium | | book |
| 1. Nabawanuka, E.; Ameda, F.; Erem, G.; Bugeza, S.; Opoka, R. O.; Kiguli, S.; Amorut, D.; Aloroker, F.; Olupot-Olupot, P.; Mnjalla, H.; Mpoya, A.; Maitland, K. | 2023 | | Cardiovascular abnormalities in chest radiographs of children with pneumonia, Uganda | | unrelated topic |
| 1. Lukhna, K.; Cupido, B.; Hitzeroth, J.; Chin, A.; Ntsekhe, M. | 2020 | | Cardiovascular care in sub-Saharan Africa during the COVID-19 crisis: lessons from the global experience | | unrelated topic |
| 1. AP Kengne, AGB Amoah, JC Mbanya | 2005 | | Cardiovascular complications of diabetes mellitus in sub-Saharan Africa | | unrelated topic |
| 1. Dimopoulos, K.; Constantine, A.; Clift, P.; Condliffe, R.; Moledina, S.; Jansen, K.; Inuzuka, R.; Veldtman, G. R.; Cua, C. L.; Tay, E. L. W.; Opotowsky, A. R.; Giannakoulas, G.; Alonso-Gonzalez, R.; Cordina, R.; Capone, G.; Namuyonga, J.; Scott, C. H.; D'Alto, M.; Gamero, F. J.; Chicoine, B.; Gu, H.; Limsuwan, A.; Majekodunmi, T.; Budts, W.; Coghlan, G.; Broberg, C. S. | 2023 | | Cardiovascular Complications of Down Syndrome: Scoping Review and Expert Consensus | | unrelated topic |
| 1. FP Cappuccio, MA Miller | 2016 | | Cardiovascular disease and hypertension in sub-Saharan Africa: burden, risk and interventions | | unrelated topic |
| 1. AK Keates, AO Mocumbi, M Ntsekhe, K Sliwa… | 2017 | | Cardiovascular disease in Africa: epidemiological profile and challenges | | unrelated topic |
| 1. Massoure, P. L.; Roche, N. C.; Lamblin, G.; Dehan, C.; Kaiser, E.; Fourcade, L. | 2013 | | Cardiovascular disease in children in Djibouti: a single-centre study | | Duplicate |
| 1. Massoure P, Roche NC, Lamblin G, Dehan C, Kaiser É, Fourcade L | 2013 | | Cardiovascular disease in children in Djibouti: a single-centre study | | Included |
| 1. Abengowe, C. U. | 1979 | | Cardiovascular disease in Northern Nigeria | | Year of publication |
| 1. Korner, N. | 1980 | | Cardiovascular disease in Papua New Guinea | | Year of publication |
| 1. K Turk-Adawi, N Sarrafzadegan, I Fadhil… | 2018 | | Cardiovascular disease in the Eastern Mediterranean region: epidemiology and risk factor burden | | unrelated topic |
| 1. Bucciarelli-Ducci, C.; Ostenfeld, E.; Baldassarre, L. A.; Ferreira, V. M.; Frank, L.; Kallianos, K.; Raman, S. V.; Srichai, M. B.; McAlindon, E.; Mavrogeni, S.; Ntusi, N. A. B.; Schulz-Menger, J.; Valente, A. M.; Ordovas, K. G. | 2020 | | Cardiovascular disease in women: insights from magnetic resonance imaging | | unrelated topic |
| 1. Yuyun, M. F.; Sliwa, K.; Kengne, A. P.; Mocumbi, A. O.; Bukhman, G. | 2020 | | Cardiovascular Diseases in Sub-Saharan Africa Compared to High-Income Countries: An Epidemiological Perspective | | unrelated topic |
| 1. MF Yuyun, K Sliwa, AP Kengne, AO Mocumbi… | 2020 | | Cardiovascular diseases in sub-Saharan Africa compared to high-income countries: an epidemiological perspective | | unrelated topic |
| 1. Temu, T. M.; Kirui, N.; Wanjalla, C.; Ndungu, A. M.; Kamano, J. H.; Inui, T. S.; Bloomfield, G. S. | 2015 | | Cardiovascular health knowledge and preventive practices in people living with HIV in Kenya | | unrelated topic |
| 1. Herrey, A. S.; Francis, J. M.; Hughes, M.; Ntusi, N. A. B. | 2019 | | Cardiovascular magnetic resonance can be undertaken in pregnancy and guide clinical decision-making in this patient population | | unrelated topic |
| 1. AA Alsheikh-Ali, MI Omar, FJ Raal, W Rashed… | 2014 | | Cardiovascular risk factor burden in Africa and the Middle East: the Africa Middle East cardiovascular epidemiological (ACE) study | | unrelated topic |
| 1. Ikeme, A. C.; Pole, D. J.; Pobee, J. O.; Larbi, E.; Blankson, J.; Williams, H. | 1978 | | Cardiovascular status and blood pressure in a population sample in Ghana–the Mamprobi survey | | Year of publication |
| 1. Ibrahim, Y. E.; Bjorvatn, K.; Birkeland, J. M. | 1997 | | Caries and dental fluorosis in a 0.25 and a 2.5 ppm fluoride area in the Sudan | | Year of publication |
| 1. Almerich-Silla, J. M.; Montiel-Company, J. M.; Ruiz-Miravet, A. | 2008 | | Caries and dental fluorosis in a western Saharan population of refugee children | | unrelated topic |
| 1. Awadia, A. K.; Birkeland, J. M.; Haugejorden, O.; Bjorvatn, K. | 2002 | | Caries experience and caries predictors–a study of Tanzanian children consuming drinking water with different fluoride concentrations | | unrelated topic |
| 1. Duru, C. O.; Okpokowuruk, F. S.; Adesina, A. D.; Worgu, G. O.; Adeniji, F. O.; Chinawa, J. M.; Aliyu, I. | 2021 | | Caring for Children with Congenital Heart Diseases:Economic Burden of Pre-Surgical Management on Nigerian Families | | unrelated topic |
| 1. Levine, E. | 1972 | | Carpal fusions in children of four South African populations | | Year of publication |
| 1. Lodhia, J.; Rego-Garcia, I.; Koipapi, S.; Sadiq, A.; Msuya, D.; Spaendonk, R. V.; Hamel, B.; Dekker, M. | 2021 | | Carpenter syndrome in a patient from Tanzania | | unrelated topic |
| 1. Ajiji, P | 2022 | | Cartography of risks related to drug exposure during pregnancy | | book |
| 1. Baltimore, R. S.; Nimkin, K.; Sparger, K. A.; Pierce, V. M.; Plotkin, S. A. | 2018 | | Case 4-2018: A Newborn with Thrombocytopenia, Cataracts, and Hepatosplenomegaly | | unrelated topic |
| 1. Danbauchi, S. S.; Alhassan, M. A. | 2002 | | Case report: dextrocardia with situs inversus; two cases presenting differently | | case report |
| 1. Hamdoun, E.; Karachunski, P.; Nathan, B.; Fischer, M.; Torkelson, J. L.; Drilling, A.; Petryk, A. | 2016 | | Case Report: The Specter of Untreated Congenital Hypothyroidism in Immigrant Families | | unrelated topic |
| 1. Zake, E. Z. | 1984 | | Case reports of 16 sets of conjoined twins from a Uganda hospital | | Year of publication |
| 1. Desalew, A.; Sintayehu, Y.; Teferi, N.; Amare, F.; Geda, B.; Worku, T.; Abera, K.; Asefaw, A. | 2020 | | Cause and predictors of neonatal mortality among neonates admitted to neonatal intensive care units of public hospitals in eastern Ethiopia: a facility-based prospective follow-up study | | unrelated topic |
| 1. Tchirkov, V.; Ambassa, S.; Siddiqui, M. A. | 2012 | | Causes and consequences of the deficiencies of the lower limbs in the Republic of Guinea | | unrelated topic |
| 1. Fiander, A. | 1990 | | Causes of infertility among 1000 patients in Ghana | | Year of publication |
| 1. Mdala, J. F.; Mash, R. | 2015 | | Causes of mortality and associated modifiable health care factors for children (< 5-years) admitted at Onandjokwe Hospital, Namibia | | unrelated topic |
| 1. Ayeni, O. | 1980 | | Causes of mortality in an African city | | Year of publication |
| 1. Osifo, O. D.; Oku, O. R. | 2009 | | Causes, spectrum and effects of surgical child abuse and neglect in a Nigerian city | | unrelated topic |
| 1. McLean, M.; Dutton, M. F. | 1995 | | Cellular interactions and metabolism of aflatoxin: an update | | Year of publication |
| 1. Adeleye, A. O.; Olowookere, K. G. | 2009 | | Central nervous system congenital anomalies: a prospective neurosurgical observational study from Nigeria | | unrelated topic |
| 1. Adeleye, A. O.; Dairo, M. D.; Olowookere, K. G. | 2010 | | Central nervous system congenital malformations in a developing country: issues and challenges against their prevention | | unrelated topic |
| 1. Kauw, F.; Kranenburg, G.; Kappelle, L. J.; Hendrikse, J.; Koek, H. L.; Visseren, F. L. J.; Mali, W. P. T.; de Jong, P. A.; Spiering, W. | 2017 | | Cerebral disease in a nationwide Dutch pseudoxanthoma elasticum cohort with a systematic review of the literature | | unrelated topic |
| 1. Akpede, G. O.; Ambe, J. P. | 2000 | | Cerebral herniation in pyogenic meningitis: prevalence and related dilemmas in emergency room populations in developing countries | | unrelated topic |
| 1. Paton, G. J.; Billings, B. K. | 2020 | | Cervical Rib Synostosis to the First Rib: A Rare Anatomic Variation | | unrelated topic |
| 1. Pohl, A. P. | 1976 | | Cervical ribs in black mineworkers | | Year of publication |
| 1. Uche, E. O.; Okorie, E.; Emejulu, J.; Ajuzieogu, O.; Uche, N. J. | 2016 | | Challenges and outcome of cranial neuroendoscopic surgery in a resource constrained developing African country | | Duplicate |
| 1. Uche, E. O.; Okorie, E.; Emejulu, J.; Ajuzieogu, O.; Uche, N. J. | 2016 | | Challenges and outcome of cranial neuroendoscopic surgery in a resource constrained developing African country | | unrelated topic |
| 1. Hasan, B.; Hansmann, G.; Budts, W.; Heath, A.; Hoodbhoy, Z.; Jing, Z. C.; Koestenberger, M.; Meinel, K.; Mocumbi, A. O.; Radchenko, G. D.; Sallmon, H.; Sliwa, K.; Kumar, R. K. | 2020 | | Challenges and Special Aspects of Pulmonary Hypertension in Middle- to Low-Income Regions: JACC State-of-the-Art Review | | unrelated topic |
| 1. Tekendo-Ngongang, C.; Dahoun, S.; Nguefack, S.; Gimelli, S.; Sloan-Béna, F.; Wonkam, A. | 2014 | | Challenges in clinical diagnosis of williams-beuren syndrome in sub-saharan africans: case reports from cameroon | | bookSection |
| 1. Abubakar, A. M.; Bello, M. A.; Chinda, J. Y.; Danladi, K.; Umar, I. M. | 2011 | | Challenges in the management of early versus late presenting congenital diaphragmatic hernia in a poor resource setting | | Duplicate |
| 1. Abubakar, A. M.; Bello, M. A.; Chinda, J. Y.; Danladi, K.; Umar, I. M. | 2011 | | Challenges in the management of early versus late presenting congenital diaphragmatic hernia in a poor resource setting | | unrelated topic |
| 1. McCulloch, M.; Luyckx, V. A.; Cullis, B.; Davies, S. J.; Finkelstein, F. O.; Yap, H. K.; Feehally, J.; Smoyer, W. E. | 2021 | | Challenges of access to kidney care for children in low-resource settings | | unrelated topic |
| 1. Olasoji, H. O.; Hassan, A.; Ligali, T. O. | 2009 | | Challenges of cleft care in Africa | | unrelated topic |
| 1. Erhabor, G. E. | 2021 | | Challenges of Congenital Heart Disease and the Impact of Covid-19 on Health Workers | | unrelated topic |
| 1. Osifo, O. D.; Efobi, A. C. | 2009 | | Challenges of giant ventral hernia repair in children in an African tertiary care center with limited resources | | unrelated topic |
| 1. Ekenze, S. O.; Ajuzieogu, O. V.; Nwomeh, B. C. | 2016 | | Challenges of management and outcome of neonatal surgery in Africa: a systematic review | | unrelated topic |
| 1. Manga, T.; Masuku, K. P. | 2020 | | Challenges of teaching the deaf-blind learner in an education setting in Johannesburg: Experiences of educators and assistant educators | | unrelated topic |
| 1. Mocumbi, A. O.; Lameira, E.; Yaksh, A.; Paul, L.; Ferreira, M. B.; Sidi, D. | 2011 | | Challenges on the management of congenital heart disease in developing countries | | unrelated topic |
| 1. Katusabe, J. L.; Hodges, A.; Galiwango, G. W.; Mulogo, E. M. | 2018 | | Challenges to achieving low palatal fistula rates following primary cleft palate repair: experience of an institution in Uganda | | unrelated topic |
| 1. Christé, G.; Chahine, M.; Chevalier, P.; Pásek, M. | 2008 | | Changes in action potentials and intracellular ionic homeostasis in a ventricular cell model related to a persistent sodium current in SCN5A mutations underlying LQT3 | | unrelated topic |
| 1. Moss, S. J. | 2009 | | Changes in coronary heart disease risk profile of adults with intellectual disabilities following a physical activity intervention | | unrelated topic |
| 1. Postoev, V. A.; Grjibovski, A. M.; Nieboer, E.; Odland, JØ | 2015 | | Changes in detection of birth defects and perinatal mortality after introduction of prenatal ultrasound screening in the Kola Peninsula (North-West Russia): combination of two birth registries | | unrelated topic |
| 1. Postoev, V. A.; Grjibovski, A. M.; Nieboer, E.; Odland, JØ | 2015 | | Changes in detection of birth defects and perinatal mortality after introduction of prenatal ultrasound screening in the Kola Peninsula (North-West Russia): combination of two birth registries | | unrelated topic |
| 1. Yang, J.; Reinach, P. S.; Zhang, S.; Pan, M.; Sun, W.; Liu, B.; Li, F.; Li, X.; Zhao, A.; Chen, T.; Jia, W.; Qu, J.; Zhou, X. | 2017 | | Changes in retinal metabolic profiles associated with form deprivation myopia development in guinea pigs | | unrelated topic |
| 1. Kolesinska, Z.; Ahmed, S. F.; Niedziela, M.; Bryce, J.; Molinska-Glura, M.; Rodie, M.; Jiang, J.; Sinnott, R. O.; Hughes, I. A.; Darendeliler, F.; Hiort, O.; van der Zwan, Y.; Cools, M.; Guran, T.; Holterhus, P. M.; Bertelloni, S.; Lisa, L.; Arlt, W.; Krone, N.; Ellaithi, M.; Balsamo, A.; Mazen, I.; Nordenstrom, A.; Lachlan, K.; Alkhawari, M.; Chatelain, P.; Weintrob, N. | 2014 | | Changes over time in sex assignment for disorders of sex development | | unrelated topic |
| 1. Liu, A.; Diller, G. P.; Moons, P.; Daniels, C. J.; Jenkins, K. J.; Marelli, A. | 2023 | | Changing epidemiology of congenital heart disease: effect on outcomes and quality of care in adults | | age scope |
| 1. Castelli, JB | 2013 | | chapter 32 1402 CMDT 2013 | | book |
| 1. Snyman, Y.; Whitelaw, A. C.; Barnes, J. M.; Maloba, M. R. B.; Newton-Foot, M. | 2021 | | Characterisation of mobile colistin resistance genes (mcr-3 and mcr-5) in river and storm water in regions of the Western Cape of South Africa | | unrelated topic |
| 1. Labuschagne, C.; Karzis, J.; Britz, H.; Petzer, I. M. | 2022 | | Characterisation of Staphylococci Isolated from Milk Samples of a Water Buffalo Herd | | unrelated topic |
| 1. Purcell, L.; Ngwira, N.; Gallaher, J.; Cairns, B.; Charles, A. | 2019 | | Characteristics and outcomes in paediatric patients presenting with congenital colorectal diseases in sub-Saharan Africa | | unrelated topic |
| 1. Kloosterman, M.; Oldgren, J.; Conen, D.; Wong, J. A.; Connolly, S. J.; Avezum, A.; Yusuf, S.; Ezekowitz, M. D.; Wallentin, L.; Ntep-Gweth, M.; Joseph, P.; Barrett, T. W.; Tanosmsup, S.; McIntyre, W. F.; Lee, S. F.; Parkash, R.; Amit, G.; Grinvalds, A.; Van Gelder, I. C.; Healey, J. S. | 2020 | | Characteristics and outcomes of atrial fibrillation in patients without traditional risk factors: an RE-LY AF registry analysis | | unrelated topic |
| 1. L Zühlke, ME Engel, G Karthikeyan… | 2015 | | Characteristics, complications, and gaps in evidence-based interventions in rheumatic heart disease: the Global Rheumatic Heart Disease Registry (the REMEDY … | | unrelated topic |
| 1. Fraser, D.; DeRoo, C. S.; Cody, R. B.; Armitage, R. A. | 2013 | | Characterization of blood in an encrustation on an African mask: spectroscopic and direct analysis in real time mass spectrometric identification of haem | | unrelated topic |
| 1. Pretorius, N. O.; Rhode, K.; Simpson, J. M.; Pasch, H. | 2015 | | Characterization of complex phthalic acid/propylene glycol based polyesters by the combination of 2D chromatography and MALDI-TOF mass spectrometry | | unrelated topic |
| 1. Hetsa, B. A.; Kumar, A.; Ateba, C. N. | 2018 | | Characterization of multiple antibiotic resistant clinical strains of Staphylococcus isolated from pregnant women vagina | | unrelated topic |
| 1. Morena, J.; Gupta, A.; Hoyle, J. C. | 2019 | | Charcot-Marie-Tooth: From Molecules to Therapy | | unrelated topic |
| 1. Denham, A. R.; Adongo, P. B.; Freydberg, N.; Hodgson, A. | 2010 | | Chasing spirits: Clarifying the spirit child phenomenon and infanticide in Northern Ghana | | unrelated topic |
| 1. Enslin, J. M. N.; Fieggen, A. G.; Figaji, A. | 2019 | | Chiari 1 malformation management: the Red Cross War Memorial Hospital approach | | unrelated topic |
| 1. Elhadji Cheikh Ndiaye, S. Y.; Troude, L.; Al-Falasi, M.; Faye, M.; Melot, A.; Roche, P. H. | 2019 | | Chiari malformations in adults: A single center surgical experience with special emphasis on the kinetics of clinical improvement | | unrelated topic |
| 1. Kassebaum, N.; Kyu, H. H.; Zoeckler, L.; Olsen, H. E.; Thomas, K.; Pinho, C.; Bhutta, Z. A.; Dandona, L.; Ferrari, A.; Ghiwot, T. T.; Hay, S. I.; Kinfu, Y.; Liang, X.; Lopez, A.; Malta, D. C.; Mokdad, A. H.; Naghavi, M.; Patton, G. C.; Salomon, J.; Sartorius, B.; Topor-Madry, R.; Vollset, S. E.; Werdecker, A.; Whiteford, H. A.; Abate, K. H.; Abbas, K.; Damtew, S. A.; Ahmed, M. B.; Akseer, N.; Al-Raddadi, R.; Alemayohu, M. A.; Altirkawi, K.; Abajobir, A. A.; Amare, A. T.; Antonio, C. A. T.; Arnlov, J.; Artaman, A.; Asayesh, H.; Avokpaho, Efga; Awasthi, A.; Ayala Quintanilla, B. P.; Bacha, U.; Betsu, B. D.; Barac, A.; Bärnighausen, T. W.; Baye, E.; Bedi, N.; Bensenor, I. M.; Berhane, A.; Bernabe, E.; Bernal, O. A.; Beyene, A. S.; Biadgilign, S.; Bikbov, B.; Boyce, C. A.; Brazinova, A.; Hailu, G. B.; Carter, A.; Castañeda-Orjuela, C. A.; Catalá-López, F.; Charlson, F. J.; Chitheer, A. A.; Choi, J. J.; Ciobanu, L. G.; Crump, J.; Dandona, R.; Dellavalle, R. P.; Deribew, A.; deVeber, G.; Dicker, D.; Ding, E. L.; Dubey, M.; Endries, A. Y.; Erskine, H. E.; Faraon, E. J. A.; Faro, A.; Farzadfar, F.; Fernandes, J. C.; Fijabi, D. O.; Fitzmaurice, C.; Fleming, T. D.; Flor, L. S.; Foreman, K. J.; Franklin, R. C.; Fraser, M. S.; Frostad, J. J.; Fullman, N.; Gebregergs, G. B.; Gebru, A. A.; Geleijnse, J. M.; Gibney, K. B.; Gidey Yihdego, M.; Ginawi, I. A. M.; Gishu, M. D.; Gizachew, T. A.; Glaser, E.; Gold, A. L.; Goldberg, E.; Gona, P.; Goto, A.; others | 2017 | | Child and Adolescent Health From 1990 to 2015: Findings From the Global Burden of Diseases, Injuries, and Risk Factors 2015 Study | | unrelated topic |
| 1. Harrison, K. A. | 1985 | | Child-bearing, health and social priorities: a survey of 22 774 consecutive hospital births in Zaria, Northern Nigeria | | Year of publication |
| 1. Bode-Thomas, F.; Ige, O. O.; Yilgwan, C. | 2013 | | Childhood acquired heart diseases in Jos, north central Nigeria | | unrelated topic |
| 1. Waddell, K. M. | 1998 | | Childhood blindness and low vision in Uganda | | Year of publication |
| 1. Ezomike, U. O.; Nwachukwu, I. E.; Nwangwu, E. I.; Chukwu, I. S.; Aliozor, S. C.; Nwankwo, E. P.; Ekenze, S. O. | 2022 | | Childhood colostomies: patterns, indications and outcomes in a Nigerian University Teaching Hospital | | unrelated topic |
| 1. Viljoen, D. L.; Dent, G. M.; Sibanda, A. G.; Seymour, M.; Chigumo, R.; Karikoga, A.; Beighton, P. | 1988 | | Childhood deafness in Zimbabwe | | Duplicate |
| 1. Viljoen, D. L.; Dent, G. M.; Sibanda, A. G.; Seymour, M.; Chigumo, R.; Karikoga, A.; Beighton, P. | 1988 | | Childhood deafness in Zimbabwe | | Year of publication |
| 1. Danaya, R. T. | 1995 | | Childhood disabilities in Papua New Guinea | | Year of publication |
| 1. Uba, A. F.; Edino, S. T.; Yakubu, A. A.; Sheshe, A. A. | 2004 | | Childhood intestinal obstruction in Northwestern Nigeria | | unrelated topic |
| 1. Abdurrahman, M. B.; Babaoye, F. A.; Aikhionbare, H. A. | 1990 | | Childhood renal disorders in Nigeria | | Year of publication |
| 1. Sporns, PB; Fullerton, HJ; Lee, S; Kim, H; Lo, WD; ... | 2022 | | Childhood stroke | | unrelated topic |
| 1. Sporns, PB; Fullerton, HJ; Lee, S; Kim, H; ... | 2022 | | Childhood stroke (Primer) | | unrelated topic |
| 1. Boolkah, S. | 2010 | | Children at heart | | unrelated topic |
| 1. Hartman, E. E.; Oort, F. J.; Aronson, D. C.; van der Steeg, A. F.; Heij, H. A.; van Heurn, E.; Madern, G. C.; van der Zee, D. C.; de Blaauw, I.; van Dijk, A.; Sprangers, M. A. | 2015 | | Children With Anorectal Malformations, Hirschsprung Disease, and Their Siblings: Proxy Reports and Self-Reports | | unrelated topic |
| 1. Sims-Williams, H. J.; Sims-Williams, H. P.; Mbabazi Kabachelor, E.; Magombe, J.; Warf, B. C. | 2019 | | Children with spina bifida in Eastern Uganda report a reasonable quality of life relative to their healthy school-attending peers | | bookSection |
| 1. Animasahun, B. A.; Madise-Wobo, A. D.; Omokhodion, S. I.; Njokanma, O. F. | 2015 | | Children With Tetralogy of Fallot in an Urban Centre in Africa | | study population |
| 1. Animasahun, B. A.; Madise-Wobo, A. D.; Gbelee, H. O.; Omokhodion, S. I. | 2017 | | Children with transposition of the great arteries: Should they actually be born in Nigeria? | | unrelated topic |
| 1. Kittur, F. S.; Vishu Kumar, A. B.; Varadaraj, M. C.; Tharanathan, R. N. | 2005 | | Chitooligosaccharides–preparation with the aid of pectinase isozyme from Aspergillus niger and their antibacterial activity | | unrelated topic |
| 1. Huber, D.; Tegl, G.; Baumann, M.; Sommer, E.; Gorji, E. G.; Borth, N.; Schleining, G.; Nyanhongo, G. S.; Guebitz, G. M. | 2017 | | Chitosan hydrogel formation using laccase activated phenolics as cross-linkers | | unrelated topic |
| 1. Rossouw, J. E.; Thompson, M. L.; Jooste, P. L.; Swanepoel, A. S.; Jordaan, P. C. | 1990 | | Choice of coronary heart disease risk factor variables in a cross-sectional study of white South Africans | | Duplicate |
| 1. Rossouw, J. E.; Thompson, M. L.; Jooste, P. L.; Swanepoel, A. S.; Jordaan, P. C. | 1990 | | Choice of coronary heart disease risk factor variables in a cross-sectional study of white South Africans | | Year of publication |
| 1. Sun, H. L.; Chu, W. F.; Dong, D. L.; Liu, Y.; Bai, Y. L.; Wang, X. H.; Zhou, J.; Yang, B. F. | 2006 | | Choline-modulated arsenic trioxide-induced prolongation of cardiac repolarization in Guinea pig | | unrelated topic |
| 1. Arnaud, A.; Harper, L.; Aulagne, M. B.; Michel, J. L.; Maurel, A.; Dobremez, E.; Fourcade, L.; Andriamananarivo, L. | 2011 | | Choosing a technique for severe hypospadias | | unrelated topic |
| 1. Uhumwangho, O. M.; Jalali, S. | 2014 | | Chorioretinal coloboma in a paediatric population | | unrelated topic |
| 1. Koen, L | 2008 | | Chromosomal aberrations in the Xhosa schizophrenia population | | book |
| 1. Nkanza, N. K.; Tobani, C. T. | 1991 | | Chromosomal abnormalities: experience at Harare Hospital | | Year of publication |
| 1. Moore, S. W. | 2012 | | Chromosomal and related Mendelian syndromes associated with Hirschsprung's disease | | unrelated topic |
| 1. Mgone, C. S.; Lembeli, C. M. | 1981 | | Chromosomal disorders as seen at Muhimbili Medical Centre Dar Es Salaam | | Year of publication |
| 1. Adeniyi, A. | 1973 | | Chromosomal pattern, renal, cardiovascular and lymphatic abnormalities in a Nigerian child with Turner's syndrome and relative hypogamma-globulinaemia | | Year of publication |
| 1. Koen, L.; Niehaus, D. J.; Wright, G.; Warnich, L.; De Jong, G.; Emsley, R. A.; Mall, S. | 2012 | | Chromosome 22q11 in a Xhosa schizophrenia population | | unrelated topic |
| 1. Ally, F. E.; Grace, H. J. | 1979 | | Chromosome abnormalities in South African mental retardates | | Duplicate |
| 1. Ally, F. E.; Grace, H. J. | 1979 | | Chromosome abnormalities in South African mental retardates | | Year of publication |
| 1. Hewitt, A. J.; Knuff, A. L.; Jefkins, M. J.; Collier, C. P.; Reynolds, J. N.; Brien, J. F. | 2011 | | Chronic ethanol exposure and folic acid supplementation: fetal growth and folate status in the maternal and fetal guinea pig | | unrelated topic |
| 1. Schultheiss, W. A.; Godley, G. A. | 1995 | | Chronic fluorosis in cattle due to the ingestion of a commercial lick | | Year of publication |
| 1. Imani, P. D.; Aujo, J.; Kiguli, S.; Srivaths, P.; Brewer, E. D. | 2021 | | Chronic kidney disease impacts health-related quality of life of children in Uganda, East Africa | | unrelated topic |
| 1. Anochie, I.; Eke, F. | 2003 | | Chronic renal failure in children: a report from Port Harcourt, Nigeria (1985-2000) | | unrelated topic |
| 1. Ali el, T. M.; Abdelraheem, M. B.; Mohamed, R. M.; Hassan, E. G.; Watson, A. R. | 2009 | | Chronic renal failure in Sudanese children: aetiology and outcomes | | unrelated topic |
| 1. Gunn, C | 2007 | | Churchill Livingstone Pocket Radiography and Medical Imaging Dictionary E-Book: Churchill Livingstone Pocket Radiography and Medical Imaging … | | book |
| 1. Ghosh, A.; Choudhury, A.; Das, A.; Chatterjee, N. S.; Das, T.; Chowdhury, R.; Panda, K.; Banerjee, R.; Chatterjee, I. B. | 2012 | | Cigarette smoke induces p-benzoquinone-albumin adduct in blood serum: Implications on structure and ligand binding properties | | unrelated topic |
| 1. Lumley, J.; Correy, J. F.; Newman, N. M.; Curran, J. T. | 1985 | | Cigarette smoking, alcohol consumption and fetal outcome in Tasmania 1981-82 | | Year of publication |
| 1. Ainsworth, C. | 2007 | | Cilia: tails of the unexpected | | bookSection |
| 1. Modekwe, V. I.; Ekwunife, O. H.; Ugwu, J. O.; Ugwunne, C. A.; Ndukwu, C. U.; Obiegbu, H. O.; Obidike, A. B. | 2023 | | Classical bladder exstrophy in an adolescent: A case report on management, challenges and outcome | | bookSection |
| 1. Oginni, F. O.; Oladele, A. O.; Adenekan, A. T.; Olabanji, J. K. | 2014 | | Cleft care in Nigeria: past, present, and future | | unrelated topic |
| 1. Pham, A. M.; Tollefson, T. T. | 2007 | | Cleft deformities in Zimbabwe, Africa: socioeconomic factors, epidemiology, and surgical reconstruction | | unrelated topic |
| 1. Osuji, O. O.; Ogar, D. I.; Akande, O. O. | 1994 | | Cleft lip and palate as seen in the University College Hospital, Ibadan | | Year of publication |
| 1. Rakotoarison, R. A.; Rakotoarivony, A. E.; Rabesandratana, N.; Razafindrabe, J. B.; Andriambololona, R.; Andriambololo-Nivo, R.; Feki, A. | 2012 | | Cleft lip and palate in Madagascar 1998-2007 | | unrelated topic |
| 1. Adekeye, E. O.; Lavery, K. M. | 1985 | | Cleft lip and palate in Nigerian children and adults: a comparative study | | Year of publication |
| 1. Kanmounye, U. S.; Dutton, J. L.; Naidu, P.; Msokera, C.; Collier, Z. J.; Fernanda Tapia, M.; Mikhail, S.; Xepoleas, M.; Auslander, A.; Yao, C. A.; Magee, 3rd, W. | 2022 | | Cleft Lip and Palate Research in Low- and Middle-income Countries: A Scientometric Analysis | | unrelated topic |
| 1. Wilson, J.; Hodges, A. | 2012 | | Cleft lip and palate surgery carried out by one team in Uganda: where have all the palates gone? | | unrelated topic |
| 1. Kwari, D. Y.; Chinda, J. Y.; Olasoji, H. O.; Adeosun, O. O. | 2010 | | Cleft lip and palate surgery in children: anaesthetic considerations | | unrelated topic |
| 1. Wanjeri, J. K.; Wachira, J. M. | 2009 | | Cleft lip and palate: a descriptive comparative, retrospective, and prospective study of patients with cleft deformities managed at 2 hospitals in Kenya | | Duplicate |
| 1. Wanjeri, J. K.; Wachira, J. M. | 2009 | | Cleft lip and palate: a descriptive comparative, retrospective, and prospective study of patients with cleft deformities managed at 2 hospitals in Kenya | | unrelated topic |
| 1. Orkar, K. S.; Ugwu, B. T.; Momoh, J. T. | 2002 | | Cleft lip and palate: the Jos experience | | unrelated topic |
| 1. Kruppa, K.; Krüger, E.; Vorster, C.; der Linde, J. V. | 2022 | | Cleft Lip and/or Palate and Associated Risks in Lower-Middle-Income Countries: A Systematic Review | | unrelated topic |
| 1. Gordon, H.; Davies, D.; Botha, V.; Friedberg, S. | 1969 | | Cleft lip palate in Cape Town | | Year of publication |
| 1. Tollefson, T. T.; Shaye, D.; Durbin-Johnson, B.; Mehdezadeh, O.; Mahomva, L.; Chidzonga, M. | 2015 | | Cleft lip-cleft palate in Zimbabwe: estimating the distribution of the surgical burden of disease using geographic information systems | | unrelated topic |
| 1. Staz, J. | 1988 | | Cleidocranial dysplasia in the South Western Cape: preliminary report | | Year of publication |
| 1. Roberts, T.; Stephen, L.; Beighton, P. | 2013 | | Cleidocranial dysplasia: a review of the dental, historical, and practical implications with an overview of the South African experience | | unrelated topic |
| 1. Nsibu, N. C.; Jaeken, J.; Carchon, H.; Mampunza, M.; Sturiale, L.; Garozzo, D.; Mashako, M. N.; Tshibassu, M. P. | 2008 | | Clinical and biochemical features in a Congolese infant with congenital disorder of glycosylation (CDG)-IIx | | unrelated topic |
| 1. Ali, S. K.; Nimeri, N. A. | 2006 | | Clinical and echocardiographic features of Ebstein's malformation in Sudanese patients | | unrelated topic |
| 1. Lambie, L.; Amin, R.; Essop, F.; Cnaan, A.; Krause, A.; Guay-Woodford, L. M. | 2015 | | Clinical and genetic characterization of a founder PKHD1 mutation in Afrikaners with ARPKD | | unrelated topic |
| 1. Moore, S. W.; Zaahl, M. | 2009 | | Clinical and genetic differences in total colonic aganglionosis in Hirschsprung's disease | | Duplicate |
| 1. Moore, S. W.; Zaahl, M. | 2009 | | Clinical and genetic differences in total colonic aganglionosis in Hirschsprung's disease | | unrelated topic |
| 1. Kayvanpour, E; Sedaghat-Hamedani, F; Gi, WT; ... | 2019 | | Clinical and genetic insights into non-compaction: a meta-analysis and systematic review on 7598 individuals | | unrelated topic |
| 1. Oloyede, O. A.; Olaide, A.; Onyinye, N. | 2014 | | Clinical and laboratory experience of chorionic villous sampling in Nigeria | | unrelated topic |
| 1. Kemeny, S.; Pebrel-Richard, C.; Eymard-Pierre, E.; Gay-Bellile, M.; Gouas, L.; Goumy, C.; Tchirkov, A.; Francannet, C.; Vago, P. | 2014 | | Clinical and molecular description of a 17q21.33 microduplication in a girl with severe kyphoscoliosis and developmental delay | | unrelated topic |
| 1. Salih, M. A.; Mahdi, A. H.; al-Rikabi, A. C.; al-Bunyan, M.; Roberds, S. L.; Anderson, R. D.; Campbell, K. P. | 1996 | | Clinical and molecular pathological features of severe childhood autosomal recessive muscular dystrophy in Saudi Arabia | | Year of publication |
| 1. Aydinbelge, M.; Sekerci, A. E.; Caliskan, S.; Gumus, H.; Sisman, Y.; Cantekin, K. | 2017 | | Clinical and radiographic evaluation of double teeth in primary dentition and associated anomalies in the permanent successors | | Duplicate |
| 1. Aydinbelge, M.; Sekerci, A. E.; Caliskan, S.; Gumus, H.; Sisman, Y.; Cantekin, K. | 2017 | | Clinical and radiographic evaluation of double teeth in primary dentition and associated anomalies in the permanent successors | | unrelated topic |
| 1. Musa, G.; Simfukwe, K.; Gots, A.; Chmutin, G.; Chmutin, E.; Chaurasia, B. | 2020 | | Clinical and radiological characteristics in fatal third ventricle colloid cyst. Literature review | | unrelated topic |
| 1. Thylstrup, A.; Fejerskov, O. | 1978 | | Clinical appearance of dental fluorosis in permanent teeth in relation to histologic changes | | Year of publication |
| 1. Sitnikov, A. R. | 2007 | | Clinical case of the late diagnosis of type-II schizencephaly | | unrelated topic |
| 1. Wozniak, PS | 2023 | | Clinical challenges to the concept of ectogestation | | unrelated topic |
| 1. Oladokun, R.; Muloiwa, R.; Hsiao, N. Y.; Valley-Omar, Z.; Nuttall, J.; Eley, B. | 2016 | | Clinical characterisation and phylogeny of respiratory syncytial virus infection in hospitalised children at Red Cross War Memorial Children's Hospital, Cape Town | | unrelated topic |
| 1. Szymanski, P. Z.; Badri, M.; Mayosi, B. M. | 2018 | | Clinical characteristics and causes of heart failure, adherence to treatment guidelines, and mortality of patients with acute heart failure: Experience at Groote Schuur Hospital, Cape Town, South Africa | | unrelated topic |
| 1. Knutzen, V. K.; Baillie, P.; Malan, A. F. | 1975 | | Clinical classification of perinatal deaths | | Year of publication |
| 1. Faundes, V.; Goh, S.; Akilapa, R.; Bezuidenhout, H.; Bjornsson, H. T.; Bradley, L.; Brady, A. F.; Brischoux-Boucher, E.; Brunner, H.; Bulk, S.; Canham, N.; Cody, D.; Dentici, M. L.; Digilio, M. C.; Elmslie, F.; Fry, A. E.; Gill, H.; Hurst, J.; Johnson, D.; Julia, S.; Lachlan, K.; Lebel, R. R.; Byler, M.; Gershon, E.; Lemire, E.; Gnazzo, M.; Lepri, F. R.; Marchese, A.; McEntagart, M.; McGaughran, J.; Mizuno, S.; Okamoto, N.; Rieubland, C.; Rodgers, J.; Sasaki, E.; Scalais, E.; Scurr, I.; Suri, M.; van der Burgt, I.; Matsumoto, N.; Miyake, N.; Benoit, V.; Lederer, D.; Banka, S. | 2021 | | Clinical delineation, sex differences, and genotype-phenotype correlation in pathogenic KDM6A variants causing X-linked Kabuki syndrome type 2 | | unrelated topic |
| 1. Bive, B. Z.; Sacheli, R.; Situakibanza Nani-Tuma, H.; Kabututu Zakayi, P.; Ka, A.; Mbula Mambimbi, M.; Muendele, G.; Boreux, R.; Landu, N.; Nzanzu Mudogo, C.; M'Buze, P. R.; Moutschen, M.; Meyer, W.; Mvumbi Lelo, G.; Hayette, M. P. | 2022 | | Clinical epidemiology and high genetic diversity amongst Cryptococcus spp. isolates infecting people living with HIV in Kinshasa, Democratic Republic of Congo | | unrelated topic |
| 1. Ekure, E. N.; Kalu, N.; Sokunbi, O. J.; Kruszka, P.; Olusegun-Joseph, A. D.; Ikebudu, D.; Bala, D.; Muenke, M.; Adeyemo, A. | 2018 | | Clinical epidemiology of congenital heart disease in Nigerian children, 2012-2017 | | Duplicate |
| 1. Ekure, E. N.; Kalu, N.; Sokunbi, O. J.; Kruszka, P.; Olusegun-Joseph, A. D.; Ikebudu, D.; Bala, D.; Muenke, M.; Adeyemo, A. | 2018 | | Clinical epidemiology of congenital heart disease in Nigerian children, 2012-2017 | | Included |
| 1. Duke, R.; Torty, C.; Nwachukwu, K.; Ameh, S.; Kim, M.; Eneli, N.; Onyedikachi, A.; Aghaji, A.; Burton, K.; Dyet, L.; Bowman, R. | 2020 | | Clinical features and aetiology of cerebral palsy in children from Cross River State, Nigeria | | unrelated topic |
| 1. Christianson, A. L.; Kromberg, J. G.; Viljoen, E. | 1995 | | Clinical features of Black African neonates with Down's syndrome | | Year of publication |
| 1. Watkins, D. A.; Hendricks, N.; Shaboodien, G.; Mbele, M.; Parker, M.; Vezi, B. Z.; Latib, A.; Chin, A.; Little, F.; Badri, M.; Moolman-Smook, J. C.; Okreglicki, A.; Mayosi, B. M. | 2009 | | Clinical features, survival experience, and profile of plakophylin-2 gene mutations in participants of the arrhythmogenic right ventricular cardiomyopathy registry of South Africa | | unrelated topic |
| 1. Moore, S. W.; Albertyn, R.; Cywes, S. | 1996 | | Clinical outcome and long-term quality of life after surgical correction of Hirschsprung's disease | | Year of publication |
| 1. de Baat, T.; Lester, R.; Ghambi, L.; Twabi, H. H.; Nielsen, M.; Gordon, S. B.; van Weissenbruch, M. M.; Feasey, N. A.; Dube, Q.; Kawaza, K.; Iroh Tam, P. Y. | 2023 | | Clinical predictors of bacteraemia in neonates with suspected early-onset sepsis in Malawi: a prospective cohort study | | unrelated topic |
| 1. Mubungu, G.; Makay, P.; Boujemla, B.; Yanda, S.; Posey, J. E.; Lupski, J. R.; Bours, V.; Lukusa, P.; Devriendt, K.; Lumaka, A. | 2021 | | Clinical presentation and evolution of Xia-Gibbs syndrome due to p.Gly375ArgfsTer3 variant in a patient from DR Congo (Central Africa) | | unrelated topic |
| 1. Arogundade, F. A.; Akinbodewa, A. A.; Sanusi, A. A.; Okunola, O.; Hassan, M. O.; Akinsola, A. | 2018 | | Clinical presentation and outcome of autosomal dominant polycystic kidney disease in Nigeria | | unrelated topic |
| 1. Makrexeni, Z. M.; Pepeta, L. | 2017 | | Clinical presentation and outcomes of patients with acute rheumatic fever and rheumatic heart disease seen at a tertiary hospital setting in Port Elizabeth, South Africa | | Duplicate |
| 1. Makrexeni, Z. M.; Pepeta, L. | 2017 | | Clinical presentation and outcomes of patients with acute rheumatic fever and rheumatic heart disease seen at a tertiary hospital setting in Port Elizabeth, South Africa | | unrelated topic |
| 1. Galal, M. S.; Musa, S. A.; Babiker, O. O.; Hamdan, H. Z.; Abdullah, M. A. | 2022 | | Clinical profile and aetiologies of delayed puberty: a 15 years' experience from a tertiary centre in Sudan | | unrelated topic |
| 1. Ekenze, S. O.; Chikani, U.; Ezomike, U. O.; Adiri, C. O.; Onuh, A. | 2022 | | Clinical profile and management challenges of disorders of sex development in Africa: a systematic review | | unrelated topic |
| 1. Gama, M.; Abitew, B.; Abebe, K. | 2022 | | Clinical Profiles and Surgical Outcome of Hypospadias Repair at a Teaching Hospital in Ethiopia | | Duplicate |
| 1. Gama, M.; Abitew, B.; Abebe, K. | 2022 | | Clinical Profiles and Surgical Outcome of Hypospadias Repair at a Teaching Hospital in Ethiopia | | unrelated topic |
| 1. Connor, S. J.; Brisighelli, G.; Patel, N.; Levitt, M. A. | 2021 | | Clinical Quiz-A Rare Case of Anal Canal Duplication in the Context of Currarino Syndrome | | bookSection |
| 1. Mazhani, T.; Steenhoff, A. P.; Tefera, E.; David, T.; Patel, Z.; Sethomo, W.; Smieja, M.; Loeto, L. | 2020 | | Clinical spectrum and prevalence of congenital heart disease in children in Botswana | | Duplicate |
| 1. Mazhani, T.; Steenhoff, A. P.; Tefera, E.; David, T.; Patel, Z.; Sethomo, W.; Smieja, M.; Loeto, L. | 2020 | | Clinical spectrum and prevalence of congenital heart disease in children in Botswana | | Included |
| 1. Nanda, A.; Liu, L.; Al-Ajmi, H.; Al-Saleh, Q. A.; Al-Fadhli, S.; Anim, J. T.; Ozoemena, L.; Mellerio, J. E.; McGrath, J. A. | 2018 | | Clinical subtypes and molecular basis of epidermolysis bullosa in Kuwait | | unrelated topic |
| 1. Ngo Um, S. S.; Betoko, R. M.; Mekone, I.; Chetcha, A. B.; Tardy, V.; Dahoun, S.; Mure, P. Y.; Plotton, I.; Morel, Y.; Etoga, M. E.; Nengom, J. T.; Moifo, B.; Tambo, F. M.; Sobngwi, E.; Ndombo, P. K. | 2022 | | Clinical, biochemical, and biomolecular aspects of congenital adrenal hyperplasia in a group of Cameroonian children and adolescents | | unrelated topic |
| 1. Kamdem, F.; Kedy Koum, D.; Hamadou, B.; Yemdji, M.; Luma, H.; Doualla, M. S.; Noukeu, D.; Barla, E.; Akazong, C.; Dzudie, A.; Ngote, H.; Monkam, Y.; Mouliom, S.; Kingue, S. | 2018 | | Clinical, echocardiographic, and therapeutic aspects of congenital heart diseases of children at Douala General Hospital: A cross-sectional study in sub-Saharan Africa | | Included |
| 1. Plit, M. L.; Blott, J. A.; Lakis, N.; Murray, J.; Plit, M. | 1997 | | Clinical, radiographic and lung function features of diffuse congenital cystic adenomatoid malformation of the lung in an adult | | Year of publication |
| 1. Oladayo, A.; Gowans, L. J. J.; Awotoye, W.; Alade, A.; Busch, T.; Naicker, T.; Eshete, M. A.; Adeyemo, W. L.; Hetmanski, J. B.; Zeng, E.; Adamson, O.; Adeleke, C.; Li, M.; Sule, V.; Kayali, S.; Olotu, J.; Mossey, P. A.; Obiri-Yeboah, S.; Buxo, C. J.; Beaty, T.; Taub, M.; Donkor, P.; Marazita, M. L.; Odukoya, O.; Adeyemo, A. A.; Murray, J. C.; Prince, A.; Butali, A. | 2023 | | Clinically actionable secondary findings in 130 triads from sub-Saharan African families with non-syndromic orofacial clefts | | unrelated topic |
| 1. Aggarwal, M.; Ornish, D.; Josephson, R.; Brown, T. M.; Ostfeld, R. J.; Gordon, N.; Madan, S.; Allen, K.; Khetan, A.; Mahmoud, A.; Freeman, A. M.; Aspry, K. | 2021 | | Closing Gaps in Lifestyle Adherence for Secondary Prevention of Coronary Heart Disease | | unrelated topic |
| 1. Bedford, K. J.; Chidothi, P.; Sakala, H.; Cashman, J.; Lavy, C. | 2011 | | Clubfoot in Malawi: local theories of causation | | unrelated topic |
| 1. Bedford, K. J.; Chidothi, P.; Sakala, H.; Cashman, J.; Lavy, C. | 2011 | | Clubfoot in Malawi: treatment-seeking behaviour | | Duplicate |
| 1. Majiyagbe, OO; Akinsete, AM; Adeyemo, TA; Salako, AO; ... | 2022 | | Coagulation abnormalities in children with uncorrected congenital heart defects seen at a teaching hospital in a developing country | | book |
| 1. Majiyagbe, OO; Akinsete, AM; Adeyemo, TA; Salako, AO; ... | 2022 | | Coagulation abnormalities in children with uncorrected congenital heart defects seen at a teaching hospital in a developing country | | Duplicate |
| 1. Majiyagbe, O. O.; Akinsete, A. M.; Adeyemo, T. A.; Salako, A. O.; Ekure, E. N.; Okoromah, C. A. N. | 2022 | | Coagulation abnormalities in children with uncorrected congenital heart defects seen at a teaching hospital in a developing country | | unrelated topic |
| 1. Pegoraro, R. J.; Ranjith, N.; Rom, L. | 2005 | | Coagulation gene polymorphisms as risk factors for myocardial infarction in young Indian Asians | | unrelated topic |
| 1. Hansen, D. P.; Silverstein, D. M.; Shah, S. K.; Warshow, M. M.; Ojiambo, H. P. | 1978 | | Coarctation of the aorta in Nairobi: A report of ten cases | | Year of publication |
| 1. Manyama, M.; Mazyala, E.; Mahalu, W. | 2015 | | Co-existence of patent ductus arteriosus and left brachiocephalic artery: a case report | | unrelated topic |
| 1. Obiechina, A. E.; Arotiba, J. T.; Ogunbiyi, J. O. | 1999 | | Coexisting congenital sublingual dermoid and bronchogenic cyst | | Year of publication |
| 1. Murphree, S. M.; Dunkley, A. S. | 1992 | | Colon atresia and stenosis in Zimbabwe: case reports and a review of the literature | | Year of publication |
| 1. Carneiro, P. M.; Doig, C. M. | 1993 | | Colon interposition for wide gap oesophageal atresia | | Year of publication |
| 1. Ameh, E. A.; Mshelbwala, P. M.; Sabiu, L.; Chirdan, L. B. | 2006 | | Colostomy in children–an evaluation of acceptance among mothers and caregivers in a developing country | | unrelated topic |
| 1. Ameh, E. A. | 2002 | | Colostomy in the newborn: technical pitfalls | | unrelated topic |
| 1. Odendaal, H. J.; Steyn, D. W.; Elliott, A.; Burd, L. | 2009 | | Combined effects of cigarette smoking and alcohol consumption on perinatal outcome | | unrelated topic |
| 1. Warf, B. C.; Campbell, J. W. | 2008 | | Combined endoscopic third ventriculostomy and choroid plexus cauterization as primary treatment of hydrocephalus for infants with myelomeningocele: long-term results of a prospective intent-to-treat study in 115 East African infants | | unrelated topic |
| 1. Theron, A.; Loveland, J.; Naidoo, J. | 2012 | | Combined oesophageal atresia with upper pouch fistula and meconium peritonitis | | unrelated topic |
| 1. Strobele, B.; Loveland, J.; Britz, R.; Gottlich, E.; Welthagen, A.; Botha, J. | 2013 | | Combined paediatric liver-kidney transplantation: analysis of our experience and literature review | | unrelated topic |
| 1. Tsague, L.; Abrams, E. J. | 2014 | | Commentary: Antiretroviral treatment for pregnant and breastfeeding women–the shifting paradigm | | unrelated topic |
| 1. Kromberg, J. G.; Jenkins, T. | 1982 | | Common birth defects in South African Blacks | | Year of publication |
| 1. Kotaska, K.; Lisá, L.; Průsa, R. | 2003 | | Common CYP21 gene mutations in Czech patients and statistical analysis of worldwide mutation distribution | | unrelated topic |
| 1. Ejim, E. C.; Ubani-Ukoma, C. B.; Nwaneli, U. C.; Onwubere, B. J. | 2013 | | Common echocardiographic abnormalities in Nigerians of different age groups | | age scope |
| 1. Robson, S. C.; Potter, P. C. | 1990 | | Common variable immunodeficiency in association with Turner's syndrome | | Year of publication |
| 1. Tadesse, L.; Tafesse, F.; Hamamy, H. | 2014 | | Communities and community genetics in Ethiopia | | unrelated topic |
| 1. Compaoré, A.; Gies, S.; Brabin, B.; Tinto, H.; Brabin, L. | 2018 | | Community approval required for periconceptional adolescent adherence to weekly iron and/or folic acid supplementation: a qualitative study in rural Burkina Faso | | unrelated topic |
| 1. Sule, S. S.; Onayade, A. A. | 2006 | | Community-based antenatal and perinatal interventions and newborn survival | | unrelated topic |
| 1. Oyedele, T. A.; Folayan, M. O.; Adekoya-Sofowora, C. A.; Oziegbe, E. O. | 2015 | | Co-morbidities associated with molar-incisor hypomineralisation in 8 to 16 year old pupils in Ile-Ife, Nigeria | | unrelated topic |
| 1. Yang, H. L.; Chang, N. T.; Wang, J. K.; Lu, C. W.; Huang, Y. C.; Moons, P. | 2020 | | Comorbidity as a mediator of depression in adults with congenital heart disease: A population-based cohort study | | unrelated topic |
| 1. Hlongwa, P.; Dandajena, T. C.; Rispel, L. C. | 2019 | | Comparative analysis of healthcare provision to individuals with cleft lip and/or palate at specialised academic centres in South Africa | | unrelated topic |
| 1. Serunjogi, R.; Barlow-Mosha, L.; Mumpe-Mwanja, D.; Williamson, D.; Valencia, D.; Tinker, S. C.; Adler, M. R.; Namale-Matovu, J.; Kalibbala, D.; Nankunda, J.; Nabunya, E.; Birabwa-Male, D.; Byamugisha, J.; Musoke, P. | 2021 | | Comparative analysis of perinatal outcomes and birth defects amongst adolescent and older Ugandan mothers: evidence from a hospital-based surveillance database | | unrelated topic |
| 1. Gaziano, T. A.; Pandya, A.; Steyn, K.; Levitt, N.; Mollentze, W.; Joubert, G.; Walsh, C. M.; Motala, A. A.; Kruger, A.; Schutte, A. E.; Naidoo, D. P.; Prakaschandra, D. R.; Laubscher, R. | 2013 | | Comparative assessment of absolute cardiovascular disease risk characterization from non-laboratory-based risk assessment in South African populations | | unrelated topic |
| 1. Qin, J.; Zhao, Y.; Wang, A.; Chi, X.; Wen, P.; Li, S.; Wu, L.; Bi, S.; Xu, H. | 2021 | | Comparative genomic characterization of multidrug-resistant Citrobacter spp. strains in Fennec fox imported to China | | unrelated topic |
| 1. Tahir, I. S.; Vos, A. G.; Damen, J. A. A.; Barth, R. E.; Tempelman, H. A.; Grobbee, D. E.; Scheuermaier, K.; Venter, W. D. F.; Klipstein-Grobusch, K. | 2022 | | Comparative performance of cardiovascular risk prediction models in people living with HIV | | unrelated topic |
| 1. Nguyen, V. Q.; Sreewongchai, T.; Siangliw, M.; Roytrakul, S.; Yokthongwattana, C. | 2022 | | Comparative proteomic analysis of chromosome segment substitution lines of Thai jasmine rice KDML105 under short-term salinity stress | | unrelated topic |
| 1. Herrera, M.; Fernández, J.; Vargas, M.; Villalta, M.; Segura, Á; León, G.; Angulo, Y.; Paiva, O.; Matainaho, T.; Jensen, S. D.; Winkel, K. D.; Calvete, J. J.; Williams, D. J.; Gutiérrez, J. M. | 2012 | | Comparative proteomic analysis of the venom of the taipan snake, Oxyuranus scutellatus, from Papua New Guinea and Australia: role of neurotoxic and procoagulant effects in venom toxicity | | unrelated topic |
| 1. Onuigbo, W. I. | 2001 | | Comparative study of adult polycystic kidney disease | | unrelated topic |
| 1. Shija, J. K. | 1981 | | Comparative study of hospital incidences of congenital anomalies, childhood malignant solid tumours and pyomyositis in mainland Tanzania | | Year of publication |
| 1. Olasoji, H. O.; Odusanya, S. A. | 2000 | | Comparative study of third molar impaction in rural and urban areas of South-Western Nigeria | | unrelated topic |
| 1. Jacobs, P. C.; Prokop, M.; van der Graaf, Y.; Gondrie, M. J.; Janssen, K. J.; de Koning, H. J.; Isgum, I.; van Klaveren, R. J.; Oudkerk, M.; van Ginneken, B.; Mali, W. P. | 2010 | | Comparing coronary artery calcium and thoracic aorta calcium for prediction of all-cause mortality and cardiovascular events on low-dose non-gated computed tomography in a high-risk population of heavy smokers | | unrelated topic |
| 1. Egbunah, U. P.; Adamson, O.; Fashina, A.; Adekunle, A. A.; James, O.; Adeyemo, W. L. | 2022 | | Comparing the Treatment Outcomes of Absorbable Sutures, Nonabsorbable Sutures, and Tissue Adhesives in Cleft Lip Repair: A Systematic Review | | unrelated topic |
| 1. Theron, A. P.; Brisighelli, G.; Theron, A. E.; Leva, E.; Numanoglu, A. | 2015 | | Comparison in the incidence of anorectal malformations between a first- and third-world referral center | | unrelated topic |
| 1. Tiam, A.; Kassaye, S. G.; Machekano, R.; Tukei, V.; Gill, M. M.; Mokone, M.; Letsie, M.; Tsietso, M.; Seipati, I.; Barasa, J.; Isavwa, A.; Tylleskär, T.; Guay, L. | 2019 | | Comparison of 6-week PMTCT outcomes for HIV-exposed and HIV-unexposed infants in the era of lifelong ART: Results from an observational prospective cohort study | | unrelated topic |
| 1. Arena, P. J.; Dzogang, C.; Gadoth, A.; Nkamba, D. M.; Hoff, N. A.; Kampilu, D.; Beia, M.; Wong, H. L.; Anderson, S. A.; Kaba, D.; Rimoin, A. W. | 2023 | | Comparison of adverse pregnancy and birth outcomes using archival medical records before and during the first wave of the COVID-19 pandemic in Kinshasa, Democratic Republic of Congo: a facility-based, retrospective cohort study | | Duplicate |
| 1. Arena, P. J.; Dzogang, C.; Gadoth, A.; Nkamba, D. M.; Hoff, N. A.; Kampilu, D.; Beia, M.; Wong, H. L.; Anderson, S. A.; Kaba, D.; Rimoin, A. W. | 2023 | | Comparison of adverse pregnancy and birth outcomes using archival medical records before and during the first wave of the COVID-19 pandemic in Kinshasa, Democratic Republic of Congo: a facility-based, retrospective cohort study | | unrelated topic |
| 1. Kassim, A.; Pflüger, V.; Premji, Z.; Daubenberger, C.; Revathi, G. | 2017 | | Comparison of biomarker based Matrix Assisted Laser Desorption Ionization-Time of Flight Mass Spectrometry (MALDI-TOF MS) and conventional methods in the identification of clinically relevant bacteria and yeast | | unrelated topic |
| 1. Kiyak, H.; Gezer, S.; Ozdemir, C.; Gunkaya, S.; Karacan, T.; Gedikbasi, A. | 2020 | | Comparison of delivery characteristics and early obstetric outcomes between Turkish women and Syrian refugee pregnancies | | unrelated topic |
| 1. McInnes, P. M.; Richardson, B. D.; Cleaton-Jones, P. E. | 1982 | | Comparison of dental fluorosis and caries in primary teeth of preschool-children living in arid high and low fluoride villages | | Year of publication |
| 1. Warf, B. C. | 2005 | | Comparison of endoscopic third ventriculostomy alone and combined with choroid plexus cauterization in infants younger than 1 year of age: a prospective study in 550 African children | | unrelated topic |
| 1. Ayeni, F. A.; Gbarabon, T.; Andersen, C.; Norskov-Lauritsen, N. | 2015 | | Comparison of identification and antimicrobial resistance pattern of Staphylococcus aureus isolated from Amassoma, Bayelsa state, Nigeria | | unrelated topic |
| 1. Alighieri, C.; Bettens, K.; Bruneel, L.; Sseremba, D.; Musasizi, D.; Ojok, I.; Van Lierde, K. | 2020 | | Comparison of motor-phonetic versus phonetic-phonological speech therapy approaches in patients with a cleft (lip and) palate: a study in Uganda | | Duplicate |
| 1. Alighieri, C.; Bettens, K.; Bruneel, L.; Sseremba, D.; Musasizi, D.; Ojok, I.; Van Lierde, K. | 2020 | | Comparison of motor-phonetic versus phonetic-phonological speech therapy approaches in patients with a cleft (lip and) palate: a study in Uganda | | unrelated topic |
| 1. Denayer, N.; Troost, E.; Santens, B.; De Meester, P.; Roggen, L.; Moons, P.; Van Calsteren, K.; Budts, W.; Van De Bruaene, A. | 2021 | | Comparison of risk stratification models for pregnancy in congenital heart disease | | unrelated topic |
| 1. Bruzoni, M.; Sudan, D. L.; Cusick, R. A.; Thompson, J. S. | 2008 | | Comparison of short bowel syndrome acquired early in life and during adolescence | | unrelated topic |
| 1. Kale, B.; Buyukcavus, M. H.; Esenlik, E. | 2018 | | Comparison of the change in inferior sclera exposure after maxillary protraction with or without skeletal anchorage | | unrelated topic |
| 1. Cleaton-Jones, P.; Hargreaves, J. A. | 1990 | | Comparison of three fluorosis indices in a Namibian community with twice optimum fluoride in the drinking water | | Year of publication |
| 1. Burger, P.; Cleaton-Jones, P.; du Plessis, J.; de Vries, J. | 1987 | | Comparison of two fluorosis indices in the primary dentition of Tswana children | | Year of publication |
| 1. Mabelya, L.; van 't Hof, M. A.; König, K. G.; van Palenstein Helderman, W. H. | 1994 | | Comparison of two indices of dental fluorosis in low, moderate and high fluorosis Tanzanian populations | | Year of publication |
| 1. Noronha, L. E.; Wilson, W. C. | 2017 | | Comparison of two zoonotic viruses from the order Bunyavirales | | unrelated topic |
| 1. Chinawa, A. T.; Chinawa, J. M. | 2021 | | Compendium of cardiac diseases among children presenting in tertiary institutions in southern Nigeria: a rising trend | | Included |
| 1. Yuko-Jowi, C. A.; Okello, C. A.; Mutai, L. | 2013 | | COMPLEMENTARY TECHNIQUES OF PERCUTANEOUS CLOSURE OF DUCTUS ARTERIOSUS USING DETACHABLE COOK COILS AND AMPLATZER DEVICES | | unrelated topic |
| 1. Ujunwa, F. A.; Ujuanbi, I. S.; Chinawa, J. M. | 2021 | | Complex congenital heart diseases among children presenting for cardiac surgery in a tertiary health facility in Enugu; South-East Nigeria. A rising trend | | unrelated topic |
| 1. Appeadu-Mensah, W.; Hesse, A. A.; Glover-Addy, H.; Osei-Nketiah, S.; Etwire, V.; Sarpong, P. A. | 2015 | | Complications of hypospadias surgery: Experience in a tertiary hospital of a developing country | | unrelated topic |
| 1. Venter, P.; Causon, T.; Pasch, H.; de Villiers, A. | 2019 | | Comprehensive analysis of chestnut tannins by reversed phase and hydrophilic interaction chromatography coupled to ion mobility and high resolution mass spectrometry | | unrelated topic |
| 1. Gungor, O. E.; Nur, B. G.; Yalcin, H.; Karayilmaz, H.; Mihci, E. | 2015 | | Comprehensive dental management in a Hallermann-Streiff syndrome patient with unusual radiographic appearance of teeth | | unrelated topic |
| 1. Amaraegbulam, P. I.; Oluwatosin, U. I.; Udemezue, C. O.; Egbe-Eni, U.; Chuku, A. | 2021 | | Comprehensive Idiopathic Clubfoot Treatment based on the Ponseti Method: The FMC, Umuahia Experience | | unrelated topic |
| 1. Totten, S. M.; Zivkovic, A. M.; Wu, S.; Ngyuen, U.; Freeman, S. L.; Ruhaak, L. R.; Darboe, M. K.; German, J. B.; Prentice, A. M.; Lebrilla, C. B. | 2012 | | Comprehensive profiles of human milk oligosaccharides yield highly sensitive and specific markers for determining secretor status in lactating mothers | | unrelated topic |
| 1. Onwuchekwa, C. R.; Alazigha, N. S. | 2017 | | Computed tomography pattern of traumatic head injury in Niger Delta, Nigeria: A multicenter evaluation | | unrelated topic |
| 1. Zühlke, L | 2011 | | Computer-assisted auscultation as a screening tool for cardiovascular disease: a cross-sectional study | | book |
| 1. Lingenhel, A.; Kraft, H. G.; Kotze, M.; Peeters, A. V.; Kronenberg, F.; Kruse, R.; Utermann, G. | 1998 | | Concentrations of the atherogenic Lp(a) are elevated in FH | | Year of publication |
| 1. Robinson, L.; Sengoatsi, T.; van Heerden, W. F. P. | 2021 | | Concomitant Congenital Intraoral Dermoid Cyst and Heterotopic Gastrointestinal Cyst | | unrelated topic |
| 1. Ting, W.; Richard, S. A.; Changwei, Z.; Chaohua, W.; Xiaodong, X. | 2019 | | Concomitant occurrence of clinoid and cavernous segment aneurysms complicated with carotid cavernous fistula: A case report | | unrelated topic |
| 1. Njoh, J.; Chellaram, R.; Ramas, L. | 1991 | | Congenital abnormalities in Liberian neonates | | Year of publication |
| 1. Abdurrahman, M. B.; Garg, S. K.; Mabogunje, O. A.; Yakubu, A. M.; Momoh, J. T.; Lawrie, J. H. | 1982 | | Congenital abnormalities of the genitourinary system in northern Nigeria | | Year of publication |
| 1. Mai, M.; Wal, A.; Dryden, R. | 1986 | | Congenital abnormalities recorded at Port Moresby General Hospital, 1980-1984 | | Duplicate |
| 1. Mai, M.; Wal, A.; Dryden, R. | 1986 | | Congenital abnormalities recorded at Port Moresby General Hospital, 1980-1984 | | Year of publication |
| 1. Singh, B.; Satyapal, K. S.; Moodley, J.; Haffejee, A. A. | 1999 | | Congenital absence of the gall bladder | | Year of publication |
| 1. Chait, L. A. | 1979 | | Congenital absence of the hand | | Year of publication |
| 1. Geefhuysen, J.; Hall, D. M.; Wolfsdorf, J. | 1976 | | Congenital adrenal hyperplasia in Blacks | | Year of publication |
| 1. Madrid, L.; Varo, R.; Sitoe, A.; Bassat, Q. | 2016 | | Congenital and perinatally-acquired infections in resource-constrained settings | | unrelated topic |
| 1. Mekonnen, D.; MollaTaye; Worku, W. | 2021 | | Congenital anomalies among newborn babies in Felege-Hiwot Comprehensive Specialized Referral Hospital, Bahir Dar, Ethiopia | | doesn’t include primary outcome |
| 1. Gedamu, S.; Sendo, E. G.; Daba, W. | 2021 | | Congenital Anomalies and Associated Factors among Newborns in Bishoftu General Hospital, Oromia, Ethiopia: A Retrospective Study | | Duplicate |
| 1. Gedamu, S.; Sendo, E. G.; Daba, W. | 2021 | | Congenital Anomalies and Associated Factors among Newborns in Bishoftu General Hospital, Oromia, Ethiopia: A Retrospective Study | | unrelated topic |
| 1. Moges, N.; Anley, D. T.; Zemene, M. A.; Adella, G. A.; Solomon, Y.; Bantie, B.; Fenta Felek, S.; Dejenie, T. A.; Bayih, W. A.; Chanie, E. S.; Getaneh, F. B.; Kassaw, A.; Mengist Dessie, A. | 2023 | | Congenital anomalies and risk factors in Africa: a systematic review and meta-analysis | | unrelated topic |
| 1. Delport, S. D.; Christianson, A. L.; van den Berg, H. J.; Wolmarans, L.; Gericke, G. S. | 1995 | | Congenital anomalies in black South African liveborn neonates at an urban academic hospital | | Year of publication |
| 1. Birhanu, K.; Tesfaye, W.; Berhane, M. | 2021 | | Congenital Anomalies in Neonates Admitted to a Tertiary Hospital in Southwest Ethiopia: A Cross Sectional Study | | study population |
| 1. Venter, P. A.; Christianson, A. L.; Hutamo, C. M.; Makhura, M. P.; Gericke, G. S. | 1995 | | Congenital anomalies in rural black South African neonates–a silent epidemic? | | Year of publication |
| 1. Postoev, V. A.; Grjibovski, A. M.; Kovalenko, A. A.; Anda, E. E.; Nieboer, E.; Odland, JØ | 2016 | | Congenital anomalies of the kidney and the urinary tract: A murmansk county birth registry study | | unrelated topic |
| 1. Wong, L. M.; Cheruiyot, I.; de Oliveira, M. H. S.; Keet, K.; Tomaszewski, K. A.; Walocha, J. A.; Tubbs, S.; Henry, B. M. | 2021 | | Congenital Anomalies of the Tracheobronchial Tree: A Meta-Analysis and Clinical Considerations | | unrelated topic |
| 1. Taye, M.; Afework, M.; Fantaye, W.; Diro, E.; Worku, A. | 2019 | | Congenital anomalies prevalence in Addis Ababa and the Amhara region, Ethiopia: a descriptive cross-sectional study | | Duplicate |
| 1. Taye, M.; Afework, M.; Fantaye, W.; Diro, E.; Worku, A. | 2019 | | Congenital anomalies prevalence in Addis Ababa and the Amhara region, Ethiopia: a descriptive cross-sectional study | | Duplicate |
| 1. Bell, D. | 1976 | | Congenital aural and pre-auricular sinuses in the South African Bantu | | Year of publication |
| 1. Zernotti, M. E.; Curet, C. A.; Cortasa, S.; Chiaraviglio, M.; Di Gregorio, M. F. | 2019 | | Congenital Aural Atresia prevalence in the Argentinian population | | unrelated topic |
| 1. Hunter, L. D.; Lloyd, G. W.; Monaghan, M. J.; Pecoraro, A. J. K.; Doubell, A. F.; Herbst, P. G. | 2020 | | Congenital bicuspid aortic valve: Differential prevalence across different South African population groups | | unrelated topic |
| 1. Della-Porta, A. J.; Murray, M. D.; Cybinski, D. H. | 1976 | | Congenital bovine epizootic arthrogryposis and hydranencephaly in Australia. Distribution of antibodies to Akabane virus in Australian Cattle after the 1974 epizootic | | Year of publication |
| 1. Muntha, A.; Moges, T. | 2019 | | Congenital Cardiovascular Anomalies among Cases of Down Syndrome: A Hospital Based Review of Cases in TikurAnbessa Specialized Hospital, Ethiopia | | unrelated topic |
| 1. Odelowo, E. O. | 1989 | | Congenital chest wall malformations in Nigerians | | Year of publication |
| 1. Idowu, O. E.; Ayodele, O. A.; Oshola, H. A. | 2019 | | Congenital Cirsoid aneurysm communicating with the sagittal sinus and supplied by extra and intracranial arteries | | unrelated topic |
| 1. Datubo-Brown, D. D.; Kejeh, B. M. | 1989 | | Congenital cleft deformities in Rivers state of Nigeria: is there any association with environmental pollution? | | Year of publication |
| 1. Oluwasanmi, J. O.; Adekunle, O. O. | 1970 | | Congenital clefts of the face in Nigeria | | Year of publication |
| 1. Adewole, O. A.; Giwa, S. O.; Kayode, M. O.; Shoga, M. O.; Balogun, R. A. | 2009 | | Congenital club foot in a teaching hospital in Lagos, Nigeria | | unrelated topic |
| 1. Okeke, L. I.; Aisuodionoe-Shadrach, O. I.; Adekanye, A. O. | 2005 | | Congenital complex penile curvature | | unrelated topic |
| 1. Last, R. D.; Hill, J. M.; Roach, M.; Kaldenberg, T. | 2006 | | Congenital dilatation of the large and segmental intrahepatic bile ducts (Caroli's disease) in two Golden retriever littermates | | unrelated topic |
| 1. Pompe van Meerdervoort, H. F. | 1974 | | Congenital dislocation of the hip in black patients | | Year of publication |
| 1. Famuyiwa, M. K. | 2020 | | Congenital disorders and community genetic services in Nigeria: A systematic review | | unrelated topic |
| 1. Chindia, M. L.; Awange, D. O. | 1994 | | Congenital epulis of the newborn: a report of two cases | | Year of publication |
| 1. Lawan, A. | 2008 | | Congenital eye and adnexial anomalies in Kano, a five year review | | unrelated topic |
| 1. Chuka-Okosa, C. M.; Magulike, N. O.; Onyekonwu, G. C. | 2005 | | Congenital eye anomalies in Enugu, South-Eastern Nigeria | | unrelated topic |
| 1. Bodunde, O. T.; Ajibode, H. A. | 2006 | | Congenital eye diseases at Olabisi Onabanjo University Teaching Hospital, Sagamu, Nigeria | | unrelated topic |
| 1. Spencer, K.; Mokhele, I.; Firnhaber, C. | 2018 | | Congenital genital abnormalities detected during routine circumcision at a South African institution: a retrospective record review | | unrelated topic |
| 1. Talargia, F and Seyoum, Girma and Moges, T | 2018 | | Congenital heart defects and associated factors in children with congenital anomalies | | Included |
| 1. Ekure, E. N.; Bode-Thomas, F.; Sadoh, W. E.; Orogade, A. A.; Otaigbe, B. E.; Ujunwa, F.; Sani, U. M.; Asani, M.; Animasahun, A. B.; Ogunkunle, O. O. | 2017 | | Congenital Heart Defects in Nigerian Children: Preliminary Data From the National Pediatric Cardiac Registry | | Duplicate |
| 1. Ekure, E. N.; Bode-Thomas, F.; Sadoh, W. E.; Orogade, A. A.; Otaigbe, B. E.; Ujunwa, F.; Sani, U. M.; Asani, M.; Animasahun, A. B.; Ogunkunle, O. O. | 2017 | | Congenital Heart Defects in Nigerian Children: Preliminary Data From the National Pediatric Cardiac Registry | | Included |
| 1. Erinoso, O. A.; James, O.; Sokunbi, O. J.; Adamson, O. O.; Adekunle, A. A.; Agbogidi, O. F.; Ogunlewe, A. O.; Ekure, E. N.; Adeyemo, W. L.; Ladeinde, A. L.; Ogunlewe, O. M. | 2021 | | Congenital heart defects in orofacial cleft: A prospective cohort study | | unrelated topic |
| 1. Benhaourech, S.; Drighil, A.; Hammiri, A. E. | 2016 | | Congenital heart disease and Down syndrome: various aspects of a confirmed association | | unrelated topic |
| 1. Zühlke, L.; Mirabel, M.; Marijon, E. | 2013 | | Congenital heart disease and rheumatic heart disease in Africa: recent advances and current priorities | | Duplicate |
| 1. Zühlke, L.; Mirabel, M.; Marijon, E. | 2013 | | Congenital heart disease and rheumatic heart disease in Africa: recent advances and current priorities | | unrelated topic |
| 1. L Zühlke, M Mirabel, E Marijon | 2013 | | Congenital heart disease and rheumatic heart disease in Africa: recent advances and current priorities | | unrelated topic |
| 1. Wood, J. B.; Serumaga, J.; Lewis, M. G. | 1969 | | Congenital heart disease at necropsy in Uganda. A 16-years survey at Mulago Hospital, Kampala | | Year of publication |
| 1. Jivanji, S. G. M.; Lubega, S.; Reel, B.; Qureshi, S. A. | 2019 | | Congenital Heart Disease in East Africa | | doesn’t include primary outcome |
| 1. Zühlke, L.; Lawrenson, J.; Comitis, G.; De Decker, R.; Brooks, A.; Fourie, B.; Swanson, L.; Hugo-Hamman, C. | 2019 | | Congenital Heart Disease in Low- and Lower-Middle-Income Countries: Current Status and New Opportunities | | Duplicate |
| 1. Zühlke, L.; Lawrenson, J.; Comitis, G.; De Decker, R.; Brooks, A.; Fourie, B.; Swanson, L.; Hugo-Hamman, C. | 2019 | | Congenital Heart Disease in Low- and Lower-Middle-Income Countries: Current Status and New Opportunities | | unrelated |
| 1. Zimmerman, M.; Sable, C. | 2020 | | Congenital heart disease in low-and-middle-income countries: Focus on sub-Saharan Africa | | Duplicate |
| 1. Zimmerman, M.; Sable, C. | 2020 | | Congenital heart disease in low-and-middle-income countries: Focus on sub-Saharan Africa | | study design |
| 1. Antia, A. U. | 1974 | | Congenital heart disease in Nigeria. Clinical and necropsy study of 260 cases | | Year of publication |
| 1. Jaiyesimi, F.; Antia, A. U. | 1981 | | Congenital heart disease in Nigeria: a ten-year experience at UCH, Ibadan | | Year of publication |
| 1. Sadoh, W. E.; Uzodimma, C. C.; Daniels, Q. | 2013 | | Congenital heart disease in Nigerian children: a multicenter echocardiographic study | | Included |
| 1. De Buse, P. | 1974 | | Congenital heart disease in Papu New Guinean children | | Year of publication |
| 1. Tefuarani, N.; Hawker, R.; Vince, J.; Sleigh, A.; Williams, G. | 2001 | | Congenital heart disease in Papua New Guinean children | | outside geographic scope |
| 1. Ekure, E. N.; Sokunbi, O.; Kalu, N.; Olusegun-Joseph, A.; Kushimo, O.; Amadi, C.; Hassan, O.; Ikebudu, D.; Onyia, S.; Onwudiwe, C.; Nwankwo, V.; Akinwunmi, R.; Awusa, F.; Akere, Z.; Dele-Salawu, O.; Ajayi, E.; Ale, O.; Muoneke, D.; Muenke, M.; Kruszka, P.; Beaton, A.; Sable, C.; Adeyemo, A. | 2020 | | Congenital heart disease in school children in Lagos, Nigeria: Prevalence and the diagnostic gap | | Duplicate |
| 1. Ekure, E. N.; Sokunbi, O.; Kalu, N.; Olusegun-Joseph, A.; Kushimo, O.; Amadi, C.; Hassan, O.; Ikebudu, D.; Onyia, S.; Onwudiwe, C.; Nwankwo, V.; Akinwunmi, R.; Awusa, F.; Akere, Z.; Dele-Salawu, O.; Ajayi, E.; Ale, O.; Muoneke, D.; Muenke, M.; Kruszka, P.; Beaton, A.; Sable, C.; Adeyemo, A. | 2020 | | Congenital heart disease in school children in Lagos, Nigeria: Prevalence and the diagnostic gap | | source and study population |
| 1. Thorne, SA | 2016 | | Congenital heart disease in the adult | | unrelated topic |
| 1. van der Horst, R. L.; Wainwright, J. | 1969 | | Congenital heart disease in the Bantu: an autopsy analysis of 123 cases | | Year of publication |
| 1. Otaigbe, B. E.; Tabansi, P. N. | 2014 | | Congenital heart disease in the Niger Delta region of Nigeria: a four-year prospective echocardiographic analysis | | Included |
| 1. Van der Horst, R. L.; Winship, W. S.; Pittaway, D.; Gibb, B. H.; Lapinsky, G. B. | 1968 | | Congenital heart disease in the South African Bantu: a report of 117 cases | | Year of publication |
| 1. Caddell, J. L.; Connor, D. H. | 1966 | | Congenital heart disease in Ugandan children | | Year of publication |
| 1. Bannerman, C. H.; Mahalu, W. | 1998 | | Congenital heart disease in Zimbabwean children | | Included |
| 1. Zakaria AMZ, Souley K, Mahamat KY, Ali BS, Yousra H, Koché T, et al | 2021 | | Congenital heart disease: Epidemiological and echocardiographic aspects of 167 cases at the Teaching Hospital of Lamorde / Niamey / Niger. | | Included |
| 1. Muenke, M; Kruszka, PS; Sable, CA; Belmont, JW | 2015 | | Congenital heart disease: molecular genetics, principles of diagnosis and treatment | | book |
| 1. Ekure, E. N.; Animashaun, A.; Bastos, M.; Ezeaka, V. C. | 2009 | | Congenital heart diseases associated with identified syndromes and other extra-cardiac congenital malformations in children in Lagos | | Included |
| 1. Ejim, E.; Anisiuba, B.; Oguanobi, N.; Ubani-Ukoma, B.; Nwaneli, U.; Ugwu, C.; Ike, S. | 2014 | | Congenital heart diseases in adults: a review of echocardiogram records in enugu, South-East Nigeria | | age scope |
| 1. Gikonyo, B. M. | 1991 | | Congenital heart diseases in east Africa | | Year of publication |
| 1. Tefuarani, N.; Sleigh, A.; Hawker, R. | 2002 | | Congenital heart diseases–a future burden for Papua New Guinea | | outside geographic scope |
| 1. Kruszka, P; Berger, S; Hong, SK; Tanpaiboon, P; ... | 2018 | | Congenital Heart Malformations in Sub-Saharan Africa and Asia | | book |
| 1. Kruszka, P; Berger, S; Hong, SK; Tanpaiboon, P; ... | 2018 | | Congenital Heart Malformations in Sub-Saharan Africa and Asia | | Duplicate |
| 1. Van der Horst, R. L.; Winship, W. S.; Gotsman, M. S. | 1970 | | Congenital heart malformations in the South African Indian | | Year of publication |
| 1. Khan, N.; Qazi, S. A. | 1997 | | Congenital hematometrocolpos in a circumcised girl. An anomaly superimposed by cultural mutilating practices | | Duplicate |
| 1. Khan, N.; Qazi, S. A. | 1997 | | Congenital hematometrocolpos in a circumcised girl. An anomaly superimposed by cultural mutilating practices | | Year of publication |
| 1. Cywes, S.; Davies, M. R.; Rode, H. | 1980 | | Congenital jejuno-ileal atresia and stenosis | | Year of publication |
| 1. Chindia, M. L.; Kimaro, S. | 1994 | | Congenital lower lip pits and bilateral clefting of the upper lip | | Year of publication |
| 1. Zar, H.; McIvor, B.; Furlan, G.; Jedeikin, L.; Pitcher, R. | 2006 | | Congenital lung mass in an asymptomatic patient | | unrelated topic |
| 1. Obu, H. A.; Chinawa, J. M.; Uleanya, N. D.; Adimora, G. N.; Obi, I. E. | 2012 | | Congenital malformations among newborns admitted in the neonatal unit of a tertiary hospital in Enugu, South-East Nigeria–a retrospective study | | study setting |
| 1. Nagalo, K.; Ouédraogo, I.; Laberge, J. M.; Caouette-Laberge, L.; Turgeon, J. | 2017 | | Congenital malformations and medical conditions associated with orofacial clefts in children in Burkina Faso | | unrelated topic |
| 1. Khan, A. A. | 1965 | | Congenital malformations in African neonates in Nairobi | | Year of publication |
| 1. Tessema, T.; Abuohay, M. | 1995 | | Congenital malformations in Gondar Hospital, Ethiopia | | Year of publication |
| 1. Cavaliere, E.; Trevisanuto, D.; Da Dalt, L.; Putoto, G.; Pizzol, D.; Muhelo, A. R.; Cavallin, F. | 2022 | | Congenital malformations in neonates admitted to a neonatal intensive care unit in a low-resource setting | | outside geographic scope |
| 1. Sunday-Adeoye, I.; Okonta, P. I.; Egwuatu, V. E. | 2007 | | Congenital malformations in singleton and twin births in rural Nigeria | | not accessible |
| 1. Ahuka, O. L.; Toko, R. M.; Omanga, F. U.; Tshimpanga, B. J. | 2006 | | Congenital malformations in the North-Eastern Democratic Republic of Congo during Civil War | | did not report outcome of interest |
| 1. Khan, A. A.; Ivanov, I. | 1977 | | Congenital malformations in Zambian neonates | | Year of publication |
| 1. Peter, J. C.; Fieggen, G. | 1999 | | Congenital malformations of the brain–a neurosurgical perspective at the close of the twentieth century | | Year of publication |
| 1. Binitie, O. P. | 1992 | | Congenital malformations of the central nervous system at the Jos University Teaching Hospital, Jos Plateau State of Nigeria | | Year of publication |
| 1. Ohaegbulam, S. C.; Saddeqi, N. | 1979 | | Congenital malformations of the central nervous system in Enugu, Nigeria | | Year of publication |
| 1. Adeloye, A.; Odeku, E. L. | 1972 | | Congenital malformations of the central nervous system in Nigeria | | Year of publication |
| 1. Odeku, E. L.; Grant, I. H.; Ekop, A. C. | 1967 | | Congenital malformations of the cerebrospinal axis seen in Western Nigeria. The spinal meningoceles | | Year of publication |
| 1. Camara, S.; Fall, M.; Mbaye, P. A.; Wese, S. F.; Lo, F. B.; Oumar, N. | 2022 | | Congenital malformations of the gastrointestinal tract in neonates at aristide le dantec university hospital in Dakar: Concerning 126 cases | | unrelated topic |
| 1. Stevenson, A. C.; Johnston, H. A.; Stewart, M. I.; Golding, D. R. | 1966 | | Congenital malformations. A report of a study of series of consecutive births in 24 centres | | Year of publication |
| 1. Akang, E. E.; Osinusi, K. O.; Pindiga, H. U.; Okpala, J. U.; Aghadiuno, P. U. | 1993 | | Congenital malformations: a review of 672 autopsies in Ibadan, Nigeria | | Year of publication |
| 1. DeSilva, M.; Munoz, F. M.; Sell, E.; Marshall, H.; Tse Kawai, A.; Kachikis, A.; Heath, P.; Klein, N. P.; Oleske, J. M.; Jehan, F.; Spiegel, H.; Nesin, M.; Tagbo, B. N.; Shrestha, A.; Cutland, C. L.; Eckert, L. O.; Kochhar, S.; Bardají, A. | 2017 | | Congenital microcephaly: Case definition & guidelines for data collection, analysis, and presentation of safety data after maternal immunisation | | unrelated topic |
| 1. Pompe van Meerdervoort, H. F. | 1976 | | Congenital musculoskeletal malformation in South African Blacks: a study of incidence | | Year of publication |
| 1. Ekenze, S. O.; Nwangwu, E. I.; Ezomike, U. O. | 2019 | | Congenital obstructive bowel anomalies presenting after neonatal age | | unrelated topic |
| 1. Gordon, H.; Davies, D.; Friedberg, S. | 1969 | | Congenital pits of the lower lip with cleft lip and palate | | Year of publication |
| 1. Plotinsky, R. N.; Talbot, E. A.; Kellenberg, J. E.; Reef, S. E.; Buseman, S. K.; Wright, K. D.; Modlin, J. F. | 2007 | | Congenital rubella syndrome in a child born to Liberian refugees: clinical and public health perspectives | | unrelated topic |
| 1. Masresha, B.; Shibeshi, M.; Kaiser, R.; Luce, R.; Katsande, R.; Mihigo, R. | 2018 | | Congenital Rubella Syndrome in The African Region - Data from Sentinel Surveillance | | unrelated topic |
| 1. Motaze, N. V.; Manamela, J.; Smit, S.; Rabie, H.; Harper, K.; duPlessis, N.; Reubenson, G.; Coetzee, M.; Ballot, D.; Moore, D.; Nuttall, J.; Linley, L.; Tooke, L.; Kriel, J.; Hallbauer, U.; Sutton, C.; Moodley, P.; Hardie, D.; Mazanderani, A. H.; Goosen, F.; Kyaw, T.; Leroux, D.; Hussain, A.; Singh, R.; Kelly, C.; Ducasse, G.; Muller, M.; Blaauw, M.; Hamese, M.; Leeuw, T.; Mekgoe, O.; Rakgole, P.; Dungwa, N.; Maphosa, T.; Sanyane, K.; Preiser, W.; Cohen, C.; Suchard, M. | 2019 | | Congenital Rubella Syndrome Surveillance in South Africa Using a Sentinel Site Approach: A Cross-sectional Study | | unrelated topic |
| 1. Martínez-Quintana, E.; Castillo-Solórzano, C.; Torner, N.; Rodríguez-González, F. | 2015 | | Congenital rubella syndrome: a matter of concern | | unrelated topic |
| 1. Staley, G. P.; van der Lugt, J. J.; Axsel, G.; Loock, A. H. | 1994 | | Congenital skeletal malformations in Holstein calves associated with putative manganese deficiency | | Year of publication |
| 1. Olusanya, A. A.; Akadiri, O. A. | 2020 | | Congenital syngnathia: review of clinical profile and proposal of a new classification-based management protocol | | unrelated topic |
| 1. Boot, J. M.; Oranje, A. P.; de Groot, R.; Tan, G.; Stolz, E. | 1992 | | Congenital syphilis | | Year of publication |
| 1. Stephens, M. M. | 1990 | | Congenital talipes equinovarus | | Year of publication |
| 1. Culverwell, A. D.; Tapping, C. R. | 2009 | | Congenital talipes equinovarus in Papua New Guinea: a difficult yet potentially manageable situation | | unrelated topic |
| 1. Shahin, S.; Hoffman, T.; van Es, W.; Grutters, J.; Mateyo, K. | 2021 | | Congenital tracheobronchomegaly (Mounier-Kuhn syndrome) in a 28-year-old Zambian male: a case report | | bookSection |
| 1. Wise, J. | 2011 | | Conjoined twins are separated in four stage operation | | unrelated topic |
| 1. Nath, S.; Munkonge, L. | 1996 | | Conjoined twins in Zambia | | Year of publication |
| 1. Thomas, J. M.; Lopez, J. T. | 2004 | | Conjoined twins–the anaesthetic management of 15 sets from 1991-2002 | | unrelated topic |
| 1. Cywes, S.; Millar, A. J.; Rode, H.; Brown, R. A. | 1997 | | Conjoined twins–the Cape Town experience | | Year of publication |
| 1. Edwin, F. | 2016 | | Conotruncal Heart Defect Repair in Sub-Saharan Africa: Remarkable Outcomes Despite Poor Access to Treatment | | book |
| 1. Edwin, F.; Entsua-Mensah, K.; Sereboe, L. A.; Tettey, M. M.; Aniteye, E. A.; Tamatey, M. M.; Adzamli, I.; Akyaa-Yao, N.; Gyan, K. B.; Ofosu-Appiah, E.; Kotei, D. | 2016 | | Conotruncal Heart Defect Repair in Sub-Saharan Africa: Remarkable Outcomes Despite Poor Access to Treatment | | unrelated topic |
| 1. Nelson, J.; Smith, M.; Bittles, A. H. | 1997 | | Consanguineous marriage and its clinical consequences in migrants to Australia | | Year of publication |
| 1. Ghosh, T. S.; Kwawukume, E. Y. | 1994 | | Construction of an artificial vagina with sigmoid colon in vaginal agenesis | | Year of publication |
| 1. BM Mayosi | 2007 | | Contemporary trends in the epidemiology and management of cardiomyopathy and pericarditis in sub-Saharan Africa | | unrelated topic |
| 1. Sibeko, S.; Baxter, C.; Yende, N.; Karim, Q. A.; Karim, S. S. | 2011 | | Contraceptive choices, pregnancy rates, and outcomes in a microbicide trial | | unrelated topic |
| 1. Abudu, O. O.; Uguru, V.; Olude, O. | 1988 | | Contribution of congenital malformation to perinatal mortality in Lagos, Nigeria | | Year of publication |
| 1. Guneren, E.; Ture, N.; Karabekmez, F. E.; Zahir, K. | 2019 | | Contribution of the Use of a Pen-Type Endoscope to Evaluate and Treat Ears Simultaneously During Cleft Palate Surgery in Late Presented Cases | | unrelated topic |
| 1. Kaseva, M. E. | 2006 | | Contribution of trona (magadi) into excessive fluorosis–a case study in Maji ya Chai ward, northern Tanzania | | Duplicate |
| 1. Kaseva, M. E. | 2006 | | Contribution of trona (magadi) into excessive fluorosis–a case study in Maji ya Chai ward, northern Tanzania | | unrelated topic |
| 1. Slater, P. E.; Roitman, M.; Leventhal, A.; Anis, E. | 1996 | | Control of rubella in Israel: progress and challenge | | Year of publication |
| 1. Al-Mustapha, A. I.; Alada, S. A.; Raufu, I. A.; Lawal, A. N.; Eskola, K.; Brouwer, M. S.; Adetunji, V.; Heikinheimo, A. | 2022 | | Co-occurrence of antibiotic and disinfectant resistance genes in extensively drug-resistant Escherichia coli isolated from broilers in Ilorin, North Central Nigeria | | unrelated topic |
| 1. Gowans, L. J. J.; Al Dhaheri, N.; Li, M.; Busch, T.; Obiri-Yeboah, S.; Oti, A. A.; Sabbah, D. K.; Arthur, F. K. N.; Awotoye, W. O.; Alade, A. A.; Twumasi, P.; Agbenorku, P.; Plange-Rhule, G.; Naicker, T.; Donkor, P.; Murray, J. C.; Sobreira, N. L. M.; Butali, A. | 2021 | | Co-occurrence of orofacial clefts and clubfoot phenotypes in a sub-Saharan African cohort: Whole-exome sequencing implicates multiple syndromes and genes | | unrelated topic |
| 1. Hlongwa, P.; Rispel, L. C. | 2021 | | Coproduction in the management of individuals with cleft lip and palate in South Africa: the Ekhaya Lethu model | | unrelated topic |
| 1. Szecówka, K.; Misiak, B.; Łaczmańska, I.; Frydecka, D.; Moustafa, A. A. | 2023 | | Copy Number Variations and Schizophrenia | | unrelated topic |
| 1. Cho, Y.; Rangan, G.; Logeman, C.; Ryu, H.; Sautenet, B.; Perrone, R. D.; Nadeau-Fredette, A. C.; Mustafa, R. A.; Htay, H.; Chonchol, M.; Harris, T.; Gutman, T.; Craig, J. C.; Ong, A. C. M.; Chapman, A.; Ahn, C.; Coolican, H.; Kao, J. T.; Gansevoort, R. T.; Torres, V.; Pei, Y.; Johnson, D. W.; Viecelli, A. K.; Teixeira-Pinto, A.; Howell, M.; Ju, A.; Manera, K. E.; Tong, A. | 2020 | | Core Outcome Domains for Trials in Autosomal Dominant Polycystic Kidney Disease: An International Delphi Survey | | unrelated topic |
| 1. Dowsett, L.; Porras, A. R.; Kruszka, P.; Davis, B.; Hu, T.; Honey, E.; Badoe, E.; Thong, M. K.; Leon, E.; Girisha, K. M.; Shukla, A.; Nayak, S. S.; Shotelersuk, V.; Megarbane, A.; Phadke, S.; Sirisena, N. D.; Dissanayake, V. H. W.; Ferreira, C. R.; Kisling, M. S.; Tanpaiboon, P.; Uwineza, A.; Mutesa, L.; Tekendo-Ngongang, C.; Wonkam, A.; Fieggen, K.; Batista, L. C.; Moretti-Ferreira, D.; Stevenson, R. E.; Prijoles, E. J.; Everman, D.; Clarkson, K.; Worthington, J.; Kimonis, V.; Hisama, F.; Crowe, C.; Wong, P.; Johnson, K.; Clark, R. D.; Bird, L.; Masser-Frye, D.; McDonald, M.; Willems, P.; Roeder, E.; Saitta, S.; Anyane-Yeoba, K.; Demmer, L.; Hamajima, N.; Stark, Z.; Gillies, G.; Hudgins, L.; Dave, U.; Shalev, S.; Siu, V.; Ades, A.; Dubbs, H.; Raible, S.; Kaur, M.; Salzano, E.; Jackson, L.; Deardorff, M.; Kline, A.; Summar, M.; Muenke, M.; Linguraru, M. G.; Krantz, I. D. | 2019 | | Cornelia de Lange syndrome in diverse populations | | unrelated topic |
| 1. Maru, M. | 1989 | | Coronary atherosclerosis and myocardial infarction in autopsied patients in Gondar, Ethiopia | | Year of publication |
| 1. Watkins, L. O. | 1984 | | Coronary heart disease and coronary disease risk factors in black populations in underdeveloped countries: the case for primordial prevention | | Year of publication |
| 1. Loock, M.; Steyn, K.; Becker, P.; Fourie, J. | 2006 | | Coronary heart disease and risk factors in Black South Africans: a case-control study | | unrelated topic |
| 1. Nkoke, C.; Luchuo, E. B. | 2016 | | Coronary heart disease in sub-Saharan Africa: still rare, misdiagnosed or underdiagnosed? | | unrelated topic |
| 1. Bo, A.; Zinckernagel, L.; Krasnik, A.; Petersen, J. H.; Norredam, M. | 2015 | | Coronary heart disease incidence among non-Western immigrants compared to Danish-born people: effect of country of birth, migrant status, and income | | unrelated topic |
| 1. Castle, W. M. | 1982 | | Coronary heart disease risk factors in black and white man in zimbabwe and the effect of living standards | | Year of publication |
| 1. Walker, A. R.; Sareli, P. | 1997 | | Coronary heart disease: outlook for Africa | | Year of publication |
| 1. Steyn, K.; Jooste, P. L.; Langenhoven, M. L.; Benadé, A. J.; Rossouw, J. E.; Steyn, M.; Jordaan, P. C.; Parry, C. D. | 1985 | | Coronary risk factors in the coloured population of the Cape Peninsula | | Year of publication |
| 1. Andronikou, S.; Pillay, T.; Gabuza, L.; Mahomed, N.; Naidoo, J.; Hlabangana, L. T.; du Plessis, V.; Prabhu, S. P. | 2015 | | Corpus callosum thickness in children: an MR pattern-recognition approach on the midsagittal image | | unrelated topic |
| 1. Ogamba, C. F.; Roberts, A. A.; Babah, O. A.; Ikwuegbuenyi, C. A.; Ologunja, O. J.; Amodeni, O. K. | 2021 | | Correlates of knowledge of genetic diseases and congenital anomalies among pregnant women attending antenatal clinics in Lagos, South-West Nigeria | | unrelated topic |
| 1. Ojukwu, C. P.; Anyanwu, E. G.; Nwafor, G. G. | 2017 | | Correlation between Foot Arch Index and the Intensity of Foot, Knee, and Lower Back Pain among Pregnant Women in a South-Eastern Nigerian Community | | unrelated topic |
| 1. Varma, N.; Dash, S.; Sarode, R. | 1990 | | Correlation between morphological and cytochemical heterogeneity of acute promyelocytic leukemia (APL) and its association with myelodysplasia | | Year of publication |
| 1. Velaphi, S.; Cilliers, A.; Beckh-Arnold, E.; Mokhachane, M.; Mphahlele, R.; Pettifor, J. | 2004 | | Cortical hyperostosis in an infant on prolonged prostaglandin infusion: case report and literature review | | unrelated topic |
| 1. Cardarelli, M.; Vaikunth, S.; Mills, K.; DiSessa, T.; Molloy, F.; Sauter, E.; Bowtell, K.; Rivera, R.; Shin, A. Y.; Novick, W. | 2018 | | Cost-effectiveness of Humanitarian Pediatric Cardiac Surgery Programs in Low- and Middle-Income Countries | | unrelated topic |
| 1. Abdul-Mumin, A.; Rotkis, L. N.; Gumanga, S.; Fay, E. E.; Denno, D. M. | 2022 | | Could ultrasound midwifery training increase antenatal detection of congenital anomalies in Ghana? | | unrelated topic |
| 1. Stassen, J.; De Meester, P.; Troost, E.; Roggen, L.; Moons, P.; Gewillig, M.; Van De Bruaene, A.; Budts, W. | 2021 | | Covered stent placement for treatment of coarctation of the aorta: immediate and long-term results | | Duplicate |
| 1. Stassen, J.; De Meester, P.; Troost, E.; Roggen, L.; Moons, P.; Gewillig, M.; Van De Bruaene, A.; Budts, W. | 2021 | | Covered stent placement for treatment of coarctation of the aorta: immediate and long-term results | | unrelated topic |
| 1. Raja, S. M.; Osman, M. E.; Musa, A. O.; Hussien, A. A.; Yusuf, K. | 2022 | | COVID-19 vaccine acceptance, hesitancy, and associated factors among medical students in Sudan | | unrelated topic |
| 1. Kleintjes, W. G. | 2005 | | Craniofacial abnormalities in twins at tygerberg hospital, craniofacial unit | | unrelated topic |
| 1. Odhiambo, A.; Rotich, E. C.; Chindia, M. L.; Macigo, F. G.; Ndavi, M.; Were, F. | 2012 | | Craniofacial anomalies amongst births at two hospitals in Nairobi, Kenya | | unrelated topic |
| 1. Mabogunje, O. A. | 1990 | | Cranium bifidum in northern Nigeria | | Year of publication |
| 1. Ombelet, F.; Goossens, E.; Willems, R.; Annemans, L.; Budts, W.; De Backer, J.; De Groote, K.; Moniotte, S.; Van Bulck, L.; Marelli, A.; Moons, P. | 2020 | | Creating the BELgian COngenital heart disease database combining administrative and clinical data (BELCODAC): Rationale, design and methodology | | unrelated topic |
| 1. Nonoyama, T.; Nonoyama, K.; Shimazaki, Y. | 2022 | | Cross-sectional study of the factors associated with the number of teeth in middle-aged and older persons with intellectual disabilities | | unrelated topic |
| 1. Chana, H. S.; Klauss, V. | 1988 | | Crouzon's craniofacial dysostosis in Kenya | | Year of publication |
| 1. Mofenson, L. M.; Abrams, E. J. | 2023 | | Crucial need for improved pharmacovigilance in pregnancy | | unrelated topic |
| 1. Brucker-Davis, F.; Wagner-Mahler, K.; Delattre, I.; Ducot, B.; Ferrari, P.; Bongain, A.; Kurzenne, J. Y.; Mas, J. C.; Fénichel, P. | 2008 | | Cryptorchidism at birth in Nice area (France) is associated with higher prenatal exposure to PCBs and DDE, as assessed by colostrum concentrations | | unrelated topic |
| 1. Adeoti, M. L.; Fadiora, S. O.; Oguntola, A. S.; Aderounmu, A. O.; Laosebikan, D. A.; Adejumobi, O. O. | 2004 | | Cryptorchidism in a local population in Nigeria | | unrelated topic |
| 1. Küry, S.; Mercier, S.; Shaboodien, G.; Besnard, T.; Barbarot, S.; Khumalo, N. P.; Mayosi, B. M.; Bézieau, S. | 2016 | | CUGC for hereditary fibrosing poikiloderma with tendon contractures, myopathy, and pulmonary fibrosis (POIKTMP) | | unrelated topic |
| 1. Olasoji, H. O.; Ugboko, V. I.; Arotiba, G. T. | 2007 | | Cultural and religious components in Nigerian parents' perceptions of the aetiology of cleft lip and palate: implications for treatment and rehabilitation | | Duplicate |
| 1. Olasoji, H. O.; Ugboko, V. I.; Arotiba, G. T. | 2007 | | Cultural and religious components in Nigerian parents' perceptions of the aetiology of cleft lip and palate: implications for treatment and rehabilitation | | unrelated topic |
| 1. Siegel, B.; Chan, C.; Willies-Jacobo, L.; Stein, M. T. | 2009 | | Culture clash: a missed opportunity | | unrelated topic |
| 1. James, T. | 1978 | | Curiosa paediatrica | | Year of publication |
| 1. M Twagirumukiza, D De Bacquer, JG Kips… | 2011 | | Current and projected prevalence of arterial hypertension in sub-Saharan Africa by sex, age and habitat: an estimate from population studies | | unrelated topic |
| 1. Chugh, SS; Jui, J; Gunson, K; Stecker, EC; ... | 2004 | | Current burden of sudden cardiac death: multiple source surveillance versus retrospective death certificate-based review in a large US community | | unrelated topic |
| 1. Guzman, K. J.; Gemo, N.; Martins, D. B.; Santos, P.; DeUgarte, D. A.; Ademo, F.; Kulber, D.; Issufo, C. | 2018 | | Current Challenges of Plastic Surgical Care in Sub-Saharan Africa (Maputo, Mozambique) | | unrelated topic |
| 1. Denadai, R; Lo, LJ | 2022 | | Current Concept in Cleft Surgery: Moving Toward Excellence of Outcome and Reducing the Burden of Care | | book |
| 1. Aminde, L. N.; Dzudie, A.; Takah, N. F.; Ngu, K. B.; Sliwa, K.; Kengne, A. P. | 2015 | | Current diagnostic and treatment strategies for Lutembacher syndrome: the pivotal role of echocardiography | | unrelated topic |
| 1. Fadeyibi, I. O.; Adeniyi, A. A.; Jewo, P. I.; Saalu, L. C.; Fasawe, A. A.; Ademiluyi, S. A. | 2012 | | Current pattern of cleft lip and palate deformities in Lagos, Nigeria | | unrelated topic |
| 1. Yalcouyé, A.; Esoh, K.; Guida, L.; Wonkam, A. | 2022 | | Current profile of Charcot-Marie-Tooth disease in Africa: A systematic review | | unrelated topic |
| 1. Antunes, M. J. | 1985 | | Current status of surgery for congenital heart disease in infancy | | Year of publication |
| 1. Adekanmbi, A. F.; Ogunlesi, T. A.; Olowu, A. O.; Fetuga, M. B. | 2007 | | Current trends in the prevalence and aetiology of childhood congestive cardiac failure in Sagamu | | unrelated topic |
| 1. Essop, M. R.; Essop, A. R.; Bedhesi, S.; Sareli, P. E. | 1995 | | Cyanosis and clubbing in a patient with iatrogenic Lutembacher syndrome | | Year of publication |
| 1. Worku, B. | 2003 | | Cyclopia | | unrelated topic |
| 1. Woodliff, H. J.; Swann, S. | 1978 | | Cytogenetic studies in Papua New Guinea | | Duplicate |
| 1. Woodliff, H. J.; Swann, S. | 1978 | | Cytogenetic studies in Papua New Guinea | | Year of publication |
| 1. Uwineza, A.; Hitayezu, J.; Jamar, M.; Caberg, J. H.; Murorunkwere, S.; Janvier, N.; Bours, V.; Mutesa, L. | 2016 | | Cytogenetic Studies of Rwandan Pediatric Patients Presenting with Global Developmental Delay, Intellectual Disability and/or Multiple Congenital Anomalies | | unrelated topic |
| 1. Moraka, N. O.; Moyo, S.; Mayondi, G.; Leidner, J.; Ibrahim, M.; Smith, C.; Weinberg, A.; Li, S.; Thami, P. K.; Kammerer, B.; Ajibola, G.; Musonda, R.; Shapiro, R.; Gaseitsiwe, S.; Lockman, S. | 2019 | | Cytomegalovirus Viremia in HIV-1 Subtype C Positive Women at Delivery in Botswana and Adverse Birth/Infant Health Outcomes | | Qu.licire |
| 1. Radwański, P. B.; Veeraraghavan, R.; Poelzing, S. | 2010 | | Cytosolic calcium accumulation and delayed repolarization associated with ventricular arrhythmias in a guinea pig model of Andersen-Tawil syndrome | | unrelated topic |
| 1. Vener, D. F.; Jacobs, J. P.; Schindler, E.; Maruszewski, B.; Andropoulos, D. | 2008 | | Databases for assessing the outcomes of the treatment of patients with congenital and paediatric cardiac disease–the perspective of anaesthesia | | unrelated topic |
| 1. Ugburo, A. O.; Desalu, I.; Adekola, A. F.; Fadeyibi, I. O. | 2009 | | Day case cleft lip surgery in Lagos, Nigeria | | unrelated topic |
| 1. Choong, C. S.; Nixon, G. M.; Blackmore, A. M.; Chen, W.; Jacoby, P.; Leonard, H.; Lafferty, A. R.; Ambler, G.; Kapur, N.; Bergman, P. B.; Schofield, C.; Seton, C.; Tai, A.; Tham, E.; Vora, K.; Crock, P.; Verge, C.; Musthaffa, Y.; Blecher, G.; Wilson, A.; Downs, J. | 2022 | | Daytime sleepiness and emotional and behavioral disturbances in Prader-Willi syndrome | | unrelated topic |
| 1. Bornman, R.; de Jager, C.; Worku, Z.; Farias, P.; Reif, S. | 2010 | | DDT and urogenital malformations in newborn boys in a malarial area | | unrelated topic |
| 1. Sellars, S.; Beighton, G.; Horan, F.; Beighton, P. H. | 1977 | | Deafness in Black children is Southern Africa | | Year of publication |
| 1. Mailosi, B. G. D.; Ruderman, T.; Klassen, S. L.; Kachimanga, C.; Aron, M. B.; Boti, M.; Kumwenda, K.; Bukhman, G.; Muula, A. S.; Banda, N. P. K.; Kwan, G. F. | 2023 | | Decentralized Heart Failure Management in Neno, Malawi | | unrelated topic |
| 1. Sayed, A. R.; Bourne, D.; Pattinson, R.; Nixon, J.; Henderson, B. | 2008 | | Decline in the prevalence of neural tube defects following folic acid fortification and its cost-benefit in South Africa | | unrelated topic |
| 1. Santens, B.; Van De Bruaene, A.; De Meester, P.; Claessen, G.; Moons, P.; Claus, P.; Goetschalckx, K.; Bogaert, J.; Budts, W. | 2023 | | Decreased cardiac reserve in asymptomatic patients after arterial switch operation for transposition of the great arteries | | unrelated topic |
| 1. Bala, U.; Leong, M. P.; Lim, C. L.; Shahar, H. K.; Othman, F.; Lai, M. I.; Law, Z. K.; Ramli, K.; Htwe, O.; Ling, K. H.; Cheah, P. S. | 2018 | | Defects in nerve conduction velocity and different muscle fibre-type specificity contribute to muscle weakness in Ts1Cje Down syndrome mouse model | | unrelated topic |
| 1. de Jong, G.; Kirby, P. A. | 2000 | | Defects of blastogenesis: counseling dilemmas in two families | | unrelated topic |
| 1. Hamad, D.; Yousef, Y.; Caminsky, N. G.; Guadagno, E.; Tran, V. A.; Laberge, J. M.; Emil, S.; Poenaru, D. | 2020 | | Defining the critical pediatric surgical workforce density for improving surgical outcomes: a global study | | unrelated topic |
| 1. Yousef, Y.; Lee, A.; Ayele, F.; Poenaru, D. | 2019 | | Delayed access to care and unmet burden of pediatric surgical disease in resource-constrained African countries | | unrelated topic |
| 1. Edmond, K. M.; Zandoh, C.; Quigley, M. A.; Amenga-Etego, S.; Owusu-Agyei, S.; Kirkwood, B. R. | 2006 | | Delayed breastfeeding initiation increases risk of neonatal mortality | | unrelated topic |
| 1. Govender, S.; Wiersma, R. | 2016 | | Delayed diagnosis of anorectal malformations (ARM): causes and consequences in a resource-constrained environment | | unrelated topic |
| 1. Nakubulwa, C.; Musiime, V.; Namiiro, F. B.; Tumwine, J. K.; Hongella, C.; Nyonyintono, J.; Hedstrom, A. B.; Opoka, R. | 2020 | | Delayed initiation of enteral feeds is associated with postnatal growth failure among preterm infants managed at a rural hospital in Uganda | | unrelated topic |
| 1. Bruneel, L.; Luyten, A.; Bettens, K.; D'Haeseleer, E.; Dhondt, C.; Hodges, A.; Galiwango, G.; Vermeersch, H.; Van Lierde, K. | 2017 | | Delayed primary palatal closure in resource-poor countries: Speech results in Ugandan older children and young adults with cleft (lip and) palate | | unrelated topic |
| 1. Ansong, A. K.; Yao, N. A.; Fynn-Thompson, F.; Edwin, F. | 2021 | | Delivering pediatric cardiac care in sub-Saharan Africa: a model for the developing countries | | unrelated topic |
| 1. Okwudire-Ejeh, I.; Ezike, K. N.; Mandong, B. M.; Dauda, A. M.; Binitie, P. O.; Shilong, D.; Akpa, P. O. | 2023 | | Demographic and Diagnostic Spectrum of Neurosurgical Biopsies: Initial Experience From a Re-established Neurosurgical Unit in a Tertiary Hospital in North Central Nigeria | | unrelated topic |
| 1. Ranjith, N.; Pegoraro, R. J.; Naidoo, D. P. | 2005 | | Demographic data and outcome of acute coronary syndrome in the South African Asian Indian population | | unrelated topic |
| 1. Gebremariam, H.; Tesfai, B.; Tewelde, S.; Abay, S.; Tekeste, D.; Kibreab, F. | 2022 | | Demographic, Clinical Profile and Outcomes of Neonates Admitted to Neonatal Intensive Care Unit of Dekemhare Hospital, Eritrea | | unrelated topic |
| 1. Okyere, P.; Ephraim, R. K. D.; Okyere, I.; Attakorah, J.; Serwaa, D.; Essuman, G.; Abaka-Yawson, A.; Adoba, P. | 2021 | | Demographic, diagnostic and therapeutic characteristics of autosomal dominant polycystic kidney disease in Ghana | | unrelated topic |
| 1. Adetayo, O. A.; Martin, M. C. | 2012 | | Demographics of cleft care providers in Africa and reported experience in training and practice: direct analysis of continent-based practitioners | | unrelated topic |
| 1. Punchak, M.; Nambi Najjuma, J.; Razak, S. S.; Nakaziba, Z.; Kasoba, A. M.; Haglund, M. M.; Fuller, A. T.; Kitya, D. | 2023 | | Demographics, referral patterns, and outcome of patients with neural tube defects in southwestern Uganda | | unrelated topic |
| 1. Nzomiwu, C. L.; Fomete, B.; Omisakin, O. O. | 2021 | | Dental Anomalies Associated with Orofacial Cleft among a Group of Individuals in Northwestern Nigeria | | unrelated topic |
| 1. Chibole, O. | 1988 | | Dental caries among children of high fluoride regions of Kenya | | Year of publication |
| 1. Olsson, B. | 1978 | | Dental caries and fluorosis in Arussi province, Ethiopia | | Year of publication |
| 1. Olsson, B. | 1978 | | Dental caries and fluorosis in Arussi province, Ethiopia | | Year of publication |
| 1. Sgan-Cohen, H. D.; Steinberg, D.; Zusman, S. P.; Sela, M. N. | 1992 | | Dental caries and its determinants among recent immigrants from rural Ethiopia | | Year of publication |
| 1. Ashi, H. | 2021 | | Dental Caries Experience among Down's Syndrome Population in Saudi Arabia - A Systematic Review | | unrelated topic |
| 1. Mapengo, M. A.; Marsicano, J. A.; Garcia de Moura, P.; Sales-Peres, A.; Hobdell, M.; de Carvalho Sales-Peres, S. H. | 2010 | | Dental caries in adolescents from public schools in Maputo, Mozambique | | unrelated topic |
| 1. Mosha, H. J.; Jorgen, L. | 1983 | | Dental caries, oral hygiene, periodontal disease and dental fluorosis among school children in northern Tanzania. Oral health surveys | | Year of publication |
| 1. Olsson, B. | 1976 | | Dental findings in high-fluoride areas in Ethiopia | | Year of publication |
| 1. Grobleri, S. R.; Louw, A. J.; van Kotze, T. J. | 2001 | | Dental fluorosis and caries experience in relation to three different drinking water fluoride levels in South Africa | | unrelated topic |
| 1. Wongdem, J. G.; Aderinokun, G. A.; Ubom, G. A.; Sridhar, M. K.; Selkur, S. | 2001 | | Dental fluorosis and fluoride mapping in Langtang town, Nigeria | | unrelated topic |
| 1. Menya, D.; Maina, S. K.; Kibosia, C.; Kigen, N.; Oduor, M.; Some, F.; Chumba, D.; Ayuo, P.; Middleton, D. R. S.; Osano, O.; Abedi-Ardekani, B.; Schüz, J.; McCormack, V. A. | 2019 | | Dental fluorosis and oral health in the African Esophageal Cancer Corridor: Findings from the Kenya ESCCAPE case-control study and a pan-African perspective | | unrelated topic |
| 1. Mabelya, L.; van Palenstein Helderman, W. H.; van't Hof, M. A.; König, K. G. | 1997 | | Dental fluorosis and the use of a high fluoride-containing trona tenderizer (magadi) | | Year of publication |
| 1. Shitumbanuma, V.; Tembo, F.; Tembo, J. M.; Chilala, S.; Van Ranst, E. | 2007 | | Dental fluorosis associated with drinking water from hot springs in Choma district in southern province, Zambia | | unrelated topic |
| 1. Manji, F.; Baelum, V.; Fejerskov, O. | 1986 | | Dental fluorosis in an area of Kenya with 2 ppm fluoride in the drinking water | | Year of publication |
| 1. Atia, G. S.; May, J. | 2013 | | Dental fluorosis in the paediatric patient | | unrelated topic |
| 1. Mabelya, L.; König, K. G.; van Palenstein Helderman, W. H. | 1992 | | Dental fluorosis, altitude, and associated dietary factors (short communication) | | Year of publication |
| 1. Makhanu, M.; Opinya, G.; Mutave, R. J. | 2009 | | Dental fluorosis, caries experience and snack intake of 13-15 year olds in Kenya | | unrelated topic |
| 1. Olsson, B. | 1979 | | Dental health situation in privileged children in Addis Ababa, Ethiopia | | Year of publication |
| 1. Peerbhay, F.; Titinchi, F. | 2014 | | Dental management of children with special healthcare needs | | unrelated topic |
| 1. Pindborg, J. J. | 1969 | | Dental mutilation and associated abnormalities in Uganda | | Duplicate |
| 1. Pindborg, J. J. | 1969 | | Dental mutilation and associated abnormalities in Uganda | | Year of publication |
| 1. Carstens, I. L.; Louw, A. J.; Kruger, E. | 1995 | | Dental status of rural school children in a sub-optimal fluoride area | | Year of publication |
| 1. Attin, T.; Mbiydzemo, F. N.; Villard, I.; Kielbassa, A. M.; Hellwig, E. | 1999 | | Dental status of schoolchildren from a rural community in Cameroon | | Year of publication |
| 1. Nalweyiso, N.; Busingye, J.; Whitworth, J.; Robinson, P. G. | 2004 | | Dental treatment needs of children in a rural subcounty of Uganda | | Duplicate |
| 1. Nalweyiso, N.; Busingye, J.; Whitworth, J.; Robinson, P. G. | 2004 | | Dental treatment needs of children in a rural subcounty of Uganda | | unrelated topic |
| 1. Igbigbi, P. S.; Adeloye, A. | 2005 | | Dermatoglyphics of mothers of Malawian children with spina bifida cystica: a comparative study with female controls | | unrelated topic |
| 1. Mensah, G. A. | 2013 | | Descriptive epidemiology of cardiovascular risk factors and diabetes in sub-Saharan Africa | | Duplicate |
| 1. Mensah, G. A. | 2013 | | Descriptive epidemiology of cardiovascular risk factors and diabetes in sub-Saharan Africa | | unrelated topic |
| 1. Obi, I. F.; Nwokoro, U. U.; Ossai, O. P.; Nwafor, M. I.; Nguku, P. | 2022 | | Descriptive epidemiology of external structural birth defects in Enugu State, Nigeria | | unrelated topic |
| 1. Eshete, M.; Butali, A.; Deressa, W.; Pagan-Rivera, K.; Hailu, T.; Abate, F.; Mohammed, I.; Demissie, Y.; Hailu, A.; Dawson, D. V.; Deribew, M.; Gessese, M.; Gravem, P. E.; Mossey, P. | 2017 | | Descriptive Epidemiology of Orofacial Clefts in Ethiopia | | unrelated topic |
| 1. Kagambèga, A. B.; Dembélé, R.; Bientz, L.; M'Zali, F.; Mayonnove, L.; Mohamed, A. H.; Coulibaly, H.; Barro, N.; Dubois, V. | 2023 | | Detection and Characterization of Carbapenemase-Producing Escherichia coli and Klebsiella pneumoniae from Hospital Effluents of Ouagadougou, Burkina Faso | | unrelated topic |
| 1. Steyn, K.; Fourie, J. M.; Shepherd, J. | 1998 | | Detection and measurement of hypercholesterolaemia in South Africans attending general practitioners in private practice–the cholesterol monitor | | Year of publication |
| 1. Sonou, A.; Hounkponou, M.; Codjo, L.; Adjagba, P. M.; Houehanou, C.; Dohou, H.; Assani, S.; Tchabi, Y.; Houenassi, M. | 2017 | | Detection of a Left Superior Vena Cava during a Pacemaker Implantation in Cotonou | | unrelated topic |
| 1. Akindolire, M. A.; Babalola, O. O.; Ateba, C. N. | 2015 | | Detection of Antibiotic Resistant Staphylococcus aureus from Milk: A Public Health Implication | | unrelated topic |
| 1. Elsaid, H. O. A.; Gadkareim, T.; Abobakr, T.; Mubarak, E.; Abdelrhem, M. A.; Abu, D.; Alhassan, E. A.; Abushama, H. | 2021 | | Detection of AZF microdeletions and reproductive hormonal profile analysis of infertile sudanese men pursuing assisted reproductive approaches | | unrelated topic |
| 1. Harika, K.; Shenoy, V. P.; Narasimhaswamy, N.; Chawla, K. | 2020 | | Detection of Biofilm Production and Its Impact on Antibiotic Resistance Profile of Bacterial Isolates from Chronic Wound Infections | | unrelated topic |
| 1. Tsehay, B.; Shitie, D.; Lake, A.; Abebaw, E.; Taye, A.; Essa, E. | 2019 | | Determinants and seasonality of major structural birth defects among newborns delivered at primary and referral hospital of East and West Gojjam zones, Northwest Ethiopia 2017-2018: case-control study | | unrelated topic |
| 1. Ahmed, A. T.; Farah, A. E.; Ali, H. N.; Ibrahim, M. O. | 2023 | | Determinants of early neonatal mortality (hospital based retrospective cohort study in Somali region of Ethiopia) | | unrelated topic |
| 1. Ademuyiwa, A. O.; Sowande, O. A.; Ijaduola, T. K.; Adejuyigbe, O. | 2009 | | Determinants of mortality in neonatal intestinal obstruction in Ile Ife, Nigeria | | unrelated topic |
| 1. Berhane, A.; Belachew, T. | 2023 | | Determinants of neural tube defects among women who gave birth in hospitals in Eastern Ethiopia: evidence from a matched case control study | | unrelated topic |
| 1. Yeshaw, Y.; Kebede, S. A.; Liyew, A. M.; Tesema, G. A.; Agegnehu, C. D.; Teshale, A. B.; Alem, A. Z. | 2020 | | Determinants of overweight/obesity among reproductive age group women in Ethiopia: multilevel analysis of Ethiopian demographic and health survey | | unrelated topic |
| 1. Astrøm, A. N.; Mashoto, K. | 2002 | | Determinants of self-rated oral health status among school children in northern Tanzania | | unrelated topic |
| 1. Tesfay, N.; Legesse, F.; Kebede, M.; Woldeyohannes, F. | 2022 | | Determinants of stillbirth among reviewed perinatal deaths in Ethiopia | | unrelated topic |
| 1. Worede, D. T.; Dagnew, G. W. | 2019 | | Determinants of stillbirth in Felege-Hiwot comprehensive specialized referral hospital, North-west, Ethiopia, 2019 | | unrelated topic |
| 1. Gizaw, W.; Feyisa, M.; Hailu, D.; Nigussie, T. | 2021 | | Determinants of stillbirth in hospitals of North Shoa Zone, Oromia region, Central Ethiopia: A case control study | | unrelated topic |
| 1. Huber, A. C.; Mosler, H. J. | 2013 | | Determining behavioral factors for interventions to increase safe water consumption: a cross-sectional field study in rural Ethiopia | | unrelated topic |
| 1. Ome-Kaius, M.; Unger, H. W.; Singirok, D.; Wangnapi, R. A.; Hanieh, S.; Umbers, A. J.; Elizah, J.; Siba, P.; Mueller, I.; Rogerson, S. J. | 2015 | | Determining effects of areca (betel) nut chewing in a prospective cohort of pregnant women in Madang Province, Papua New Guinea | | unrelated topic |
| 1. Warnakulasuriya, K. A.; Balasuriya, S.; Perera, P. A.; Peiris, L. C. | 1992 | | Determining optimal levels of fluoride in drinking water for hot, dry climates–a case study in Sri Lanka | | Duplicate |
| 1. Warnakulasuriya, K. A.; Balasuriya, S.; Perera, P. A.; Peiris, L. C. | 1992 | | Determining optimal levels of fluoride in drinking water for hot, dry climates–a case study in Sri Lanka | | Year of publication |
| 1. Berghoff, N. M.; Wilmshurst, J. M.; Page, T. A.; Wessels, M.; Schlegel, B.; Malcolm-Smith, S. | 2023 | | Determining the neurocognitive profile of children with tuberous sclerosis complex within the Western Cape region of South Africa | | unrelated topic |
| 1. Wright, N.; Abantanga, F.; Amoah, M.; Appeadu-Mensah, W.; Bokhary, Z.; Bvulani, B.; Davies, J.; Miti, S.; Nandi, B.; Nimako, B.; Poenaru, D.; Tabiri, S.; Yifieyeh, A.; Ade-Ajayi, N.; Sevdalis, N.; Leather, A. | 2019 | | Developing and implementing an interventional bundle to reduce mortality from gastroschisis in low-resource settings | | unrelated topic |
| 1. Sokhna, C.; Gaye, O.; Doumbo, O. | 2017 | | Developing Research in Infectious and Tropical Diseases in Africa: The Paradigm of Senegal | | unrelated topic |
| 1. Shrivastava, N.; Shrivastava, A.; Ninawe, S. M.; Sharma, S.; Kumar, J. S.; Alam, S. I.; Kanani, A.; Sharma, S. K.; Dash, P. K. | 2019 | | Development of Multispecies Recombinant Nucleoprotein-Based Indirect ELISA for High-Throughput Screening of Crimean-Congo Hemorrhagic Fever Virus-Specific Antibodies | | unrelated topic |
| 1. Wainwright, H.; Viljoen, D. | 1993 | | Developmental anomalies in monozygous twins resembling the human homologue of the mouse mutant disorganization | | Year of publication |
| 1. Masumo, R.; Bårdsen, A.; Astrøm, A. N. | 2013 | | Developmental defects of enamel in primary teeth and association with early life course events: a study of 6-36 month old children in Manyara, Tanzania | | unrelated topic |
| 1. Skinner, M. F.; Skinner, M. M.; Boesch, C. | 2012 | | Developmental defects of the dental crown in chimpanzees from the Taï National Park, Côte D'Ivoire: coronal waisting | | unrelated topic |
| 1. Folayan, M. O.; Chukwumah, N. M.; Popoola, B. O.; Temilola, D. O.; Onyejaka, N. K.; Oyedele, T. A.; Lawal, F. B. | 2018 | | Developmental defects of the enamel and its impact on the oral health quality of life of children resident in Southwest Nigeria | | unrelated topic |
| 1. Done, J. T. | 1976 | | Developmental disorders of the nervous system in animals | | Year of publication |
| 1. Eidelman, M.; Chezar, A.; Bialik, V. | 2002 | | Developmental dysplasia of the hip incidence in Ethiopian Jews revisited: 7-year prospective study | | Duplicate |
| 1. Eidelman, M.; Chezar, A.; Bialik, V. | 2002 | | Developmental dysplasia of the hip incidence in Ethiopian Jews revisited: 7-year prospective study | | unrelated topic |
| 1. Enslin, J. M.; Lefeuvre, D.; Taylor, A. | 2013 | | Developmental venous anomaly with contralateral impaired venous drainage in a 17-year-old male. A case report | | unrelated topic |
| 1. Salih, M. A.; Tuvemo, T. | 1991 | | Diabetes insipidus, diabetes mellitus, optic atrophy and deafness (DIDMOAD syndrome). A clinical study in two Sudanese families | | Year of publication |
| 1. Shipton, S. E.; van der Merwe, P. L.; Nel, E. D. | 2001 | | Diagnosis of haemodynamically significant patent ductus arteriosus in neonates– is the ECG of diagnostic help? | | unrelated topic |
| 1. Boutall, A.; Urban, M. F.; Stewart, C. | 2011 | | Diagnosis, etiology, and outcome of fetal ascites in a South African hospital | | unrelated topic |
| 1. Sulafa, K. M.; Karani, Z. | 2007 | | Diagnosis, management and outcome of heart disease in Sudanese patients | | age scope |
| 1. Edmond, K. M.; Quigley, M. A.; Zandoh, C.; Danso, S.; Hurt, C.; Owusu Agyei, S.; Kirkwood, B. R. | 2008 | | Diagnostic accuracy of verbal autopsies in ascertaining the causes of stillbirths and neonatal deaths in rural Ghana | | Duplicate |
| 1. Edmond, K. M.; Quigley, M. A.; Zandoh, C.; Danso, S.; Hurt, C.; Owusu Agyei, S.; Kirkwood, B. R. | 2008 | | Diagnostic accuracy of verbal autopsies in ascertaining the causes of stillbirths and neonatal deaths in rural Ghana | | unrelated topic |
| 1. Dimala, C. A.; Bechem, N. N.; Kadia, B. M.; Feteh, V. F.; Choukem, S. P. | 2017 | | Diagnostic and therapeutic challenges of an ambiguous cystic kidney disease in a resource limited setting: a case report | | unrelated topic |
| 1. Abegaz, B. | 1989 | | Diagnostic utility of echocardiography in Ethiopia | | Year of publication |
| 1. Dela, H.; Egyir, B.; Majekodunmi, A. O.; Behene, E.; Yeboah, C.; Ackah, D.; Bongo, R. N. A.; Bonfoh, B.; Zinsstag, J.; Bimi, L.; Addo, K. K. | 2022 | | Diarrhoeagenic E. coli occurrence and antimicrobial resistance of Extended Spectrum Beta-Lactamases isolated from diarrhoea patients attending health facilities in Accra, Ghana | | unrelated topic |
| 1. Ibinaiye, P. O.; Mshelbwala, P. M.; Abdulgafar, N.; Lawal, A. K. | 2013 | | Dicephalus dipus tetrabrachius conjoined twins of Zaria: case report and literature review | | unrelated topic |
| 1. Bornman, M. S.; Chevrier, J.; Rauch, S.; Crause, M.; Obida, M.; Sathyanarayana, S.; Barr, D. B.; Eskenazi, B. | 2016 | | Dichlorodiphenyltrichloroethane exposure and anogenital distance in the Venda Health Examination of Mothers, Babies and their Environment (VHEMBE) birth cohort study, South Africa | | unrelated topic |
| 1. Walker, A. R. | 1978 | | Diet and coronary heart disease | | Duplicate |
| 1. Walker, A. R. | 1978 | | Diet and coronary heart disease | | Year of publication |
| 1. Musaiger, A. O. | 2002 | | Diet and prevention of coronary heart disease in the Arab Middle East countries | | unrelated topic |
| 1. Kebede, A.; Retta, N.; Abuye, C.; Whiting, S. J.; Kassaw, M.; Zeru, T.; Tessema, M.; Kjellevold, M. | 2016 | | Dietary Fluoride Intake and Associated Skeletal and Dental Fluorosis in School Age Children in Rural Ethiopian Rift Valley | | unrelated topic |
| 1. Njelekela, M.; Ikeda, K.; Mtabaji, J.; Yamori, Y. | 2005 | | Dietary habits, plasma polyunsaturated fatty acids and selected coronary disease risk factors in Tanzania | | unrelated topic |
| 1. Kimble, R.; Keane, K. M.; Lodge, J. K.; Howatson, G. | 2019 | | Dietary intake of anthocyanins and risk of cardiovascular disease: A systematic review and meta-analysis of prospective cohort studies | | unrelated topic |
| 1. Wolmarans, P.; Seedat, Y. K.; Mayet, F. G.; Joubert, G.; Wentzel, E. | 1999 | | Dietary intake of Indians living in the metropolitan area of Durban | | Year of publication |
| 1. Berhane, A.; Fikadu, T.; Belachew, T. | 2022 | | Dietary practice among cohort pregnant women who gave birth to neonates with and without neural tube defect: a comparative cross-sectional study | | unrelated topic |
| 1. Kalk, W. J.; Joffe, B. I. | 2007 | | Differences in coronary heart disease prevalence and risk factors in African and White patients with type 2 diabetes | | unrelated topic |
| 1. Bütow, K. W.; van Wyk, P. J.; Zwahlen, R. A. | 2007 | | Differences in the clinical appearances of white versus black patients with facial cleft deformities: a retrospective study of a South African clinic | | unrelated topic |
| 1. Willems, R.; Ombelet, F.; Goossens, E.; De Groote, K.; Budts, W.; Moniotte, S.; de Hosson, M.; Van Bulck, L.; Marelli, A.; Moons, P.; De Backer, J.; Annemans, L. | 2021 | | Different levels of care for follow-up of adults with congenital heart disease: a cost analysis scrutinizing the impact on medical costs, hospitalizations, and emergency department visits | | unrelated topic |
| 1. Reda, S. M.; Chandra, M. | 2019 | | Dilated cardiomyopathy mutation (R174W) in troponin T attenuates the length-mediated increase in cross-bridge recruitment and myofilament Ca(2+) sensitivity | | unrelated topic |
| 1. Agou, S. H.; Basri, A. A.; Mudhaffer, S. M.; Altarazi, A. T.; Elhussein, M. A.; Imam, A. Y. | 2020 | | Dimensions of Maxillary Lateral Incisor on the Esthetic Perception of Smile: A Comparative Study of Dental Professionals and the General Population | | unrelated topic |
| 1. Dewan, P. A.; Lawrence, M. J.; Pip, A.; Kasa, S. | 1998 | | Diphallus associated with partial caudal duplication | | Year of publication |
| 1. Moons, P.; Skogby, S.; Bratt, E. L.; Zühlke, L.; Marelli, A.; Goossens, E. | 2021 | | Discontinuity of Cardiac Follow-Up in Young People With Congenital Heart Disease Transitioning to Adulthood: A Systematic Review and Meta-Analysis | | unrelated topic |
| 1. RS Cooper, B Osotimehin, JS Kaufman, T Forrester | 1998 | | Disease burden in sub-Saharan Africa: what should we conclude in the absence of data? | | unrelated topic |
| 1. van der Linden, W.; Cleaton-Jones, P.; Lownie, M. | 1995 | | Diseases and lesions associated with third molars. Review of 1001 cases | | Year of publication |
| 1. Robinson, D. C.; De Buse, P. J. | 1970 | | Dislocatable hip in Ugandan newborn infants | | Year of publication |
| 1. Griffiths, J. C. | 1970 | | Dislocated hip in East African infants and children | | Year of publication |
| 1. Abdullah, M. A.; Saeed, U.; Abass, A.; Lubna, K.; Weam, A.; Ali, A. S.; Elmwla, I. F. | 2012 | | Disorders of sex development among Sudanese children: 5-year experience of a pediatric endocrinology clinic | | unrelated topic |
| 1. Ekenze, S. O.; Nwangwu, E. I.; Amah, C. C.; Agugua-Obianyo, N. E.; Onuh, A. C.; Ajuzieogu, O. V. | 2015 | | Disorders of sex development in a developing country: perspectives and outcome of surgical management of 39 cases | | unrelated topic |
| 1. Ganie, Y.; Aldous, C.; Balakrishna, Y.; Wiersma, R. | 2017 | | Disorders of sex development in children in KwaZulu-Natal Durban South Africa: 20-year experience in a tertiary centre | | unrelated topic |
| 1. Ehua, A. M.; Moulot, M. O.; Agbara, K. S.; Enache, T.; Bankole, S. R. | 2023 | | Disorders of sex development: Challenges in a low-resource country | | unrelated topic |
| 1. Cairo, S.; Kakembo, N.; Kisa, P.; Muzira, A.; Cheung, M.; Healy, J.; Ozgediz, D.; Sekabira, J. | 2017 | | Disparity in access and outcomes for emergency neonatal surgery: intestinal atresia in Kampala, Uganda | | unrelated topic |
| 1. Bowa, K.; Rodriguez, V. J.; Malik, F. S.; Knight, J.; Cristofari, N.; Parrish, M. S.; Jones, D. L.; Zulu, R.; Weiss, S. M. | 2022 | | Dissemination of the Spear & Shield Project using a Training of Trainers Model: A reflection on challenges and successes | | unrelated topic |
| 1. Solomon, L.; McLaren, P.; Irwig, L.; Gear, J. S.; Schnitzler, C. M.; Gear, A.; Mann, D. | 1986 | | Distinct types of hip disorder in Mseleni joint disease | | Year of publication |
| 1. Fadare, F. T.; Okoh, A. I. | 2021 | | Distribution and molecular characterization of ESBL, pAmpC β-lactamases, and non-β-lactam encoding genes in Enterobacteriaceae isolated from hospital wastewater in Eastern Cape Province, South Africa | | unrelated topic |
| 1. Spritz, R. A.; Arnold, T. D.; Buonocore, S.; Carter, D.; Fingerlin, T.; Odero, W. W.; Wambani, J. O.; Tenge, R. K.; Weatherley-White, R. C. | 2007 | | Distribution of orofacial clefts and frequent occurrence of an unusual cleft variant in the Rift Valley of Kenya | | unrelated topic |
| 1. Awamleh, Z.; Chater-Diehl, E.; Choufani, S.; Wei, E.; Kianmahd, R. R.; Yu, A.; Chad, L.; Costain, G.; Tan, W. H.; Scherer, S. W.; Arboleda, V. A.; Russell, B. E.; Weksberg, R. | 2022 | | DNA methylation signature associated with Bohring-Opitz syndrome: a new tool for functional classification of variants in ASXL genes | | unrelated topic |
| 1. Gao, Y.; Ma, X. J.; Huang, G. Y.; Zhang, J.; Wang, H. J.; Ma, D.; Wu, Y. | 2012 | | DNA sequencing of TGFβ2 in sporadic patients with tetralogy of Fallot | | unrelated topic |
| 1. Abdur-Rahman, L. O.; Shawyer, A.; Vizcarra, R.; Bailey, K.; Cameron, B. H. | 2014 | | Do geography and resources influence the need for colostomy in Hirschsprung's disease and anorectal malformations? A Canadian association of paediatric surgeons: association of paediatric surgeons of Nigeria survey | | unrelated topic |
| 1. Vishram, J. K.; Borglykke, A.; Andreasen, A. H.; Jeppesen, J.; Ibsen, H.; Jørgensen, T.; Broda, G.; Palmieri, L.; Giampaoli, S.; Donfrancesco, C.; Kee, F.; Mancia, G.; Cesana, G.; Kuulasmaa, K.; Salomaa, V.; Sans, S.; Ferrieres, J.; Tamosiunas, A.; Söderberg, S.; McElduff, P.; Arveiler, D.; Pajak, A.; Olsen, M. H. | 2014 | | Do other cardiovascular risk factors influence the impact of age on the association between blood pressure and mortality? The MORGAM Project | | unrelated topic |
| 1. Brisighelli, G.; Loveland, J.; Bebington, C.; Dyamara, L.; Ferrari, G.; Westgarth-Taylor, C. | 2020 | | Do social circumstances dictate a change in the setup of an anorectal malformation clinic? | | unrelated topic |
| 1. Alemu, F. M.; Yalew, A. W. | 2021 | | Does antiretroviral therapy cause congenital malformations? A systematic review and meta-analysis | | unrelated topic |
| 1. Gericke, G. S.; Hofmeyr, G. J.; Laburn, H.; Isaacs, H. | 1989 | | Does heat damage fetuses? | | Year of publication |
| 1. Durhan, M. A.; Agrali, O. B.; Kiyan, E.; Ikizoglu, N. B.; Ersu, R.; Tanboga, I. | 2019 | | Does obstructive sleep apnea affect oral and periodontal health in children with down syndrome? A preliminary study | | un+B2596:B2609related topic |
| 1. Heradien, M. J.; Goosen, A.; Crotti, L.; Durrheim, G.; Corfield, V.; Brink, P. A.; Schwartz, P. J. | 2006 | | Does pregnancy increase cardiac risk for LQT1 patients with the KCNQ1-A341V mutation? | | unrelated topic |
| 1. Brhane, M.; Hagos, B.; Abrha, M. W.; Weldearegay, H. G. | 2019 | | Does short inter-pregnancy interval predicts the risk of preterm birth in Northern Ethiopia? | | Duplicate |
| 1. Brhane, M.; Hagos, B.; Abrha, M. W.; Weldearegay, H. G. | 2019 | | Does short inter-pregnancy interval predicts the risk of preterm birth in Northern Ethiopia? | | unrelated topic |
| 1. Amoo, A. T.; James, O.; Adeyemi, M.; Taiwo, A. O.; Adeyemo, W. L. | 2021 | | Does the Initial Width of Cleft Lip Play a Role in the Occurrence of Immediate Local Complications Following Primary Cleft Lip Repairs? | | unrelated topic |
| 1. Anselem, O.; Saurel-Cubizolles, M. J.; Khoshnood, B.; Blondel, B.; Sauvegrain, P.; Bertille, N.; Azria, E. | 2021 | | Does women's place of birth affect their opportunity for an informed choice about Down syndrome screening? A population-based study in France | | unrelated topic |
| 1. Pereira, G. F. M.; Kim, A.; Jalil, E. M.; Fernandes Fonseca, F.; Shepherd, B. E.; Veloso, V. G.; Rick, F.; Ribeiro, R.; Pimenta, M. C.; Beber, A.; Corrêa, R. G.; Lima, R.; Maruri, F.; McGowan, C. C.; Schwartz Benzaken, A.; Grinsztejn, B.; Castilho, J. L. | 2021 | | Dolutegravir and pregnancy outcomes in women on antiretroviral therapy in Brazil: a retrospective national cohort study | | unrelated topic |
| 1. Kourtis, A. P.; Zhu, W.; Lampe, M. A.; Huang, Y. A.; Hoover, K. W. | 2023 | | Dolutegravir and pregnancy outcomes including neural tube defects in the USA during 2008-20: a national cohort study | | unrelated topic |
| 1. Raesima, M. M.; Ogbuabo, C. M.; Thomas, V.; Forhan, S. E.; Gokatweng, G.; Dintwa, E.; Petlo, C.; Motswere-Chirwa, C.; Rabold, E. M.; Tinker, S. C.; Odunsi, S.; Malima, S.; Mmunyane, O.; Modise, T.; Kefitlhile, K.; Dare, K.; Letebele, M.; Roland, M. E.; Moore, C. A.; Modi, S.; Williamson, D. M. | 2019 | | Dolutegravir Use at Conception - Additional Surveillance Data from Botswana | | unrelated topic |
| 1. An, F.; Chen, T.; Stéphanie, D. M.; Li, K.; Li, Q. X.; Carvalho, L. J.; Tomlins, K.; Li, J.; Gu, B.; Chen, S. | 2016 | | Domestication Syndrome Is Investigated by Proteomic Analysis between Cultivated Cassava (Manihot esculenta Crantz) and Its Wild Relatives | | unrelated topic |
| 1. Lesan, W. R.; Wandenya, R. | 1989 | | Double supernumerary impacted premolar teeth: case report | | Year of publication |
| 1. Kruszka, PS | 2015 | | Down syndrome | | unrelated topic |
| 1. Christianson, A. L. | 1997 | | Down syndrome in black South African infants and children–clinical features and delayed diagnosis | | Year of publication |
| 1. Kruszka, P.; Porras, A. R.; Sobering, A. K.; Ikolo, F. A.; La Qua, S.; Shotelersuk, V.; Chung, B. H.; Mok, G. T.; Uwineza, A.; Mutesa, L.; Moresco, A.; Obregon, M. G.; Sokunbi, O. J.; Kalu, N.; Joseph, D. A.; Ikebudu, D.; Ugwu, C. E.; Okoromah, C. A.; Addissie, Y. A.; Pardo, K. L.; Brough, J. J.; Lee, N. C.; Girisha, K. M.; Patil, S. J.; Ng, I. S.; Min, B. C.; Jamuar, S. S.; Tibrewal, S.; Wallang, B.; Ganesh, S.; Sirisena, N. D.; Dissanayake, V. H.; Paththinige, C. S.; Prabodha, L. B.; Richieri-Costa, A.; Muthukumarasamy, P.; Thong, M. K.; Jones, K. L.; Abdul-Rahman, O. A.; Ekure, E. N.; Adeyemo, A. A.; Summar, M.; Linguraru, M. G.; Muenke, M. | 2017 | | Down syndrome in diverse populations | | unrelated topic |
| 1. Christianson, A. L. | 1996 | | Down syndrome in sub-Saharan Africa | | Year of publication |
| 1. Kromberg, J. G.; Christianson, A. L.; Duthie-Nurse, G.; Zwane, E.; Jenkins, T. | 1992 | | Down syndrome in the black population | | Year of publication |
| 1. Smart, R. D. | 1981 | | Down syndrome in the Cape Peninsula and the value of amniocentesis as a preventive measure | | Year of publication |
| 1. Boroffice, R. A. | 1978 | | Down's syndrome in Nigeria: dermatoglyphic analysis of 50 cases | | Year of publication |
| 1. Boroffice, R. A. | 1979 | | Down's syndrome in Nigeria: pregnancy wastage in mothers of Down's syndrome | | Year of publication |
| 1. Op't Hof, J.; Venter, P. A.; Louw, M. | 1991 | | Down's syndrome in South Africa–incidence, maternal age and utilisation of prenatal diagnosis | | Year of publication |
| 1. Gatford, A. | 2001 | | Down's syndrome: experiences of mothers from different cultures | | unrelated topic |
| 1. Mugadza, D. T.; Nduku, S. I.; Gweme, E.; Manhokwe, S.; Marume, P.; Mugari, A.; Magogo, C.; Jombo, T. Z. | 2021 | | Drinking water quality and antibiotic resistance of E. coli and Salmonella spp. from different sources in Gweru urban, Zimbabwe | | unrelated topic |
| 1. Scholz, E. P.; Zitron, E.; Kiesecker, C.; Lueck, S.; Kathöfer, S.; Thomas, D.; Weretka, S.; Peth, S.; Kreye, V. A.; Schoels, W.; Katus, H. A.; Kiehn, J.; Karle, C. A. | 2003 | | Drug binding to aromatic residues in the HERG channel pore cavity as possible explanation for acquired Long QT syndrome by antiparkinsonian drug budipine | | unrelated topic |
| 1. Offor, I.; Awodele, O.; Oshikoya, K. A. | 2019 | | Drug-related teratogenic and pathologic causes of birth defects in a tertiary hospital in Southwestern Nigeria | | unrelated topic |
| 1. Källén, B | 2009 | | Drugs during pregnancy | | book |
| 1. Källén, B | 2016 | | Drugs during pregnancy: Methodological aspects | | book |
| 1. Aghaji, A. E.; Bowman, R.; Ofoegbu, V. C.; Smith, A. | 2017 | | Dual sensory impairment in special schools in South-Eastern Nigeria | | unrelated topic |
| 1. Adeyemi, S. D. | 1986 | | Duodenal obstruction in Nigerian newborns and infants | | Year of publication |
| 1. Paraskevas, GK; Raikos, A; Ioannidis, O; ... | 2011 | | Duplicated gallbladder: surgical application and review of the literature | | unrelated topic |
| 1. Mirazo, J. E.; Page, P.; Rubio-Martinez, L.; Marais, H. J.; Lyle, C. | 2014 | | Dynamic upper respiratory abnormalities in Thoroughbred racehorses in South Africa | | unrelated topic |
| 1. Oosthuizen, P.; Lambert, T.; Castle, D. J. | 1998 | | Dysmorphic concern: prevalence and associations with clinical variables | | Year of publication |
| 1. Mubungu, G.; Makay, P.; Lumaka, A.; Mvuama, N.; Tshika, D.; Tady, B. P.; Biselele, T.; Roelants, M.; Tshilobo, P. L.; Devriendt, K. | 2021 | | Dysmorphism and major anomalies are a main predictor of survival in newborns admitted to the neonatal intensive care unit in the Democratic Republic of Congo | | unrelated topic |
| 1. Aliyu, M. H.; Salihu, H. M.; Kouam, L. | 2003 | | Eagle-Barrett syndrome: occurrence and outcomes | | unrelated topic |
| 1. Onakpoya, U. U.; Adenle, A. D.; Adenekan, A. T. | 2017 | | Early experience with open heart surgery in a pioneer private hospital in West Africa: the Biket medical centre experience | | unrelated topic |
| 1. Lehtonen, L.; Gimeno, A.; Parra-Llorca, A.; Vento, M. | 2017 | | Early neonatal death: A challenge worldwide | | unrelated topic |
| 1. Kabuluzi, E.; Campbell, M.; McGowan, L.; Chirwa, E.; Brabin, L. | 2014 | | Early pregnancy exposure to feto-toxic medications among out-patients in Malawi | | unrelated topic |
| 1. Wolf, B.; Kalangu, K. | 1993 | | Early repair of frontoethmoidal meningoencephalocele in Bulawayo, Zimbabwe | | Year of publication |
| 1. Manuel, V.; Morais, H.; Magalhães, M. P.; Nunes, M. A.; Leon, G.; Ferreira, M.; Filipe Júnior, A. P. | 2015 | | Ebstein's anomaly in children: a single-center study in Angola | | unrelated topic |
| 1. Freers, J.; Mayanja-Kizza, H.; Ziegler, J. L.; Rutakingirwa, M. | 1996 | | Echocardiographic diagnosis of heart disease in Uganda | | Year of publication |
| 1. Oyati, A. I.; Danbauchi, S. S.; Ameh, E. A.; Mshelbwala, P. M.; Anumah, M. A.; Ogunrinde, G. O.; Anyiam, J. O.; Azuh, P. C. | 2009 | | Echocardiographic findings in children with surgically correctable non-cardiac congenital anomalies | | unrelated topic |
| 1. Mapelli, M.; Zagni, P.; Calbi, V.; Fusini, L.; Twalib, A.; Ferrara, R.; Mattavelli, I.; Alberghina, L.; Salvioni, E.; Opira, C.; Kansiime, J.; Tamborini, G.; Pepi, M.; Agostoni, P. | 2022 | | Echocardiographic Screening for Rheumatic Heart Disease in a Ugandan Orphanage: Feasibility and Outcomes | | unrelated topic |
| 1. Ekure, E. N.; Amadi, C.; Sokunbi, O.; Kalu, N.; Olusegun-Joseph, A.; Kushimo, O.; Hassan, O.; Ikebudu, D.; Onyia, S.; Onwudiwe, C.; Nwankwo, V.; Akinwunmi, R.; Awusa, F.; Akere, Z.; Dele-Salawu, O.; Ajayi, E.; Ale, O.; Muoneke, D.; Muenke, M.; Kruszka, P.; Beaton, A.; Sable, C.; Adeyemo, A. | 2019 | | Echocardiographic screening of 4107 Nigerian school children for rheumatic heart disease | | unrelated topic |
| 1. Ellis, J.; Martin, R.; Wilde, P.; Tometzki, A.; Senkungu, J.; Nansera, D. | 2007 | | Echocardiographic, chest X-ray and electrocardiogram findings in children presenting with heart failure to a Ugandan paediatric ward | | unrelated topic |
| 1. Otaigbe, B. E.; Tabansi, P. N.; Agbedey, G. O. | 2012 | | Echocardiography findings in clinically confirmed congenital rubella syndrome cases seen at the University of Port Harcourt Teaching Hospital, Nigeria | | unrelated topic |
| 1. Agomuoh, D. I.; Akpa, M. R.; Alasia, D. D. | 2006 | | Echocardiography in the University of Port Harcourt Teaching Hospital: April 2000 to March 2003 | | unrelated topic |
| 1. Beaton, A.; Okello, E.; Lwabi, P.; Mondo, C.; McCarter, R.; Sable, C. | 2012 | | Echocardiography screening for rheumatic heart disease in Ugandan schoolchildren | | unrelated topic |
| 1. A Beaton, E Okello, P Lwabi, C Mondo, R McCarter… | 2012 | | Echocardiography screening for rheumatic heart disease in Ugandan schoolchildren | | unrelated topic |
| 1. Isezuo, S. A.; Ekele, B. A. | 2004 | | Eclampsia and abnormal QTc | | unrelated topic |
| 1. Edwin, F. | 2010 | | eComment: Incomplete left ventricular reverse remodeling after revascularization of anomalous left coronary artery from the pulmonary artery | | Duplicate |
| 1. Edwin, F. | 2010 | | eComment: outcome of extracorporeal membrane oxygenation in pediatric cardiac surgery - impact of residual lesions | | unrelated topic |
| 1. Poenaru, D.; Lin, D.; Corlew, S. | 2016 | | Economic Valuation of the Global Burden of Cleft Disease Averted by a Large Cleft Charity | | unrelated topic |
|  | 1975 | | Editorial: Blood transfusion services | | Year of publication |
| 1. Sluman, M. A.; Apers, S.; Sluiter, J. K.; Nieuwenhuijsen, K.; Moons, P.; Luyckx, K.; Kovacs, A. H.; Thomet, C.; Budts, W.; Enomoto, J.; Yang, H. L.; Jackson, J. L.; Khairy, P.; Cook, S. C.; Subramanyan, R.; Alday, L.; Eriksen, K.; Dellborg, M.; Berghammer, M.; Mattsson, E.; Mackie, A. S.; Menahem, S.; Caruana, M.; Gosney, K.; Soufi, A.; Fernandes, S. M.; White, K. S.; Callus, E.; Kutty, S.; Bouma, B. J.; Mulder, B. J. M. | 2019 | | Education as important predictor for successful employment in adults with congenital heart disease worldwide | | unrelated topic |
| 1. Racape, J.; De Spiegelaere, M.; Dramaix, M.; Haelterman, E.; Alexander, S. | 2013 | | Effect of adopting host-country nationality on perinatal mortality rates and causes among immigrants in Brussels | | unrelated topic |
| 1. Abdelwahab, M. T.; Court, R.; Everitt, D.; Diacon, A. H.; Dawson, R.; Svensson, E. M.; Maartens, G.; Denti, P. | 2021 | | Effect of Clofazimine Concentration on QT Prolongation in Patients Treated for Tuberculosis | | unrelated topic |
| 1. Franz, D. N.; Budde, K.; Kingswood, J. C.; Belousova, E.; Sparagana, S.; de Vries, P. J.; Berkowitz, N.; Ridolfi, A.; Bissler, J. J. | 2018 | | Effect of everolimus on skin lesions in patients treated for subependymal giant cell astrocytoma and renal angiomyolipoma: final 4-year results from the randomized EXIST-1 and EXIST-2 studies | | unrelated topic |
| 1. Berhane, A.; Belachew, T. | 2022 | | Effect of Picture-based health education and counselling on knowledge and adherence to preconception Iron-folic acid supplementation among women planning to be pregnant in Eastern Ethiopia: a randomized controlled trial | | unrelated topic |
| 1. Jonson, K. M.; Lyle, J. G.; Edwards, M. J.; Penny, R. H. | 1976 | | Effect of prenatal heat stress on brain growth and serial discrimination reversal learning in the guinea pig | | Year of publication |
| 1. Knorst, M. M.; Kienast, K.; Riechelmann, H.; Müller-Quernheim, J.; Ferlinz, R. | 1994 | | Effect of sulfur dioxide on mucociliary activity and ciliary beat frequency in guinea pig trachea | | Year of publication |
| 1. Urban, M. F.; Stewart, C.; Ruppelt, T.; Geerts, L. | 2011 | | Effectiveness of prenatal screening for Down syndrome on the basis of maternal age in Cape Town | | unrelated topic |
| 1. Rigato, M.; Nollino, L.; Tiago, A.; Spedicato, L.; Simango, L. M. C.; Putoto, G.; Avogaro, A.; Fadini, G. P. | 2022 | | Effectiveness of remote screening for diabetic retinopathy among patients referred to Mozambican Diabetes Association (AMODIA): a retrospective observational study | | unrelated topic |
| 1. Bratt, E. L.; Mora, M. A.; Sparud-Lundin, C.; Saarijärvi, M.; Burström, Å; Skogby, S.; Fernlund, E.; Fadl, S.; Rydberg, A.; Hanseus, K.; Kazamia, K.; Moons, P. | 2023 | | Effectiveness of the STEPSTONES Transition Program for Adolescents With Congenital Heart Disease-A Randomized Controlled Trial | | unrelated topic |
| 1. Familoni, O. B.; Alebiosu, C. O.; Ayodele, O. E. | 2006 | | Effects and outcome of haemodialysis on QT intervals and QT dispersion in patients with chronic kidney disease | | unrelated topic |
| 1. Beserra, S. S.; Santos-Miranda, A.; Sarmento, J. O.; Miranda, V. M.; Roman-Campos, D. | 2020 | | Effects of amiodarone on rodent ventricular cardiomyocytes: Novel perspectives from a cellular model of Long QT Syndrome Type 3 | | unrelated topic |
| 1. de Costa, C.; Griew, A. R. | 1982 | | Effects of betel chewing on pregnancy outcome | | Year of publication |
| 1. de Costa, C.; riew, A. R. | 1982 | | Effects of betel chewing on pregnancy outcome | | Year of publication |
| 1. Dopke, C.; Connor, J.; Zheleva, B.; Gauvreau, K.; Bakalcheva, B.; Bina, N.; Calvimontes, G.; Cerovic, I.; Majani, N.; Oketcho, M.; Pechilkov, D.; Shidhika, F.; Shiryaev, T.; Jenkins, K. | 2022 | | Effects of COVID-19 on paediatric cardiac centres in low-income and middle-income countries: a mixed-methods study | | unrelated topic |
| 1. Boer, P. H. | 2018 | | Effects of detraining on anthropometry, aerobic capacity and functional ability in adults with Down syndrome | | unrelated topic |
| 1. Ndibazza, J.; Muhangi, L.; Akishule, D.; Kiggundu, M.; Ameke, C.; Oweka, J.; Kizindo, R.; Duong, T.; Kleinschmidt, I.; Muwanga, M.; Elliott, A. M. | 2010 | | Effects of deworming during pregnancy on maternal and perinatal outcomes in Entebbe, Uganda: a randomized controlled trial | | unrelated topic |
| 1. Crocker, M. E.; Hossen, S.; Goodman, D.; Simkovich, S. M.; Kirby, M.; Thompson, L. M.; Rosa, G.; Garg, S. S.; Thangavel, G.; McCollum, E. D.; Peel, J.; Clasen, T.; Checkley, W. | 2020 | | Effects of high altitude on respiratory rate and oxygen saturation reference values in healthy infants and children younger than 2 years in four countries: a cross-sectional study | | unrelated topic |
| 1. Ezenwa, B.; Pena, E.; Schlegel, A.; Bapat, R.; Shepherd, E. G.; Nelin, L. D. | 2019 | | Effects of practice change on outcomes of extremely preterm infants with patent ductus arteriosus | | unrelated topic |
| 1. Shibeshi, W.; Baye, A. M.; Alemkere, G.; Engidawork, E. | 2021 | | Efficacy and Safety of Artemisinin-Based Combination Therapy for the Treatment of Uncomplicated Malaria in Pregnant Women: A Systematic Review and Meta-Analysis | | unrelated topic |
| 1. Kimani, J.; Phiri, K.; Kamiza, S.; Duparc, S.; Ayoub, A.; Rojo, R.; Robbins, J.; Orrico, R.; Vandenbroucke, P. | 2016 | | Efficacy and Safety of Azithromycin-Chloroquine versus Sulfadoxine-Pyrimethamine for Intermittent Preventive Treatment of Plasmodium falciparum Malaria Infection in Pregnant Women in Africa: An Open-Label, Randomized Trial | | unrelated topic |
| 1. Ndyomugyenyi, R.; Kabatereine, N.; Olsen, A.; Magnussen, P. | 2008 | | Efficacy of ivermectin and albendazole alone and in combination for treatment of soil-transmitted helminths in pregnancy and adverse events: a randomized open label controlled intervention trial in Masindi district, western Uganda | | Duplicate |
| 1. Ndyomugyenyi, R.; Kabatereine, N.; Olsen, A.; Magnussen, P. | 2008 | | Efficacy of ivermectin and albendazole alone and in combination for treatment of soil-transmitted helminths in pregnancy and adverse events: a randomized open label controlled intervention trial in Masindi district, western Uganda | | unrelated topic |
| 1. Simões, E. A. F.; Madhi, S. A.; Muller, W. J.; Atanasova, V.; Bosheva, M.; Cabañas, F.; Baca Cots, M.; Domachowske, J. B.; Garcia-Garcia, M. L.; Grantina, I.; Nguyen, K. A.; Zar, H. J.; Berglind, A.; Cummings, C.; Griffin, M. P.; Takas, T.; Yuan, Y.; Wählby Hamrén, U.; Leach, A.; Villafana, T. | 2023 | | Efficacy of nirsevimab against respiratory syncytial virus lower respiratory tract infections in preterm and term infants, and pharmacokinetic extrapolation to infants with congenital heart disease and chronic lung disease: a pooled analysis of randomised controlled trials | | unrelated topic |
| 1. Han, M.; Xing, H.; Chen, L.; Cui, M.; Zhang, Y.; Qi, L.; Jin, M.; Yang, Y.; Gao, C.; Gao, Z.; Xing, X.; Huang, W. | 2021 | | Efficient antiglioblastoma therapy in mice through doxorubicin-loaded nanomicelles modified using a novel brain-targeted RVG-15 peptide | | unrelated topic |
| 1. Chinawa, J. M.; Arodiwe, I.; Onyia, J. T.; Chinawa, A. T. | 2023 | | Eisenmenger Syndrome: A Revisit of a Hidden but Catastrophic Disease | | unrelated topic |
| 1. Levy, A. | 2009 | | Either/or: sports, sex, and the case of Caster Semenya | | unrelated topic |
| 1. Oguanobi, N. I.; Onwubere, B. J.; Ike, S. O.; Anisiuba, B. C.; Ejim, E. C.; Ibegbulam, O. G. | 2010 | | Electocardiographic findings in adult Nigerians with sickle cell anaemia | | unrelated topic |
| 1. Roozen, G. V. T.; Meel, R.; Peper, J.; Venter, W. D. F.; Barth, R. E.; Grobbee, D. E.; Klipstein-Grobusch, K.; Vos, A. G. | 2021 | | Electrocardiographic and echocardiographic abnormalities in urban African people living with HIV in South Africa | | unrelated topic |
| 1. Carlsson, L.; Amos, G. J.; Andersson, B.; Drews, L.; Duker, G.; Wadstedt, G. | 1997 | | Electrophysiological characterization of the prokinetic agents cisapride and mosapride in vivo and in vitro: implications for proarrhythmic potential? | | Year of publication |
| 1. Diaz, S. B. | 1991 | | EM waves standards effectiveness | | Year of publication |
| 1. Gomes, A. S.; Mali, W. P.; Oppenheim, W. L. | 1982 | | Embolization therapy in the management of congenital arteriovenous malformations | | Year of publication |
| 1. Hill, S. C.; Vasconcelos, J.; Neto, Z.; Jandondo, D.; Zé-Zé, L.; Aguiar, R. S.; Xavier, J.; Thézé, J.; Mirandela, M.; Micolo Cândido, A. L.; Vaz, F.; Sebastião, C. D. S.; Wu, C. H.; Kraemer, M. U. G.; Melo, A.; Schamber-Reis, B. L. F.; de Azevedo, G. S.; Tanuri, A.; Higa, L. M.; Clemente, C.; da Silva, S. P.; da Silva Candido, D.; Claro, I. M.; Quibuco, D.; Domingos, C.; Pocongo, B.; Watts, A. G.; Khan, K.; Alcantara, L. C. J.; Sabino, E. C.; Lackritz, E.; Pybus, O. G.; Alves, M. J.; Afonso, J.; Faria, N. R. | 2019 | | Emergence of the Asian lineage of Zika virus in Angola: an outbreak investigation | | unrelated topic |
| 1. Ameh, E. A.; Dogo, P. M.; Nmadu, P. T. | 2001 | | Emergency neonatal surgery in a developing country | | unrelated topic |
| 1. Joseph Davey, D. L.; Pintye, J.; Baeten, J. M.; Aldrovandi, G.; Baggaley, R.; Bekker, L. G.; Celum, C.; Chi, B. H.; Coates, T. J.; Haberer, J. E.; Heffron, R.; Kinuthia, J.; Matthews, L. T.; McIntyre, J.; Moodley, D.; Mofenson, L. M.; Mugo, N.; Myer, L.; Mujugira, A.; Shoptaw, S.; Stranix-Chibanda, L.; John-Stewart, G. | 2020 | | Emerging evidence from a systematic review of safety of pre-exposure prophylaxis for pregnant and postpartum women: where are we now and where are we heading? | | unrelated topic |
| 1. Akinduti, P. A.; Obafemi, Y. D.; Ugboko, H.; El-Ashker, M.; Akinnola, O.; Agunsoye, C. J.; Oladotun, A.; Phiri, B. S. J.; Oranusi, S. U. | 2022 | | Emerging vancomycin-non susceptible coagulase negative Staphylococci associated with skin and soft tissue infections | | unrelated topic |
| 1. Bordi, L.; Avsic-Zupanc, T.; Lalle, E.; Vairo, F.; Capobianchi, M. R.; da Costa Vasconcelos, P. F. | 2017 | | Emerging Zika Virus Infection: A Rapidly Evolving Situation | | unrelated topic |
| 1. Manji, F.; Baelum, V.; Fejerskov, O.; Gemert, W. | 1986 | | Enamel changes in two low-fluoride areas of Kenya | | Year of publication |
| 1. Rasmussen, P.; Elhassan, E.; Raadal, M. | 1992 | | Enamel defects in primary canines related to traditional treatment of teething problems in Sudan | | Year of publication |
| 1. Feller, L.; Wood, N. H.; Anagnostopoulos, C.; Bouckaert, M.; Raubenheimer, E. J.; Kramer, B.; Lemmer, J. | 2008 | | Enamel dysplasia with hamartomatous atypical follicular hyperplasia: review of the literature and report of a case | | unrelated topic |
| 1. Richards, A.; Fejerskov, O.; Baelum, V. | 1989 | | Enamel fluoride in relation to severity of human dental fluorosis | | Year of publication |
| 1. Brunet, M.; Fronty, P.; Sapanet, M.; de Bonis, L.; Viriot, L. | 2002 | | Enamel hypoplasia in a pliocene hominid from Chad | | unrelated topic |
| 1. Mizrahi, E. | 1982 | | Enamel opacities in primary and high school pupils | | Year of publication |
| 1. Warf, B. C.; Stagno, V.; Mugamba, J. | 2011 | | Encephalocele in Uganda: ethnic distinctions in lesion location, endoscopic management of hydrocephalus, and survival in 110 consecutive children | | Duplicate |
| 1. Warf, B. C.; Stagno, V.; Mugamba, J. | 2011 | | Encephalocele in Uganda: ethnic distinctions in lesion location, endoscopic management of hydrocephalus, and survival in 110 consecutive children | | unrelated topic |
| 1. Ugwuanyi, U.; Ayogu, O.; Onobun, D. E.; Salawu, M.; Mordi, C. O. | 2022 | | Encephalocele: A Case Series From Abuja, North Central Nigeria | | unrelated topic |
| 1. Munyi, N.; Poenaru, D.; Bransford, R.; Albright, L. | 2009 | | Encephalocele–a single institution African experience | | unrelated topic |
| 1. Onuigbo, W. I. | 1977 | | Encephaloceles in Nigerian Igbos | | Year of publication |
| 1. Kwan, G. F.; Mayosi, B. M.; Mocumbi, A. O.; Miranda, J. J.; Ezzati, M.; Jain, Y.; Robles, G.; Benjamin, E. J.; Subramanian, S. V.; Bukhman, G. | 2016 | | Endemic Cardiovascular Diseases of the Poorest Billion | | Duplicate |
| 1. Kwan, G. F.; Mayosi, B. M.; Mocumbi, A. O.; Miranda, J. J.; Ezzati, M.; Jain, Y.; Robles, G.; Benjamin, E. J.; Subramanian, S. V.; Bukhman, G. | 2016 | | Endemic Cardiovascular Diseases of the Poorest Billion | | unrelated topic |
| 1. Hamilton, W. | 1976 | | Endemic cretinism | | Year of publication |
| 1. McGill, P. E. | 1995 | | Endemic fluorosis | | Year of publication |
| 1. Walvekar, S. V.; Qureshi, B. A. | 1982 | | Endemic fluorosis and partial defluoridation of water supplies - A public health concern in Kenya | | Year of publication |
| 1. Nair, K. R.; Manji, F. | 1982 | | Endemic fluorosis in deciduous dentition. A study of 1276 children in typically high fluoride area (Kiambu) in Kenya | | Year of publication |
| 1. Haimanot, R. T.; Fekadu, A.; Bushra, B. | 1987 | | Endemic fluorosis in the Ethiopian Rift Valley | | Duplicate |
| 1. Haimanot, R. T.; Fekadu, A.; Bushra, B. | 1987 | | Endemic fluorosis in the Ethiopian Rift Valley | | Year of publication |
| 1. Mwaniki, D. L.; Courtney, J. M.; Gaylor, J. D. | 1994 | | Endemic fluorosis: an analysis of needs and possibilities based on case studies in Kenya | | Year of publication |
| 1. Pharoah, P. D.; Heywood, P. F. | 1994 | | Endemic goitre and cretinism in the Simbai and Tep-Tep areas of Madang Province, Papua New Guinea | | Year of publication |
| 1. Hodes, R. M. | 1993 | | Endocarditis in Ethiopia. Analysis of 51 cases from Addis Ababa | | Year of publication |
| 1. Adeogun, A. O.; Onibonoje, K.; Ibor, O. R.; Omiwole, R. A.; Chukwuka, A. V.; Ugwumba, A. O.; Ugwumba, A. A.; Arukwe, A. | 2016 | | Endocrine-disruptor molecular responses, occurrence of intersex and gonado-histopathological changes in tilapia species from a tropical freshwater dam (Awba Dam) in Ibadan, Nigeria | | unrelated topic |
| 1. Buchanan, G. D.; Tredoux, S.; Nel, C.; Gamieldien, M. Y. | 2021 | | Endodontic treatment of dentin dysplasia type I D | | unrelated topic |
| 1. Jimenez-Gomez, A.; Castillo, H.; Burckart, C.; Castillo, J. | 2017 | | Endoscopic Third Ventriculostomy to address hydrocephalus in Africa: A call for education and community-based rehabilitation | | unrelated topic |
| 1. S Naicker | 2013 | | End-stage renal disease in Sub-Saharan Africa | | unrelated topic |
| 1. Amuasi, G. R.; Dsani, E.; Owusu-Nyantakyi, C.; Owusu, F. A.; Mohktar, Q.; Nilsson, P.; Adu, B.; Hendriksen, R. S.; Egyir, B. | 2023 | | Enterococcus species: insights into antimicrobial resistance and whole-genome features of isolates recovered from livestock and raw meat in Ghana | | unrelated topic |
| 1. Demelash Enyew, H.; Bogale, B. G.; Hailu, A. B.; Mereta, S. T. | 2023 | | Environmental exposures and adverse pregnancy outcomes in Ethiopia: A systematic review and meta-analysis | | unrelated topic |
| 1. Riana Bornman, M. S.; Bouwman, H. | 2012 | | Environmental pollutants and diseases of sexual development in humans and wildlife in South Africa: harbingers of impact on overall health? | | unrelated topic |
| 1. Acolatse, J. E. E.; Portal, E. A. R.; Boostrom, I.; Akafity, G.; Dakroah, M. P.; Chalker, V. J.; Sands, K.; Spiller, O. B. | 2022 | | Environmental surveillance of ESBL and carbapenemase-producing gram-negative bacteria in a Ghanaian Tertiary Hospital | | unrelated topic |
| 1. Bhettay, E.; Nelson, M. M.; Beighton, P. | 1975 | | Epidemic of conjoined twins in Southern Africa? | | Year of publication |
| 1. Shakib, K. | 2016 | | Epidemic of Zika virus and maxillofacial surgery | | unrelated topic |
| 1. Yameogo, N. V.; Sondo, K. A.; Yameogo, A. A.; Kagambega, L. J.; Mandi, D. G.; Kologo, K. J.; Millogo, G.; Toguyeni, B. J.; Samadoulougou, A. K.; Kabore, N. J.; Zabsonre, P. | 2013 | | Epidemiological and clinical features, ultrasound findings and prognosis of right-sided infective endocarditis in a teaching hospital in Ouagadougou | | unrelated topic |
| 1. Impellizzeri, A.; Giannantoni, I.; Polimeni, A.; Barbato, E.; Galluccio, G. | 2019 | | Epidemiological characteristic of Orofacial clefts and its associated congenital anomalies: retrospective study | | unrelated topic |
| 1. Rakotoarivelo, R. A.; Raberahona, M.; Rasamoelina, T.; Rabezanahary, A.; Rakotomalala, F. A.; Razafinambinintsoa, T.; Bénet, T.; Vanhems, P.; Randria, M. J. D.; Romanò, L.; Cogliati, M.; Cornet, M.; Rakoto Andrianarivelo, M. | 2020 | | Epidemiological characteristics of cryptococcal meningoencephalitis associated with Cryptococcus neoformans var. grubii from HIV-infected patients in Madagascar: A cross-sectional study | | unrelated topic |
| 1. Naouali, C.; Jones, M.; Nabouli, I.; Jerbi, M.; Tounsi, H.; Ben Rekaya, M.; Ben Ahmed, M.; Bouhaouala, B.; Messaoud, O.; Khaled, A.; Zghal, M.; Abdelhak, S.; Boubaker, S.; Yacoub-Youssef, H. | 2017 | | Epidemiological trends and clinicopathological features of cutaneous melanoma in sporadic and xeroderma pigmentosum Tunisian patients | | unrelated topic |
| 1. Rosas-Navarro, J.; Gaillot, H.; Agoulon, A.; Ferri, C.; Ruel, Y.; Paoletti, C.; Quinton, J. F.; Curros-Moreno, Á; Gouni, V. | 2023 | | Epidemiological, clinical, radiographic, echocardiographic findings and outcome in client-owned guinea pigs (Cavia porcellus) with cardiac disease: 80 cases (2010-2021) | | unrelated topic |
| 1. Ngouana, T. K.; Toghueo, R. M. K.; Kenfack, I. F.; Lachaud, L.; Nana, A. K.; Tadjou, L.; Kouanfack, C.; Boyom, F. F.; Bertout, S. | 2019 | | Epidemiology and antifungal susceptibility testing of non-albicansCandida species colonizing mucosae of HIV-infected patients in Yaoundé (Cameroon) | | unrelated topic |
| 1. Hlongwa, P.; Levin, J.; Rispel, L. C. | 2019 | | Epidemiology and clinical profile of individuals with cleft lip and palate utilising specialised academic treatment centres in South Africa | | unrelated topic |
| 1. Olowu, W. A.; Adefehinti, O.; Aladekomo, T. A. | 2013 | | Epidemiology and clinicopathologic outcome of pediatric chronic kidney disease in Nigeria, a single cenetr study | | unrelated topic |
| 1. Endalifer, M. L.; Diress, G. | 2020 | | Epidemiology and determinant factors of neural tube defect: Narrative review | | unrelated topic |
| 1. K Sliwa, A Damasceno, BM Mayosi | 2005 | | Epidemiology and etiology of cardiomyopathy in Africa | | unrelated topic |
| 1. Iani, F. C. M.; Giovanetti, M.; Fonseca, V.; Souza, W. M.; Adelino, T. E. R.; Xavier, J.; Jesus, J. G.; Pereira, M. A.; Silva, M. V. F.; Costa, A. V. B.; Silva, E. C.; Mendes, M. C. O.; Filippis, A. M. B.; Albuquerque, C. F. C.; Abreu, A. L.; Oliveira, M. A. A.; Alcantara, L. C. J.; Faria, N. R. | 2021 | | Epidemiology and evolution of Zika virus in Minas Gerais, Southeast Brazil | | unrelated topic |
| 1. Cheung, M.; Kakembo, N.; Rizgar, N.; Grabski, D.; Ullrich, S.; Muzira, A.; Kisa, P.; Sekabira, J.; Ozgediz, D. | 2019 | | Epidemiology and mortality of pediatric surgical conditions: insights from a tertiary center in Uganda | | unrelated topic |
| 1. Taverne-Ghadwal, L.; Kuhns, M.; Buhl, T.; Schulze, M. H.; Mbaitolum, W. J.; Kersch, L.; Weig, M.; Bader, O.; Groß, U. | 2022 | | Epidemiology and Prevalence of Oral Candidiasis in HIV Patients From Chad in the Post-HAART Era | | unrelated topic |
| 1. VT Nkomo | 2007 | | Epidemiology and prevention of valvular heart diseases and infective endocarditis in Africa | | unrelated topic |
| 1. Ajike, S. O.; Adebola, R. A.; Efunkoya, A.; Adeoye, J.; Akitoye, O.; Veror, N. | 2013 | | Epidemiology of adult cleft patients in North-western Nigeria: our experience | | unrelated topic |
| 1. Loufoua-Lemay, Anne Berthe M’pemba and Massamba, Alphonse | 2016 | | Epidemiology of cardiovascular diseases in children at the teaching hospital of Brazzaville, Congo | | Included |
| 1. Mezei, G.; Sudan, M.; Izraeli, S.; Kheifets, L. | 2014 | | Epidemiology of childhood leukemia in the presence and absence of Down syndrome | | unrelated topic |
| 1. Madaree, A. | 2023 | | Epidemiology of Clefts in Kwazulu Natal: Comparison With Systematic Review Analysis, Similarities, and Differences | | unrelated topic |
| 1. Kouame, B. D.; N'Guetta-Brou, I. A.; Kouame, G. S.; Sounkere, M.; Koffi, M.; Yaokreh, J. B.; Odehouri-Koudou, T.; Tembely, S.; Dieth, G. A.; Ouattara, O.; Dick, R. | 2015 | | Epidemiology of congenital abnormalities in West Africa: Results of a descriptive study in teaching hospitals in Abidjan: Cote d'Ivoire | | source and study population |
| 1. Eke, C. B.; Uche, E. O.; Chinawa, J. M.; Obi, I. E.; Obu, H. A.; Ibekwe, R. C. | 2016 | | Epidemiology of congenital anomalies of the central nervous system in children in Enugu, Nigeria: A retrospective study | | unrelated topic |
| 1. Chibole, O. | 1987 | | Epidemiology of dental fluorosis in Kenya | | Year of publication |
| 1. Bakare, T. I.; Sowande, O. A.; Adejuyigbe, O. O.; Chinda, J. Y.; Usang, U. E. | 2009 | | Epidemiology of external birth defects in neonates in Southwestern Nigeria | | unrelated topic |
| 1. NBA Ntusi, BM Mayosi | 2009 | | Epidemiology of heart failure in sub-Saharan Africa | | unrelated topic |
| 1. Noubiap, J. J.; Nkeck, J. R.; Kwondom, B. S.; Nyaga, U. F. | 2022 | | Epidemiology of infective endocarditis in Africa: a systematic review and meta-analysis | | unrelated topic |
| 1. CL Onen | 2013 | | Epidemiology of ischaemic heart disease in sub-Saharan Africa | | unrelated topic |
| 1. Bobowick, A. R.; Brody, J. A. | 1973 | | Epidemiology of motor-neuron diseases | | Year of publication |
| 1. Bickler, S. W.; Sanno-Duanda, B. | 2000 | | Epidemiology of paediatric surgical admissions to a government referral hospital in the Gambia | | unrelated topic |
| 1. Getahun, M.; Beyene, B.; Gallagher, K.; Ademe, A.; Teshome, B.; Tefera, M.; Asha, A.; Afework, A.; Assefa, E.; HaileMariam, Y.; HaileGiorgis, Y.; Ketema, H.; Shiferaw, D.; Bekele, A.; Jima, D.; Kebede, A. | 2016 | | Epidemiology of rubella virus cases in the pre-vaccination era of Ethiopia, 2009-2015 | | Duplicate |
| 1. Getahun, M.; Beyene, B.; Gallagher, K.; Ademe, A.; Teshome, B.; Tefera, M.; Asha, A.; Afework, A.; Assefa, E.; HaileMariam, Y.; HaileGiorgis, Y.; Ketema, H.; Shiferaw, D.; Bekele, A.; Jima, D.; Kebede, A. | 2016 | | Epidemiology of rubella virus cases in the pre-vaccination era of Ethiopia, 2009-2015 | | unrelated topic |
| 1. Deseda, C. C. | 2017 | | Epidemiology of Zika | | unrelated topic |
| 1. Désiré, A. M.; Buhendwa, C.; Césaire, T. M. A.; Prisca, K. I. S.; Levi, L. N.; David, N. B.; Longombe, A. O.; Pierre, W. | 2020 | | Epidemiology, Diagnosis and Therapeutic Approaches of Cryptorchidism at the Panzi General Hospital, DR Congo: A 5-year Retrospective Study | | unrelated topic+B2572:B2583 |
| 1. Burg, M. L.; Chai, Y.; Yao, C. A.; Magee, 3rd, W.; Figueiredo, J. C. | 2016 | | Epidemiology, Etiology, and Treatment of Isolated Cleft Palate | | unrelated topic |
| 1. Wright, N. J.; Zani, A.; Ade-Ajayi, N. | 2015 | | Epidemiology, management and outcome of gastroschisis in Sub-Saharan Africa: Results of an international survey | | unrelated topic |
| 1. Howe, E. G. | 2014 | | Epilogue: ethical goals for the future | | unrelated topic |
| 1. Poenaru, D.; Pemberton, J.; Frankfurter, C.; Cameron, B. H.; Stolk, E. | 2017 | | Establishing disability weights for congenital pediatric surgical conditions: a multi-modal approach | | unrelated topic |
| 1. Wataganara, T.; Seshadri, S.; Leung, T. Y.; Matter, C.; Ngerncham, M.; Triyasunant, N.; Mali, P. V.; Biswas, A.; Nawapun, K.; Phithakwatchara, N.; Flake, A. W.; Johnson, M. P.; Choolani, M. | 2017 | | Establishing Prenatal Surgery for Myelomeningocele in Asia: The Singapore Consensus | | unrelated topic |
| 1. Allotey, P.; Reidpath, D. | 2001 | | Establishing the causes of childhood mortality in Ghana: the 'spirit child' | | unrelated topic |
| 1. Patel, V. P.; Patroneva, A.; Glaze, D. G.; Davis Ms, K.; Merikle, E.; Revana, A. | 2022 | | Establishing the content validity of the Epworth Sleepiness Scale for Children and Adolescents in Prader-Willi syndrome | | unrelated topic |
| 1. Stewart, B. T.; Carlson, L.; Hatcher, K. W.; Sengupta, A.; Vander Burg, R. | 2016 | | Estimate of Unmet Need for Cleft Lip and/or Palate Surgery in India | | unrelated topic |
| 1. Rios-Blancas, M. J.; Pando-Robles, V.; Razo, C.; Carcamo, C. P.; Mendoza, W.; Pacheco-Barrios, K.; Miranda, J. J.; Lansingh, V. C.; Demie, T. G.; Saha, M.; Okonji, O. C.; Yigit, A.; Cahuana-Hurtado, L.; Chacón-Uscamaita, P. R.; Bernabe, E.; Culquichicon, C.; Chirinos-Caceres, J. L.; Cárdenas, R.; Alcalde-Rabanal, J. E.; Barrera, F. J.; Quintanilla, B. P. A.; Shorofi, S. A.; Wickramasinghe, N. D.; Ferreira, N.; Almidani, L.; Gupta, V. K.; Karimi, H.; Alayu, D. S.; Benziger, C. P.; Fukumoto, T.; Mostafavi, E.; Redwan, E. M. M.; Gebrehiwot, M.; Khatab, K.; Koyanagi, A.; Krapp, F.; Lee, S.; Noori, M.; Qattea, I.; Rosenthal, V. D.; Sakshaug, J. W.; Wagaye, B.; Zare, I.; Ortega-Altamirano, D. V.; Murillo-Zamora, E.; Vervoort, D.; Silva, D. A. S.; Oulhaj, A.; Herrera-Serna, B. Y.; Mehra, R.; Amir-Behghadami, M.; Adib, N.; Cortés, S.; Dang, A. K.; Nguyen, B. T.; Mokdad, A. H.; Hay, S. I.; Murray, C. J. L.; Lozano, R.; García, P. J. | 2023 | | Estimating mortality and disability in Peru before the COVID-19 pandemic: a systematic analysis from the Global Burden of the Disease Study 2019 | | unrelated topic |
| 1. Lawn, J. E.; Wilczynska-Ketende, K.; Cousens, S. N. | 2006 | | Estimating the causes of 4 million neonatal deaths in the year 2000 | | unrelated topic |
| 1. André, J. M.; Berger, J. M.; De Turckheim, M.; Veyssière, G. | 1975 | | Estimation of testosterone and androstenedione in the plasma and testes of cryptorchid offspring of mice treated with oestradiol during pregnancy | | Year of publication |
| 1. Nguah, S. B. | 2014 | | Ethical aspects of arranging local medical collaboration and care | | unrelated topic |
| 1. Tambo, E.; Madjou, G.; Khayeka-Wandabwa, C.; Olalubi, O. A.; Chengho, C. F.; Khater, E. I. M. | 2017 | | Ethical, legal and societal considerations on Zika virus epidemics complications in scaling-up prevention and control strategies | | bookSection |
| 1. Sirois, F.; Gbeha, E.; Sanni, A.; Chretien, M.; Labuda, D.; Mbikay, M. | 2008 | | Ethnic differences in the frequency of the cardioprotective C679X PCSK9 mutation in a West African population | | unrelated topic |
| 1. Ravelli, A. C.; Schaaf, J. M.; Eskes, M.; Abu-Hanna, A.; de Miranda, E.; Mol, B. W. | 2013 | | Ethnic disparities in perinatal mortality at 40 and 41 weeks of gestation | | unrelated topic |
| 1. Miller, G. J.; Beckles, G. L.; Maude, G. H.; Carson, D. C.; Alexis, S. D.; Price, S. G.; Byam, N. T. | 1989 | | Ethnicity and other characteristics predictive of coronary heart disease in a developing community: principal results of the St James Survey, Trinidad | | Year of publication |
| 1. Adere, A.; Bedru, M.; Afework, M. | 2023 | | Etiologies and Patterns of Valvular Heart Disease Among Cardiac Patients at the Cardiac Center of Ethiopia During February 2000 to April 2022 | | unrelated topic |
| 1. Olurin, O. | 1970 | | Etiology of blindness in Nigerian children | | Year of publication |
| 1. Chang, J.; Wang, S.; Zheng, Z. | 2020 | | Etiology of Hypospadias: A Comparative Review of Genetic Factors and Developmental Processes Between Human and Animal Models | | unrelated topic |
| 1. Stacey, RB; Caine, AJ; Hundley, WG | 2015 | | Evaluation and management of left ventricular noncompaction cardiomyopathy | | unrelated topic |
| 1. TU, K. Dilek; Oktay, A.; Aygun, E. G.; Ünsal, G.; Pata, Ö | 2023 | | Evaluation fetal heart in the first and second trimester: Results and limitations | | unrelated topic |
| 1. Neethling, W. M.; Strange, G.; Firth, L.; Smit, F. E. | 2013 | | Evaluation of a tissue-engineered bovine pericardial patch in paediatric patients with congenital cardiac anomalies: initial experience with the ADAPT-treated CardioCel(R) patch | | unrelated topic |
| 1. Mercaldo, R. A.; Bellan, S. E. | 2019 | | Evaluation of alternative endpoints for ZIKV vaccine efficacy trials | | unrelated topic |
| 1. Naik, S. S.; Joshi, A.; Winnier, J. J.; Patil, D. D.; Gore, P. J.; Mali, S. S. | 2023 | | Evaluation of dental anxiety in children with Down's syndrome using dog-assisted therapy: A pilot study | | unrelated topic |
| 1. Robert, M. G.; Romero, C.; Dard, C.; Garnaud, C.; Cognet, O.; Girard, T.; Rasamoelina, T.; Cornet, M.; Maubon, D. | 2020 | | Evaluation of ID Fungi Plates Medium for Identification of Molds by MALDI Biotyper | | unrelated topic |
| 1. Bekiroglu, N.; Mete, S.; Ozbay, G.; Yalcinkaya, S.; Kargul, B. | 2015 | | Evaluation of panoramic radiographs taken from 1,056 Turkish children | | unrelated topic |
| 1. Ogbole, O. O.; Ndabai, N. C.; Akinleye, T. E.; Attah, A. F. | 2020 | | Evaluation of peptide-rich root extracts of Calliandria portoriscensis (Jacq.) Benth (Mimosaceae) for in vitro antimicrobial activity and brine shrimp lethality | | unrelated topic |
| 1. Sadoh, W. E.; Okperi, B.; Ikhurionan, P.; Monday, P.; Sadoh, A. E. | 2020 | | Evaluation of Predictors of Mortality Associated With Childhood Heart Failure in Nigeria: A 2-Center Study | | unrelated topic |
| 1. Monday, P.; Idouriyekemwen, N. J.; Sadoh, W. E. | 2020 | | Evaluation of renal injury in children with uncorrected CHDs with significant shunt using urinary neutrophil gelatinase-associated lipocalin | | unrelated topic |
| 1. Eghwrudjakpor, P. O.; Amadi, C. E.; Amusan, E. O. | 2011 | | Evaluation of the level of awareness of the role of folic acid in the prevention of neural tube defects amongst women of reproductive age in a tertiary health institution | | unrelated topic |
| 1. Sadoh, W. E.; Eyo-Ita, E.; Okugbo, S. O. | 2022 | | Evaluation of the Prevalence and Anatomic Types of Congenital Heart Diseases: An Echocardiographic Study in a Tertiary Hospital in Nigeria | | unrelated topic |
| 1. Bissler, J. J.; Kingswood, J. C.; Radzikowska, E.; Zonnenberg, B. A.; Frost, M.; Belousova, E.; Sauter, M.; Nonomura, N.; Brakemeier, S.; de Vries, P. J.; Berkowitz, N.; Miao, S.; Segal, S.; Peyrard, S.; Budde, K. | 2016 | | Everolimus for renal angiomyolipoma in patients with tuberous sclerosis complex or sporadic lymphangioleiomyomatosis: extension of a randomized controlled trial | | unrelated topic |
| 1. Le Saux, O.; Beck, K.; Sachsinger, C.; Treiber, C.; Göring, H. H.; Curry, K.; Johnson, E. W.; Bercovitch, L.; Marais, A. S.; Terry, S. F.; Viljoen, D. L.; Boyd, C. D. | 2002 | | Evidence for a founder effect for pseudoxanthoma elasticum in the Afrikaner population of South Africa | | unrelated topic |
| 1. Gupta, H.; Sakharwade, S. C.; Angural, A.; Kotambail, A.; Bhat, G. K.; Hande, M. H.; D'Souza, S. C.; Rao, P.; Kumari, V.; Saadi, A. V.; Satyamoorthy, K. | 2013 | | Evidence for genetic linkage between a polymorphism in the GNAS gene and malaria in South Indian population | | unrelated topic |
| 1. Viljoen, D.; Ramesar, R. | 1992 | | Evidence for paternal imprinting in familial Beckwith-Wiedemann syndrome | | Year of publication |
| 1. Homs, M.; Rodriguez-Frias, F.; Gregori, J.; Ruiz, A.; Reimundo, P.; Casillas, R.; Tabernero, D.; Godoy, C.; Barakat, S.; Quer, J.; Riveiro-Barciela, M.; Roggendorf, M.; Esteban, R.; Buti, M. | 2016 | | Evidence of an Exponential Decay Pattern of the Hepatitis Delta Virus Evolution Rate and Fluctuations in Quasispecies Complexity in Long-Term Studies of Chronic Delta Infection | | unrelated topic |
| 1. Prakash, A.; Sharma, C.; Singh, A.; Kumar Singh, P.; Kumar, A.; Hagen, F.; Govender, N. P.; Colombo, A. L.; Meis, J. F.; Chowdhary, A. | 2016 | | Evidence of genotypic diversity among Candida auris isolates by multilocus sequence typing, matrix-assisted laser desorption ionization time-of-flight mass spectrometry and amplified fragment length polymorphism | | unrelated topic |
| 1. Lubick, N. | 2010 | | Examining DDT's urogenital effects | | bookSection |
| 1. Sapra, R.; Ghose, T.; Sudan, D.; Singh, B.; Kaul, U.; Wasir, H. S. | 1999 | | Exceptional survival of a patient with ventricular septal defect and Eisenmenger syndrome | | Year of publication |
| 1. Maddirevula, S.; Alsahli, S.; Alhabeeb, L.; Patel, N.; Alzahrani, F.; Shamseldin, H. E.; Anazi, S.; Ewida, N.; Alsaif, H. S.; Mohamed, J. Y.; Alazami, A. M.; Ibrahim, N.; Abdulwahab, F.; Hashem, M.; Abouelhoda, M.; Monies, D.; Al Tassan, N.; Alshammari, M.; Alsagheir, A.; Seidahmed, M. Z.; Sogati, S.; Aglan, M. S.; Hamad, M. H.; Salih, M. A.; Hamed, A. A.; Alhashmi, N.; Nabil, A.; Alfadli, F.; Abdel-Salam, G. M. H.; Alkuraya, H.; Peitee, W. O.; Keng, W. T.; Qasem, A.; Mushiba, A. M.; Zaki, M. S.; Fassad, M. R.; Alfadhel, M.; Alexander, S.; Sabr, Y.; Temtamy, S.; Ekbote, A. V.; Ismail, S.; Hosny, G. A.; Otaify, G. A.; Amr, K.; Al Tala, S.; Khan, A. O.; Rizk, T.; Alaqeel, A.; Alsiddiky, A.; Singh, A.; Kapoor, S.; Alhashem, A.; Faqeih, E.; Shaheen, R.; Alkuraya, F. S. | 2018 | | Expanding the phenome and variome of skeletal dysplasia | | unrelated topic |
| 1. Edwin, F.; Sereboe, L. A.; Tettey, M. M.; Aniteye, E. A.; Kotei, D. A.; Tamatey, M. M.; Entsua-Mensah, K.; Frimpong-Boateng, K. | 2010 | | Experience from a single centre concerning the surgical spectrum and outcome of adolescents and adults with congenitally malformed hearts in West Africa | | unrelated topic |
| 1. Leidinger, A.; Piquer, J.; Kim, E. E.; Nahonda, H.; Qureshi, M. M.; Young, P. H. | 2019 | | Experience in the Early Surgical Management of Myelomeningocele in Zanzibar | | unrelated topic |
| 1. Lasebikan, O. A.; Anetekhai, W. I.; Asuquo, J. E.; Anikwe, I. A.; Oguzie, G. C.; Abang, I. E.; Omoke, N. I.; Asuquo, B. J. | 2023 | | Experience with accelerated ponseti technique for treatment of idiopathic clubfoot in a regional orthopaedic hospital in Nigeria | | unrelated topic |
| 1. Anyanwu, C. H.; Umeh, B. U.; Swarup, A. S. | 1982 | | Experience with civilian vascular injuries in eastern Nigeria | | Year of publication |
| 1. Adeyemo, W. L.; Jokomba, L. A.; Somefun, O. A.; Mofikoya, B. O. | 2008 | | Experience with prosthetic reconstruction of ear defects at LUTH, Lagos, Nigeria | | unrelated topic |
| 1. Ogundoyin, O. O.; Olulana, D. I.; Lawal, T. A. | 2021 | | Experience with the management of anorectal malformations in Ibadan, Nigeria | | unrelated topic |
| 1. Olasoji, O.; Arotiba, T.; Dogo, D. | 2002 | | Experience with unoperated cleft lip and palate patients in a Nigerian teaching hospital | | unrelated topic |
| 1. Goy, R. W. | 1970 | | Experimental control of psychosexuality | | Year of publication |
| 1. Nagano, K.; Nakayama, E.; Oobayashi, H.; Nishizawa, T.; Okuda, H.; Yamazaki, K. | 1984 | | Experimental studies on toxicity of ethylene glycol alkyl ethers in Japan | | Year of publication |
| 1. Kengne Kamga, K.; De Vries, J.; Nguefack, S.; Munung, N. S.; Wonkam, A. | 2021 | | Explanatory models for the cause of Fragile X Syndrome in rural Cameroon | | Duplicate |
| 1. Kengne Kamga, K.; De Vries, J.; Nguefack, S.; Munung, N. S.; Wonkam, A. | 2021 | | Explanatory models for the cause of Fragile X Syndrome in rural Cameroon | | unrelated topic |
| 1. Dellicour, S.; Desai, M.; Mason, L.; Odidi, B.; Aol, G.; Phillips-Howard, P. A.; Laserson, K. F.; Ter Kuile, F. O. | 2013 | | Exploring risk perception and attitudes to miscarriage and congenital anomaly in rural Western Kenya | | unrelated topic |
| 1. Gebrehiwot, A. G.; Melka, D. S.; Kassaye, Y. M.; Gemechu, T.; Lako, W.; Hinou, H.; Nishimura, S. I. | 2019 | | Exploring serum and immunoglobulin G N-glycome as diagnostic biomarkers for early detection of breast cancer in Ethiopian women | | unrelated topic |
| 1. Kidane, S.; Shamebo, S. D.; Ntaganda, E.; Petroze, R. T.; McNatt, Z.; Wong, R.; Rabideau, M. | 2022 | | Exploring the lived experiences of parents caring for infants with gastroschisis in Rwanda: The untold story | | unrelated topic |
| 1. Bede-Ojimadu, O.; Orisakwe, O. E. | 2020 | | Exposure to Wood Smoke and Associated Health Effects in Sub-Saharan Africa: A Systematic Review | | unrelated topic |
| 1. Adeyinka, A. O.; Ibinaiye, P. O. | 2006 | | Expression of adult polycystic renal disease in a 17-year-old male | | unrelated topic |
| 1. Chiurazzi, P.; Destro-Bisol, G.; Genuardi, M.; Oostra, B. A.; Spedini, G.; Neri, G. | 1996 | | Extended gene diversity at the FMR1 locus and neighbouring CA repeats in a sub-Saharan population | | Year of publication |
| 1. Al-Ani, M. H.; Mageet, A. O. | 2018 | | Extraction Planning in Orthodontics | | unrelated topic |
| 1. Syed, S; O'Sullivan, TL; Phillips, KP | 2022 | | Extreme heat and pregnancy outcomes: a scoping review of the epidemiological evidence | | book |
| 1. Engelken, J.; Carnero-Montoro, E.; Pybus, M.; Andrews, G. K.; Lalueza-Fox, C.; Comas, D.; Sekler, I.; de la Rasilla, M.; Rosas, A.; Stoneking, M.; Valverde, M. A.; Vicente, R.; Bosch, E. | 2014 | | Extreme population differences in the human zinc transporter ZIP4 (SLC39A4) are explained by positive selection in Sub-Saharan Africa | | unrelated topic |
| 1. Manni, J. J. | 1982 | | F.P. Weber syndrome in a Ghanaian child | | Year of publication |
| 1. Porras, A. R.; Bramble, M. S.; Mosema Be Amoti, K.; Spencer, D.; Dakande, C.; Manya, H.; Vashist, N.; Likuba, E.; Ebwel, J. M.; Musasa, C.; Malherbe, H.; Mohammed, B.; Tor-Diez, C.; Ngoyi, D. M.; Katumbay, D. T.; Linguraru, M. G.; Vilain, E. | 2021 | | Facial analysis technology for the detection of Down syndrome in the Democratic Republic of the Congo | | unrelated topic |
| 1. Benjamin, F.; Adebayo, E. T.; Mohammed, R.; Adekeye, E. O. | 2013 | | Facial cleft with Amelia: a Nigerian case report | | unrelated topic |
| 1. Lumaka, A.; Cosemans, N.; Lulebo Mampasi, A.; Mubungu, G.; Mvuama, N.; Lubala, T.; Mbuyi-Musanzayi, S.; Breckpot, J.; Holvoet, M.; de Ravel, T.; Van Buggenhout, G.; Peeters, H.; Donnai, D.; Mutesa, L.; Verloes, A.; Lukusa Tshilobo, P.; Devriendt, K. | 2017 | | Facial dysmorphism is influenced by ethnic background of the patient and of the evaluator | | unrelated topic |
| 1. Manyama, M.; Larson, J. R.; Liberton, D. K.; Rolian, C.; Smith, F. J.; Kimwaga, E.; Gilyoma, J.; Lukowiak, K. D.; Spritz, R. A.; Hallgrimsson, B. | 2014 | | Facial morphometrics of children with non-syndromic orofacial clefts in Tanzania | | unrelated topic |
| 1. Sforza, C.; Dolci, C.; Rosati, R.; de Menezes, M.; Pisoni, L.; Ferrario, V. F.; Elamin, F. | 2014 | | Facial soft-tissue volumes in adult Northern Sudanese individuals with Down syndrome | | Duplicate |
| 1. Sforza, C.; Dolci, C.; Rosati, R.; de Menezes, M.; Pisoni, L.; Ferrario, V. F.; Elamin, F. | 2014 | | Facial soft-tissue volumes in adult Northern Sudanese individuals with Down syndrome | | unrelated topic |
| 1. Louw, B.; Shibambu, M.; Roemer, K. | 2006 | | Facilitating cleft palate team participation of culturally diverse families in South Africa | | unrelated topic |
| 1. Ogunkunle, T. O.; Gabriel, T. Y.; Bello, S. O.; Abdullahi, Y.; Bulus, J.; Ozhe, S. I.; Imam, A. | 2021 | | Facility-Based Newborn Deaths at a Referral Tertiary Hospital in North-Central Nigeria during the Sustainable Development Goal Era: A Retrospective Cohort Analysis | | Duplicate |
| 1. Ogunkunle, T. O.; Gabriel, T. Y.; Bello, S. O.; Abdullahi, Y.; Bulus, J.; Ozhe, S. I.; Imam, A. | 2021 | | Facility-Based Newborn Deaths at a Referral Tertiary Hospital in North-Central Nigeria during the Sustainable Development Goal Era: A Retrospective Cohort Analysis | | unrelated topic |
| 1. Warf, B. C.; Wright, E. J.; Kulkarni, A. V. | 2011 | | Factors affecting survival of infants with myelomeningocele in southeastern Uganda | | unrelated topic |
| 1. Osifo, D. O.; Oriaifo, I. A. | 2008 | | Factors affecting the management and outcome of neonatal surgery in Benin City, Nigeria | | unrelated topic |
| 1. Taye, M.; Afework, M.; Fantaye, W.; Diro, E.; Worku, A. | 2018 | | Factors associated with congenital anomalies in Addis Ababa and the Amhara Region, Ethiopia: a case-control study | | doesn’t include primary outcome |
| 1. Alsaied, T.; Bokma, J. P.; Engel, M. E.; Kuijpers, J. M.; Hanke, S. P.; Zuhlke, L.; Zhang, B.; Veldtman, G. R. | 2017 | | Factors associated with long-term mortality after Fontan procedures: a systematic review | | unrelated topic |
| 1. Kishimba, R. S.; Mpembeni, R.; Mghamba, J. | 2015 | | Factors associated with major structural birth defects among newborns delivered at Muhimbili National Hospital and Municipal Hospitals in Dar Es Salaam, Tanzania 2011 - 2012 | | unrelated topic |
| 1. Mulu, G. B.; Atinafu, B. T.; Tarekegn, F. N.; Adane, T. D.; Tadese, M.; Wubetu, A. D.; Kebede, W. M. | 2021 | | Factors Associated With Neural Tube Defects Among Newborns Delivered at Debre Berhan Specialized Hospital, North Eastern Ethiopia, 2021. Case-Control Study | | unrelated topic |
| 1. Tegene, D.; Mannekulih, E. | 2023 | | Factors Associated with Neural Tube Defects among Women Who Gave Birth at Adama Hospital Medical College, Adama, Ethiopia: A Case Control Study | | Duplicate |
| 1. Tegene, D.; Mannekulih, E. | 2023 | | Factors Associated with Neural Tube Defects among Women Who Gave Birth at Adama Hospital Medical College, Adama, Ethiopia: A Case Control Study | | unrelated topic |
| 1. Manniën, J.; de Jonge, A.; Cornel, M. C.; Spelten, E.; Hutton, E. K. | 2014 | | Factors associated with not using folic acid supplements preconceptionally | | unrelated topic |
| 1. Getiye, Y.; Fantahun, M. | 2017 | | Factors associated with perinatal mortality among public health deliveries in Addis Ababa, Ethiopia, an unmatched case control study | | unrelated topic |
| 1. Tefera, E.; Gedlu, E.; Nega, B.; Tadesse, B. T.; Chanie, Y.; Dawoud, A.; Moges, F. H.; Bezabih, A.; Moges, T.; Centella, T.; Marianeschi, S.; Coca, A.; Collado, R.; Kassa, M. W.; Johansson, S.; van Doorn, C.; Barber, B. J.; Teodori, M. | 2019 | | Factors associated with perioperative mortality in children and adolescents operated for tetralogy of Fallot: A sub-Saharan experience | | unrelated topic |
| 1. Kelkay, B.; Omer, A.; Teferi, Y.; Moges, Y. | 2019 | | Factors Associated with Singleton Preterm Birth in Shire Suhul General Hospital, Northern Ethiopia, 2018 | | unrelated topic |
| 1. Mbabazi, L.; Nabaggala, M. S.; Kiwanuka, S.; Kiguli, J.; Laker, E.; Kiconco, A.; Okoboi, S.; Lamorde, M.; Castelnuovo, B. | 2022 | | Factors associated with uptake of contraceptives among HIV positive women on dolutegravir based anti-retroviral treatment-a cross sectional survey in urban Uganda | | unrelated topic |
| 1. Komolafe, E. O.; Komolafe, M. A.; Adeolu, A. A. | 2008 | | Factors implicated for late presentations of gross congenital anomaly of the nervous system in a developing nation | | unrelated topic |
| 1. Kalis, N. N.; Pieper, C.; van der Merwe, P. L.; Nel, E. D. | 2001 | | Factors influencing successful closure with indomethacin of the patent ductus arteriosus in premature infants | | unrelated topic |
| 1. Loh, T. F.; Ang, Y. H.; Wong, Y. K.; Tan, H. Y. | 1973 | | Fallot's tetralogy–natural history | | Year of publication |
| 1. Moore, S. W.; Rode, H.; Millar, A. J.; Albertyn, R.; Cywes, S. | 1991 | | Familial aspects of Hirschsprung's disease | | Year of publication |
| 1. Moore, S. W.; Zaahl, M. | 2010 | | Familial associations in medullary thyroid carcinoma with Hirschsprung disease: the role of the RET-C620 "Janus" genetic variation | | unrelated topic |
| 1. Durowaye, M.; Adeboye, M.; Yahaya-Kongoila, S.; Adaje, A.; Adesiyun, O.; Ernest, S. K.; Mokuolu, O. A.; Adegboye, A. | 2011 | | Familial ectrodactyly syndrome in a nigerian child: a case report | | bookSection |
| 1. Wiegman, A.; Gidding, S. S.; Watts, G. F.; Chapman, M. J.; Ginsberg, H. N.; Cuchel, M.; Ose, L.; Averna, M.; Boileau, C.; Borén, J.; Bruckert, E.; Catapano, A. L.; Defesche, J. C.; Descamps, O. S.; Hegele, R. A.; Hovingh, G. K.; Humphries, S. E.; Kovanen, P. T.; Kuivenhoven, J. A.; Masana, L.; Nordestgaard, B. G.; Pajukanta, P.; Parhofer, K. G.; Raal, F. J.; Ray, K. K.; Santos, R. D.; Stalenhoef, A. F.; Steinhagen-Thiessen, E.; Stroes, E. S.; Taskinen, M. R.; Tybjærg-Hansen, A.; Wiklund, O. | 2015 | | Familial hypercholesterolaemia in children and adolescents: gaining decades of life by optimizing detection and treatment | | unrelated topic |
| 1. Delport, R. | 2009 | | Familial hypercholesterolaemia in South Africans: tracking findings and developments over time - with reference to : prevalence of hypercholesterolaemia in young Afrikaners with myocardial infarction. Ischaemic heart disease risk factors | | unrelated topic |
| 1. Grace, H. J.; Harris, E. | 1970 | | Familial occurrence of an abnormal Y chromosome | | Year of publication |
| 1. Duvie, S. O.; Evbuomwan, I.; Scott-Emuakpor, A. B.; Kadiri, I. | 1990 | | Familial occurrence of cryptorchidism | | Year of publication |
| 1. Hitzeroth, H. W.; de Villiers, L. S.; Groeneveld, H. T. | 1992 | | Family history as a risk factor for coronary heart disease in South African families | | Year of publication |
| 1. MacLean, J. E.; Mahant, S.; Bitnun, A.; Read, S.; Capra, M.; Solomon, M. | 2006 | | Family matters: a twelve-year-old male with respiratory symptoms, cachexia and clubbing | | unrelated topic |
| 1. Basson, A. L.; Essop, M. R.; Libhaber, E.; Peters, F. | 2020 | | Family screening in black patients with isolated left ventricular non-compaction: the Chris Hani Baragwanath experience | | unrelated topic |
| 1. Wainstein, T.; Kerr, R.; Mitchell, C. L.; Madaree, S.; Essop, F. B.; Vorster, E.; Wainwright, R.; Poole, J.; Krause, A. | 2013 | | Fanconi anaemia in black South African patients heterozygous for the FANCG c.637-643delTACCGCC founder mutation | | unrelated topic |
| 1. Larin, ME | 2015 | | Fanconi Anemia Signalling and Mus81-Eme1 operate in distinct pathways to facilitate in utero development and crosslink repair | | book |
| 1. Rosendorff, J.; Bernstein, R.; Macdougall, L.; Jenkins, T. | 1987 | | Fanconi anemia: another disease of unusually high prevalence in the Afrikaans population of South Africa | | Year of publication |
| 1. Iezzi, F.; Di Summa, M.; Sarto, P. D.; Munene, J. | 2019 | | Fast track extubation in paediatric cardiothoracic surgery in developing countries | | bookSection |
| 1. McGrath, K.; Reid, D. J.; Guatelli-Steinberg, D.; Arbenz-Smith, K.; El Zaatari, S.; Fatica, L. M.; Kralick, A. E.; Cranfield, M. R.; Stoinski, T. S.; Bromage, T. G.; Mudakikwa, A.; McFarlin, S. C. | 2019 | | Faster growth corresponds with shallower linear hypoplastic defects in great ape canines | | unrelated topic |
| 1. van Wijck, S. F.; Oomen, A. M.; van der Heide, H. J. | 2015 | | Feasibility and barriers of treating clubfeet in four countries | | unrelated topic |
| 1. Van Niekerk, A. M.; Cullis, R. M.; Linley, L. L.; Zühlke, L. | 2016 | | Feasibility of Pulse Oximetry Pre-discharge Screening Implementation for detecting Critical Congenital heart Lesions in newborns in a secondary level maternity hospital in the Western Cape, South Africa: The 'POPSICLe' study | | unrelated topic |
| 1. Ouédraogo, A. S.; Sanou, S.; Kissou, A.; Poda, A.; Aberkane, S.; Bouzinbi, N.; Nacro, B.; Ouédraogo, R.; Van De Perre, P.; Carriere, C.; Decré, D.; Jean-Pierre, H.; Godreuil, S. | 2017 | | Fecal Carriage of Enterobacteriaceae Producing Extended-Spectrum Beta-Lactamases in Hospitalized Patients and Healthy Community Volunteers in Burkina Faso | | unrelated topic |
| 1. Hazirolan, G.; Mumcuoglu, I.; Altan, G.; Özmen, B. B.; Aksu, N.; Karahan, Z. C. | 2018 | | Fecal carriage of extended-spectrum beta-lactamase and ampc beta-lactamase-producing enterobacteriaceae in a turkish community | | unrelated topic |
| 1. Ouchar Mahamat, O.; Tidjani, A.; Lounnas, M.; Hide, M.; Benavides, J.; Somasse, C.; Ouedraogo, A. S.; Sanou, S.; Carrière, C.; Bañuls, A. L.; Jean-Pierre, H.; Dumont, Y.; Godreuil, S. | 2019 | | Fecal carriage of extended-spectrum β-lactamase-producing Enterobacteriaceae in hospital and community settings in Chad | | Duplicate |
| 1. Ouchar Mahamat, O.; Tidjani, A.; Lounnas, M.; Hide, M.; Benavides, J.; Somasse, C.; Ouedraogo, A. S.; Sanou, S.; Carrière, C.; Bañuls, A. L.; Jean-Pierre, H.; Dumont, Y.; Godreuil, S. | 2019 | | Fecal carriage of extended-spectrum β-lactamase-producing Enterobacteriaceae in hospital and community settings in Chad | | unrelated topic |
| 1. Ize-Iyamu, I. N.; Saheeb, B. D. | 2011 | | Feeding intervention in cleft lip and palate babies: a practical approach to feeding efficiency and weight gain | | unrelated topic |
| 1. Lazarus, J.; van den Heever, A.; Kortekaas, B.; Alexander, A. | 2012 | | Female epispadias managed by bladder neck plication via a perineal approach | | unrelated topic |
| 1. Naidoo, S.; Chikte, U.; Laubscher, R.; Lombard, C. | 2005 | | Fetal alcohol syndrome: anthropometric and oral health status | | unrelated topic |
| 1. Malan, A. F.; Vader, C.; Knutzen, V. K. | 1975 | | Fetal and early neonatal mortality | | Year of publication |
| 1. Osanyin, G. E.; Odeseye, A. K.; Okojie, O. O.; Akinajo, O. R.; Okusanya, B. O. | 2019 | | Fetal Congenital Anomaly in Tertiary Hospital in Lagos, South-West Nigeria: A Review of Presentation and its Outcome | | unrelated topic |
| 1. Sium, A. F.; Abdosh, A. A.; Gudu, W. | 2023 | | Fetal echocardiography in a low-income setting: relying on local Maternal-fetal medicine experts for detection of fetal cardiac anomalies | | unrelated topic |
| 1. Reinisch, J. M. | 1974 | | Fetal hormones, the brain, and human sex differences: a heuristic, integrative review of the recent literature | | Year of publication |
| 1. Vally, I. M.; Altini, M. | 1990 | | Fibromatoses of the oral and paraoral soft tissues and jaws. Review of the literature and report of 12 new cases | | Year of publication |
| 1. Wilhelm, M. J.; Ruschitzka, F.; Flammer, A. J.; Bettex, D.; Turina, M. I.; Maisano, F. | 2020 | | Fiftieth anniversary of the first heart transplantation in Switzerland in the context of the worldwide history of heart transplantation | | unrelated topic |
| 1. Donaghy, K; Isern, MTI | 2012 | | Films in Health Sciences Education. Learning through moving images | | book |
| 1. Sassetti, M.; Zé-Zé, L.; Franco, J.; Cunha, J. D.; Gomes, A.; Tomé, A.; Alves, M. J. | 2018 | | First case of confirmed congenital Zika syndrome in continental Africa | | unrelated topic |
| 1. Betz, O.; Maurer, A.; Verheyden, A. N.; Schmitt, C.; Kowalik, T.; Braun, J.; Grunwald, I.; Hartwig, A.; Neuenfeldt, M. | 2016 | | First protein and peptide characterization of the tarsal adhesive secretions in the desert locust, Schistocerca gregaria, and the Madagascar hissing cockroach, Gromphadorhina portentosa | | unrelated topic |
| 1. Dellicour, S.; Sevene, E.; McGready, R.; Tinto, H.; Mosha, D.; Manyando, C.; Rulisa, S.; Desai, M.; Ouma, P.; Oneko, M.; Vala, A.; Rupérez, M.; Macete, E.; Menéndez, C.; Nakanabo-Diallo, S.; Kazienga, A.; Valéa, I.; Calip, G.; Augusto, O.; Genton, B.; Njunju, E. M.; Moore, K. A.; d'Alessandro, U.; Nosten, F.; Ter Kuile, F.; Stergachis, A. | 2017 | | First-trimester artemisinin derivatives and quinine treatments and the risk of adverse pregnancy outcomes in Africa and Asia: A meta-analysis of observational studies | | unrelated topic |
| 1. Abich, Y.; Mihiret, T.; Yihunie Akalu, T.; Gashaw, M.; Janakiraman, B. | 2020 | | Flatfoot and associated factors among Ethiopian school children aged 11 to 15 years: A school-based study | | unrelated topic |
| 1. Shaheen, S.; Mursal, H.; Rabih, M.; Johari, A. | 2015 | | Flexor digitorum accessorius longus muscle in resistant clubfoot patients: introduction of a new sign predicting its presence | | unrelated topic |
| 1. Deleon, V. B. | 2007 | | Fluctuating asymmetry and stress in a medieval Nubian population | | unrelated topic |
| 1. Moola, M. H. | 1996 | | Fluoridation in South Africa | | Year of publication |
| 1. Demelash, H.; Beyene, A.; Abebe, Z.; Melese, A. | 2019 | | Fluoride concentration in ground water and prevalence of dental fluorosis in Ethiopian Rift Valley: systematic review and meta-analysis | | un+B2596:B2609related topic |
| 1. Kerdoun, M. A.; Mekhloufi, S.; Adjaine, O. E. K.; Bechki, Z.; Gana, M.; Belkhalfa, H. | 2022 | | Fluoride concentrations in drinking water and health risk assessment in the south of Algeria | | unrelated topic |
| 1. Rango, T.; Vengosh, A.; Jeuland, M.; Tekle-Haimanot, R.; Weinthal, E.; Kravchenko, J.; Paul, C.; McCornick, P. | 2014 | | Fluoride exposure from groundwater as reflected by urinary fluoride and children's dental fluorosis in the Main Ethiopian Rift Valley | | unrelated topic |
| 1. Tobayiwa, C.; Musiyambiri, M.; Chironga, L.; Mazorodze, O.; Sapahla, S. | 1991 | | Fluoride levels and dental fluorosis in two districts in Zimbabwe | | Year of publication |
| 1. Solanki, Y. S.; Agarwal, M.; Gupta, A. B.; Gupta, S.; Shukla, P. | 2022 | | Fluoride occurrences, health problems, detection, and remediation methods for drinking water: A comprehensive review | | unrelated topic |
| 1. Manji, F.; Baelum, V.; Fejerskov, O. | 1986 | | Fluoride, altitude and dental fluorosis | | Duplicate |
| 1. Manji, F.; Baelum, V.; Fejerskov, O. | 1986 | | Fluoride, altitude and dental fluorosis | | Duplicate |
| 1. Manji, F.; Kapila, S. | 1986 | | Fluorides and fluorosis in Kenya. Part I: The occurrence of fluorides | | Duplicate |
| 1. Manji, F.; Kapila, S. | 1986 | | Fluorides and fluorosis in Kenya. Part I: The occurrence of fluorides | | Year of publication |
| 1. Manji, F.; Kapila, S. | 1986 | | Fluorides and fluorosis in Kenya. Part II: The occurrence of dental and skeletal fluorosis | | Duplicate |
| 1. Manji, F.; Kapila, S. | 1986 | | Fluorides and fluorosis in Kenya. Part III: Fluorides, fluorosis and dental caries | | Duplicate |
| 1. Lewis, H. A.; Chikte, U. M.; Butchart, A. | 1992 | | Fluorosis and dental caries in schoolchildren from rural areas with about 9 and 1 ppm F in the water supplies | | Year of publication |
| 1. Qureshi, B. A. | 1977 | | Fluorosis in Kenya | | Year of publication |
| 1. El-Nadeef, M. A.; Honkala, E. | 1998 | | Fluorosis in relation to fluoride levels in water in central Nigeria | | Year of publication |
| 1. Grech, P.; Latham, M. C. | 1964 | | FLUOROSIS IN THE NORTHERN REGION OF TANGANYIKA | | Year of publication |
| 1. Opinya, G. N.; Valderhaug, J.; Birkeland, J. M.; Løkken, P. | 1991 | | Fluorosis of deciduous teeth and first permanent molars in a rural Kenyan community | | Duplicate |
| 1. Opinya, G. N.; Valderhaug, J.; Birkeland, J. M.; Løkken, P. | 1991 | | Fluorosis of deciduous teeth and first permanent molars in a rural Kenyan community | | Year of publication |
| 1. Berndt, Ch; Meller, Ch; Poppe, D.; Splieth Ch, H. | 2010 | | Fluorosis, caries and oral hygiene in schoolchildren on the Ombili Foundation in Namibia | | unrelated topic |
| 1. Mocumbi, A. O. | 2013 | | Focus on non-communicable diseases: an important agenda for the African continent | | unrelated topic |
| 1. Chen, L.; Mutabandama, Y.; McCall, N.; Umuhoza, C. | 2022 | | Focused Cardiac Ultrasound Findings in Children Presenting With Shock to a Tertiary Care Hospital in Rwanda | | unrelated topic |
| 1. Adesiyun, A. G.; Eka, A.; Samaila, M. O. | 2007 | | Foetal chondrodysplasia: intrauterine diagnosis | | unrelated topic |
| 1. Singh, S.; Chukwunyere, D. N.; Omembelede, J.; Onankpa, B. | 2015 | | Foetal congenital anomalies: An experience from a tertiary health institution in north-west nigeria (2011-2013) | | unrelated |
| 1. Ubbink, J. B.; Christianson, A.; Bester, M. J.; Van Allen, M. I.; Venter, P. A.; Delport, R.; Blom, H. J.; van der Merwe, A.; Potgieter, H.; Vermaak, W. J. | 1999 | | Folate status, homocysteine metabolism, and methylene tetrahydrofolate reductase genotype in rural South African blacks with a history of pregnancy complicated by neural tube defects | | Year of publication |
| 1. Sayed, A. R.; Nixon, J.; Bourne, D. E. | 2006 | | Folic acid awareness among women of reproductive age in Cape Town | | unrelated topic |
| 1. van Eijsden, M.; van der Wal, M. F.; Bonsel, G. J. | 2006 | | Folic acid knowledge and use in a multi-ethnic pregnancy cohort: the role of language proficiency | | unrelated topic |
| 1. Yesehak, B.; Dorsey, A.; Zewdie, K.; Kancherla, V.; Ashagre, Y. | 2023 | | Folic acid prescription practice for high-risk prevention of spina bifida at a tertiary care hospital in Addis Ababa, Ethiopia | | unrelated topic |
| 1. Toivonen, K. I.; Lacroix, E.; Flynn, M.; Ronksley, P. E.; Oinonen, K. A.; Metcalfe, A.; Campbell, T. S. | 2018 | | Folic acid supplementation during the preconception period: A systematic review and meta-analysis | | unrelated topic |
| 1. Dessie, M. A.; Zeleke, E. G.; Workie, S. B.; Berihun, A. W. | 2017 | | Folic acid usage and associated factors in the prevention of neural tube defects among pregnant women in Ethiopia: cross-sectional study | | unrelated topic |
| 1. Bafor, A.; Chibuzom, C. N. | 2020 | | Foot and ankle abnormalities among a cohort of Nigerian school children: an epidemiological study | | unrelated topic |
| 1. Motebejane, M. S.; Choi, I. S. | 2018 | | Foramen Magnum Dural Arteriovenous Fistulas: Clinical Presentations and Treatment Outcomes, A Case-Series of 12 Patients | | unrelated topic |
| 1. Udayakumaran, S.; Onyia, C. U.; Kumar, R. K. | 2017 | | Forgotten? Not Yet. Cardiogenic Brain Abscess in Children: A Case Series-Based Review | | unrelated topic |
| 1. Alamgholiloo, H.; Rostamnia, S.; Hassankhani, A.; Liu, X.; Eftekhari, A.; Hasanzadeh, A.; Zhang, K.; Karimi-Maleh, H.; Khaksar, S.; Varma, R. S.; Shokouhimehr, M. | 2020 | | Formation and stabilization of colloidal ultra-small palladium nanoparticles on diamine-modified Cr-MIL-101: Synergic boost to hydrogen production from formic acid | | unrelated topic |
| 1. Centeno Tablante, E.; Pachón, H.; Guetterman, H. M.; Finkelstein, J. L. | 2019 | | Fortification of wheat and maize flour with folic acid for population health outcomes | | unrelated topic |
| 1. Torrington, M.; Viljoen, D. L. | 1991 | | Founder effect in 20 Afrikaner kindreds with pseudoxanthoma elasticum | | Year of publication |
| 1. Rode, H.; Fieggen, A. G.; Brown, R. A.; Cywes, S.; Davies, M. R.; Hewitson, J. P.; Hoffman, E. B.; Jee, L. D.; Lawrenson, J.; Mann, M. D.; Matthews, L. S.; Millar, A. J.; Numanoglu, A.; Peter, J. C.; Thomas, J.; Wainwright, H. | 2006 | | Four decades of conjoined twins at Red Cross Children's Hospital–lessons learned | | unrelated topic |
| 1. Ira, A. V. B.; Krasteva, D.; Kouadjo, F.; Roger, F.; Bellet, V.; Koffi, D.; Pottier, C.; Toure, O. A.; Drakulovski, P.; Djaman, A. J.; Ranque, S.; Bertout, S. | 2023 | | Four uncommon clinical fungi, Lodderomyces elongisporus, Kodamaea ohmeri, Cyberlindnera fabianii and Wickerhamomyces anomalus, isolated in superficial samples from Côte d'Ivoire | | unrelated topic |
| 1. Njemanze, P. C.; Beck, O. J.; Gomez, C. R.; Horenstein, S. | 1991 | | Fourier analysis of the cerebrovascular system | | Year of publication |
| 1. Getahun, S.; Masresha, S.; Zenebe, E.; Laeke, T.; Tirsit, A. | 2021 | | Four-Year Treatment Outcomes of Children Operated for Neural Tube Defect in Addis Ababa, Ethiopia: A Retrospective Study | | unrelated topic |
| 1. Goldman, A.; Krause, A.; Jenkins, T. | 1997 | | Fragile X syndrome occurs in the South African black population | | Year of publication |
| 1. Smart, R. D. | 1992 | | Fragile X syndrome: as common as first thought? | | Year of publication |
| 1. Piras, I.; Falchi, A.; Moral, P.; Melis, A.; Giovannoni, L.; Paoli, G.; Calò, C.; Vona, G.; Varesi, L. | 2008 | | Frequencies of promoter pentanucleotide (TTTTA)n of CYP11A gene in European and North African populations | | unrelated topic |
| 1. Too, R. J.; Gitao, G. C.; Bebora, L. C.; Mollenkopf, D. F.; Kariuki, S. M.; Wittum, T. E. | 2023 | | Frequency and diversity of carbapenemase-producing Enterobacterales recovered from untreated wastewater impacted by selective media containing cefotaxime and meropenem in Ohio, USA | | unrelated topic |
| 1. Yersin, C.; Bovet, P.; Wauters, J. P.; Schorderet, D. F.; Pescia, G.; Paccaud, F. | 1997 | | Frequency and impact of autosomal dominant polycystic kidney disease in the Seychelles (Indian Ocean) | | Year of publication |
| 1. Yusuf, Mohamed Farah and Icen, Yahya Kemal and Ahmed, Said Abdirahman and Osman, Abdirahman Abdikadir and Hussein, Abdinafic Mohamud | 2021 | | Frequency and pattern of congenital heart diseases among children in a Tertiary Hospital in Mogadishu, Somali, 2019 | | Included |
| 1. Clur, S. A. | 2006 | | Frequency and severity of rheumatic heart disease in the catchment area of Gauteng hospitals, 1993-1995 | | unrelated topic |
| 1. Kaheel, H.; Breß, A.; Hassan, M. A.; Shah, A. A.; Amin, M.; Bakhit, Y. H. Y.; Kniper, M. | 2017 | | Frequency of c.35delG Mutation in GJB2 Gene (Connexin 26) in Syrian Patients with Nonsyndromic Hearing Impairment | | unrelated topic |
| 1. van der Horst, R. L. | 1984 | | Frequency of different types of heart disease in black children in South Africa | | Year of publication |
| 1. Kaimbo Wa Kaimbo, D. | 2016 | | Frequency of prepapillary vascular loops in Congolese patients | | unrelated topic |
| 1. Akinboro, A. O.; Onayemi, O.; Mejiuni, A. D. | 2014 | | Frequency, pattern, and extent of skin diseases in relation to CD4+ cell count among adults with human immunodeficiency virus infection or acquired immunodeficiency syndrome in Osogbo, southwestern Nigeria | | unrelated topic |
| 1. Lu, N.; Zhao, L.; Du, Q.; Liu, Y.; Oprescu, F. I.; Morcuende, J. A. | 2010 | | From cutting to casting: impact and initial barriers to the Ponseti method of clubfoot treatment in China | | unrelated topic |
| 1. Segal, N. L. | 2023 | | From the Library of the Late Irving I. Gottesman: Memories and Treasures/Twin Research Reviews: Twin Study of Callous-Unemotional Traits; Depressive Symptoms in Prospective Chinese Twin Mothers; Twins With Sagittal Suture Craniosynostosis; Creative Expressiveness and Educational Achievement/Media Reports: Male-Female Twin Holocaust Survivors; Nontuplets Born in Mali; Indian Twins Marry Same Man; Twins Born From Longest-Frozen Embryos; Infant Twin Abduction; Twins Born in Different Years | | unrelated topic |
| 1. De Klerk, D. J.; De Villiers, J. C. | 1973 | | Frontal encephaloceles | | Year of publication |
| 1. Zabsonre, D. S.; Kabre, A.; Haro, Y. | 2015 | | Frontoethmoidal cephalocele: our experience of eleven cases managed surgically | | unrelated topic |
| 1. Smit, C. S.; Zeeman, B. J.; Smith, R. M.; de, V. Cluver P. F. | 1993 | | Frontoethmoidal meningoencephaloceles: a review of 14 consecutive patients | | Year of publication |
| 1. Aymé, S.; Julian, C.; Gambarelli, D.; Mariotti, B.; Luciani, A.; Sudan, N.; Maurin, N.; Philip, N.; Serville, F.; Carles, D.; al, et | 1989 | | Fryns syndrome: report on 8 new cases | | Year of publication |
| 1. Domijan, A. M. | 2012 | | Fumonisin B(1): a neurotoxic mycotoxin | | unrelated topic |
| 1. Marasas, W. F.; Riley, R. T.; Hendricks, K. A.; Stevens, V. L.; Sadler, T. W.; Gelineau-van Waes, J.; Missmer, S. A.; Cabrera, J.; Torres, O.; Gelderblom, W. C.; Allegood, J.; Martínez, C.; Maddox, J.; Miller, J. D.; Starr, L.; Sullards, M. C.; Roman, A. V.; Voss, K. A.; Wang, E.; Merrill, Jr., A. H. | 2004 | | Fumonisins disrupt sphingolipid metabolism, folate transport, and neural tube development in embryo culture and in vivo: a potential risk factor for human neural tube defects among populations consuming fumonisin-contaminated maize | | unrelated topic |
| 1. Rice, G. I.; Del Toro Duany, Y.; Jenkinson, E. M.; Forte, G. M.; Anderson, B. H.; Ariaudo, G.; Bader-Meunier, B.; Baildam, E. M.; Battini, R.; Beresford, M. W.; Casarano, M.; Chouchane, M.; Cimaz, R.; Collins, A. E.; Cordeiro, N. J.; Dale, R. C.; Davidson, J. E.; De Waele, L.; Desguerre, I.; Faivre, L.; Fazzi, E.; Isidor, B.; Lagae, L.; Latchman, A. R.; Lebon, P.; Li, C.; Livingston, J. H.; Lourenço, C. M.; Mancardi, M. M.; Masurel-Paulet, A.; McInnes, I. B.; Menezes, M. P.; Mignot, C.; O'Sullivan, J.; Orcesi, S.; Picco, P. P.; Riva, E.; Robinson, R. A.; Rodriguez, D.; Salvatici, E.; Scott, C.; Szybowska, M.; Tolmie, J. L.; Vanderver, A.; Vanhulle, C.; Vieira, J. P.; Webb, K.; Whitney, R. N.; Williams, S. G.; Wolfe, L. A.; Zuberi, S. M.; Hur, S.; Crow, Y. J. | 2014 | | Gain-of-function mutations in IFIH1 cause a spectrum of human disease phenotypes associated with upregulated type I interferon signaling | | unrelated topic |
| 1. Bejiga, G.; Ahmed, Z. | 2022 | | Gangrenous Meckel's diverticulum with small bowel obstruction mimicking complicated appendicitis: 'Case report' | | unrelated topic |
| 1. Kibwana, U. O.; Manyahi, J.; Sandnes, H. H.; Blomberg, B.; Mshana, S. E.; Langeland, N.; Moyo, S. J. | 2022 | | Gastrointestinal colonization of extended-spectrum beta-lactamase-producing bacteria among children below five years of age hospitalized with fever in Dar es Salaam, Tanzania | | unrelated topic |
| 1. Olajide, A. R.; Yisau, A. A.; Abdulraseed, N. A.; Kashim, I. O.; Olaniyi, A. J.; Morohunfade, A. O. | 2010 | | Gastrointestinal duplications: Experience in seven children and a review of the literature | | unrelated topic |
| 1. Agugua, N. E.; Nwako, F. A. | 1990 | | Gastroschisis a fifteen-year experience | | Year of publication |
| 1. Amado, V.; Dias, I.; Filipe, M.; DeUgarte, D. A. | 2023 | | Gastroschisis in Mozambique: current status and priorities for improving care from Hospital Central de Maputo, the largest and referral hospital of the country | | unrelated topic |
| 1. Fatona, O.; Opashola, K.; Faleye, A.; Adeyanju, T.; Adekanmbi, A.; Etiubon, E.; Jesuyajolu, D.; Zubair, A. | 2023 | | Gastroschisis in Sub-Saharan Africa: a scoping review of the prevalence, management practices, and associated outcomes | | unrelated topic |
| 1. Wesonga, A. S.; Fitzgerald, T. N.; Kabuye, R.; Kirunda, S.; Langer, M.; Kakembo, N.; Ozgediz, D.; Sekabira, J. | 2016 | | Gastroschisis in Uganda: Opportunities for improved survival | | unrelated topic |
| 1. Borgstein, E. S. | 2001 | | Gastroschisis minor | | unrelated topic |
| 1. Manson, J.; Ameh, E.; Canvassar, N.; Chen, T.; den Hoeve, A. V.; Lever, F.; Hesse, A.; Millar, A.; Emil, S.; Ade-Ajayi, N. | 2012 | | Gastroschisis: a multi-centre comparison of management and outcome | | unrelated topic |
| 1. Sekabira, J.; Hadley, G. P. | 2009 | | Gastroschisis: a third world perspective | | unrelated topic |
| 1. Ford, K.; Poenaru, D.; Moulot, O.; Tavener, K.; Bradley, S.; Bankole, R.; Tshifularo, N.; Ameh, E.; Alema, N.; Borgstein, E.; Hickey, A.; Ade-Ajayi, N. | 2016 | | Gastroschisis: Bellwether for neonatal surgery capacity in low resource settings? | | unrelated topic |
| 1. Doshi, H.; Shukla, S.; Patel, S.; Bhatt, P.; Bhatt, N.; Anim-Koranteng, C.; Ameley, A.; Biney, B.; Dapaah-Siakwan, F.; Donda, K. | 2022 | | Gastrostomy Tube Placement and Resource Use in Neonatal Hospitalizations With Down Syndrome | | Duplicate |
| 1. Doshi, H.; Shukla, S.; Patel, S.; Bhatt, P.; Bhatt, N.; Anim-Koranteng, C.; Ameley, A.; Biney, B.; Dapaah-Siakwan, F.; Donda, K. | 2022 | | Gastrostomy Tube Placement and Resource Use in Neonatal Hospitalizations With Down Syndrome | | unrelated topic |
| 1. Dormehl, I. C.; van Gelder, A. L.; Hugo, N.; Weller, R.; Stanton, J. J.; Beverley, G. H.; Clausen, M. | 1993 | | Gated blood pool SPECT and phase analysis to assess simulated Wolff-Parkinson-White syndrome in the baboon | | Year of publication |
| 1. Rebelo, E.; Szabo, C. P.; Pitcher, G. | 2008 | | Gender assignment surgery on children with disorders of sex development: a case report and discussion from South Africa | | unrelated topic |
| 1. MA Njelekela, R Mpembeni, A Muhihi… | 2009 | | Gender-related differences in the prevalence of cardiovascular disease risk factors and their correlates in urban Tanzania | | unrelated topic |
| 1. Gelineau-van Waes, J.; van Waes, M. A.; Hallgren, J.; Hulen, J.; Bredehoeft, M.; Ashley-Koch, A. E.; Krupp, D.; Gregory, S. G.; Stessman, H. A. | 2023 | | Gene-nutrient interactions that impact magnesium homeostasis increase risk for neural tube defects in mice exposed to dolutegravir | | unrelated topic |
| 1. Hasan, B. S.; Rasheed, M. A.; Wahid, A.; Kumar, R. K.; Zuhlke, L. | 2021 | | Generating Evidence From Contextual Clinical Research in Low- to Middle Income Countries: A Roadmap Based on Theory of Change | | unrelated topic |
| 1. Ogunye, O.; Adedinsewo, A. | 1987 | | Genetic content of pediatric practice in the tropics | | Year of publication |
| 1. op't Hof, J. | 1977 | | Genetic disease–risks of occurrence and recurrence | | Year of publication |
| 1. Salih, MAM | 2010 | | Genetic disorders in Sudan | | unrelated topic |
| 1. Winship, W. S.; Beighton, P. | 2011 | | Genetic disorders in the Indian community of South Africa | | unrelated topic |
| 1. Eachus, H.; Zaucker, A.; Oakes, J. A.; Griffin, A.; Weger, M.; Güran, T.; Taylor, A.; Harris, A.; Greenfield, A.; Quanson, J. L.; Storbeck, K. H.; Cunliffe, V. T.; Müller, F.; Krone, N. | 2017 | | Genetic Disruption of 21-Hydroxylase in Zebrafish Causes Interrenal Hyperplasia | | unrelated topic |
| 1. Hamdi, Y.; Jerbi, M.; Romdhane, L.; Ben Rekaya, M.; El Benna, H.; Chouchane, L.; Boubaker, M. S.; Abdelhak, S.; Yacoub-Youssef, H. | 2020 | | Genetic diversity and functional effect of common polymorphisms in genes involved in the first heterodimeric complex of the Nucleotide Excision Repair pathway | | unrelated topic |
| 1. Peprah, E. K.; Allen, E. G.; Williams, S. M.; Woodard, L. M.; Sherman, S. L. | 2010 | | Genetic diversity of the fragile X syndrome gene (FMR1) in a large Sub-Saharan West African population | | Duplicate |
| 1. Peprah, E. K.; Allen, E. G.; Williams, S. M.; Woodard, L. M.; Sherman, S. L. | 2010 | | Genetic diversity of the fragile X syndrome gene (FMR1) in a large Sub-Saharan West African population | | unrelated topic |
| 1. Winship, I. M.; Connor, J. M.; Beighton, P. H. | 1990 | | Genetic heterogeneity in tuberous sclerosis: phenotypic correlations | | Year of publication |
| 1. Moore, S. W. | 2017 | | Genetic impact on the treatment & management of Hirschsprung disease | | unrelated topic |
| 1. Crotti, L.; Lahtinen, A. M.; Spazzolini, C.; Mastantuono, E.; Monti, M. C.; Morassutto, C.; Parati, G.; Heradien, M.; Goosen, A.; Lichtner, P.; Meitinger, T.; Brink, P. A.; Kontula, K.; Swan, H.; Schwartz, P. J. | 2016 | | Genetic Modifiers for the Long-QT Syndrome: How Important Is the Role of Variants in the 3' Untranslated Region of KCNQ1? | | unrelated topic |
| 1. Butali, A.; Mossey, P. A.; Adeyemo, W. L.; Jezewski, P. A.; Onwuamah, C. K.; Ogunlewe, M. O.; Ugboko, V. I.; Adejuyigbe, O.; Adigun, A. I.; Abdur-Rahman, L. O.; Onah, II; Audu, R. A.; Idigbe, E. O.; Mansilla, M. A.; Dragan, E. A.; Petrin, A. L.; Bullard, S. A.; Uduezue, A. O.; Akpata, O.; Osaguona, A. O.; Olasoji, H. O.; Ligali, T. O.; Kejeh, B. M.; Iseh, K. R.; Olaitan, P. B.; Adebola, A. R.; Efunkoya, E.; Adesina, O. A.; Oluwatosin, O. M.; Murray, J. C. | 2011 | | Genetic studies in the Nigerian population implicate an MSX1 mutation in complex oral facial clefting disorders | | unrelated topic |
| 1. Akporiaye, L. E.; Oguike, T. C.; Evbuomwan, I. | 2009 | | Genito-urinary reconstruction in southern Nigeria | | unrelated topic |
| 1. Manojlovic, Z.; Auslander, A.; Jin, Y.; Schmidt, R. J.; Xu, Y.; Chang, S.; Song, R.; Ingles, S. A.; Nunes, A.; Vavra, K. C.; Feigelson, D.; Rakotoarison, S.; DiBona, M.; Magee, K.; Smile, O.; Ramamonjisoa, A.; Magee Iii, W. | 2023 | | Genome Analysis Using Whole-Exome Sequencing of Non-Syndromic Cleft Lip and/or Palate from Malagasy Trios Identifies Variants Associated with Cilium-Related Pathways and Asian Genetic Ancestry | | unrelated topic |
| 1. Mukhopadhyay, N.; Feingold, E.; Moreno-Uribe, L.; Wehby, G.; Valencia-Ramirez, L. C.; Restrepo Muñeton, C. P.; Padilla, C.; Deleyiannis, F.; Christensen, K.; Poletta, F. A.; Orioli, I. M.; Hecht, J. T.; Buxó, C. J.; Butali, A.; Adeyemo, W. L.; Vieira, A. R.; Shaffer, J. R.; Murray, J. C.; Weinberg, S. M.; Leslie, E. J.; Marazita, M. L. | 2022 | | Genome-wide association study of multiethnic nonsyndromic orofacial cleft families identifies novel loci specific to family and phenotypic subtypes | | unrelated topic |
| 1. Yilmaz, F.; Null, M.; Astling, D.; Yu, H. C.; Cole, J.; Santorico, S. A.; Hallgrimsson, B.; Manyama, M.; Spritz, R. A.; Hendricks, A. E.; Shaikh, T. H. | 2021 | | Genome-wide copy number variations in a large cohort of bantu African children | | unrelated topic |
| 1. Awotoye, W.; Comnick, C.; Pendleton, C.; Zeng, E.; Alade, A.; Mossey, P. A.; Gowans, L. J. J.; Eshete, M. A.; Adeyemo, W. L.; Naicker, T.; Adeleke, C.; Busch, T.; Li, M.; Petrin, A.; Olotu, J.; Hassan, M.; Pape, J.; Miller, S. E.; Donkor, P.; Anand, D.; Lachke, S. A.; Marazita, M. L.; Adeyemo, A. A.; Murray, J. C.; Albokhari, D.; Sobreira, N.; Butali, A. | 2022 | | Genome-wide Gene-by-Sex Interaction Studies Identify Novel Nonsyndromic Orofacial Clefts Risk Locus | | unrelated topic |
| 1. Butali, A.; Mossey, P. A.; Adeyemo, W. L.; Eshete, M. A.; Gowans, L. J. J.; Busch, T. D.; Jain, D.; Yu, W.; Huan, L.; Laurie, C. A.; Laurie, C. C.; Nelson, S.; Li, M.; Sanchez-Lara, P. A.; Magee, W. P.; Magee, K. S.; Auslander, A.; Brindopke, F.; Kay, D. M.; Caggana, M.; Romitti, P. A.; Mills, J. L.; Audu, R.; Onwuamah, C.; Oseni, G. O.; Owais, A.; James, O.; Olaitan, P. B.; Aregbesola, B. S.; Braimah, R. O.; Oginni, F. O.; Oladele, A. O.; Bello, S. A.; Rhodes, J.; Shiang, R.; Donkor, P.; Obiri-Yeboah, S.; Arthur, F. K. N.; Twumasi, P.; Agbenorku, P.; Plange-Rhule, G.; Oti, A. A.; Ogunlewe, O. M.; Oladega, A. A.; Adekunle, A. A.; Erinoso, A. O.; Adamson, O. O.; Elufowoju, A. A.; Ayelomi, O. I.; Hailu, T.; Hailu, A.; Demissie, Y.; Derebew, M.; Eliason, S.; Romero-Bustillous, M.; Lo, C.; Park, J.; Desai, S.; Mohammed, M.; Abate, F.; Abdur-Rahman, L. O.; Anand, D.; Saadi, I.; Oladugba, A. V.; Lachke, S. A.; Amendt, B. A.; Rotimi, C. N.; Marazita, M. L.; Cornell, R. A.; Murray, J. C.; Adeyemo, A. A. | 2019 | | Genomic analyses in African populations identify novel risk loci for cleft palate | | unrelated topic |
| 1. Faye, O.; de Lourdes Monteiro, M.; Vrancken, B.; Prot, M.; Lequime, S.; Diarra, M.; Ndiaye, O.; Valdez, T.; Tavarez, S.; Ramos, J.; da Veiga Leal, S.; Pires, C.; Moreira, A.; Tavares, M. F.; Fernandes, L.; Barreto, J. N.; do Céu Teixeira, M.; de Lima Mendonça, M. D. L.; Gomes, Ccdsl; Castellon, M. S.; Ma, L.; Lemoine, F.; Gámbaro-Roglia, F.; Delaune, D.; Fall, G.; Fall, I. S.; Diop, M.; Sakuntabhai, A.; Loucoubar, C.; Lemey, P.; Holmes, E. C.; Sall, A. A.; Simon-Loriere, E. | 2020 | | Genomic Epidemiology of 2015-2016 Zika Virus Outbreak in Cape Verde | | unrelated topic |
| 1. Legese, M. H.; Asrat, D.; Mihret, A.; Hasan, B.; Mekasha, A.; Aseffa, A.; Swedberg, G. | 2022 | | Genomic Epidemiology of Carbapenemase-Producing and Colistin-Resistant Enterobacteriaceae among Sepsis Patients in Ethiopia: a Whole-Genome Analysis | | unrelated topic |
| 1. Albesher, N | 2023 | | Genomic Screening of Non-syndromic Congenital Heart Defects in the Saudi Population | | book |
| 1. Thomford, NE; Dzobo, K; Yao, NA; ... | 2018 | | Genomics and epigenomics of congenital heart defects: expert review and lessons learned in Africa | | Duplicate |
| 1. Thomford, N. E.; Dzobo, K.; Yao, N. A.; Chimusa, E.; Evans, J.; Okai, E.; Kruszka, P.; Muenke, M.; Awandare, G.; Wonkam, A.; Dandara, C. | 2018 | | Genomics and Epigenomics of Congenital Heart Defects: Expert Review and Lessons Learned in Africa | | unrelated topic |
| 1. Thomford, NE; Dzobo, K; Yao, NA; ... | 2018 | | Genomics and epigenomics of congenital heart defects: expert review and lessons learned in Africa | | unrelated topic |
| 1. Bartstra, J. W.; Risseeuw, S.; de Jong, P. A.; van Os, B.; Kalsbeek, L.; Mol, C.; Baas, A. F.; Verschuere, S.; Vanakker, O.; Florijn, R. J.; Hendrikse, J.; Mali, W.; Imhof, S.; Ossewaarde-van Norel, J.; van Leeuwen, R.; Spiering, W. | 2021 | | Genotype-phenotype correlation in pseudoxanthoma elasticum | | unrelated topic |
| 1. Pukuta, E.; Waku-Kouomou, D.; Abernathy, E.; Illunga, B. K.; Obama, R.; Mondonge, V.; Dahl, B. A.; Maresha, B. G.; Icenogle, J.; Muyembe, J. J. | 2016 | | Genotypes of rubella virus and the epidemiology of rubella infections in the Democratic Republic of the Congo, 2004-2013 | | unrelated topic |
| 1. Spray, P. | 1967 | | Genu valgum in Nigeria | | Year of publication |
| 1. Fantong, W. Y.; Satake, H.; Ayonghe, S. N.; Suh, E. C.; Adelana, S. M.; Fantong, E. B.; Banseka, H. S.; Gwanfogbe, C. D.; Woincham, L. N.; Uehara, Y.; Zhang, J. | 2010 | | Geochemical provenance and spatial distribution of fluoride in groundwater of Mayo Tsanaga River Basin, Far North Region, Cameroon: implications for incidence of fluorosis and optimal consumption dose | | unrelated topic |
| 1. Rabbitts, J. A.; Groenewald, C. B.; Räsänen, J. | 2012 | | Geographic differences in perioperative opioid administration in children | | unrelated topic |
| 1. Suwanwela, C. | 1972 | | Geographical distribution of fronto-ethmoidal encephalomeningocele | | Year of publication |
| 1. Larsson, L.; Johansson, B.; Sandberg, C.; Apers, S.; Kovacs, A. H.; Luyckx, K.; Thomet, C.; Budts, W.; Enomoto, J.; Sluman, M. A.; Wang, J. K.; Jackson, J. L.; Khairy, P.; Cook, S. C.; Alday, L.; Eriksen, K.; Dellborg, M.; Berghammer, M.; Rempel, G.; Menahem, S.; Caruana, M.; Tomlin, M.; Soufi, A.; Fernandes, S. M.; White, K.; Callus, E.; Kutty, S.; Moons, P. | 2019 | | Geographical variation and predictors of physical activity level in adults with congenital heart disease | | unrelated topic |
| 1. Msamati, B. C.; Igbigbi, P. S.; Lavy, C. B. | 2003 | | Geometric measurements of the acetabulum in adult Malawians: radiographic study | | unrelated topic |
| 1. Serris, A.; Zoungrana, J.; Diallo, M.; Toby, R.; Mpoudi Ngolle, M.; Le Gac, S.; Coutherut, J.; Cournil, A.; De Beaudrap, P.; Koulla-Shiro, S.; Delaporte, E.; Ciaffi, L. | 2016 | | Getting pregnant in HIV clinical trials: women's choice and safety needs. The experience from the ANRS12169-2LADY and ANRS12286-MOBIDIP trials | | unrelated topic |
| 1. Poenaru, D. | 2013 | | Getting the job done: analysis of the impact and effectiveness of the SmileTrain program in alleviating the global burden of cleft disease | | unrelated topic |
| 1. Bot, G. M.; Ismail, N. J.; Mahmud, M. R.; Hassan, I.; Lasseini, A.; Shilong, D. J.; Obande, J. O.; Usman, B.; Houlihan, L. M.; Preul, M. C.; Shehu, B. B. | 2020 | | Giant Encephalocele in Sokoto, Nigeria: A 5-Year Review of Operated Cases | | Duplicate |
| 1. Bot, G. M.; Ismail, N. J.; Mahmud, M. R.; Hassan, I.; Lasseini, A.; Shilong, D. J.; Obande, J. O.; Usman, B.; Houlihan, L. M.; Preul, M. C.; Shehu, B. B. | 2020 | | Giant Encephalocele in Sokoto, Nigeria: A 5-Year Review of Operated Cases | | unrelated topic |
| 1. Hailu, S. S.; Derbew, H. M.; Zeray, A.; Hailemariam, T.; Otero, H. J. | 2023 | | Giant pulmonary artery aneurysm in a child: Rare complication of congenital heart disease | | bookSection |
| 1. Manji, F. | 1984 | | Gingivitis, dental fluorosis, and dental caries in primary school children of Nairobi, Kenya | | Year of publication |
| 1. Yalcouyé, A.; Diallo, S. H.; Cissé, L.; Karembé, M.; Diallo, S.; Coulibaly, T.; Diarra, S.; Coulibaly, D.; Keita, M.; Guinto, C. O.; Fischbeck, K. H.; Wonkam, A.; Landouré, G. | 2022 | | GJB1 variants in Charcot-Marie-Tooth disease X-linked type 1 in Mali | | unrelated topic |
| 1. van der Merwe, E. B.; Bhika, R. E.; Meyer, D. | 2019 | | Glaucoma in Phacomatosis Pigmentovascularis in a Young African Adolescent Boy: A Case Report | | Duplicate |
| 1. van der Merwe, E. B.; Bhika, R. E.; Meyer, D. | 2019 | | Glaucoma in Phacomatosis Pigmentovascularis in a Young African Adolescent Boy: A Case Report | | unrelated topic |
| 1. Lozano, R.; Naghavi, M.; Foreman, K.; Lim, S.; Shibuya, K.; Aboyans, V.; Abraham, J.; Adair, T.; Aggarwal, R.; Ahn, S. Y.; Alvarado, M.; Anderson, H. R.; Anderson, L. M.; Andrews, K. G.; Atkinson, C.; Baddour, L. M.; Barker-Collo, S.; Bartels, D. H.; Bell, M. L.; Benjamin, E. J.; Bennett, D.; Bhalla, K.; Bikbov, B.; Bin Abdulhak, A.; Birbeck, G.; Blyth, F.; Bolliger, I.; Boufous, S.; Bucello, C.; Burch, M.; Burney, P.; Carapetis, J.; Chen, H.; Chou, D.; Chugh, S. S.; Coffeng, L. E.; Colan, S. D.; Colquhoun, S.; Colson, K. E.; Condon, J.; Connor, M. D.; Cooper, L. T.; Corriere, M.; Cortinovis, M.; de Vaccaro, K. C.; Couser, W.; Cowie, B. C.; Criqui, M. H.; Cross, M.; Dabhadkar, K. C.; Dahodwala, N.; De Leo, D.; Degenhardt, L.; Delossantos, A.; Denenberg, J.; Des Jarlais, D. C.; Dharmaratne, S. D.; Dorsey, E. R.; Driscoll, T.; Duber, H.; Ebel, B.; Erwin, P. J.; Espindola, P.; Ezzati, M.; Feigin, V.; Flaxman, A. D.; Forouzanfar, M. H.; Fowkes, F. G.; Franklin, R.; Fransen, M.; Freeman, M. K.; Gabriel, S. E.; Gakidou, E.; Gaspari, F.; Gillum, R. F.; Gonzalez-Medina, D.; Halasa, Y. A.; Haring, D.; Harrison, J. E.; Havmoeller, R.; Hay, R. J.; Hoen, B.; Hotez, P. J.; Hoy, D.; Jacobsen, K. H.; James, S. L.; Jasrasaria, R.; Jayaraman, S.; Johns, N.; Karthikeyan, G.; Kassebaum, N.; Keren, A.; Khoo, J. P.; Knowlton, L. M.; Kobusingye, O.; Koranteng, A.; Krishnamurthi, R.; Lipnick, M.; Lipshultz, S. E.; Ohno, S. L.; others | 2012 | | Global and regional mortality from 235 causes of death for 20 age groups in 1990 and 2010: a systematic analysis for the Global Burden of Disease Study 2010 | | unrelated topic |
| 1. Liu, Y.; Chen, S.; Zühlke, L.; Black, G. C.; Choy, M. K.; Li, N.; Keavney, B. D. | 2019 | | Global birth prevalence of congenital heart defects 1970-2017: updated systematic review and meta-analysis of 260 studies | | unrelated topic |
| 1. Panamonta, V.; Pradubwong, S.; Panamonta, M.; Chowchuen, B. | 2015 | | Global Birth Prevalence of Orofacial Clefts: A Systematic Review | | unrelated topic |
| 1. Coffey, S.; Roberts-Thomson, R.; Brown, A.; Carapetis, J.; Chen, M.; Enriquez-Sarano, M.; Zühlke, L.; Prendergast, B. D. | 2021 | | Global epidemiology of valvular heart disease | | unrelated topic |
| 1. Dewan, M. C.; Rattani, A.; Mekary, R.; Glancz, L. J.; Yunusa, I.; Baticulon, R. E.; Fieggen, G.; Wellons, J. C.; Park, K. B.; Warf, B. C. | 2019 | | Global hydrocephalus epidemiology and incidence: systematic review and meta-analysis | | unrelated topic |
| 1. Mahomed, S.; Dhai, A. | 2019 | | Global injustice in sport: The Caster Semenya ordeal - prejudice, discrimination and racial bias | | unrelated topic |
| 1. H Dokainish, K Teo, J Zhu, A Roy… | 2017 | | Global mortality variations in patients with heart failure: results from the International Congestive Heart Failure (INTER-CHF) prospective cohort study | | unrelated topic |
| 1. Murray, C. J.; Lopez, A. D. | 1997 | | Global mortality, disability, and the contribution of risk factors: Global Burden of Disease Study | | Year of publication |
| 1. McClure, E. M.; Bose, C. L.; Garces, A.; Esamai, F.; Goudar, S. S.; Patel, A.; Chomba, E.; Pasha, O.; Tshefu, A.; Kodkany, B. S.; Saleem, S.; Carlo, W. A.; Derman, R. J.; Hibberd, P. L.; Liechty, E. A.; Hambidge, K. M.; Krebs, N. F.; Bauserman, M.; Koso-Thomas, M.; Moore, J.; Wallace, D. D.; Jobe, A. H.; Goldenberg, R. L. | 2015 | | Global network for women's and children's health research: a system for low-resource areas to determine probable causes of stillbirth, neonatal, and maternal death | | unrelated topic |
| 1. McClure, E. M.; Garces, A.; Saleem, S.; Moore, J. L.; Bose, C. L.; Esamai, F.; Goudar, S. S.; Chomba, E.; Mwenechanya, M.; Pasha, O.; Tshefu, A.; Patel, A.; Dhaded, S. M.; Tenge, C.; Marete, I.; Bauserman, M.; Sunder, S.; Kodkany, B. S.; Carlo, W. A.; Derman, R. J.; Hibberd, P. L.; Liechty, E. A.; Hambidge, K. M.; Krebs, N. F.; Koso-Thomas, M.; Miodovnik, M.; Wallace, D. D.; Goldenberg, R. L. | 2018 | | Global Network for Women's and Children's Health Research: probable causes of stillbirth in low- and middle-income countries using a prospectively defined classification system | | unrelated topic |
| 1. Kantar, R. S.; Hamdan, U. S.; Muller, J. N.; Hemal, K.; Younan, R. A.; Haddad, M.; Melhem, A. M.; Don Griot, J. P. W.; Breugem, C. C.; Mokdad, A. H. | 2023 | | Global Prevalence and Burden of Orofacial Clefts: A Systematic Analysis for the Global Burden of Disease Study 2019 | | unrelated topic |
| 1. Dave, M.; Rankin, J.; Pearce, M.; Foster, H. E. | 2020 | | Global prevalence estimates of three chronic musculoskeletal conditions: club foot, juvenile idiopathic arthritis and juvenile systemic lupus erythematosus | | unrelated topic |
| 1. Liu, Y.; Chen, S.; Zühlke, L.; Babu-Narayan, S. V.; Black, G. C.; Choy, M. K.; Li, N.; Keavney, B. D. | 2020 | | Global prevalence of congenital heart disease in school-age children: a meta-analysis and systematic review | | Review |
| 1. Dave, M.; Taylor, G. | 2018 | | Global prevalence of molar incisor hypomineralisation | | unrelated topic |
| 1. Gitau, E. N.; Kokwaro, G. O.; Newton, C. R.; Ward, S. A. | 2011 | | Global proteomic analysis of plasma from mice infected with Plasmodium berghei ANKA using two dimensional gel electrophoresis and matrix assisted laser desorption ionization-time of flight mass spectrometry | | unrelated topic |
| 1. Mossey, PA; Catilla, EE | 2003 | | Global registry and database on craniofacial anomalies: Report of a WHO Registry Meeting on Craniofacial Anomalies | | book |
| 1. Zilla, P.; Yacoub, M.; Zühlke, L.; Beyersdorf, F.; Sliwa, K.; Khubulava, G.; Bouzid, A.; Mocumbi, A. O.; Velayoudam, D.; Shetty, D.; Ofoegbu, C.; Geldenhuys, A.; Brink, J.; Scherman, J.; du Toit, H.; Hosseini, S.; Zhang, H.; Luo, X. J.; Wang, W.; Mejia, J.; Kofidis, T.; Higgins, R. S. D.; Pomar, J.; Bolman, R. M.; Mayosi, B. M.; Madansein, R.; Bavaria, J.; Yanes-Quintana, A. A.; Kumar, A. S.; Adeoye, O.; Chauke, R. F.; Williams, D. F. | 2018 | | Global Unmet Needs in Cardiac Surgery | | unrelated topic |
| 1. Cubitt, J. J.; Hodges, A. M.; Van Lierde, K. M.; Swan, M. C. | 2014 | | Global variation in cleft palate repairs: an analysis of 352,191 primary cleft repairs in low- to higher-middle-income countries | | unrelated topic |
| 1. Wang, D.; Zhang, B.; Zhang, Q.; Wu, Y. | 2023 | | Global, regional and national burden of orofacial clefts from 1990 to 2019: an analysis of the Global Burden of Disease Study 2019 | | unrelated topic |
| 1. Liu, L.; Oza, S.; Hogan, D.; Chu, Y.; Perin, J.; Zhu, J.; Lawn, J. E.; Cousens, S.; Mathers, C.; Black, R. E. | 2016 | | Global, regional, and national causes of under-5 mortality in 2000-15: an updated systematic analysis with implications for the Sustainable Development Goals | | unrelated topic |
| 1. Lu, V. M. | 2023 | | Global, regional, and national epidemiological trends in neural tube defects between 1990 and 2019: a summary | | unrelated topic |
| 1. Kang, L.; Cao, G.; Jing, W.; Liu, J.; Liu, M. | 2023 | | Global, regional, and national incidence and mortality of congenital birth defects from 1990 to 2019 | | unrelated topic |
| 1. Su, Z.; Zou, Z.; Hay, S. I.; Liu, Y.; Li, S.; Chen, H.; Naghavi, M.; Zimmerman, M. S.; Martin, G. R.; Wilner, L. B.; Sable, C. A.; Murray, C. J. L.; Kassebaum, N. J.; Patton, G. C.; Zhang, H. | 2022 | | Global, regional, and national time trends in mortality for congenital heart disease, 1990-2019: An age-period-cohort analysis for the Global Burden of Disease 2019 study | | unrelated topic |
|  | 2016 | | Global, regional, national, and selected subnational levels of stillbirths, neonatal, infant, and under-5 mortality, 1980-2015: a systematic analysis for the Global Burden of Disease Study 2015 | | unrelated topic |
| 1. Obel, A. O. | 1982 | | Goitre and fluorosis in Kenya | | Year of publication |
| 1. Lucas-Herald, A. K.; Bryce, J.; Kyriakou, A.; Ljubicic, M. L.; Arlt, W.; Audi, L.; Balsamo, A.; Baronio, F.; Bertelloni, S.; Bettendorf, M.; Brooke, A.; Claahsen van der Grinten, H. L.; Davies, J. H.; Hermann, G.; de Vries, L.; Hughes, I. A.; Tadokoro-Cuccaro, R.; Darendeliler, F.; Poyrazoglu, S.; Ellaithi, M.; Evliyaoglu, O.; Fica, S.; Nedelea, L.; Gawlik, A.; Globa, E.; Zelinska, N.; Guran, T.; Güven, A.; Hannema, S. E.; Hiort, O.; Holterhus, P. M.; Iotova, V.; Mladenov, V.; Jain, V.; Sharma, R.; Jennane, F.; Johnston, C.; Guerra Junior, G.; Konrad, D.; Gaisl, O.; Krone, N.; Krone, R.; Lachlan, K.; Li, D.; Lichiardopol, C.; Lisa, L.; Markosyan, R.; Mazen, I.; Mohnike, K.; Niedziela, M.; Nordenstrom, A.; Rey, R.; Skaeil, M.; Tack, L. J. W.; Tomlinson, J.; Weintrob, N.; Cools, M.; Ahmed, S. F. | 2021 | | Gonadectomy in conditions affecting sex development: a registry-based cohort study | | unrelated topic |
| 1. Terrier, B.; Dechartres, A.; Deligny, C.; Godmer, P.; Charles, P.; Hayem, G.; Dunogué, B.; de Bandt, M.; Cohen, P.; Puéchal, X.; Jeunne, C. L.; Arfi, S.; Mouthon, L.; Guillevin, L. | 2017 | | Granulomatosis with polyangiitis according to geographic origin and ethnicity: clinical-biological presentation and outcome in a French population | | unrelated topic |
| 1. Thiagarajan, R. I.; Scheurer, M. A.; Salvin, J. W. | 2014 | | Great need, scarce resources, and choice: reflections on ethical issues following a medical mission | | unrelated topic |
| 1. Ngnintedem Yonti, C.; Kenfack Tsobnang, P.; Lontio Fomekong, R.; Devred, F.; Mignolet, E.; Larondelle, Y.; Hermans, S.; Delcorte, A.; Lambi Ngolui, J. | 2021 | | Green Synthesis of Iron-Doped Cobalt Oxide Nanoparticles from Palm Kernel Oil via Co-Precipitation and Structural Characterization | | unrelated topic |
| 1. Lubala, T. K.; Lubala, N.; Munkana, A. N.; Nyenga, A. M.; Mutombo, A. M. | 2013 | | Greenberg Skeletal Dysplasia: first reported case in the Democratic Republic of Congo | | unrelated topic |
| 1. Abantanga, F. A. | 2003 | | Groin and scrotal swellings in children aged 5 years and below: a review of 535 cases | | unrelated topic |
| 1. Gbadebo, A. M. | 2012 | | Groundwater fluoride and dental fluorosis in southwestern Nigeria | | unrelated topic |
| 1. Rango, T.; Kravchenko, J.; Atlaw, B.; McCornick, P. G.; Jeuland, M.; Merola, B.; Vengosh, A. | 2012 | | Groundwater quality and its health impact: An assessment of dental fluorosis in rural inhabitants of the Main Ethiopian Rift | | unrelated topic |
| 1. Kapito-Tembo, A. P.; Bauleni, A.; Wesevich, A.; Ongubo, D.; Hosseinipour, M. C.; Dube, Q.; Mwale, P.; Corbett, A.; Mwapasa, V.; Phiri, S. | 2021 | | Growth and Neurodevelopment Outcomes in HIV-, Tenofovir-, and Efavirenz-Exposed Breastfed Infants in the PMTCT Option B+ Program in Malawi | | unrelated topic |
| 1. Mabhandi, T.; Ramdin, T.; Ballot, D. E. | 2019 | | Growth of extremely low birth weight infants at a tertiary hospital in a middle-income country | | unrelated topic |
| 1. West, K. L.; Fernandez, M. L. | 2004 | | Guinea pigs as models to study the hypocholesterolemic effects of drugs | | unrelated topic |
| 1. Touré, A. M.; Landry, M.; Souchkova, O.; Kembel, S. W.; Pilon, N. | 2019 | | Gut microbiota-mediated Gene-Environment interaction in the TashT mouse model of Hirschsprung disease | | unrelated topic |
| 1. Langesæter, E | 2009 | | Haemodynamic changes during spinal anaesthesia for caesarean section | | book |
| 1. Ekure, E. N.; Sokunbi, O.; Kruszka, P.; Muenke, M.; Adeyemo, A. A. | 2023 | | Hajdu-Cheney syndrome with atypical cardiovascular abnormalities | | unrelated topic |
| 1. Adebayo, Bosede E and Ogunkunle, Oluwatoyin O and Omokhodion, Samuel I and Luke, Ronita D | 2016 | | he spectrum of structural heart defects seen in children at the University College Hospital, Ibadan | | Included |
| 1. Evans, C.; Chasekwa, B.; Ntozini, R.; Humphrey, J. H.; Prendergast, A. J. | 2016 | | Head circumferences of children born to HIV-infected and HIV-uninfected mothers in Zimbabwe during the preantiretroviral therapy era | | unrelated topic |
| 1. Steyn, K.; Steyn, M.; Langenhoven, M. L.; Rossouw, J. E.; Fourie, J. | 1990 | | Health actions and disease patterns related to coronary heart disease in the coloured population of the Cape Peninsula | | Duplicate |
| 1. Steyn, K.; Steyn, M.; Langenhoven, M. L.; Rossouw, J. E.; Fourie, J. | 1990 | | Health actions and disease patterns related to coronary heart disease in the coloured population of the Cape Peninsula | | Year of publication |
| 1. Holbein, C. E.; Peugh, J.; Veldtman, G. R.; Apers, S.; Luyckx, K.; Kovacs, A. H.; Thomet, C.; Budts, W.; Enomoto, J.; Sluman, M. A.; Lu, C. W.; Jackson, J. L.; Khairy, P.; Cook, S. C.; Chidambarathanu, S.; Alday, L.; Eriksen, K.; Dellborg, M.; Berghammer, M.; Johansson, B.; Mackie, A. S.; Menahem, S.; Caruana, M.; Soufi, A.; Fernandes, S. M.; White, K.; Callus, E.; Kutty, S.; Moons, P. | 2020 | | Health behaviours reported by adults with congenital heart disease across 15 countries | | unrelated topic |
| 1. Redfield, R. R.; Modi, S.; Moore, C. A.; Delaney, A.; Honein, M. A.; Tomlinson, H. L. | 2019 | | Health Care Autonomy of Women Living with HIV | | unrelated topic |
| 1. Letamo, G.; Majelantle, R. G. | 2001 | | Health implications of early childbearing on pregnancy outcome in Botswana: insights from the institutional records | | unrelated topic |
| 1. Adekunle, A. A.; James, O.; Adeyemo, W. L. | 2020 | | Health Information Seeking Through Social Media and Search Engines by Parents of Children With Orofacial Cleft in Nigeria | | unrelated topic |
| 1. Buzon, M. R. | 2006 | | Health of the non-elites at Tombos: Nutritional and disease stress in New Kingdom Nubia | | unrelated topic |
| 1. Richter, L.; Slemming, W.; Norris, S. A.; Stein, A.; Poston, L.; Pasupathy, D. | 2020 | | Health Pregnancy, Healthy Baby: testing the added benefits of pregnancy ultrasound scan for child development in a randomised control trial | | unrelated topic |
| 1. Van Bulck, L.; Goossens, E.; Luyckx, K.; Apers, S.; Oechslin, E.; Thomet, C.; Budts, W.; Enomoto, J.; Sluman, M. A.; Lu, C. W.; Jackson, J. L.; Khairy, P.; Cook, S. C.; Chidambarathanu, S.; Alday, L.; Eriksen, K.; Dellborg, M.; Berghammer, M.; Johansson, B.; Mackie, A. S.; Menahem, S.; Caruana, M.; Veldtman, G.; Soufi, A.; Fernandes, S. M.; White, K.; Callus, E.; Kutty, S.; Moons, P. | 2020 | | Healthcare system inputs and patient-reported outcomes: a study in adults with congenital heart defect from 15 countries | | unrelated topic |
| 1. Miranda, R. N.; Ximenes, R.; Gebretekle, G. B.; Bielecki, J. M.; Sander, B. | 2020 | | Health-Related Quality of Life in Neurological Disorders Most Commonly Associated With Zika-Virus Infection: A Systematic Review | | unrelated topic |
| 1. Bruneel, L.; Alighieri, C.; De Smet, S.; Bettens, K.; De Bodt, M.; Van Lierde, K. | 2019 | | Health-related quality of life in patients with cleft palate: Reproducibility, responsiveness and construct validity of the Dutch version of the VELO questionnaire | | unrelated topic |
| 1. Bruneel, L.; Van Lierde, K.; Bettens, K.; Corthals, P.; Van Poel, E.; De Groote, E.; Keppler, H. | 2017 | | Health-related quality of life in patients with cleft palate: Validity and reliability of the VPI Effects on Life Outcomes (VELO) questionnaire translated to Dutch | | unrelated topic |
| 1. Gebrehiwot, A. G.; Melka, D. S.; Kassaye, Y. M.; Rehan, I. F.; Rangappa, S.; Hinou, H.; Kamiyama, T.; Nishimura, S. I. | 2018 | | Healthy human serum N-glycan profiling reveals the influence of ethnic variation on the identified cancer-relevant glycan biomarkers | | unrelated topic |
| 1. Douglas, B. L. | 1977 | | Healthy with high fluoride | | Year of publication |
| 1. Lubega, S.; Zirembuzi, G. W.; Lwabi, P. | 2005 | | Heart disease among children with HIV/AIDS attending the paediatric infectious disease clinic at Mulago Hospital | | unrelated topic |
| 1. Raphael, D. M.; Roos, L.; Myovela, V.; McHomvu, E.; Namamba, J.; Kilindimo, S.; Gingo, W.; Hatz, C.; Paris, D. H.; Weisser, M.; Kobza, R.; Rohacek, M. | 2018 | | Heart diseases and echocardiography in rural Tanzania: Occurrence, characteristics, and etiologies of underappreciated cardiac pathologies | | study setting |
| 1. Lu, C. W.; Wang, J. K.; Yang, H. L.; Kovacs, A. H.; Luyckx, K.; Ruperti-Repilado, F. J.; Van De Bruaene, A.; Enomoto, J.; Sluman, M. A.; Jackson, J. L.; Khairy, P.; Cook, S. C.; Chidambarathanu, S.; Alday, L.; Oechslin, E.; Eriksen, K.; Dellborg, M.; Berghammer, M.; Johansson, B.; Mackie, A. S.; Menahem, S.; Caruana, M.; Veldtman, G.; Soufi, A.; Fernandes, S. M.; White, K.; Callus, E.; Kutty, S.; Apers, S.; Moons, P. | 2022 | | Heart Failure and Patient-Reported Outcomes in Adults With Congenital Heart Disease from 15 Countries | | unrelated topic |
| 1. H Dokainish, K Teo, J Zhu, A Roy, KF AlHabib… | 2016 | | Heart failure in Africa, Asia, the Middle East and South America: the INTER-CHF study | | unrelated topic |
| 1. Nigussie, B.; Tadele, H. | 2019 | | Heart Failure in Ethiopian Children: Mirroring the Unmet Cardiac Services | | unrelated topic |
| 1. H Dokainish, K Teo, J Zhu, A Roy, K Al-Habib… | 2015 | | Heart failure in low-and middle-income countries: Background, rationale, and design of the INTERnational Congestive Heart Failure Study (INTER-CHF) | | unrelated topic |
| 1. VN Agbor, M Essouma, NAB Ntusi, UF Nyaga… | 2018 | | Heart failure in sub-Saharan Africa: a contemporaneous systematic review and meta-analysis | | unrelated topic |
| 1. Arnaert, S.; De Meester, P.; Troost, E.; Droogne, W.; Van Aelst, L.; Van Cleemput, J.; Voros, G.; Gewillig, M.; Cools, B.; Moons, P.; Rega, F.; Meyns, B.; Zhang, Z.; Budts, W.; Van De Bruaene, A. | 2021 | | Heart failure related to adult congenital heart disease: prevalence, outcome and risk factors | | unrelated topic |
| 1. Bakari, S.; Koca, B.; Oztunç, F.; Abuhandan, M. | 2013 | | Heart rate variability in patients with atrial septal defect and healthy children | | unrelated topic |
| 1. Ofodile, F. A.; Oluwasanmi, J. O. | 1980 | | Hemangiomas in Nigeria | | Year of publication |
| 1. Alemseged, S.; Tefera, E. | 2023 | | Hematologic Derangements among Children with Unoperated Cyanotic Congenital Heart Disease in Ethiopia | | unrelated topic |
| 1. Schoub, B. D.; Johnson, S.; McAnerney, J. M.; Blackburn, N. K. | 1993 | | Hepatitis B virus prevalence in two institutions for the mentally handicapped | | Year of publication |
|  | 1971 | | Herbicide commission reports extensive damage | | Year of publication |
| 1. Ladipo, G. O. | 1978 | | Hereditary haemorrhagic telangiectasia (Sutton-Rendu-Osler-Weber disease): first case reports in Nigerians | | Year of publication |
| 1. Kitonyi, G. W.; Wambugu, P. M.; Oburra, H. O.; Ireri, J. M. | 2008 | | Hereditary haemorrhagic telangiectasia in a black adult male: case report | | unrelated topic |
| 1. Jaiyesimi, A. E.; Mba, E. C. | 1989 | | Hereditary haemorrhagic telangiectasia in Nigeria: case report | | Year of publication |
| 1. Canzonieri, C.; Ornati, F.; Matti, E.; Chu, F.; Manfredi, G.; Olivieri, C.; Buscarini, E.; Pagella, F. | 2014 | | Hereditary haemorrhagic telangiectasia in North African and sub-Saharan patients | | unrelated topic |
| 1. Aiyesimoju, A. B.; Osuntokun, B. O.; Bademosi, O.; Adeuja, A. O. | 1984 | | Hereditary neurodegenerative disorders in Nigerian Africans | | Year of publication |
| 1. Weatherall, D. J.; Clegg, J. B. | 1975 | | Hereditary persistence of fetal haemoglobin | | Year of publication |
| 1. Diarra, S.; Coulibaly, T.; Dembélé, K.; Ngouth, N.; Cissé, L.; Diallo, S. H.; Ouologuem, M.; Diallo, S.; Coulibaly, O.; Bagayoko, K.; Coulibaly, D.; Simaga, A.; Sango, H. A.; Traoré, M.; Jacobson, S.; Fischbeck, K. H.; Landouré, G.; Guinto, C. O. | 2023 | | Hereditary spastic paraplegia in Mali: epidemiological and clinical features | | unrelated topic |
| 1. Landouré, G.; Dembélé, K.; Cissé, L.; Samassékou, O.; Diarra, S.; Bocoum, A.; Dembélé, M. E.; Fischbeck, K. H.; Guinto, C. O. | 2019 | | Hereditary spastic paraplegia type 35 in a family from Mali | | unrelated topic |
| 1. Puckerin, A.; Aromolaran, K. A.; Chang, D. D.; Zukin, R. S.; Colecraft, H. M.; Boutjdir, M.; Aromolaran, A. S. | 2016 | | hERG 1a LQT2 C-terminus truncation mutants display hERG 1b-dependent dominant negative mechanisms | | unrelated topic |
| 1. Milani, G.; Budriesi, R.; Tavazzani, E.; Cavalluzzi, M. M.; Mattioli, L. B.; Miniero, D. V.; Delre, P.; Belviso, B. D.; Denegri, M.; Cuocci, C.; Rotondo, N. P.; De Palma, A.; Gualdani, R.; Caliandro, R.; Mangiatordi, G. F.; Kumawat, A.; Camilloni, C.; Priori, S.; Lentini, G. | 2023 | | hERG stereoselective modulation by mexiletine-derived ureas: Molecular docking study, synthesis, and biological evaluation | | unrelated topic |
| 1. Kay, G. W.; Grobbelaar, J. A.; Hattingh, J. | 1992 | | Heritable testicular hypoplasia in Nguni (Bos indicus) bulls: vascular characteristics and testosterone production | | Year of publication |
| 1. Yunis, J. J.; Yasmineh, W. G. | 1971 | | Heterochromatin, satellite DNA, and cell function. Structural DNA of eucaryotes may support and protect genes and aid in speciation | | Year of publication |
| 1. Hageman, M. J. | 1980 | | Heterogeneity of Waardenburg syndrome in Kenyan Africans | | Year of publication |
| 1. Hageman, M. J. | 1980 | | Heterogeneity of Waardenburg syndrome in Kenyan Africans | | Year of publication |
| 1. Lokanga, R. A.; Senejani, A. G.; Sweasy, J. B.; Usdin, K. | 2015 | | Heterozygosity for a hypomorphic Polβ mutation reduces the expansion frequency in a mouse model of the Fragile X-related disorders | | unrelated topic |
| 1. Beighton, P. | 1997 | | Heterozygous manifestations in the heritable disorders of the skeleton | | Year of publication |
| 1. Wonkam, A.; Noubiap, J. J.; Bosch, J.; Dandara, C.; Toure, G. B. | 2013 | | Heterozygous p.Asp50Asn mutation in the GJB2 gene in two Cameroonian patients with keratitis-ichthyosis-deafness (KID) syndrome | | unrelated topic |
| 1. Holtz, A. M.; VanCoillie, R.; Vansickle, E. A.; Carere, D. A.; Withrow, K.; Torti, E.; Juusola, J.; Millan, F.; Person, R.; Guillen Sacoto, M. J.; Si, Y.; Wentzensen, I. M.; Pugh, J.; Vasileiou, G.; Rieger, M.; Reis, A.; Argilli, E.; Sherr, E. H.; Aldinger, K. A.; Dobyns, W. B.; Brunet, T.; Hoefele, J.; Wagner, M.; Haber, B.; Kotzaeridou, U.; Keren, B.; Heron, D.; Mignot, C.; Heide, S.; Courtin, T.; Buratti, J.; Murugasen, S.; Donald, K. A.; O'Heir, E.; Moody, S.; Kim, K. H.; Burton, B. K.; Yoon, G.; Campo, M. D.; Masser-Frye, D.; Kozenko, M.; Parkinson, C.; Sell, S. L.; Gordon, P. L.; Prokop, J. W.; Karaa, A.; Bupp, C.; Raby, B. A. | 2022 | | Heterozygous variants in MYH10 associated with neurodevelopmental disorders and congenital anomalies with evidence for primary cilia-dependent defects in Hedgehog signaling | | unrelated topic |
| 1. Noakes, T. D. | 2021 | | Hiding unhealthy heart outcomes in a low-fat diet trial: the Women's Health Initiative Randomized Controlled Dietary Modification Trial finds that postmenopausal women with established coronary heart disease were at increased risk of an adverse outcome if they consumed a low-fat 'heart-healthy' diet | | unrelated topic |
| 1. Gebreyesus, H. A.; Abreha, G. F.; Besherae, S. D.; Abera, M. A.; Weldegerima, A. H.; Gidey, A. H.; Bezabih, A. M.; Lemma, T. B.; Nigatu, T. G. | 2022 | | High atherogenic risk concomitant with elevated HbA1c among persons with type 2 diabetes mellitus in North Ethiopia | | unrelated topic |
| 1. Berihu, B. A.; Welderufael, A. L.; Berhe, Y.; Magana, T.; Mulugeta, A.; Asfaw, S.; Gebreselassie, K. | 2018 | | High burden of neural tube defects in Tigray, Northern Ethiopia: Hospital-based study | | unrelated topic |
| 1. Webb, R.; Culliford-Semmens, N.; ChanMow, A.; Doughty, R.; Tilton, E.; Peat, B.; Stirling, J.; Gentles, T. L.; Wilson, N. J. | 2023 | | High burden of rheumatic heart disease confirmed by echocardiography among Pacific adults living in New Zealand | | unrelated topic |
| 1. Hammarsjö, A.; Pettersson, M.; Chitayat, D.; Handa, A.; Anderlid, B. M.; Bartocci, M.; Basel, D.; Batkovskyte, D.; Beleza-Meireles, A.; Conner, P.; Eisfeldt, J.; Girisha, K. M.; Chung, B. H.; Horemuzova, E.; Hyodo, H.; Korņejeva, L.; Lagerstedt-Robinson, K.; Lin, A. E.; Magnusson, M.; Moosa, S.; Nayak, S. S.; Nilsson, D.; Ohashi, H.; Ohashi-Fukuda, N.; Stranneheim, H.; Taylan, F.; Traberg, R.; Voss, U.; Wirta, V.; Nordgren, A.; Nishimura, G.; Lindstrand, A.; Grigelioniene, G. | 2021 | | High diagnostic yield in skeletal ciliopathies using massively parallel genome sequencing, structural variant screening and RNA analyses | | unrelated topic |
| 1. Bignoumba, M.; Onanga, R.; Kumulungui, B. S.; Kassa, R. F. K.; Ndzime, Y. M.; Moghoa, K. M.; Stubbe, D.; Becker, P. | 2023 | | High diversity of yeast species and strains responsible for vulvovaginal candidiasis in South-East Gabon | | unrelated topic |
| 1. Wambu, E. W.; Agong, S. G.; Anyango, B.; Akuno, W.; Akenga, T. | 2014 | | High fluoride water in Bondo-Rarieda area of Siaya County, Kenya: a hydro-geological implication on public health in the Lake Victoria Basin | | Duplicate |
| 1. Wambu, E. W.; Agong, S. G.; Anyango, B.; Akuno, W.; Akenga, T. | 2014 | | High fluoride water in Bondo-Rarieda area of Siaya County, Kenya: a hydro-geological implication on public health in the Lake Victoria Basin | | unrelated topic |
| 1. Ben Rekaya, M.; Messaoud, O.; Talmoudi, F.; Nouira, S.; Ouragini, H.; Amouri, A.; Boussen, H.; Boubaker, S.; Mokni, M.; Mokthar, I.; Abdelhak, S.; Zghal, M. | 2009 | | High frequency of the V548A fs X572 XPC mutation in Tunisia: implication for molecular diagnosis | | unrelated topic |
| 1. Walker, A. R.; Walker, B. F. | 1978 | | High high-density-lipoprotein cholesterol in African children and adults in a population free of coronary heart diseae | | Year of publication |
| 1. Karamagi, H. C.; Berhane, A.; Ngusbrhan Kidane, S.; Nyawira, L.; Ani-Amponsah, M.; Nyanjau, L.; Maoulana, K.; Seydi, A. B. W.; Nzinga, J.; Dangou, J. M.; Nkurunziza, T.; G, K. Bisoborwa; Sillah, J. S.; A, W. Muriithi; Nirina Razakasoa, H.; Bigirimana, F. | 2022 | | High impact health service interventions for attainment of UHC in Africa: A systematic review | | unrelated topic |
| 1. Mbehang Nguema, P. P.; Onanga, R.; Ndong Atome, G. R.; Tewa, J. J.; Mabika Mabika, A.; Muandze Nzambe, J. U.; Obague Mbeang, J. C.; Bitome Essono, P. Y.; Bretagnolle, F.; Godreuil, S. | 2021 | | High level of intrinsic phenotypic antimicrobial resistance in enterobacteria from terrestrial wildlife in Gabonese national parks | | unrelated topic |
| 1. Mushi, M. F.; Mtemisika, C. I.; Bader, O.; Bii, C.; Mirambo, M. M.; Groß, U.; Mshana, S. E. | 2016 | | High Oral Carriage of Non-albicans Candida spp. among HIV-infected individuals | | unrelated topic |
| 1. Racape, J.; De Spiegelaere, M.; Alexander, S.; Dramaix, M.; Buekens, P.; Haelterman, E. | 2010 | | High perinatal mortality rate among immigrants in Brussels | | unrelated topic |
| 1. Dixon, M.; Kancherla, V.; Magana, T.; Mulugeta, A.; Oakley, Jr., G. P. | 2019 | | High potential for reducing folic acid-preventable spina bifida and anencephaly, and related stillbirth and child mortality, in Ethiopia | | unrelated topic |
| 1. Magak, P.; King, C. H.; Ireri, E.; Kadzo, H.; Ouma, J. H.; Muchiri, E. M. | 2004 | | High prevalence of ectopic kidney in Coast Province, Kenya | | unrelated topic |
| 1. Ouedraogo, A. S.; Sanou, M.; Kissou, A.; Sanou, S.; Solaré, H.; Kaboré, F.; Poda, A.; Aberkane, S.; Bouzinbi, N.; Sano, I.; Nacro, B.; Sangaré, L.; Carrière, C.; Decré, D.; Ouégraogo, R.; Jean-Pierre, H.; Godreuil, S. | 2016 | | High prevalence of extended-spectrum ß-lactamase producing enterobacteriaceae among clinical isolates in Burkina Faso | | unrelated topic |
| 1. Namuyonga, J.; Lubega, S.; Aliku, T.; Omagino, J.; Sable, C.; Lwabi, P. | 2019 | | High prevalence of truncus arteriosus in pediatric congenital heart disease in Uganda | | unrelated topic |
| 1. Bediako-Bowan, A. A. A.; Kurtzhals, J. A. L.; Mølbak, K.; Labi, A. K.; Owusu, E.; Newman, M. J. | 2020 | | High rates of multi-drug resistant gram-negative organisms associated with surgical site infections in a teaching hospital in Ghana | | unrelated topic |
| 1. Funari, C. S.; Eugster, P. J.; Martel, S.; Carrupt, P. A.; Wolfender, J. L.; Silva, D. H. | 2012 | | High resolution ultra high pressure liquid chromatography-time-of-flight mass spectrometry dereplication strategy for the metabolite profiling of Brazilian Lippia species | | unrelated topic |
| 1. James, DK; Steer, PJ; Weiner, CP; Gonik, B | 2010 | | High risk pregnancy e-book: Management options-expert consult | | book |
| 1. Idemudia, J. O.; Idogun, E. S. | 2012 | | High sensitive C-reactive protein (HsCRP) as a cardiovascular risk factor in hypertensive Nigerians | | unrelated topic |
| 1. Neary, J.; Langat, A.; Singa, B.; Kinuthia, J.; Itindi, J.; Nyaboe, E.; Ng'anga, L. W.; Katana, A.; John-Stewart, G. C.; McGrath, C. J. | 2022 | | Higher prevalence of stunting and poor growth outcomes in HIV-exposed uninfected than HIV-unexposed infants in Kenya | | unrelated topic |
| 1. Adeloye, A. | 1977 | | Highlights of neurosurgery in Nigeria | | Year of publication |
| 1. Roper, A. | 1976 | | Hip dysplasia in the African Bantu | | Year of publication |
| 1. Archibong, A. E. | 2001 | | Hirschsprung's disease in children in South Eastern Nigeria | | unrelated topic |
| 1. Mabula, J. B.; Kayange, N. M.; Manyama, M.; Chandika, A. B.; Rambau, P. F.; Chalya, P. L. | 2014 | | Hirschsprung's disease in children: a five year experience at a university teaching hospital in northwestern Tanzania | | unrelated topic |
| 1. Bandré, E.; Kaboré, R. A.; Ouedraogo, I.; Soré, O.; Tapsoba, T.; Bambara, C.; Wandaogo, A. | 2010 | | Hirschsprung's disease: management problem in a developing country | | unrelated topic |
| 1. Saad, S. A.; Elseed, M. M. G.; AbouZeid, A. A.; Ibrahim, E. A.; Radwan, A. B.; Hay, S. A.; El-Behery, M. M. | 2020 | | Histopathological perspective of the pulled-through colon in Hirschsprung disease: Impact on clinical outcome | | unrelated topic |
| 1. Vachiat, A.; McCutcheon, K.; Tsabedze, N.; Zachariah, D.; Manga, P. | 2017 | | HIV and Ischemic Heart Disease | | unrelated topic |
| 1. Ajibola, G.; Leidner, J.; Mayondi, G. K.; van Widenfelt, E.; Madidimalo, T.; Petlo, C.; Moyo, S.; Mmalane, M.; Williams, P. L.; Cassidy, A. R.; Shapiro, R.; Kammerer, B.; Lockman, S. | 2018 | | HIV Exposure and Formula Feeding Predict Under-2 Mortality in HIV-Uninfected Children, Botswana | | unrelated topic |
| 1. Bai, Y. L.; Liu, H. B.; Sun, B.; Zhang, Y.; Li, Q.; Hu, C. W.; Zhu, J. X.; Gong, D. M.; Teng, X.; Zhang, Q.; Yang, B. F.; Dong, D. L. | 2011 | | HIV Tat protein inhibits hERG K+ channels: a potential mechanism of HIV infection induced LQTs | | Duplicate |
| 1. Bai, Y. L.; Liu, H. B.; Sun, B.; Zhang, Y.; Li, Q.; Hu, C. W.; Zhu, J. X.; Gong, D. M.; Teng, X.; Zhang, Q.; Yang, B. F.; Dong, D. L. | 2011 | | HIV Tat protein inhibits hERG K+ channels: a potential mechanism of HIV infection induced LQTs | | unrelated topic |
| 1. Moorjani, S.; Roy, M.; Gagné, C.; Davignon, J.; Brun, D.; Toussaint, M.; Lambert, M.; Campeau, L.; Blaichman, S.; Lupien, P. | 1989 | | Homozygous familial hypercholesterolemia among French Canadians in Québec Province | | Year of publication |
| 1. Jost, A. | 1970 | | Hormonal factors in the sex differentiation of the mammalian foetus | | Year of publication |
| 1. Tavani, A.; Bertuzzi, M.; Gallus, S.; Negri, E.; La Vecchia, C. | 2005 | | Hormone replacement therapy and risk of nonfatal acute myocardial infarction in Italy | | unrelated topic |
| 1. Wondimeneh, Y.; Tiruneh, M.; Ferede, G.; Denekew, K.; Admassu, F.; Tessema, B. | 2018 | | Hospital based surveillance of congenital rubella syndrome cases in the pre-vaccine era in Amhara Regional State, Ethiopia: A base line information for the country | | unrelated topic |
| 1. Choi, S.; Shin, H.; Heo, J.; Gedlu, E.; Nega, B.; Moges, T.; Bezabih, A.; Park, J.; Kim, W. H. | 2021 | | How do caregivers of children with congenital heart diseases access and navigate the healthcare system in Ethiopia? | | unrelated topic |
| 1. Caymaz, M. G.; Onoral, O. | 2020 | | How to address a biological complication by using advanced platelet-rich fibrin during the interdisciplinary management of congenitally missing maxillary lateral incisors | | bookSection |
| 1. Rosildo, J. F.; Dos Santos, M. F.; de Santa Barbara Rde, C. | 2015 | | Huge interparietal posterior fontanel meningohydroencephalocele | | bookSection |
| 1. Utkus, A.; Kazakevicius, R.; Ptasekas, R.; Kucinskas, V.; Beckwith, J. B.; Opitz, J. M. | 2001 | | Human anotocephaly (aprosopus, acrania-synotia) in the Vilnius anatomical collection | | unrelated topic |
| 1. Guidozzi, F.; Ballot, D.; Rothberg, A. D. | 1994 | | Human B19 parvovirus infection in an obstetric population. A prospective study determining fetal outcome | | Year of publication |
|  | 1982 | | Human guinea pigs have a choice | | Year of publication |
| 1. Blokzijl, M. L. | 1988 | | Human immunodeficiency virus infection in childhood | | Year of publication |
| 1. DeAsis, F.; Gitelis, M.; Chao, S.; Lapin, B.; Linn, J.; Denham, W.; Haggerty, S.; Carbray, J.; Ujiki, M.; Olory-Togbe, J. L.; Gbessi, D. G.; Dossou, F. M.; Lawani, I.; Souaibou, Y. I.; Gnangnon, I.; Denakpo, M.; Soton, R. R.; Djrouo, G.; Gogan, P.; Trukhalev, W.; Kukosh, M.; Panyushkin, A.; Safronova, E.; Jairam, A.; Kaufmann, R.; Jeekel, J.; Lange, J. F.; Volmer, U.; Kersten, C. C.; Arlt, G.; Skach, J.; Harcubova, R.; Petrakova, V.; Mandoboy, J. D.; Ngom, G.; Faye, A. L.; Ndour, O.; Sankale, A. A.; Ndoye, M.; Daneiii, P.; Leone, N.; Ballerini, A.; Bondurri, A.; Cavallaro, G.; Silecchia, G.; Raparelli, L.; Greco, F.; Iorio, O.; Iossa, A.; De Angelis, F.; Rizzello, M.; Olmi, S.; Cesana, G.; Baldazzi, G.; Manoocheri, F.; Campanile, F. C.; Munipalle, P.; Khan, S.; Gwiti, P.; Kanakala, V.; Viswanath, Y.; Kokotovic, D.; Sjølander, H.; Gögenur, I.; Helgstrand, F.; Devadhar, S.; Hounnou, G.; Elegbede, O. T.; Hadonou, A. A.; Mensah, E. D.; Agossou-Voyeme, A. K.; Konate, I.; Toure, A. O.; Cisse, M.; Zaki, M.; Diao, M. L.; Tendeng, J. N.; Toure, F. B.; Toure, C. T.; Subramanian, V.; Froghi, F.; de Carvalho, F. C.; Salimin, L.; Drabble, E. | 2015 | | Humbilical & Epigastric Hernia | | unrelated topic |
| 1. Magill, AJ; Strickland, GT; Maguire, JH; Ryan, ET; ... | 2012 | | Hunter's Tropical Medicine and Emerging Infectious Disease E-Book | | book |
| 1. Ryan, ET; Hill, DR; Solomon, T; Aronson, N; Endy, TP | 2019 | | Hunter's tropical medicine and emerging infectious diseases | | book |
| 1. Elhence, P.; Sinha, A. | 2020 | | Hyaline cartilage at porta hepatis in extrahepatic biliary atresia: metaplasia or choristoma | | unrelated topic |
| 1. Iqbal, K.; Iqbal, A.; Kirillov, A. M.; Liu, W.; Tang, Y. | 2018 | | Hybrid Metal-Organic-Framework/Inorganic Nanocatalyst toward Highly Efficient Discoloration of Organic Dyes in Aqueous Medium | | unrelated topic |
| 1. Xie, F.; Huang, L.; Ye, Y.; Hao, J.; Lv, J.; Richard, S. A. | 2022 | | Hybrid operation for arteriovenous malformations with associated multiple intracranial aneurysms and subarachnoid hemorrhage: Case report | | unrelated topic |
| 1. Schneebeli, M.; Inoue, S.; Madarame, H. | 1993 | | Hydranencephaly in newborn calves in Zambia | | Year of publication |
| 1. Warf, B. C. | 2011 | | Hydrocephalus associated with neural tube defects: characteristics, management, and outcome in sub-Saharan Africa | | unrelated topic |
| 1. Seligson, D.; Levy, L. F. | 1974 | | Hydrocephalus in a developing country: a ten-year experience | | Year of publication |
| 1. Seligson, D.; Levy, L. F. | 1974 | | Hydrocephalus in a developing country: a ten-year experience | | Year of publication |
| 1. Beck, J.; Lipschitz, R. | 1969 | | Hydrocephalus in African children: a survey of 3 years' experience at Baragwanath Hospital | | Year of publication |
| 1. Peacock, W. J.; Currer, T. H. | 1984 | | Hydrocephalus in childhood. A study of 440 cases | | Year of publication |
| 1. de Ravel, T. J.; van der Griendt, M. C.; Evan, P.; Wright, C. A. | 1999 | | Hydrolethalus syndrome in a non-Finnish family: confirmation of the entity and early prenatal diagnosis | | Year of publication |
| 1. Gaye, M.; Amanzougaghene, N.; Laidoudi, Y.; Niang, E. H. A.; Sekeyová, Z.; Laroche, M.; Bérenger, J. M.; Raoult, D.; Kazimírová, M.; Fenollar, F.; Mediannikov, O. | 2020 | | Hymenopteran Parasitoids of Hard Ticks in Western Africa and the Russian Far East | | unrelated topic |
| 1. Steyn, K.; Benadé, A. J.; Langenhoven, M. L.; Joubert, G.; Rossouw, J. E. | 1987 | | Hypercholesterolaemia in the coloured population of the Cape Peninsula (CRISIC study) | | Year of publication |
| 1. Irish, J. D. | 2022 | | Hyperdontia across sub-Saharan Africa: Prevalence, patterning, and implications | | unrelated topic |
| 1. Ramlakhan, K. P.; Malhamé, I.; Marelli, A.; Rutz, T.; Goland, S.; Franx, A.; Sliwa, K.; Elkayam, U.; Johnson, M. R.; Hall, R.; Cornette, J.; Roos-Hesselink, J. W. | 2022 | | Hypertensive disorders of pregnant women with heart disease: the ESC EORP ROPAC Registry | | unrelated topic |
| 1. Edwards, M. J. | 1986 | | Hyperthermia as a teratogen: a review of experimental studies and their clinical significance | | Year of publication |
| 1. Potgieter, H.; van der Westhuizen, R.; Rohwer, E.; Malan, D. | 2013 | | Hyphenation of supercritical fluid chromatography and two-dimensional gas chromatography-mass spectrometry for group type separations | | unrelated topic |
| 1. Hargreaves, J. A.; Cleaton-Jones, P. E.; Williams, S. D. | 1989 | | Hypocalcification and hypoplasia in permanent teeth of children from different ethnic groups in South Africa assessed with a new index | | Year of publication |
| 1. Hargreaves, J. A.; Cleaton-Jones, P. E.; Roberts, G. J.; Williams, S. D. | 1989 | | Hypocalcification and hypoplasia in primary teeth of pre-school children from different ethnic groups in South Africa | | Year of publication |
| 1. Ng'ang'a, R. N.; Ng'ang'a, P. M. | 2001 | | Hypodontia of permanent teeth in a Kenyan population | | unrelated topic |
| 1. Norval, E. J.; van Wyk, C. W.; Basson, N. J.; Coldrey, J. | 1988 | | Hypohidrotic ectodermal dysplasia: a genealogic, stereomicroscope, and scanning electron microscope study | | Year of publication |
| 1. Oyedele, T. A.; Folayan, M. O.; Oziegbe, E. O. | 2016 | | Hypomineralised second primary molars: prevalence, pattern and associated co morbidities in 8- to 10-year-old children in Ile-Ife, Nigeria | | unrelated topic |
| 1. Ensor, B. E.; Irish, J. D. | 1995 | | Hypoplastic area method for analyzing dental enamel hypoplasia | | Year of publication |
| 1. Abdelrahman, M. Y.; Abdeljaleel, I. A.; Mohamed, E.; Bagadi, A. T.; Khair, O. E. | 2011 | | Hypospadias in Sudan, clinical and surgical review | | unrelated topic |
| 1. Aisuodionoe-Shadrach, O. I.; Atim, T.; Eniola, B. S.; Ohemu, A. A. | 2015 | | Hypospadias repair and outcome in Abuja, Nigeria: A 5-year single-centre experience | | Duplicate |
| 1. Aisuodionoe-Shadrach, O. I.; Atim, T.; Eniola, B. S.; Ohemu, A. A. | 2015 | | Hypospadias repair and outcome in Abuja, Nigeria: A 5-year single-centre experience | | unrelated topic |
| 1. Mammo, T. N.; Negash, S. A.; Negussie, T.; Getachew, H.; Dejene, B.; Tadesse, A.; Derbew, M. | 2018 | | Hypospadias Repair in Ethiopia: A Five Year Review | | Duplicate |
| 1. Mammo, T. N.; Negash, S. A.; Negussie, T.; Getachew, H.; Dejene, B.; Tadesse, A.; Derbew, M. | 2018 | | Hypospadias Repair in Ethiopia: A Five Year Review | | unrelated topic |
| 1. Renwick, J. H. | 1972 | | Hypothesis: anencephaly and spina bifida are usually preventable by avoidance of a specific but unidentified substance present in certain potato tubers | | Year of publication |
| 1. Kitilla, T. | 2010 | | Hysterosalpingography in the evaluation of infertility: a five years review. (FGAE, 2001 -5) | | unrelated topic |
| 1. Przybojewski, J. Z. | 1982 | | Iatrogenic aortocoronary vein fistula. A case presentation and review of the literature | | Year of publication |
| 1. Schmidt, T.; Kock, M. M.; Ehlers, M. M. | 2018 | | Identification and characterization of Staphylococcus devriesei isolates from bovine intramammary infections in KwaZulu-Natal, South Africa | | unrelated topic |
| 1. Carlson, J. C.; Standley, J.; Petrin, A.; Shaffer, J. R.; Butali, A.; Buxó, C. J.; Castilla, E.; Christensen, K.; Deleyiannis, F. W.; Hecht, J. T.; Field, L. L.; Garidkhuu, A.; Moreno Uribe, L. M.; Nagato, N.; Orioli, I. M.; Padilla, C.; Poletta, F.; Suzuki, S.; Vieira, A. R.; Wehby, G. L.; Weinberg, S. M.; Beaty, T. H.; Feingold, E.; Murray, J. C.; Marazita, M. L.; Leslie, E. J. | 2017 | | Identification of 16q21 as a modifier of nonsyndromic orofacial cleft phenotypes | | unrelated topic |
| 1. Zhong, M.; Huang, L. N.; Zhang, S. J.; Yan, S. J. | 2022 | | Identification of a novel mutation in ALMS1 in a Chinese patient with monogenic diabetic syndrome by whole-exome sequencing | | bookSection |
| 1. Zhang, W.; Liu, J.; Chen, Q.; Ding, W.; Li, S.; Ma, L. | 2022 | | Identification of ADP/ATP Translocase 1 as a Novel Glycoprotein and Its Association with Parkinson's Disease | | unrelated topic |
| 1. Niare, S.; Berenger, J. M.; Dieme, C.; Doumbo, O.; Raoult, D.; Parola, P.; Almeras, L. | 2016 | | Identification of blood meal sources in the main African malaria mosquito vector by MALDI-TOF MS | | unrelated topic |
| 1. Adam, O.; Ali, A. K.; Hübschen, J. M.; Muller, C. P. | 2014 | | Identification of congenital rubella syndrome in Sudan | | unrelated topic |
| 1. Briolant, S.; Costa, M. M.; Nguyen, C.; Dusfour, I.; Pommier de Santi, V.; Girod, R.; Almeras, L. | 2020 | | Identification of French Guiana anopheline mosquitoes by MALDI-TOF MS profiling using protein signatures from two body parts | | unrelated topic |
| 1. Predel, R.; Gäde, G. | 2002 | | Identification of the abundant neuropeptide from abdominal perisympathetic organs of locusts | | unrelated topic |
| 1. Govender, R.; Amoah, I. D.; Adegoke, A. A.; Singh, G.; Kumari, S.; Swalaha, F. M.; Bux, F.; Stenström, T. A. | 2021 | | Identification, antibiotic resistance, and virulence profiling of Aeromonas and Pseudomonas species from wastewater and surface water | | unrelated topic |
| 1. Millow, C. J.; Mackintosh, S. A.; Lewison, R. L.; Dodder, N. G.; Hoh, E. | 2015 | | Identifying bioaccumulative halogenated organic compounds using a nontargeted analytical approach: seabirds as sentinels | | unrelated topic |
| 1. Jit, I. | 1988 | | Ileal band in northwest Indian subjects | | Year of publication |
[truncated: 429,539 more chars]
