# Supplementary material for: Pooled prevalence and subgroup variations of Tetralogy of Fallot among children and adolescents with congenital heart defect in Sub-Saharan Africa: A systematic review and meta-analysis
Source: PLoS One. 2025 Jan 17;20(1):e0311686. doi: 10.1371/journal.pone.0311686 (PMC11741593; doi:10.1371/journal.pone.0311686)
Supplement: S3 Table — (DOCX) [file pone.0311686.s003.docx]

| **Quality Assessment Result for all included studies**   1. **Cross-sectional studies** | | |  |  |
| --- | --- | --- | --- | --- |
| 1. Mazhani et al, 2020 |  |  |  |  |
| JBI CHECKLIST FOR  PREVALENCE STUDIES | **Yes** | **No** | **Unclear** | **Not applicable** |
| 1.         Was the sample frame appropriate to address the target population? | √ |  |  |  |
| 2.         Were study participants sampled in an appropriate way? |  | √ |  |  |
| 3.         Was the sample size adequate? | √ |  |  |  |
| 4.         Were the study subjects and the setting described in detail? | √ |  |  |  |
| 5.         Was the data analysis conducted with sufficient coverage of the identified sample? |  | √ |  |  |
| 6.         Were valid methods used for the identification of the condition? | √ |  |  |  |
| 7.         Was the condition measured in a standard, reliable way for all participants? | √ |  |  |  |
| 8.         Was there appropriate statistical analysis? | √ |  |  |  |
| 9.       Was the response rate adequate, and if not, was the low response rate managed appropriately? |  | √ |  |  |
| Total | 6 | 3 |  |  |
| 1. Puepi 2022 |  |  |  |  |
| JBI CHECKLIST FOR  PREVALENCE STUDIES | **Yes** | **No** | **Unclear** | **Not applicable** |
| 1.         Was the sample frame appropriate to address the target population? |  | √ |  |  |
| 2.         Were study participants sampled in an appropriate way? |  | √ |  |  |
| 3.         Was the sample size adequate? |  | √ |  |  |
| 4.         Were the study subjects and the setting described in detail? | √ |  |  |  |
| 5.         Was the data analysis conducted with sufficient coverage of the identified sample? | √ |  |  |  |
| 6.         Were valid methods used for the identification of the condition? | √ |  |  |  |
| 7.         Was the condition measured in a standard, reliable way for all participants? | √ |  |  |  |
| 8.         Was there appropriate statistical analysis? | √ |  |  |  |
| 9.       Was the response rate adequate, and if not, was the low response rate managed appropriately? | √ |  |  |  |
| Total | 6 | 3 |  |  |
| 3. Nkoke et al 2017 |  |  |  |  |
| JBI CHECKLIST FOR  PREVALENCE STUDIES | **Yes** | **No** | **Unclear** | **Not applicable** |
| 1.         Was the sample frame appropriate to address the target population? |  | √ |  |  |
| 2.         Were study participants sampled in an appropriate way? |  | √ |  |  |
| 3.         Was the sample size adequate? | √ |  |  |  |
| 4.         Were the study subjects and the setting described in detail? | √ |  |  |  |
| 5.         Was the data analysis conducted with sufficient coverage of the identified sample? | √ |  |  |  |
| 6.         Were valid methods used for the identification of the condition? | √ |  |  |  |
| 7.         Was the condition measured in a standard, reliable way for all participants? | √ |  |  |  |
| 8.         Was there appropriate statistical analysis? | √ |  |  |  |
| 9.       Was the response rate adequate, and if not, was the low response rate managed appropriately? | √ |  |  |  |
| Total | 7 | 2 |  |  |
| 4. Kamdem et al 2018 |  |  |  |  |
| JBI CHECKLIST FOR  PREVALENCE STUDIES | **Yes** | **No** | **Unclear** | **Not applicable** |
| 1.         Was the sample frame appropriate to address the target population? | √ |  |  |  |
| 2.         Were study participants sampled in an appropriate way? |  | √ |  |  |
| 3.         Was the sample size adequate? | √ |  |  |  |
| 4.         Were the study subjects and the setting described in detail? | √ |  |  |  |
| 5.         Was the data analysis conducted with sufficient coverage of the identified sample? | √ |  |  |  |
| 6.         Were valid methods used for the identification of the condition? | √ |  |  |  |
| 7.         Was the condition measured in a standard, reliable way for all participants? | √ |  |  |  |
| 8.         Was there appropriate statistical analysis? | √ |  |  |  |
| 9.       Was the response rate adequate, and if not, was the low response rate managed appropriately? | √ |  |  |  |
| Total | 8 | 1 |  |  |
| 5. Chelo et al 2016 |  |  |  |  |
| JBI CHECKLIST FOR  PREVALENCE STUDIES | **Yes** | **No** | **Unclear** | **Not applicable** |
| 1.         Was the sample frame appropriate to address the target population? | √ |  |  |  |
| 2.         Were study participants sampled in an appropriate way? |  | √ |  |  |
| 3.         Was the sample size adequate? | √ |  |  |  |
| 4.         Were the study subjects and the setting described in detail? | √ |  |  |  |
| 5.         Was the data analysis conducted with sufficient coverage of the identified sample? | √ |  |  |  |
| 6.         Were valid methods used for the identification of the condition? | √ |  |  |  |
| 7.         Was the condition measured in a standard, reliable way for all participants? | √ |  |  |  |
| 8.         Was there appropriate statistical analysis? | √ |  |  |  |
| 9.       Was the response rate adequate, and if not, was the low response rate managed appropriately? | √ |  |  |  |
| Total | 8 | 1 |  |  |
| 6. Mehadi et al 2006 |  |  |  |  |
| JBI CHECKLIST FOR  PREVALENCE STUDIES | **Yes** | **No** | **Unclear** | **Not applicable** |
| 1.         Was the sample frame appropriate to address the target population? | √ |  |  |  |
| 2.         Were study participants sampled in an appropriate way? |  | √ |  |  |
| 3.         Was the sample size adequate? | √ |  |  |  |
| 4.         Were the study subjects and the setting described in detail? |  | √ |  |  |
| 5.         Was the data analysis conducted with sufficient coverage of the identified sample? | √ |  |  |  |
| 6.         Were valid methods used for the identification of the condition? | √ |  |  |  |
| 7.         Was the condition measured in a standard, reliable way for all participants? |  | √ |  |  |
| 8.         Was there appropriate statistical analysis? | √ |  |  |  |
| 9.       Was the response rate adequate, and if not, was the low response rate managed appropriately? | √ |  |  |  |
| Total | 6 | 3 |  |  |
| 7. Talarge et al 2018 |  |  |  |  |
| JBI CHECKLIST FOR  PREVALENCE STUDIES | **Yes** | **No** | **Unclear** | **Not applicable** |
| 1.         Was the sample frame appropriate to address the target population? | √ |  |  |  |
| 2.         Were study participants sampled in an appropriate way? | √ |  |  |  |
| 3.         Was the sample size adequate? | √ |  |  |  |
| 4.         Were the study subjects and the setting described in detail? |  | √ |  |  |
| 5.         Was the data analysis conducted with sufficient coverage of the identified sample? | √ |  |  |  |
| 6.         Were valid methods used for the identification of the condition? | √ |  |  |  |
| 7.         Was the condition measured in a standard, reliable way for all participants? |  | √ |  |  |
| 8.         Was there appropriate statistical analysis? | √ |  |  |  |
| 9.       Was the response rate adequate, and if not, was the low response rate managed appropriately? | √ |  |  |  |
| Total | 7 | 2 |  |  |
| 8. Malede et al 2006 |  |  |  |  |
| JBI CHECKLIST FOR  PREVALENCE STUDIES | **Yes** | **No** | **Unclear** | **Not applicable** |
| 1.         Was the sample frame appropriate to address the target population? | √ |  |  |  |
| 2.         Were study participants sampled in an appropriate way? |  | √ |  |  |
| 3.         Was the sample size adequate? |  | √ |  |  |
| 4.         Were the study subjects and the setting described in detail? |  | √ |  |  |
| 5.         Was the data analysis conducted with sufficient coverage of the identified sample? |  | √ |  |  |
| 6.         Were valid methods used for the identification of the condition? | √ |  |  |  |
| 7.         Was the condition measured in a standard, reliable way for all participants? | √ |  |  |  |
| 8.         Was there appropriate statistical analysis? | √ |  |  |  |
| 9.       Was the response rate adequate, and if not, was the low response rate managed appropriately? | √ |  |  |  |
| Total | 5 | 4 |  |  |
| 9. Tsega et al 2022 |  |  |  |  |
| JBI CHECKLIST FOR  PREVALENCE STUDIES | **Yes** | **No** | **Unclear** | **Not applicable** |
| 1.         Was the sample frame appropriate to address the target population? | √ |  |  |  |
| 2.         Were study participants sampled in an appropriate way? | √ |  |  |  |
| 3.         Was the sample size adequate? | √ |  |  |  |
| 4.         Were the study subjects and the setting described in detail? |  | √ |  |  |
| 5.         Was the data analysis conducted with sufficient coverage of the identified sample? | √ |  |  |  |
| 6.         Were valid methods used for the identification of the condition? | √ |  |  |  |
| 7.         Was the condition measured in a standard, reliable way for all participants? | √ |  |  |  |
| 8.         Was there appropriate statistical analysis? | √ |  |  |  |
| 9.       Was the response rate adequate, and if not, was the low response rate managed appropriately? | √ |  |  |  |
| Total | 8 | 1 |  |  |
| 10. Awori 2013 |  |  |  |  |
| JBI CHECKLIST FOR  PREVALENCE STUDIES | **Yes** | **No** | **Unclear** | **Not applicable** |
| 1.         Was the sample frame appropriate to address the target population? | √ |  |  |  |
| 2.         Were study participants sampled in an appropriate way? | √ |  |  |  |
| 3.         Was the sample size adequate? | √ |  |  |  |
| 4.         Were the study subjects and the setting described in detail? |  | √ |  |  |
| 5.         Was the data analysis conducted with sufficient coverage of the identified sample? |  | √ |  |  |
| 6.         Were valid methods used for the identification of the condition? | √ |  |  |  |
| 7.         Was the condition measured in a standard, reliable way for all participants? | √ |  |  |  |
| 8.         Was there appropriate statistical analysis? | √ |  |  |  |
| 9.       Was the response rate adequate, and if not, was the low response rate managed appropriately? | √ |  |  |  |
| Total | 7 | 2 |  |  |
| 11. Kennedy et al 2013 |  |  |  |  |
| JBI CHECKLIST FOR  PREVALENCE STUDIES | **Yes** | **No** | **Unclear** | **Not applicable** |
| 1.         Was the sample frame appropriate to address the target population? | √ |  |  |  |
| 2.         Were study participants sampled in an appropriate way? | √ |  |  |  |
| 3.         Was the sample size adequate? | √ |  |  |  |
| 4.         Were the study subjects and the setting described in detail? | √ |  |  |  |
| 5.         Was the data analysis conducted with sufficient coverage of the identified sample? |  | √ |  |  |
| 6.         Were valid methods used for the identification of the condition? | √ |  |  |  |
| 7.         Was the condition measured in a standard, reliable way for all participants? | √ |  |  |  |
| 8.         Was there appropriate statistical analysis? | √ |  |  |  |
| 9.       Was the response rate adequate, and if not, was the low response rate managed appropriately? | √ |  |  |  |
| Total | 8 | 1 |  |  |
| 12. Zakaria et al 2021 |  |  |  |  |
| JBI CHECKLIST FOR  PREVALENCE STUDIES | **Yes** | **No** | **Unclear** |  |
| 1.         Was the sample frame appropriate to address the target population? | √ |  |  |  |
| 2.         Were study participants sampled in an appropriate way? | √ |  |  |  |
| 3.         Was the sample size adequate? | √ |  |  |  |
| 4.         Were the study subjects and the setting described in detail? |  | √ |  |  |
| 5.         Was the data analysis conducted with sufficient coverage of the identified sample? |  | √ |  |  |
| 6.         Were valid methods used for the identification of the condition? | √ |  |  |  |
| 7.         Was the condition measured in a standard, reliable way for all participants? | √ |  |  |  |
| 8.         Was there appropriate statistical analysis? | √ |  |  |  |
| 9.       Was the response rate adequate, and if not, was the low response rate managed appropriately? | √ |  |  |  |
| Total | 7 | 2 |  |  |
| 13. Chinawa et al 2013 |  |  |  |  |
| JBI CHECKLIST FOR  PREVALENCE STUDIES | **Yes** | **No** | **Unclear** |  |
| 1.         Was the sample frame appropriate to address the target population? | √ |  |  |  |
| 2.         Were study participants sampled in an appropriate way? | √ |  |  |  |
| 3.         Was the sample size adequate? |  | √ |  |  |
| 4.         Were the study subjects and the setting described in detail? | √ |  |  |  |
| 5.         Was the data analysis conducted with sufficient coverage of the identified sample? | √ |  |  |  |
| 6.         Were valid methods used for the identification of the condition? | √ |  |  |  |
| 7.         Was the condition measured in a standard, reliable way for all participants? | √ |  |  |  |
| 8.         Was there appropriate statistical analysis? | √ |  |  |  |
| 9.       Was the response rate adequate, and if not, was the low response rate managed appropriately? | √ |  |  |  |
| Total | 8 | 1 |  |  |
| 14. Ekure et al 2009 |  |  |  |  |
| JBI CHECKLIST FOR  PREVALENCE STUDIES | **Yes** | **No** | **Unclear** |  |
| 1.         Was the sample frame appropriate to address the target population? | √ |  |  |  |
| 2.         Were study participants sampled in an appropriate way? | √ |  |  |  |
| 3.         Was the sample size adequate? |  | √ |  |  |
| 4.         Were the study subjects and the setting described in detail? |  | √ |  |  |
| 5.         Was the data analysis conducted with sufficient coverage of the identified sample? | √ |  |  |  |
| 6.         Were valid methods used for the identification of the condition? | √ |  |  |  |
| 7.         Was the condition measured in a standard, reliable way for all participants? | √ |  |  |  |
| 8.         Was there appropriate statistical analysis? | √ |  |  |  |
| 9.       Was the response rate adequate, and if not, was the low response rate managed appropriately? | √ |  |  |  |
| Total | 7 | 2 |  |  |
| 15. Adebayo et al 2016 |  |  |  |  |
| JBI CHECKLIST FOR  PREVALENCE STUDIES | **Yes** | **No** | **Unclear** |  |
| 1.         Was the sample frame appropriate to address the target population? | √ |  |  |  |
| 2.         Were study participants sampled in an appropriate way? | √ |  |  |  |
| 3.         Was the sample size adequate? | √ |  |  |  |
| 4.         Were the study subjects and the setting described in detail? |  | √ |  |  |
| 5.         Was the data analysis conducted with sufficient coverage of the identified sample? |  | √ |  |  |
| 6.         Were valid methods used for the identification of the condition? | √ |  |  |  |
| 7.         Was the condition measured in a standard, reliable way for all participants? | √ |  |  |  |
| 8.         Was there appropriate statistical analysis? | √ |  |  |  |
| 9.       Was the response rate adequate, and if not, was the low response rate managed appropriately? | √ |  |  |  |
| Total | 7 | 2 |  |  |
| 16. Sadoh et al 2013 |  |  |  |  |
| JBI CHECKLIST FOR  PREVALENCE STUDIES | **Yes** | **No** |  |  |
| 1.         Was the sample frame appropriate to address the target population? | √ |  |  |  |
| 2.         Were study participants sampled in an appropriate way? | √ |  |  |  |
| 3.         Was the sample size adequate? | √ |  |  |  |
| 4.         Were the study subjects and the setting described in detail? |  | √ |  |  |
| 5.         Was the data analysis conducted with sufficient coverage of the identified sample? | √ |  |  |  |
| 6.         Were valid methods used for the identification of the condition? | √ |  |  |  |
| 7.         Was the condition measured in a standard, reliable way for all participants? | √ |  |  |  |
| 8.         Was there appropriate statistical analysis? | √ |  |  |  |
| 9.       Was the response rate adequate, and if not, was the low response rate managed appropriately? | √ |  |  |  |
| Total | 8 | 1 |  |  |
| 17. Chinawa et al 2021 |  |  |  |  |
| JBI CHECKLIST FOR  PREVALENCE STUDIES | **Yes** | **No** |  |  |
| 1.         Was the sample frame appropriate to address the target population? | √ |  |  |  |
| 2.         Were study participants sampled in an appropriate way? |  | √ |  |  |
| 3.         Was the sample size adequate? | √ |  |  |  |
| 4.         Were the study subjects and the setting described in detail? | √ |  |  |  |
| 5.         Was the data analysis conducted with sufficient coverage of the identified sample? | √ |  |  |  |
| 6.         Were valid methods used for the identification of the condition? | √ |  |  |  |
| 7.         Was the condition measured in a standard, reliable way for all participants? | √ |  |  |  |
| 8.         Was there appropriate statistical analysis? | √ |  |  |  |
| 9.       Was the response rate adequate, and if not, was the low response rate managed appropriately? | √ |  |  |  |
| Total | 8 | 1 |  |  |
| 18. Ekure et al 2018 |  |  |  |  |
| JBI CHECKLIST FOR  PREVALENCE STUDIES | **Yes** | **No** |  |  |
| 1.         Was the sample frame appropriate to address the target population? | √ |  |  |  |
| 2.         Were study participants sampled in an appropriate way? |  | √ |  |  |
| 3.         Was the sample size adequate? | √ |  |  |  |
| 4.         Were the study subjects and the setting described in detail? |  | √ |  |  |
| 5.         Was the data analysis conducted with sufficient coverage of the identified sample? | √ |  |  |  |
| 6.         Were valid methods used for the identification of the condition? | √ |  |  |  |
| 7.         Was the condition measured in a standard, reliable way for all participants? | √ |  |  |  |
| 8.         Was there appropriate statistical analysis? | √ |  |  |  |
| 9.       Was the response rate adequate, and if not, was the low response rate managed appropriately? | √ |  |  |  |
| Total | 7 | 2 |  |  |
| 19. Ekure et al 2017 |  |  |  |  |
| JBI CHECKLIST FOR  PREVALENCE STUDIES | **Yes** | **No** |  |  |
| 1.         Was the sample frame appropriate to address the target population? | √ |  |  |  |
| 2.         Were study participants sampled in an appropriate way? |  | √ |  |  |
| 3.         Was the sample size adequate? | √ |  |  |  |
| 4.         Were the study subjects and the setting described in detail? | √ |  |  |  |
| 5.         Was the data analysis conducted with sufficient coverage of the identified sample? |  | √ |  |  |
| 6.         Were valid methods used for the identification of the condition? | √ |  |  |  |
| 7.         Was the condition measured in a standard, reliable way for all participants? | √ |  |  |  |
| 8.         Was there appropriate statistical analysis? |  | √ |  |  |
| 9.       Was the response rate adequate, and if not, was the low response rate managed appropriately? | √ |  |  |  |
| Total | 6 | 3 |  |  |
| 20. Animasahun et al 2016 |  |  |  |  |
| JBI CHECKLIST FOR  PREVALENCE STUDIES | **Yes** | **No** |  |  |
| 1.         Was the sample frame appropriate to address the target population? | √ |  |  |  |
| 2.         Were study participants sampled in an appropriate way? |  | √ |  |  |
| 3.         Was the sample size adequate? | √ |  |  |  |
| 4.         Were the study subjects and the setting described in detail? | √ |  |  |  |
| 5.         Was the data analysis conducted with sufficient coverage of the identified sample? | √ |  |  |  |
| 6.         Were valid methods used for the identification of the condition? | √ |  |  |  |
| 7.         Was the condition measured in a standard, reliable way for all participants? | √ |  |  |  |
| 8.         Was there appropriate statistical analysis? | √ |  |  |  |
| 9.       Was the response rate adequate, and if not, was the low response rate managed appropriately? | √ |  |  |  |
| Total | 8 | 1 |  |  |
| 21. Coundoul et al 2023 |  |  |  |  |
| JBI CHECKLIST FOR  PREVALENCE STUDIES | **Yes** | **No** |  |  |
| 1.         Was the sample frame appropriate to address the target population? |  | √ |  |  |
| 2.         Were study participants sampled in an appropriate way? |  | √ |  |  |
| 3.         Was the sample size adequate? |  | √ |  |  |
| 4.         Were the study subjects and the setting described in detail? |  | √ |  |  |
| 5.         Was the data analysis conducted with sufficient coverage of the identified sample? | √ |  |  |  |
| 6.         Were valid methods used for the identification of the condition? | √ |  |  |  |
| 7.         Was the condition measured in a standard, reliable way for all participants? | √ |  |  |  |
| 8.         Was there appropriate statistical analysis? | √ |  |  |  |
| 9.       Was the response rate adequate, and if not, was the low response rate managed appropriately? | √ |  |  |  |
| Total | 5 | 4 |  |  |
| 22. Yusuf et al 2021 |  |  |  |  |
| JBI CHECKLIST FOR  PREVALENCE STUDIES | **Yes** | **No** |  |  |
| 1.         Was the sample frame appropriate to address the target population? | √ |  |  |  |
| 2.         Were study participants sampled in an appropriate way? |  | √ |  |  |
| 3.         Was the sample size adequate? | √ |  |  |  |
| 4.         Were the study subjects and the setting described in detail? |  | √ |  |  |
| 5.         Was the data analysis conducted with sufficient coverage of the identified sample? | √ |  |  |  |
| 6.         Were valid methods used for the identification of the condition? | √ |  |  |  |
| 7.         Was the condition measured in a standard, reliable way for all participants? | √ |  |  |  |
| 8.         Was there appropriate statistical analysis? | √ |  |  |  |
| 9.       Was the response rate adequate, and if not, was the low response rate managed appropriately? | √ |  |  |  |
| Total | 7 | 2 |  |  |
| 23. Ibrahim et al 2012 |  |  |  |  |
| JBI CHECKLIST FOR  PREVALENCE STUDIES | **Yes** | **No** |  |  |
| 1.         Was the sample frame appropriate to address the target population? | √ |  |  |  |
| 2.         Were study participants sampled in an appropriate way? |  | √ |  |  |
| 3.         Was the sample size adequate? | √ |  |  |  |
| 4.         Were the study subjects and the setting described in detail? | √ |  |  |  |
| 5.         Was the data analysis conducted with sufficient coverage of the identified sample? |  | √ |  |  |
| 6.         Were valid methods used for the identification of the condition? | √ |  |  |  |
| 7.         Was the condition measured in a standard, reliable way for all participants? | √ |  |  |  |
| 8.         Was there appropriate statistical analysis? | √ |  |  |  |
| 9.       Was the response rate adequate, and if not, was the low response rate managed appropriately? | √ |  |  |  |
| Total | 7 | 2 |  |  |
| 24. Abdelrahman et al 2022 |  |  |  |  |
| JBI CHECKLIST FOR  PREVALENCE STUDIES | **Yes** | **No** |  |  |
| 1.         Was the sample frame appropriate to address the target population? | √ |  |  |  |
| 2.         Were study participants sampled in an appropriate way? |  | √ |  |  |
| 3.         Was the sample size adequate? | √ |  |  |  |
| 4.         Were the study subjects and the setting described in detail? |  | √ |  |  |
| 5.         Was the data analysis conducted with sufficient coverage of the identified sample? | √ |  |  |  |
| 6.         Were valid methods used for the identification of the condition? | √ |  |  |  |
| 7.         Was the condition measured in a standard, reliable way for all participants? | √ |  |  |  |
| 8.         Was there appropriate statistical analysis? | √ |  |  |  |
| 9.       Was the response rate adequate, and if not, was the low response rate managed appropriately? | √ |  |  |  |
| Total | 7 | 2 |  |  |
| 25. Aliku et al 2021 |  |  |  |  |
| JBI CHECKLIST FOR  PREVALENCE STUDIES | **Yes** | **No** |  |  |
| 1.         Was the sample frame appropriate to address the target population? | √ |  |  |  |
| 2.         Were study participants sampled in an appropriate way? | √ |  |  |  |
| 3.         Was the sample size adequate? | √ |  |  |  |
| 4.         Were the study subjects and the setting described in detail? |  | √ |  |  |
| 5.         Was the data analysis conducted with sufficient coverage of the identified sample? | √ |  |  |  |
| 6.         Were valid methods used for the identification of the condition? | √ |  |  |  |
| 7.         Was the condition measured in a standard, reliable way for all participants? | √ |  |  |  |
| 8.         Was there appropriate statistical analysis? | √ |  |  |  |
| 9.       Was the response rate adequate, and if not, was the low response rate managed appropriately? | √ |  |  |  |
| Total | 8 | 1 |  |  |
| **B. Cohort studies** | |  |  |  |
| 1. Loufoua-Lemay et al 2016 |  |  |  |  |
| JBI checklist for cohort studies | **Yes** | **No** | **Unclear** | **Not applicable** |
| 1.       Were the two groups similar and recruited from the same population? | √ |  |  |  |
| 2.       Were the exposures measured similarly to assign people to both exposed and unexposed groups? | √ |  |  |  |
| 3.       Was the exposure measured in a valid and reliable way? |  | √ |  |  |
| 4.       Were confounding factors identified? | √ |  |  |  |
| 5.       Were strategies to deal with confounding factors stated? | √ |  |  |  |
| 6.       Were the groups/participants free of the outcome at the start of the study (or at the moment of exposure)? |  |  | √ |  |
| 7.       Were the outcomes measured in a valid and reliable way? | √ |  |  |  |
| 8.       Was the follow up time reported and sufficient to be long enough for outcomes to occur? |  | √ |  |  |
| 9.       Was follow up complete, and if not, were the reasons to loss to follow up described and explored? |  | √ |  |  |
| 10.   Were strategies to address incomplete follow up utilized? |  | √ |  |  |
| 11.   Was appropriate statistical analysis used? | √ |  |  |  |
| Total | 6 | 4 | 1 |  |
| 2. Massoure et al 2013 |  |  |  |  |
| JBI checklist for cohort studies | **Yes** | **No** | **Unclear** | **Not applicable** |
| 1.       Were the two groups similar and recruited from the same population? |  |  | √ |  |
| 2.       Were the exposures measured similarly to assign people to both exposed and unexposed groups? |  |  | √ |  |
| 3.       Was the exposure measured in a valid and reliable way? | √ |  |  |  |
| 4.       Were confounding factors identified? | √ |  |  |  |
| 5.       Were strategies to deal with confounding factors stated? | √ |  |  |  |
| 6.       Were the groups/participants free of the outcome at the start of the study (or at the moment of exposure)? |  |  | √ |  |
| 7.       Were the outcomes measured in a valid and reliable way? | √ |  |  |  |
| 8.       Was the follow up time reported and sufficient to be long enough for outcomes to occur? | √ |  |  |  |
| 9.       Was follow up complete, and if not, were the reasons to loss to follow up described and explored? | √ |  |  |  |
| 10.   Were strategies to address incomplete follow up utilized? | √ |  |  |  |
| 11.   Was appropriate statistical analysis used? | √ |  |  |  |
| Total | 8 |  | 3 |  |
| 3. Puri et al 2013 |  |  |  |  |
| JBI checklist for cohort studies | **Yes** | **No** | **Unclear** | **Not applicable** |
| 1.       Were the two groups similar and recruited from the same population? |  |  | √ |  |
| 2.       Were the exposures measured similarly to assign people to both exposed and unexposed groups? |  |  | √ |  |
| 3.       Was the exposure measured in a valid and reliable way? | √ |  |  |  |
| 4.       Were confounding factors identified? |  | √ |  |  |
| 5.       Were strategies to deal with confounding factors stated? |  | √ |  |  |
| 6.       Were the groups/participants free of the outcome at the start of the study (or at the moment of exposure)? |  |  | √ |  |
| 7.       Were the outcomes measured in a valid and reliable way? | √ |  |  |  |
| 8.       Was the follow up time reported and sufficient to be long enough for outcomes to occur? | √ |  |  |  |
| 9.       Was follow up complete, and if not, were the reasons to loss to follow up described and explored? | √ |  |  |  |
| 10.   Were strategies to address incomplete follow up utilized? | √ |  |  |  |
| 11.   Was appropriate statistical analysis used? | √ |  |  |  |
| Total | 6 | 2 | 3 |  |
| 4. Otaigbe et al 2014 |  |  |  |  |
| JBI checklist for cohort studies | **Yes** | **No** | **Unclear** | **Not applicable** |
| 1.       Were the two groups similar and recruited from the same population? |  |  | √ |  |
| 2.       Were the exposures measured similarly to assign people to both exposed and unexposed groups? |  |  | √ |  |
| 3.       Was the exposure measured in a valid and reliable way? | √ |  |  |  |
| 4.       Were confounding factors identified? | √ |  |  |  |
| 5.       Were strategies to deal with confounding factors stated? |  | √ |  |  |
| 6.       Were the groups/participants free of the outcome at the start of the study (or at the moment of exposure)? |  |  | √ |  |
| 7.       Were the outcomes measured in a valid and reliable way? | √ |  |  |  |
| 8.       Was the follow up time reported and sufficient to be long enough for outcomes to occur? | √ |  |  |  |
| 9.       Was follow up complete, and if not, were the reasons to loss to follow up described and explored? |  | √ |  |  |
| 10.   Were strategies to address incomplete follow up utilized? | √ |  |  |  |
| 11.   Was appropriate statistical analysis used? | √ |  |  |  |
| Total | 6 | 2 | 3 |  |
| 5. Zuechner et al 2019 |  |  |  |  |
| JBI checklist for cohort studies | **Yes** | **No** | **Unclear** | **Not applicable** |
| 1.       Were the two groups similar and recruited from the same population? | √ |  |  |  |
| 2.       Were the exposures measured similarly to assign people to both exposed and unexposed groups? |  |  | √ |  |
| 3.       Was the exposure measured in a valid and reliable way? | √ |  |  |  |
| 4.       Were confounding factors identified? |  | √ |  |  |
| 5.       Were strategies to deal with confounding factors stated? |  | √ |  |  |
| 6.       Were the groups/participants free of the outcome at the start of the study (or at the moment of exposure)? |  |  | √ |  |
| 7.       Were the outcomes measured in a valid and reliable way? | √ |  |  |  |
| 8.       Was the follow up time reported and sufficient to be long enough for outcomes to occur? | √ |  |  |  |
| 9.       Was follow up complete, and if not, were the reasons to loss to follow up described and explored? | √ |  |  |  |
| 10.   Were strategies to address incomplete follow up utilized? |  | √ |  |  |
| 11.   Was appropriate statistical analysis used? | √ |  |  |  |
| Total | 6 | 3 | 2 |  |
| 6. Bannerman et al 2020 |  |  |  |  |
| JBI checklist for cohort studies | **Yes** | **No** | **Unclear** | **Not applicable** |
| 1.       Were the two groups similar and recruited from the same population? | √ |  |  |  |
| 2.       Were the exposures measured similarly to assign people to both exposed and unexposed groups? |  |  | √ |  |
| 3.       Was the exposure measured in a valid and reliable way? | √ |  |  |  |
| 4.       Were confounding factors identified? | √ |  |  |  |
| 5.       Were strategies to deal with confounding factors stated? |  | √ |  |  |
| 6.       Were the groups/participants free of the outcome at the start of the study (or at the moment of exposure)? |  |  | √ |  |
| 7.       Were the outcomes measured in a valid and reliable way? | √ |  |  |  |
| 8.       Was the follow up time reported and sufficient to be long enough for outcomes to occur? | √ |  |  |  |
| 9.       Was follow up complete, and if not, were the reasons to loss to follow up described and explored? | √ |  |  |  |
| 10.   Were strategies to address incomplete follow up utilized? |  | √ |  |  |
| 11.   Was appropriate statistical analysis used? | √ |  |  |  |
| Total | 7 | 2 | 2 |  |
