# Supplementary material for: Pooled prevalence and subgroup variations of Tetralogy of Fallot among children and adolescents with congenital heart defect in Sub-Saharan Africa: A systematic review and meta-analysis
Source: PLoS One. 2025 Jan 17;20(1):e0311686. doi: 10.1371/journal.pone.0311686 (PMC11741593; doi:10.1371/journal.pone.0311686)
Supplement: S1 File — MeSH and keywords employed in different databases. (DOCX) [file pone.0311686.s006.docx]

**Keywords used in Google Scholar, Scopus and African Index Medicus**

([Prevalence OR proportion OR Pattern OR Epidemiology OR Occurrence OR Frequency OR Magnitude OR Burden AND Congenital anomal* OR Birth defect OR Birth anomal* OR Congenital abnormalit* OR Congenital Heart disease OR CHD OR Cardiac congenital anomaly OR Cyanotic heart disease OR Tetralogy of fallot OR TOF AND Sub Sahara* Africa OR Angola OR Benin OR Botswana OR Burkina Faso OR Burundi OR Cameroon OR Cape Verde OR Central African Republic OR Chad OR Comoros OR Congo Brazzaville OR Congo Democratic Republic OR Côte d'Ivoire OR Djibouti OR Equatorial Guinea OR Eritrea OR Ethiopia OR Gabon OR Gambia OR Ghana OR Guinea OR Guinea-Bissau OR Kenya OR Lesotho OR Liberia OR Madagascar OR Malawi OR Mali OR Mauritania OR Mauritius OR Mozambique OR Namibia OR Niger OR Nigeria OR Rwanda OR Sao Tome and Principe OR Senegal OR Seychelles OR Sierra Leone OR Somalia OR South Africa OR Sudan OR Swaziland OR Tanzania OR Togo OR Uganda OR Western Sahara OR Zambia OR Zimbabwe]).

**MeSH and keyword combinations used in Medline/PubMed**

(["Prevalence"[Mesh] OR Prevalence OR Proportion OR Pattern OR Epidemiology OR Occurrence OR Frequency OR Magnitude OR Burden AND "Congenital Abnormalities"[Mesh] OR "Heart Defects, Congenital"[Mesh] OR "Tetralogy of Fallot"[Mesh] OR Congenital anomal* OR Birth defect OR Birth anomal* OR Congenital abnormalit* OR Congenital Heart disease OR CHD OR Cardiac congenital anomaly OR Cyanotic heart disease OR Tetralogy of fallot OR TOF AND "Africa South of the Sahara"[Mesh] OR Sub Sahara* Africa OR Angola OR Benin OR Botswana OR Burkina Faso OR Burundi OR Cameroon OR Cape Verde OR Central African Republic OR Chad OR Comoros OR Congo Brazzaville OR Congo Democratic Republic OR Côte d'Ivoire OR Djibouti OR Equatorial Guinea OR Eritrea OR Ethiopia OR Gabon OR Gambia OR Ghana OR Guinea OR Guinea-Bissau OR Kenya OR Lesotho OR Liberia OR Madagascar OR Malawi OR Mali OR Mauritania OR Mauritius OR Mozambique OR Namibia OR Niger OR Nigeria OR Rwanda OR Sao Tome and Principe OR Senegal OR Seychelles OR Sierra Leone OR Somalia OR South Africa OR Sudan OR Swaziland OR Tanzania OR Togo OR Uganda OR Western Sahara OR Zambia OR Zimbabwe])
